# Supplementary material for: Antiviral and clinical activity of bamlanivimab in a randomized trial of non-hospitalized adults with COVID-19
Source: Nat Commun. 2022 Aug 22;13:4931. doi: 10.1038/s41467-022-32551-2 (PMC9395368; doi:10.1038/s41467-022-32551-2)
Supplement: Supplementary file 1 — Supplementary Information [file 41467_2022_32551_MOESM1_ESM.pdf]

# Bamlanivimab reduces nasopharyngeal SARS-CoV-2 RNA levels in a randomized trial of non-hospitalized adults with COVID-19

**Supplementary Tables 1-10**

**Supplementary Figures 1-7**

**Supplementary Methods**

**Supplementary Notes**

**Supplementary References**

**Additional Supplementary Methods: Protocol and Statistical Analysis Plan**

**Supplementary Table 1. Characteristics for higher risk for COVID-19 progression met by study participants, by dose cohort and treatment arm.**

| High risk characteristic | 7000 mg dose cohort | | | 700 mg dose cohort | | |
|---|---|---|---|---|---|---|
| | Bamlanivimab (N=48) | Placebo (N=46) | Total (N=94) | Bamlanivimab (N=111) | Placebo (N=112) | Total (N=223) |
| Age 55 years or older, n (%) | 18 (37.5) | 11 (23.9) | 29 (30.9) | 27 (24.3) | 31 (27.7) | 58 (26.0) |
| Chronic lung disease, n (%) | 3 (6.3) | 1 (2.2) | 4 (4.3) | 3 (2.7) | 3 (2.7) | 6 (2.7) |
| Moderate to severe asthma, n (%) | 1 (2.1) | 3 (6.5) | 4 (4.3) | 4 (3.6) | 3 (2.7) | 7 (3.1) |
| Obesity (body mass index [BMI] >35 $kg/m^2$), n (%) | 6 (12.5) | 5 (10.9) | 11 (11.7) | 22 (19.8) | 14 (12.5) | 36 (16.1) |
| Hypertension, n (%) | 14 (29.2) | 10 (21.7) | 24 (25.5) | 26 (23.4) | 34 (30.4) | 60 (26.9) |
| Cardiovascular disease (including history of stroke), n (%) | 4 (8.3) | 1 (2.2) | 5 (5.3) | 10 (9.0) | 11 (9.8) | 21 (9.4) |
| Diabetes, n (%) | 5 (10.4) | 5 (10.9) | 10 (10.6) | 13 (11.7) | 10 (8.9) | 23 (10.3) |
| Chronic kidney disease, n (%) | 0 | 0 | 0 | 3 (2.7) | 3 (2.7) | 6 (2.7) |
| Chronic liver disease, n (%) | 1 (2.1) | 0 | 1 (1.1) | 0 | 3 (2.7) | 3 (1.3) |

BMI = body mass index

**Supplementary Table 2. Symptoms present within 48 hours of study entry, by dose cohort and treatment arm.**

| Symptom | 7000 mg dose cohort | | | 700 mg dose cohort | | |
|---|---|---|---|---|---|---|
| | **Bamlanivimab (N=48)** | **Placebo (N=46)** | **Total (N=94)** | **Bamlanivimab (N=111)** | **Placebo (N=112)** | **Total (N=223)** |
| Subjective fever or feeling feverish, n (%) | 13 (27.1) | 16 (34.8) | 29 (30.9) | 46 (41.4) | 40 (35.7) | 86 (38.6) |
| Shortness of breath or difficulty breathing at rest or with activity, n (%) | 17 (35.4) | 17 (37.0) | 34 (36.2) | 42 (37.8) | 34 (30.4) | 76 (34.1) |
| Nasal Discharge, n (%) | 19 (39.6) | 17 (37.0) | 36 (38.3) | 43 (38.7) | 48 (42.9) | 91 (40.8) |
| Cough, n (%) | 34 (70.8) | 34 (73.9) | 68 (72.3) | 81 (73.0) | 73 (65.2) | 154 (69.1) |
| Chills, n (%) | 13 (27.1) | 18 (39.1) | 31 (33.0) | 38 (34.2) | 46 (41.1) | 84 (37.7) |
| Headache, n (%) | 28 (58.3) | 32 (69.6) | 60 (63.8) | 60 (54.1) | 66 (58.9) | 126 (56.5) |
| Sore throat, n (%) | 17 (35.4) | 11 (23.9) | 28 (29.8) | 35 (31.5) | 34 (30.4) | 69 (30.9) |
| Body pain or muscle pain/aches, n (%) | 27 (56.3) | 25 (54.3) | 52 (55.3) | 65 (58.6) | 68 (60.7) | 133 (59.6) |
| Fatigue, n (%) | 31 (64.6) | 27 (58.7) | 58 (61.7) | 72 (64.9) | 78 (69.6) | 150 (67.3) |
| Diarrhea, n (%) | 11 (22.9) | 12 (26.1) | 23 (24.5) | 25 (22.5) | 23 (20.5) | 48 (21.5) |
| Nausea or vomiting, n (%) | 6 (12.5) | 10 (21.7) | 16 (17.0) | 18 (16.2) | 22 (19.6) | 40 (17.9) |
| Nasal obstruction or congestion, n (%) | 22 (45.8) | 21 (45.7) | 43 (45.7) | 56 (50.5) | 53 (47.3) | 109 (48.9) |
| Loss of taste or smell, n (%) | 26 (54.2) | 26 (56.5) | 52 (55.3) | 53 (47.7) | 46 (41.1) | 99 (44.4) |
| Temperature ≥38°C (100.4°F), n (%) | 1 (2.1) | 5 (10.9) | 6 (6.4) | 11 (9.9) | 14 (12.5) | 25 (11.2) |

## Supplementary Table 3. Adverse events (AEs) through week 24

| | 7000 mg dose cohort | | 700 mg dose cohort | |
|---|---|---|---|---|
| **Event** | **Bamlanivimab (n=48)** | **Placebo (n=46)** | **Bamlanivimab (n=111)** | **Placebo (n=112)** |
| Grade 3 or higher TEAEs through week 24, number of participants (%) | 7 (14.6) | 6 (13.0) | 12 (10.8) | 11 (9.8) |
| Grade 2 or higher TEAEs through week 24, number of participants (%) | 23 (47.9) | 18 (39.1) | 55 (49.5) | 40 (35.7) |
| AESI through week 24, number of participants (%) | 1 (2.1) | 2 (4.3) | 1 (0.9) | 3 (2.7) |
| SAEs through week 24, number of participants (%) | 2 (4.2) | 4 (8.7) | 5 (4.5) | 4 (3.6) |

TEAE = treatment emergent adverse event; AESI = adverse event of special interest (infusion-related reactions and hypersensitivity reactions); SAEs = serious adverse events

**Supplementary Table 4. Detailed summary of grade 3 and higher treatment-emergent adverse events (TEAEs) through Day 28.**

| **Adverse events according to preferred term**[a] | **7000 mg dose cohort** | | **700 mg dose cohort** | |
|---|---|---|---|---|
| | **Bamlanivimab (n=48)** | **Placebo (n=46)** | **Bamlanivimab (n=111)** | **Placebo (n=112)** |
| | Number of participants (%) | | | |
| Abdominal pain | 1 (2.1) | 0 | 0 | 0 |
| Acute kidney injury | 0 | 0 | 1 (0.9) | 0 |
| Alanine aminotransferase increased | 0 | 0 | 1 (0.9) | 0 |
| Aspartate aminotransferase increased | 0 | 1 (2.2) | 0 | 0 |
| Atrial fibrillation | 0 | 0 | 2 (1.8)[b] | 0 |
| Blood creatinine decreased | 0 | 0 | 1 (0.9) | 0 |
| Blood creatinine increased | 1 (2.1) | 0 | 0 | 1 (0.9) |
| Blood glucose increased | 0 | 0 | 3 (2.7) | 0 |
| Blood pressure increased | 1 (2.1) | 0 | 0 | 0 |
| COVID-19 | 0 | 0 | 0 | 1 (0.9)[b] |
| COVID-19 pneumonia | 0 | 2 (4.3)[b] | 0 | 1 (0.9)[b] |
| Catheter site infection | 1 (2.1)[b] | 0 | 0 | 0 |
| Diabetes mellitus | 0 | 1 (2.2)[b] | 0 | 0 |
| Diarrhoea | 1 (2.1) | 2 (4.3) | 0 | 0 |
| Dyspnoea | 0 | 1 (2.2) | 1 (0.9) | 0 |
| Dyspnoea exertional | 0 | 0 | 0 | 1 (0.9) |
| Fatigue | 0 | 0 | 2 (1.8) | 2 (1.8) |
| Haemoglobin decreased | 0 | 0 | 0 | 1 (0.9) |
| Hypertension | 1 (2.1) | 0 | 1 (0.9) | 1 (0.9) |
| Hypoxia | 0 | 0 | 0 | 1 (0.9)[b] |
| Infusion related reaction | 1 (2.1)[b] | 0 | 0 | 0 |
| Insomnia | 0 | 0 | 1 (0.9) | 0 |
| Lymphocyte count decreased | 0 | 1 (2.2) | 0 | 0 |
| Migraine | 0 | 1 (2.2)[b] | 0 | 0 |
| Myelosuppression | 0 | 0 | 1 (0.9)[b] | 0 |
| Nausea | 0 | 1 (2.2) | 0 | 0 |
| Pneumonia | 0 | 1 (2.2)[b] | 0 | 0 |
| Pyelonephritis | 0 | 0 | 0 | 1 (0.9)[b] |
| Pyrexia | 1 (2.1) | 0 | 2 (1.8) | 0 |
| Serratia bacteraemia | 0 | 1 (2.2)[b] | 0 | 0 |

[a]The preferred terms were defined according to the Medical Dictionary for Regulatory Activities Version 24.0. Events reported more than once in a given participant were counted once in the participant count.

[b]Reported as serious adverse events (SAEs). For 2 atrial fibrillation TEAEs in 700 mg bamlanivimab arm, both were reported as SAEs. In addition to SAEs reported in this table, one 700 mg dose cohort bamlanivimab recipient experienced a grade 2 hypoxia TEAE reported as an SAE.

**Supplementary Table 5. Detailed summary of grade 2 and higher treatment-emergent adverse events TEAEs through Day 28.**

| Adverse events according to preferred term[a] | 7000 mg dose cohort | | 700 mg dose cohort | |
|---|---|---|---|---|
| | Bamlanivimab (n=48) | Placebo (n=46) | Bamlanivimab (n=111) | Placebo (n=112) |
| | Number of participants (%) | | | |
| Abdominal distension | 0 | 0 | 0 | 1 (0.9) |
| Abdominal pain | 1 (2.1) | 0 | 0 | 0 |
| Activated partial thromboplastin time prolonged | 0 | 0 | 1 (0.9) | 0 |
| Acute kidney injury | 0 | 0 | 1 (0.9) | 0 |
| Adjustment disorder with anxiety | 0 | 1 (2.2) | 0 | 0 |
| Alanine aminotransferase increased | 0 | 1 (2.2) | 3 (2.7) | 4 (3.6) |
| Amnesia | 0 | 0 | 1 (0.9) | 0 |
| Arthralgia | 1 (2.1) | 0 | 1 (0.9) | 0 |
| Aspartate aminotransferase increased | 0 | 1 (2.2) | 1 (0.9) | 0 |
| Asthma | 0 | 0 | 0 | 1 (0.9) |
| Atrial fibrillation | 0 | 0 | 2 (1.8) | 0 |
| Back injury | 0 | 0 | 0 | 1 (0.9) |
| Back pain | 1 (2.1) | 0 | 1 (0.9) | 1 (0.9) |
| Bipolar I disorder | 0 | 0 | 0 | 1 (0.9) |
| Blood bilirubin increased | 0 | 0 | 1 (0.9) | 0 |
| Blood creatinine decreased | 0 | 0 | 1 (0.9) | 0 |
| Blood creatinine increased | 2 (4.2) | 2 (4.3) | 1 (0.9) | 2 (1.8) |
| Blood glucose decreased | 1 (2.1) | 1 (2.2) | 0 | 1 (0.9) |
| Blood glucose increased | 4 (8.3) | 1 (2.2) | 7 (6.3) | 5 (4.5) |
| Blood pressure increased | 1 (2.1) | 1 (2.2) | 1 (0.9) | 3 (2.7) |
| Blood sodium decreased | 0 | 0 | 0 | 1 (0.9) |
| Bronchitis viral | 0 | 0 | 1 (0.9) | 0 |
| COVID-19 | 0 | 0 | 0 | 1 (0.9) |
| COVID-19 pneumonia | 0 | 2 (4.3) | 0 | 2 (1.8) |
| Catheter site infection | 1 (2.1) | 0 | 0 | 0 |
| Chest discomfort | 0 | 0 | 1 (0.9) | 0 |
| Chills | 0 | 0 | 1 (0.9) | 1 (0.9) |
| Constipation | 1 (2.1) | 0 | 0 | 0 |
| Cough | 2 (4.2) | 0 | 2 (1.8) | 0 |
| Decreased appetite | 0 | 1 (2.2) | 0 | 0 |
| Dehydration | 0 | 1 (2.2) | 0 | 0 |
| Dermatitis | 0 | 0 | 1 (0.9) | 0 |
| Diabetes mellitus | 0 | 1 (2.2) | 0 | 0 |
| Diarrhoea | 3 (6.3) | 3 (6.5) | 1 (0.9) | 0 |
| Dissociation | 0 | 0 | 1 (0.9) | 0 |
| Disturbance in attention | 1 (2.1) | 0 | 1 (0.9) | 0 |
| Dizziness | 1 (2.1) | 0 | 1 (0.9) | 1 (0.9) |
| Dysmenorrhoea | 0 | 0 | 1 (0.9) | 0 |
| Dyspepsia | 0 | 0 | 0 | 1 (0.9) |

| | | | | |
|---|---|---|---|---|
| Dyspnoea | 2 (4.2) | 2 (4.3) | 3 (2.7) | 3 (2.7) |
| Dyspnoea exertional | 0 | 0 | 2 (1.8) | 1 (0.9) |
| Eye pain | 0 | 1 (2.2) | 0 | 0 |
| Fatigue | 4 (8.3) | 4 (8.7) | 5 (4.5) | 2 (1.8) |
| Flank pain | 0 | 0 | 0 | 1 (0.9) |
| Flushing | 0 | 0 | 0 | 1 (0.9) |
| Foot fracture | 1 (2.1) | 0 | 0 | 0 |
| Gastroenteritis Escherichia coli | 0 | 0 | 1 (0.9) | 0 |
| Gastrooesophageal reflux disease | 1 (2.1) | 0 | 2 (1.8) | 0 |
| Headache | 2 (4.2) | 4 (8.7) | 3 (2.7) | 2 (1.8) |
| Haemoglobin decreased | 1 (2.1) | 0 | 3 (2.7) | 4 (3.6) |
| Headache | 2 (4.2) | 4 (8.7) | 3 (2.7) | 2 (1.8) |
| Hepatic enzyme increased | 0 | 0 | 0 | 1 (0.9) |
| Hypersensitivity | 0 | 0 | 0 | 1 (0.9) |
| Hypertension | 2 (4.2) | 1 (2.2) | 1 (0.9) | 2 (1.8) |
| Hypoaesthesia | 0 | 0 | 1 (0.9) | 0 |
| Hypotension | 0 | 1 (2.2) | 0 | 0 |
| Hypoxia | 0 | 0 | 1 (0.9) | 1 (0.9) |
| Infusion related reaction | 1 (2.1) | 0 | 0 | 0 |
| Injection site haemorrhage | 1 (2.1) | 0 | 0 | 0 |
| Insomnia | 0 | 0 | 2 (1.8) | 0 |
| Libido decreased | 0 | 1 (2.2) | 0 | 0 |
| Lymphocyte count decreased | 0 | 1 (2.2) | 0 | 0 |
| Malaise | 1 (2.1) | 0 | 0 | 0 |
| Metabolic acidosis | 0 | 0 | 0 | 1 (0.9) |
| Migraine | 0 | 1 (2.2) | 0 | 0 |
| Muscular weakness | 0 | 0 | 0 | 1 (0.9) |
| Myalgia | 3 (6.3) | 1 (2.2) | 4 (3.6) | 0 |
| Myelosuppression | 0 | 0 | 1 (0.9) | 0 |
| Nasal congestion | 2 (4.2) | 1 (2.2) | 1 (0.9) | 2 (1.8) |
| Nasal obstruction | 0 | 0 | 0 | 2 (1.8) |
| Nausea | 4 (8.3) | 2 (4.3) | 1 (0.9) | 3 (2.7) |
| Neck pain | 0 | 0 | 0 | 1 (0.9) |
| Neutrophil count decreased | 0 | 0 | 1 (0.9) | 1 (0.9) |
| Non-cardiac chest pain | 0 | 1 (2.2) | 1 (0.9) | 1 (0.9) |
| Oropharyngeal pain | 0 | 0 | 1 (0.9) | 0 |
| Pain | 1 (2.1) | 0 | 2 (1.8) | 0 |
| Panic attack | 0 | 1 (2.2) | 0 | 0 |
| Paraesthesia | 0 | 0 | 1 (0.9) | 0 |
| Pleuritic pain | 0 | 0 | 1 (0.9) | 0 |
| Pneumonia | 0 | 3 (6.5) | 0 | 0 |
| Pneumonia bacterial | 0 | 0 | 1 (0.9) | 0 |
| Productive cough | 0 | 1 (2.2) | 0 | 0 |
| Pyelonephritis | 0 | 1 (2.2) | 0 | 1 (0.9) |
| Pyrexia | 2 (4.2) | 1 (2.2) | 7 (6.3) | 0 |
| Rhinorrhoea | 1 (2.1) | 0 | 0 | 1 (0.9) |
| Sciatica | 1 (2.1) | 0 | 0 | 0 |
| Serratia bacteraemia | 0 | 1 (2.2) | 0 | 0 |
| Sinusitis | 0 | 0 | 0 | 1 (0.9) |
| Syncope | 1 (2.1) | 0 | 2 (1.8) | 0 |

| | | | | |
|---|---|---|---|---|
| Synovial cyst | 1 (2.1) | 0 | 0 | 0 |
| Tachycardia | 0 | 0 | 0 | 1 (0.9) |
| Tooth abscess | 0 | 0 | 1 (0.9) | 0 |
| Toothache | 0 | 0 | 1 (0.9) | 0 |
| Urinary retention | 0 | 1 (2.2) | 0 | 0 |
| Urinary tract infection | 0 | 0 | 1 (0.9) | 0 |
| Vomiting | 0 | 0 | 2 (1.8) | 0 |
| Weight decreased | 1 (2.1) | 0 | 0 | 0 |

[a]The preferred terms were defined according to the Medical Dictionary for Regulatory Activities Version 24.0. Events reported more than once in a given participant were counted once in the participant count.

**Supplementary Table 6. SARS-CoV-2 variant and frequency by dose cohort and treatment arm.**

S-gene sequencing was attempted on nasopharyngeal swab samples at study entry or the earliest follow-up time point with a SARS-CoV-2 RNA level ≥2 $\log_{10}$ copies/mL. Variant calling was successful on 77 of 78 samples for 7000 mg dose cohort participants, and 207 or 208 samples for the 700 dose cohort participants. There was one sequencing failure in each placebo arm.

| | Number of participants with given virus clade | |
|---|---|---|
| | Dose cohort: 7000 mg | |
| **SARS-CoV-2 Clade** | **Bamlanivimab** | **Placebo** |
| 19A | 2 | 1 |
| 19B | 1 | 0 |
| 20A | 33 | 29 |
| 20B | 2 | 2 |
| 20C | 1 | 2 |
| 20D | 1 | 0 |
| 20G | 2 | 1 |
| | **Dose cohort: 700 mg** | |
| **SARS-CoV-2 Clade** | **Bamlanivimab** | **Placebo** |
| 19A | 10 | 11 |
| 19B | 1 | 4 |
| 20A | 72 | 73 |
| 20B | 8 | 5 |
| 20C | 4 | 7 |
| 20E (EU1) | 2 | 0 |
| 20G | 4 | 4 |
| 21C (Epsilon) | 1 | 2 |

**Supplementary Table 7. Nasopharyngeal SARS-CoV-2 RNA levels at each study day by dose cohort, treatment arm, and time from symptom onset (randomization strata, ≤5 vs >5 days).**

The assay limit of detection (LoD) was 1.4 $\log_{10}$ copies/mL, lower limit of quantification (LLoQ) was 2 $\log_{10}$ copies/mL, and upper limit of quantification (ULoQ) was 7 $\log_{10}$ copies/mL. Values below the LoD were imputed as 0.7 $\log_{10}$ copies/ml (i.e., half the distance from zero to the LoD), values above the LoD but below the LLoQ were imputed as 1.7 $\log_{10}$ copies/ml (i.e., half the distance from the LoD to the LLoQ), and values above the ULoQ were imputed as 8 $\log_{10}$ copies/ml if a numerical value was not available.

| | **700 mg dose cohort** | | | |
|---|---|---|---|---|
| **Visit** | **≤5 days from symptom onset** | | **>5 days from symptom onset** | |
| | **Bamlanivimab NP SARS-CoV-2 RNA level, Median (Q1, Q3), log10 copies/mL** | **Placebo NP SARS-CoV-2 RNA level, Median (Q1, Q3), log10 copies/mL** | **Bamlanivimab NP SARS-CoV-2 RNA level, Median (Q1, Q3), log10 copies/mL** | **Placebo NP SARS-CoV-2 RNA level, Median (Q1, Q3), log10 copies/mL** |
| Day 0 | 6.7 (5.4, 7.7) *n=44* | 6.5 (5.8, 7.8) *n=42* | 4.9 (3.6, 6.0) *N=65* | 4.8 (3.5, 6.0) *n=70* |
| Day 3 | 3.5 (2.5, 5.3) *n=44* | 4.9 (3.5, 6.2) *n=39* | 2.5 (1.7, 3.2) *n=61* | 3.4 (2.3, 4.5) *n=68* |
| Day 7 | 2.3 (1.7, 3.1) *n=44* | 2.7 (2.1, 3.5) *n=40* | 1.7 (1.7, 3.0) *n=60* | 1.7 (1.7, 2.7) *n=66* |
| Day 14 | 1.7 (0.7, 2.2) *n=39* | 1.7 (0.7, 1.7) *n=38* | 1.7 (0.7, 1.7) *n=61* | 1.7 (0.7, 1.7) *n=64* |
| Day 21 | 0.7 (0.7, 1.7) *n=43* | 1.7 (0.7, 1.7) *n=39* | 0.7 (0.7, 1.7) *n=60* | 0.7 (0.7, 1.7) *n=65* |
| Day 28 | 0.7 (0.7, 1.7) *n=39* | 0.7 (0.7, 1.7) *n=37* | 0.7 (0.7, 1.7) *n=58* | 0.7 (0.7, 1.7) *n=66* |
| | **7000 mg dose cohort** | | | |
| | **≤5 days from symptom onset** | | **>5 days from symptom onset** | |
| **Visit** | **Bamlanivimab NP SARS-CoV-2 RNA level, Median (Q1, Q3), log10 copies/mL** | **Placebo NP SARS-CoV-2 RNA level, Median (Q1, Q3), log10 copies/mL** | **Bamlanivimab NP SARS-CoV-2 RNA level, Median (Q1, Q3), log10 copies/mL** | **Placebo NP SARS-CoV-2 RNA level, Median (Q1, Q3), log10 copies/mL** |
| Day 0 | 5.8 (3.1, 7.4) *n=18* | 6.3 (2.6, 7.3) *n=23* | 4.3 (2.2, 6.2) *n=30* | 4.6 (1.7, 6.8) *n=22* |
| Day 3 | 2.3 (1.7, 4.2) *n=17* | 4.2 (2.3, 6.1) *n=22* | 2.2 (1.7, 3.6) *n=29* | 2.5 (1.7, 4.3) *n=22* |
| Day 7 | 1.7 (1.7, 2.5) *n=18* | 2.6 (1.7, 3.9) *n=22* | 1.7 (1.2, 2.2) *n=28* | 1.7 (0.7, 2.7) *n=21* |
| Day 14 | 0.7 (0.7, 1.7) *n=18* | 0.7 (0.7, 2.2) *n=21* | 1.7 (0.7, 1.7) *n=28* | 0.7 (0.7, 0.7) *n=21* |
| Day 21 | 0.7 (0.7, 0.7) *n=17* | 0.7 (0.7, 1.7) *n=19* | 0.7 (0.7, 1.7) *n=29* | 0.7 (0.7, 0.7) *n=21* |
| Day 28 | 0.7 (0.7, 0.7) *n=18* | 0.7 (0.7, 1.7) *n=21* | 0.7 (0.7, 1.7) *n=29* | 0.7 (0.7, 0.7) *n=21* |

**Supplementary Table 8. Results of modeling of nasopharyngeal (NP) and anterior nasal swab (AN) viral decay.**
(8A) Number (%) of participants with fitted nasopharyngeal (NP) and anterior nasal (AN) SARS-CoV-2 viral load data by dose cohort and treatment arm, (8B) NP and AN viral load decay rates by treatment arm and dose, and (8C) population parameter estimates for NP and AN data fitting combining 700 and 7000 mg doses (700 mg and 7000 mg dose cohort data were combined as no difference was observed in viral decay rates between the cohorts).

**8A. Number (%) of participants with sufficient data to estimate viral decay rates**

| Dose cohort | Arm | Total | Fitted AN | Fitted NP |
|---|---|---|---|---|
| **700 mg** | Bamlanivimab | 111 | 60 (54) | 66 (59) |
| | Placebo | 112 | 75 (67) | 81 (72) |
| **7000 mg** | Bamlanivimab | 48 | 26 (54) | 25 (52) |
| | Placebo | 46 | 25 (54) | 24 (52) |

**8B. Viral load decay rates ($\lambda_1$) by arm and dose cohort**

| | | Decay rate (95% CI), day$^{-1}$ | | |
|---|---|---|---|---|
| **Swab type** | **Arm** | **700 mg** | **7000 mg** | **p-value*** |
| **NP** | Bamlanivimab | 2.06 (1.74-2.43) | 1.97 (1.54-2.39) | 0.73 |
| | Placebo | 1.36 (1.24-1.49) | 1.27 (1.07-1.46) | 0.42 |
| **AN** | Bamlanivimab | 2.47 (2.20-2.77) | 2.66 (2.21-3.12) | 0.43 |
| | Placebo | 2.06 (1.84-2.31) | 2.49 (2.03-2.96) | 0.08 |

CI = confidence interval, *two-sided Wald chi-square test

**8C. Faster first phase of viral decay with bamlanivimab compared to placebo in both NP and AN models**

| Parameter | AN | NP |
|---|---|---|
| $V_0$ ($\log_{10}$) (95% CI) | 5.85 (5.65-6.05) | 6.26 (6.07-6.45) |
| A (95% CI) | 1 (-) | 0.96 (0.91-0.99) |
| $\lambda_1$ (Placebo) (day$^{-1}$) (95% CI) | 2.12 (1.94-2.33) | 1.62 (1.45-1.80) |
| $\lambda_1$ (Bamlanivimab) (day$^{-1}$) (95% CI) | 2.55 (2.20-2.80) | 2.32 (1.92-2.71) |
| p-value* for $\lambda_1$ | 0.0049 | 0.0002 |
| $\lambda_2$ (day$^{-1}$) (95% CI) | (-) | 1.10 (0.95-1.27) |

*two-sided Wald chi-square test

**Supplementary Table 9. Hospitalizations and deaths through day 28 and through week 24 by dose cohort and treatment arm.**

| | 7000 mg dose group | | | 700 mg dose cohort | | |
|---|---|---|---|---|---|---|
| **Event** | **Bamlanivimab (n=48)** | **Placebo (n=46)** | **Cumulative proportion ratio death + hospitalization (bamlanivimab 7000 mg vs placebo) (95% CI), p-value[a]** | **Bamlanivimab (n=111)** | **Placebo (n=112)** | **Cumulative proportion ratio death + hospitalization (bamlanivimab 700 mg vs placebo) (95% CI), p-value[a]** |
| Hospitalizations through day 28, n (%) | 2 (4.2) | 4 (8.7) | 0.48 (0.09, 2.50), p=0.38 | 4 (3.6) | 4 (3.6) | 1.02 (0.26, 3.99), p=0.97 |
| Deaths through day 28, n (%) | 0 | 0 | | 0 | 0 | |
| Hospitalizations through week 24, n (%) | 2 (4.2) | 4 (8.7) | 0.48 (0.09, 2.50), p=0.38 | 5 (4.5) | 5 (4.5) | 1.02 (0.30, 3.4), p=0.97 |
| Deaths through week 24, n (%) | 0 | 0 | | 0 | 0 | |

CI = confidence interval

[a]asymptotic Normal test for the log-transformed proportion difference

**Supplementary Table 10. Bamlanivimab Pharmacokinetic Characteristics**

| | Cmax (µg/mL) | $AUC_{0-\infty}$ (hr*µg/mL) | Half-Life (days) | C28d (µg/mL) | CL (L/day) | Age (years) | Weight (kg) |
|---|---|---|---|---|---|---|---|
| **All Participants, 700 mg** | | | | | | | |
| N | 71 | 70 | 70 | 70 | 70 | 71 | 71 |
| Mean | 205.94 | 68288 | 18.15 | 29.37 | 0.278 | 45.7 | 88.2 |
| SD | 116.46 | 23156 | 5.58 | 9.44 | 0.113 | 14.4 | 22.7 |
| CV | 56.6% | 33.9% | 30.7% | 32.1% | 40.5% | 31.5% | 25.8% |
| Median | 201.21 | 67841 | 17.82 | 28.79 | 0.240 | 47.0 | 86.4 |
| Min | 40.81 | 23572 | 4.10 | BLQ | 0.120 | 18.0 | 44.8 |
| Max | 962.08 | 133777 | 32.92 | 53.90 | 0.720 | 86.0 | 182.3 |
| **All Participants, 7000 mg** | | | | | | | |
| N | 37 | 37 | 37 | 36 | 37 | 37 | 37 |
| Mean | 1876.15 | 613290 | 23.64 | 235.51 | 0.374 | 47.4 | 82.2 |
| SD | 907.42 | 262859 | 18.34 | 127.79 | 0.333 | 15.3 | 13.6 |
| CV | 48.4% | 42.9% | 77.6% | 54.3% | 88.9% | 32.3% | 16.6% |
| Median | 2131.17 | 647535 | 18.93 | 234.48 | 0.264 | 46.0 | 83.6 |
| Min | 42.62 | 82331 | 8.75 | 25.94 | 0.120 | 21.0 | 58.4 |
| Max | 3251.32 | 1402569 | 114.86 | 640.77 | 2.040 | 79.0 | 109.3 |

Cmax, maximum concentration; C28d, concentration at day 28 post dose; $AUC_{0-\infty}$, area under the concentration-time curve from time 0 to infinity; CL, total body clearance; BLQ, below the limit of quantitation; SD, standard deviation; CV, coefficient of variation.

**Supplementary Fig. 1. Nasopharyngeal (NP) SARS-CoV-2 RNA levels (viral loads) by dose cohort, treatment arm, and visit, stratified by time from symptom onset at study entry (≤5 vs >5 days).**
Randomization to bamlanivimab or placebo in each dose cohort was stratified on time from symptom onset. Larger differences between bamlanivimab (represented by red) and placebo (represented by blue) arms were observed at day 3 among participants treated within 5 days of symptom onset than those treated >5 days from symptom onset. The lower limit of detection was 1.4 $\log_{10}$ copies/mL. Presented are median values with error bars for interquartile ranges (IQRs), and individual participant values as dots. Numerical values are provided in Supplementary Table 7. Source data for summary measures are provided as a Source Data file.

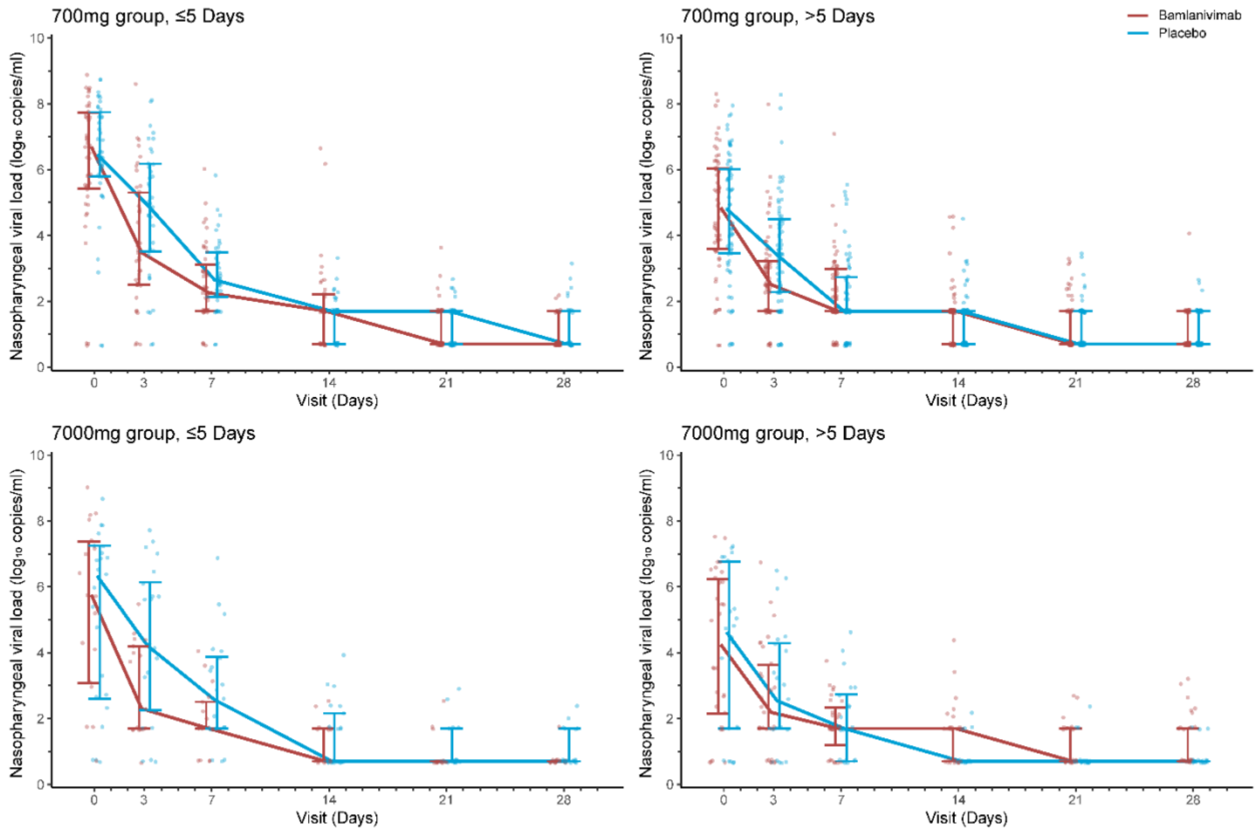

**Supplementary Fig. 2. Fitting of nasopharyngeal viral loads.**
The best fits to the decay in viral load in the nasopharyngeal (NP) samples are biexponential. The data (circles) and best fit (lines) for all the fitted participants (each participant shown as a figure) both in the (A) bamlanivimab (green circles) and (B) placebo (blue) arms. Both dose cohorts (700 mg and 7000 mg) were fitted together and are not distinguished in these fits. Pink symbols represent data at or below the lower limit of quantification (LLoQ) and red symbols data at or below the limit of detection (LoD).

**(A)**

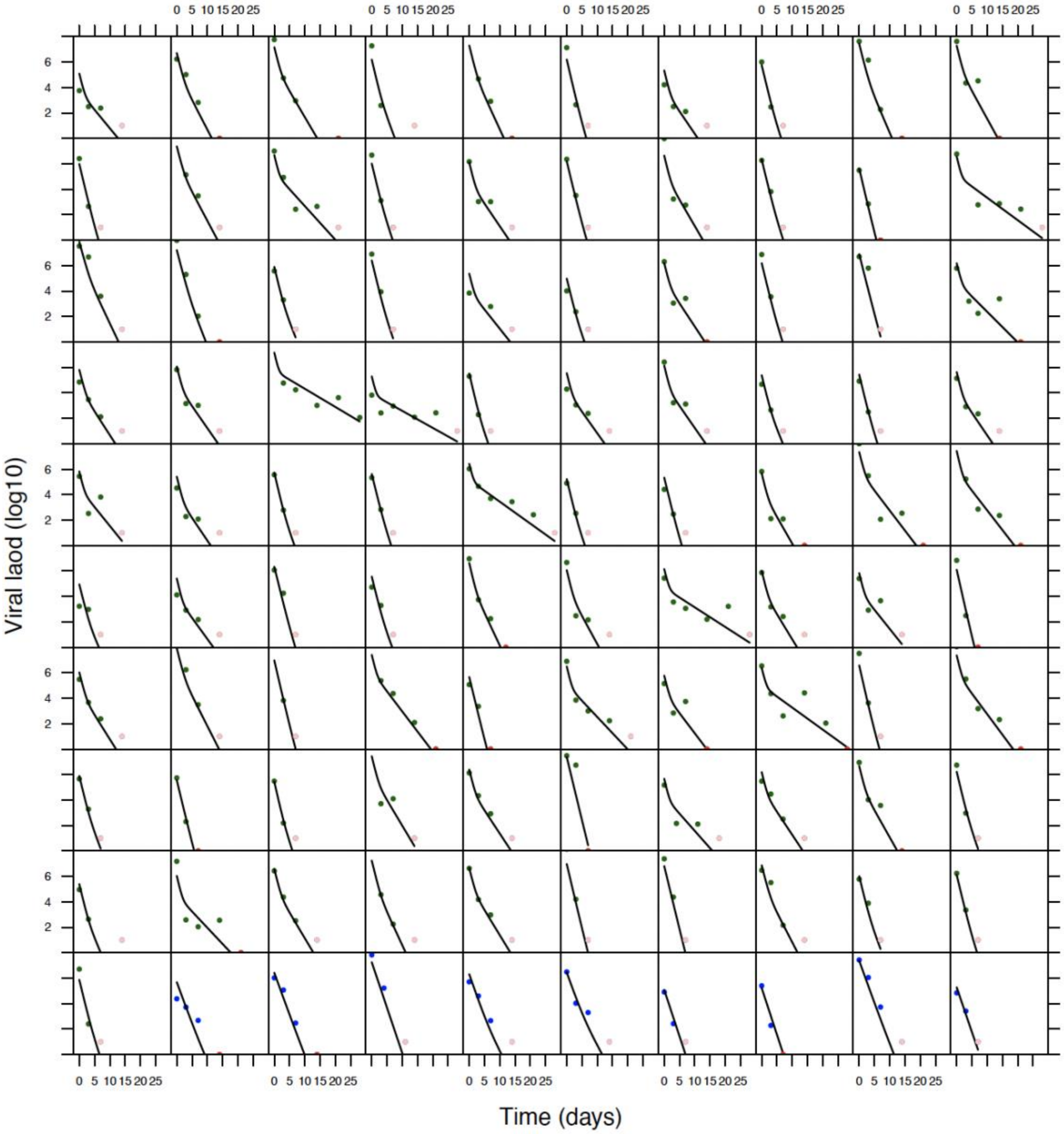

**(B)**

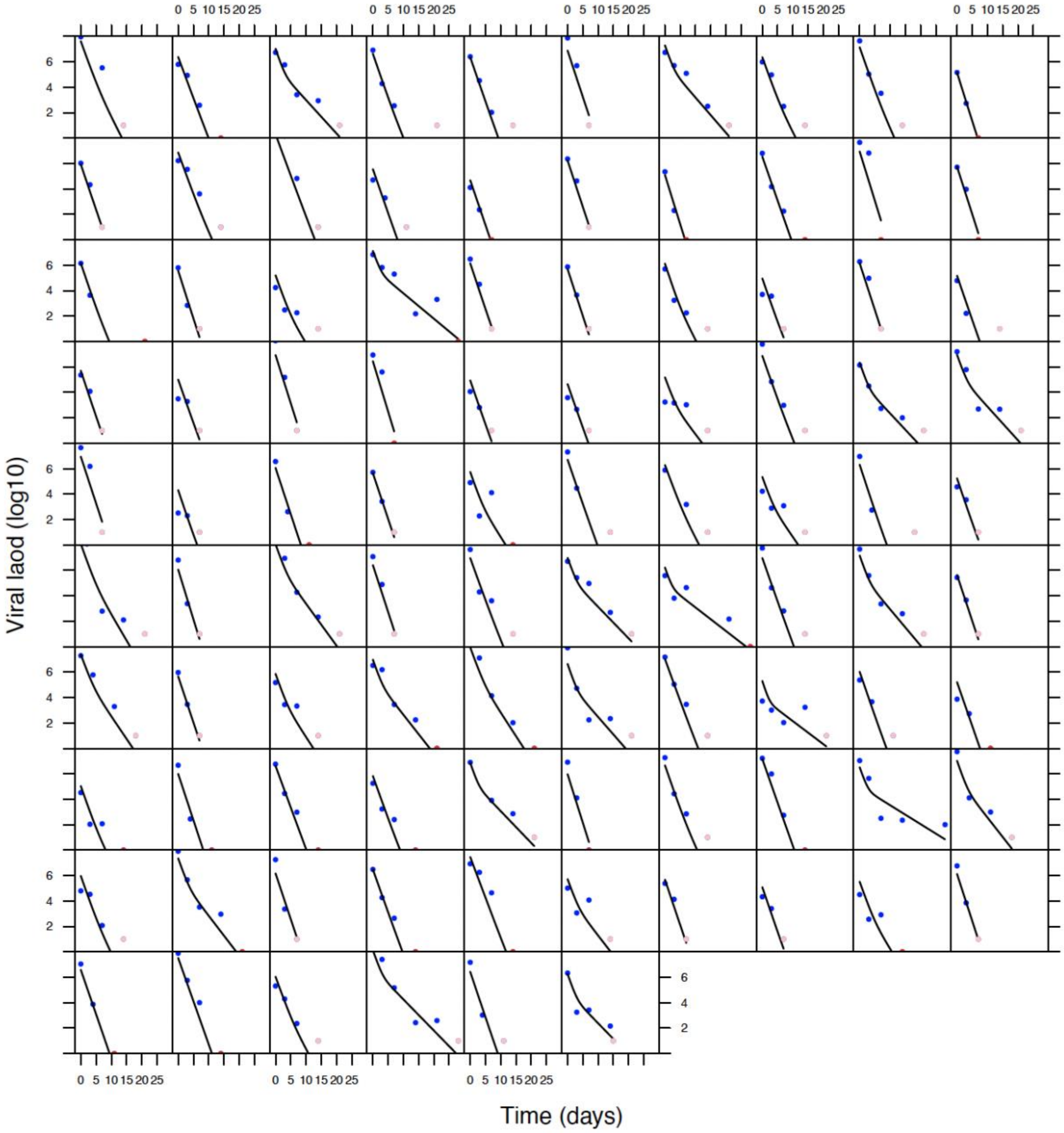

**Supplementary Fig. 3. Fitting of anterior nasal viral loads.**
The best fits to the decay in viral load in the AN samples are a single exponential. The data (circles) and best fit (lines) for all the fitted participants (each participant shown as a figure) both in the (A) bamlanivimab (green circles) and (B) placebo (blue) arms. Both dose cohorts (700 mg and 7000 mg) were fitted together and are not distinguished in these fits. Pink symbols represent data at or below the lower limit of quantification (LLoQ) and red symbols data at or below the limit of detection (LoD).

**(A)**

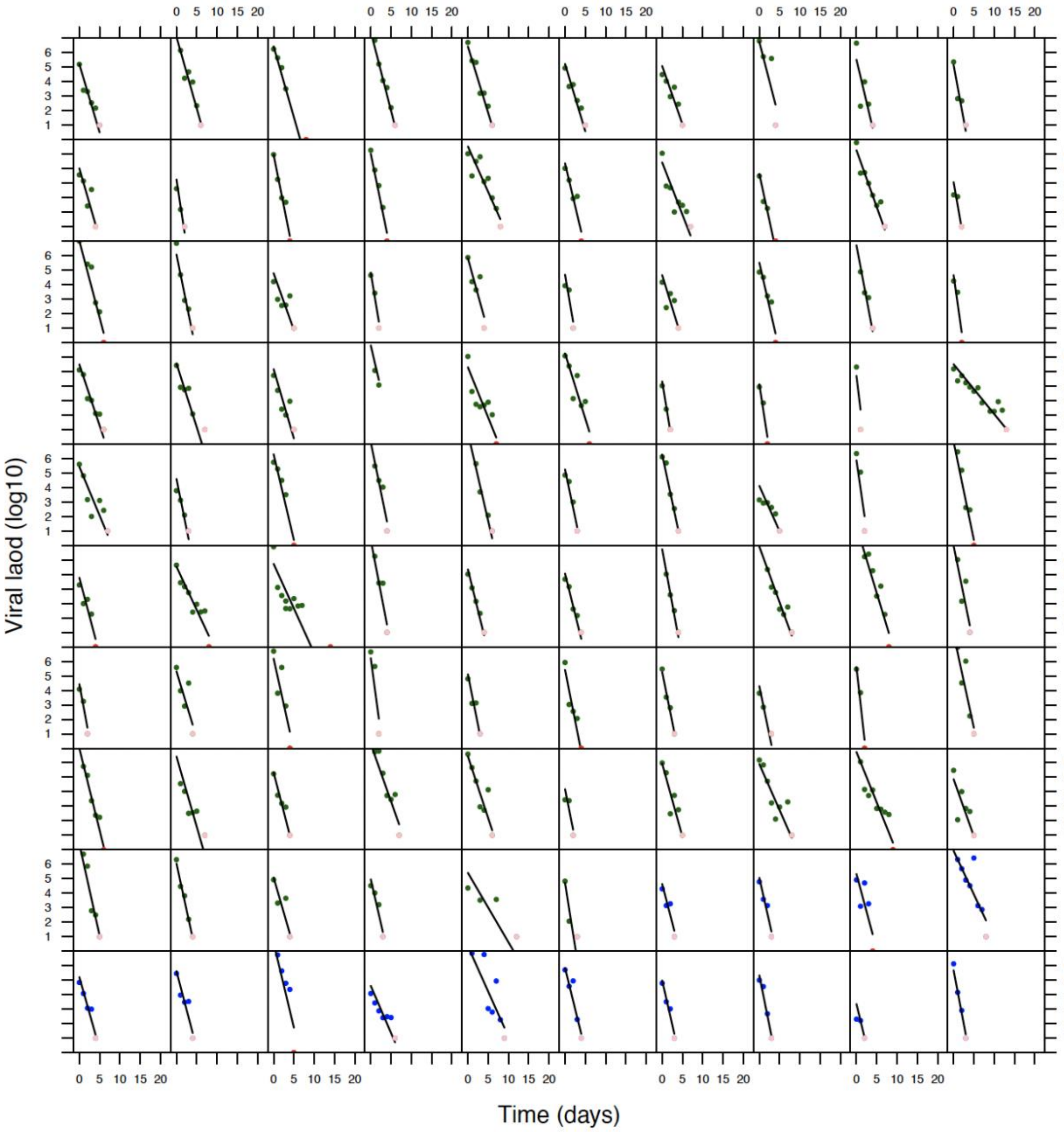

**(B)**

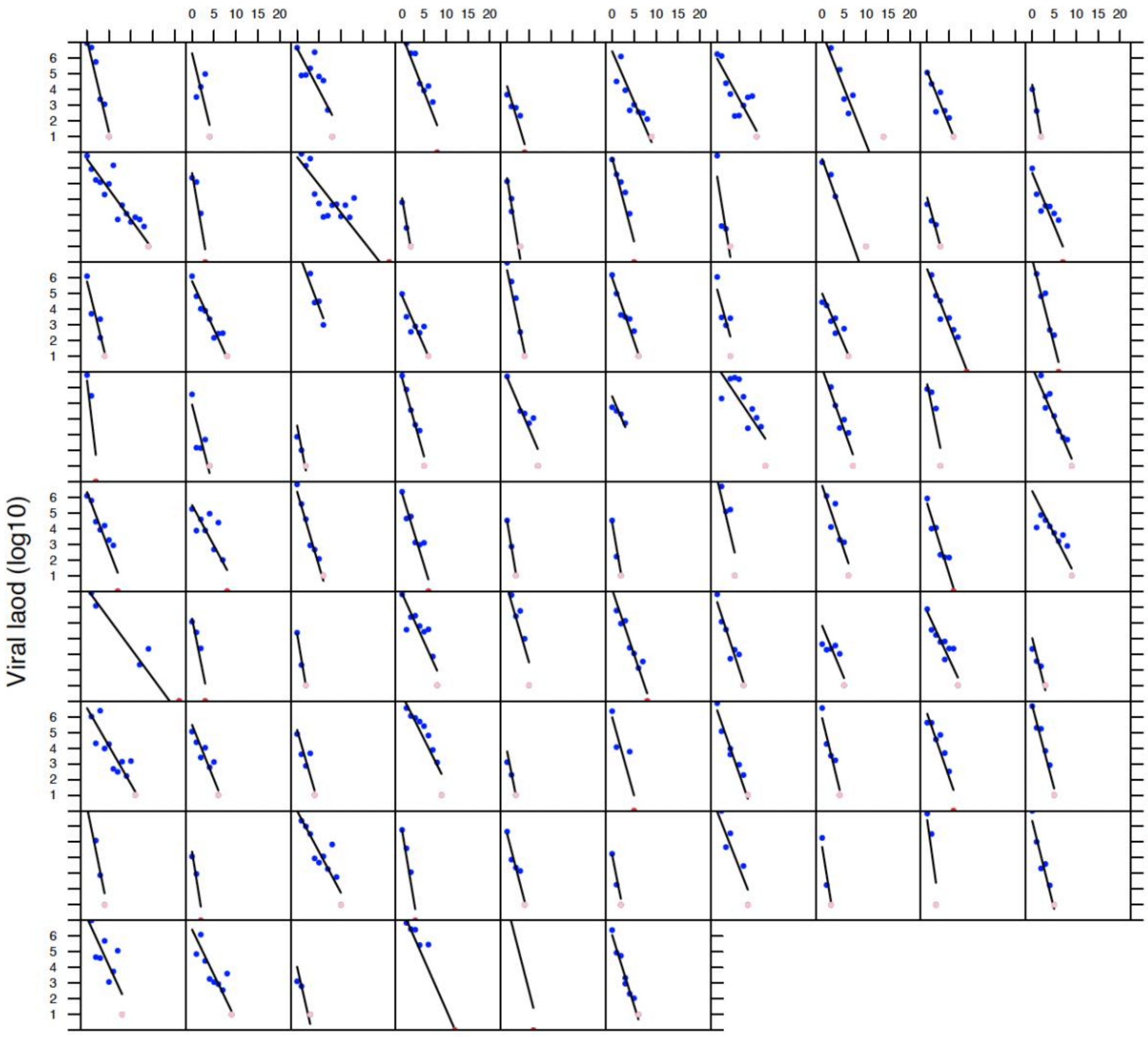

**Supplementary Fig. 4. Viral decay modeling comparing nasopharyngeal (NP) and anterior nasal (AN) SARS-CoV-2 RNA decay rates among participants who were treated within 5 days vs >5 days from symptom onset.**
700 and 7000 mg dose cohort data were combined and bamlanivimab and placebo participants analyzed separately. (4A) Comparison of population parameter estimates for NP data fitting and (4B) AN data fitting. In the boxplots, the horizontal line represents the median and the box limits represent the first and third quartiles (Q1 and Q3). The whiskers extend 1.5 times the interquartile range, except if that extension would go past the minimum or maximum of the data, in which case the whiskers extend to that value. Extreme values outside the range of the whiskers are represented individually as circles. Source Data for the boxplots are provided as a Source Data file.

**4A.** Comparison of the rate of 1st phase decay (top) and rate of 2nd phase decay (bottom) by time from symptom onset at study entry for the NP dataset, for placebo participants (left, n=46 “<=5 days” and n=59 “>5 days”) and bamlanivimab 700 and 7000 mg treated participants (right, n=42 “<=5 days” and n=49 “>5 days”). P-values based on two-sided t-tests.

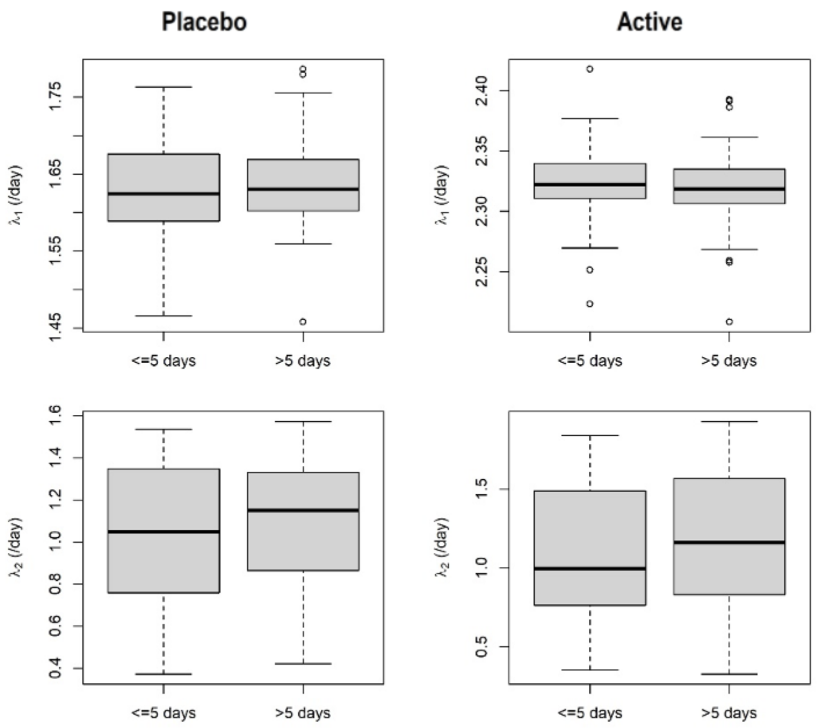


| Parameter | Time from symptom onset at study entry | | p-value |
|---|---|---|---|
| | **≤ 5 days** | **>5 days** | |
| ***Bamlanivimab*** | | | |
| Rate of 1st phase decay, $\lambda_1$ (day$^{-1}$) (95% CI) | 2.32 (2.31 – 2.33) | 2.32 (2.31 – 2.33) | 0.52 |
| Rate of 2nd phase decay, $\lambda_2$ (day$^{-1}$) (95% CI) | 1.11 (0.99 – 1.24) | 1.16 (1.03 – 1.28) | 0.60 |
| ***Placebo*** | | | |
| Rate of 1st phase decay, $\lambda_1$ (day$^{-1}$) (95% CI) | 1.63 (1.61 – 1.65) | 1.64 (1.63 – 1.66) | 0.53 |
| Rate of 2nd phase decay, $\lambda_2$ (day$^{-1}$) (95% CI) | 1.04 (0.94 – 1.14) | 1.08 (1.00 – 1.16) | 0.51 |

**4B.** Comparison of the rate of decay by time from symptom onset at study entry for the AN dataset, for placebo participants (left, n=52 “<=5 days” and n=48 “>5 days” ) and bamlanivimab 700 and 7000 mg treated participants (right, n=40 “<=5 days” and n=46 “>5 days” ). P-values based on two-sided t-tests.

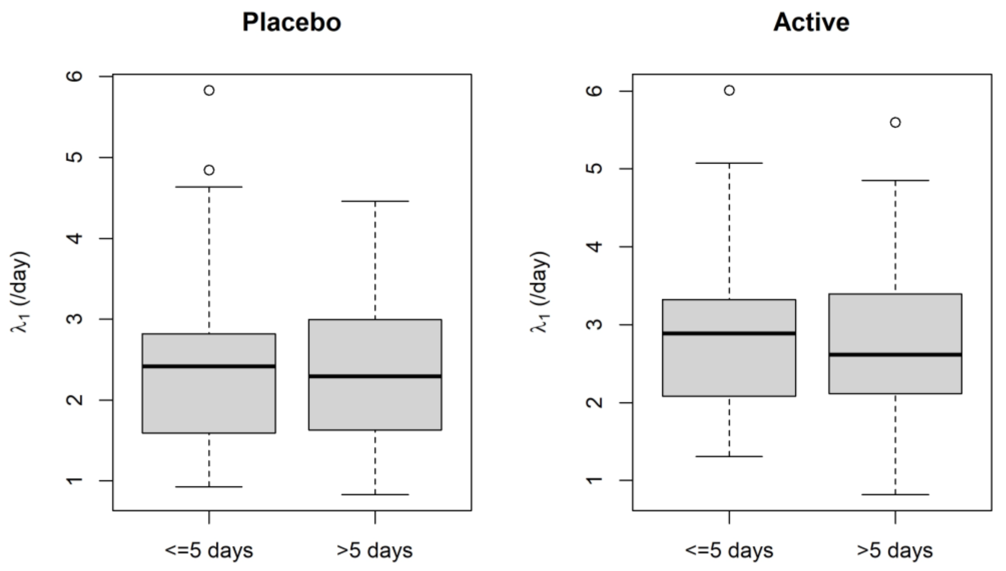


| Parameter | Time from symptom onset at study entry | | p-value |
|---|---|---|---|
| | **≤ 5 days** | **>5 days** | |
| ***Bamlanivimab*** | | | |
| Rate of decay, $\lambda_1$ (day$^{-1}$) (95% CI) | 2.88 (2.55 – 3.20) | 2.83 (2.53 – 3.13) | 0.48 |
| ***Placebo*** | | | |
| Rate of decay, $\lambda_1$ (day$^{-1}$) (95% CI) | 2.46 (2.16 – 2.77) | 2.37 (2.12 – 2.62) | 0.66 |

**Supplementary Fig. 5. Associations between baseline virology and symptom scores and comparisons by subgroups, combining bamlanivimab 700 and 7000 mg dose cohorts.** (A) Baseline nasopharyngeal (NP) and anterior nasal (AN) SARS-CoV-2 RNA levels (viral loads) were highly correlated. The diagonal line indicates line of equality. Participants that received bamlanivimab 700 mg are represented by red dots, bamlanivimab 7000 mg by orange dots, placebo for 700 mg by light blue dots, and placebo for 7000 mg by indigo dots. (B and C) NP and AN viral loads did not differ by protocol-defined risk category (higher [in indigo] vs lower [in red]) for COVID-19 progression (B), but were significantly higher among viremic (in orange) vs aviremic (in blue) participants (C). (D) The proportion with SARS-CoV-2 viremia at study entry was the same for participants at higher (in indigo) vs lower (in red) risk for COVID-19 progression. (E) Total symptom scores reported at study entry in the participant diary were higher among viremic (in orange) participants. (F) Symptom scores did not correlate with NP or AN viral loads. Spearman correlation was used for correlation analyses (A, F). Two-sided Wilcoxon rank sum tests were used for all comparisons of continuous values (B, C, F) and chi-square test to compare proportions (D). In Figures B, C, and E, boxplots were used to demonstrate the distribution of viral loads or symptom score, where the horizontal line represents the median and the box limits represent the first and third quartiles (Q1 and Q3). The lower whisker is the minimum value within 1.5 times the interquartile range (IQR) under Q1, while the upper whisker is the maximum value within 1.5 times the interquartile range (IQR) over Q3. Source data for summary measures are provided as a Source Data file.

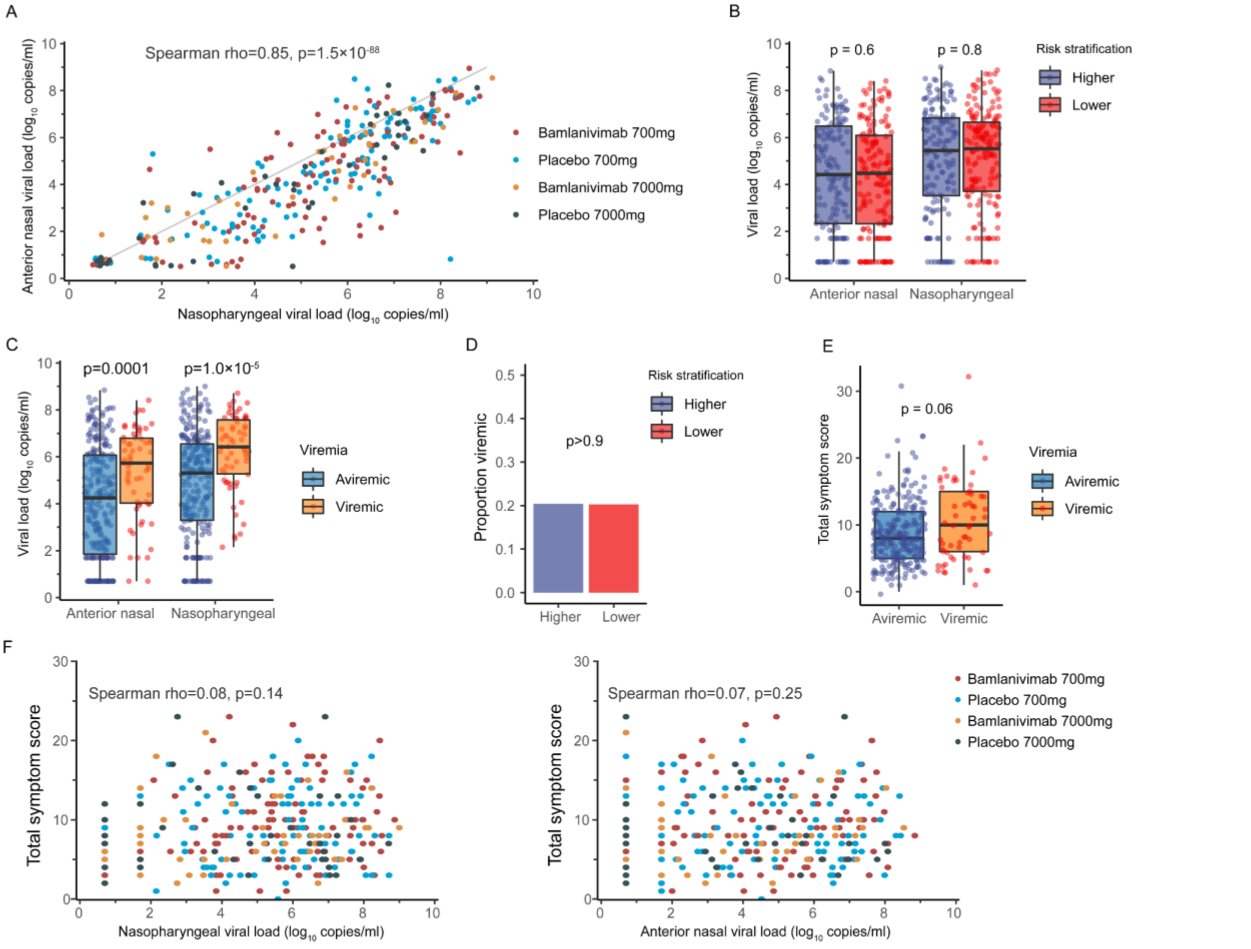

**Supplementary Fig. 6. Absolute values (left-hand panels) and fold-change from baseline (right-hand panels) in serum and plasma inflammatory and coagulation biomarker levels by treatment arm and visit for bamlanivimab 700 dose cohort.**
(A) C-reactive protein (CRP), (B) ferritin, (C) fibrinogen, (D) prothrombin time (PT), (E) lactate dehydrogenase, (F) activated partial thromboplastin time. Only absolute values are given for D-dimer (G) and prothrombin time international normalized ratio (INR) (H) as levels were largely normal/low and/or in a narrow range, and not suitable for fold-change analysis. Dots denote median values and bars interquartile range. * = $p<0.05$ for between-arm (bamlanivimab vs placebo) comparison by Wilcoxon test using log-transformed values. P-values are two-tailed and not adjusted for multiple comparisons. P-values below 0.05 were identified for the following evaluations: CRP at Day 7 ($p=0.002$), Day 14 ($p=0.046$), and Week 24 ($p=0.023$); ferritin at Day 7 ($p=0.014$) and Day 14 ($p=0.016$); fibrinogen at Day 7 ($p=0.024$) and Day 14 ($p=0.017$); and PT at Day 14 ($p=0.006$), Day 21 ($p=0.034$), and Week 12 ($p=0.002$). Source data for are provided as a Source Data file.

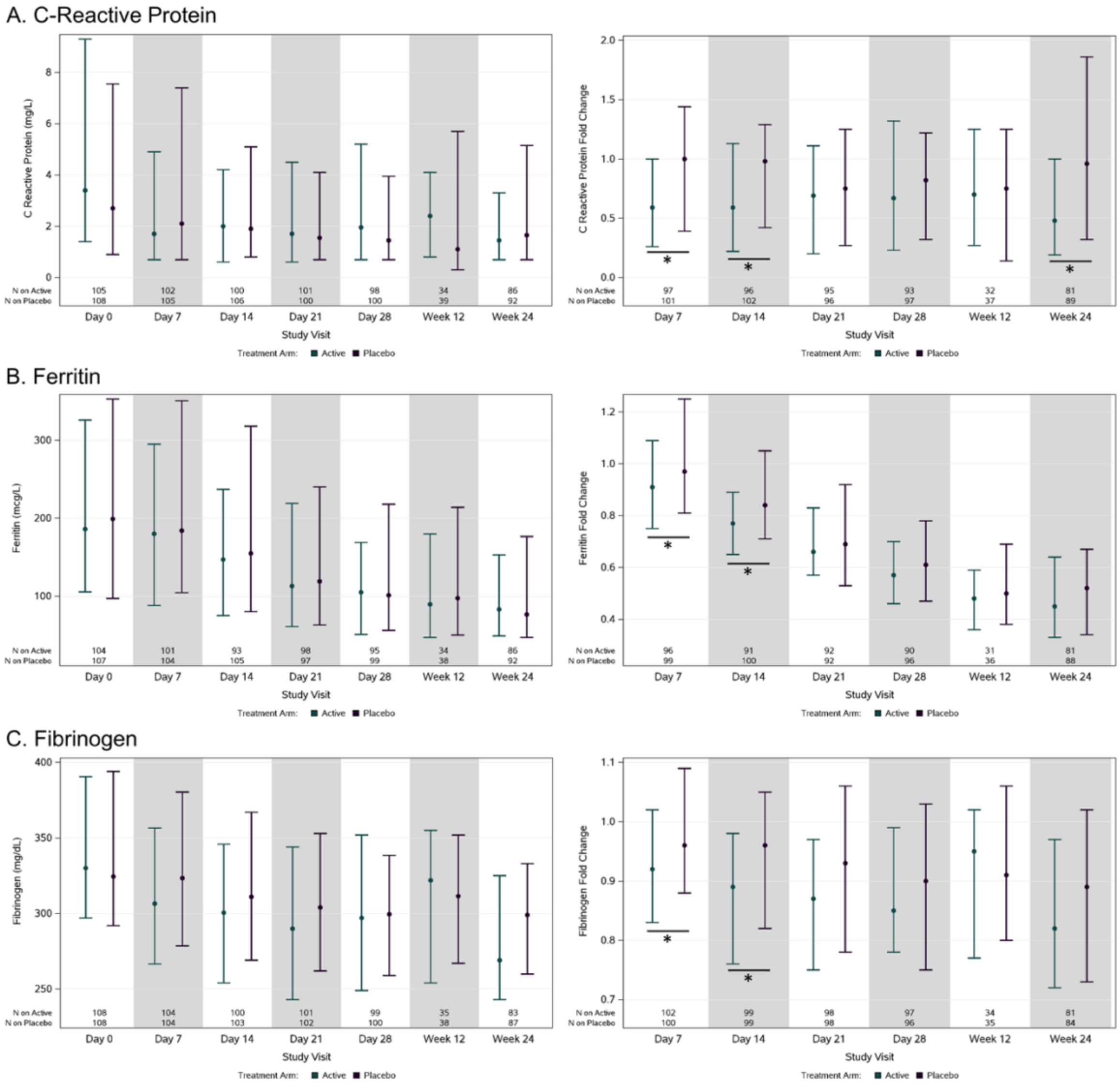

## D. Prothrombin Time

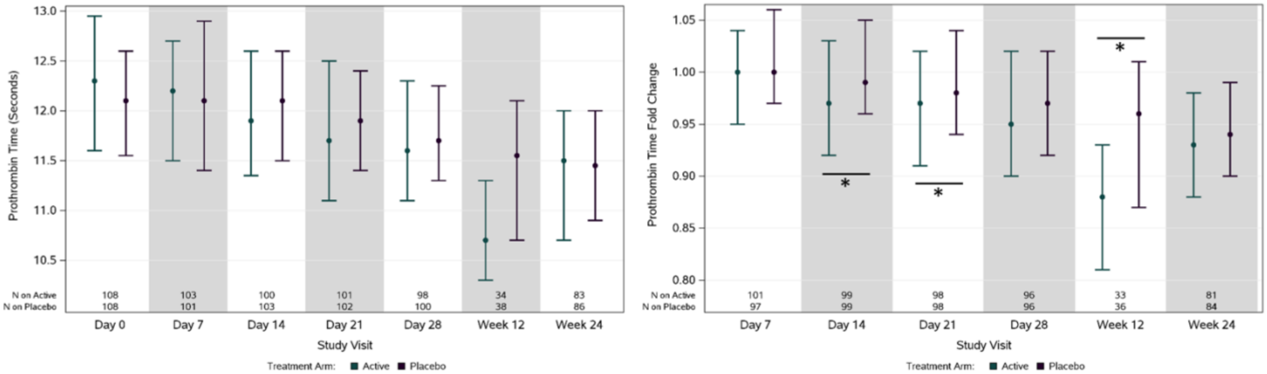


## E. Lactate Dehydrogenase

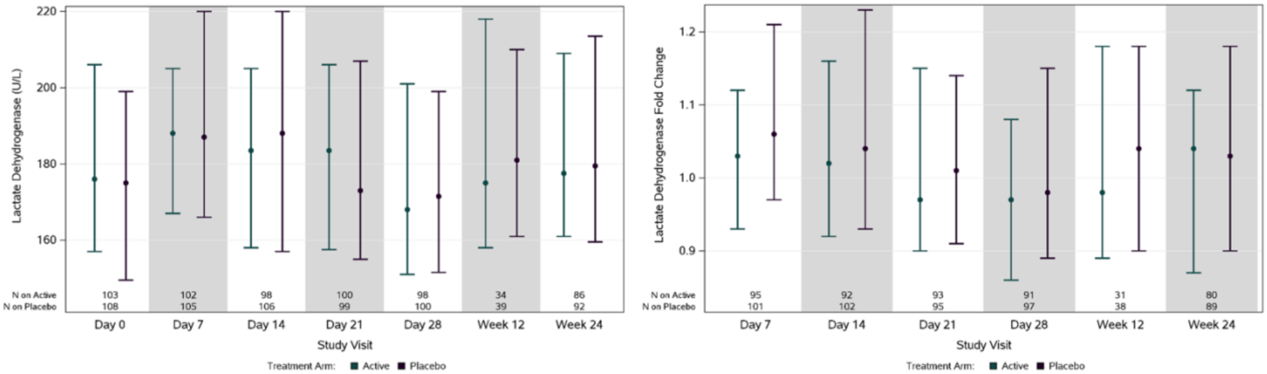


## F. Activated Partial Thromboplastin Time

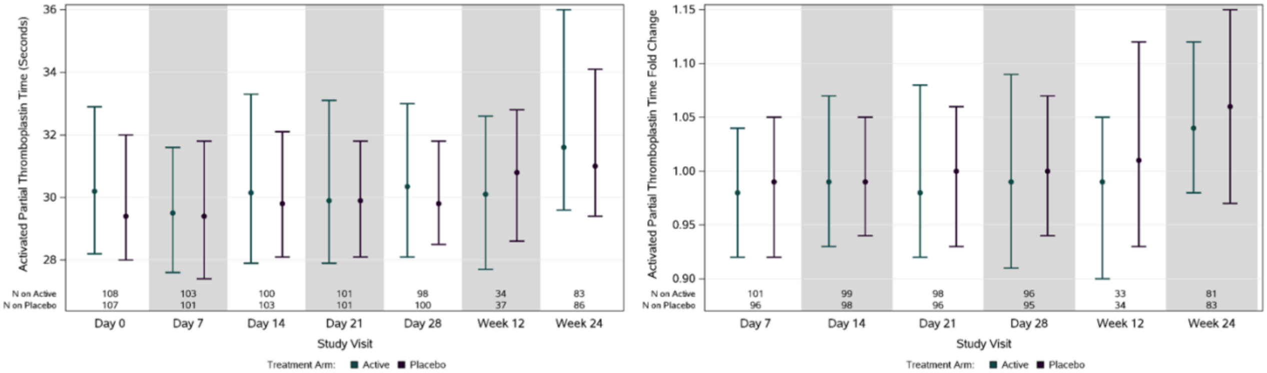


## G. D-dimer

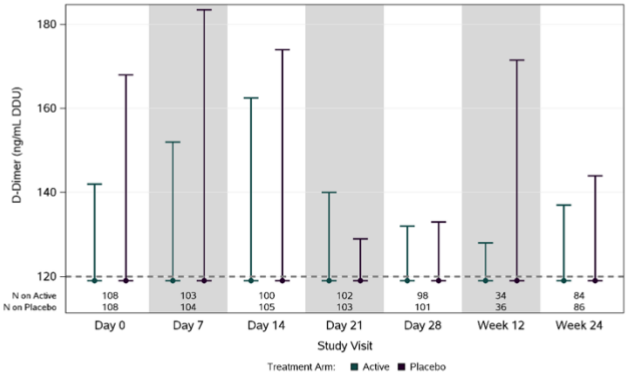


## H. Prothrombin Time International Normalized Ratio

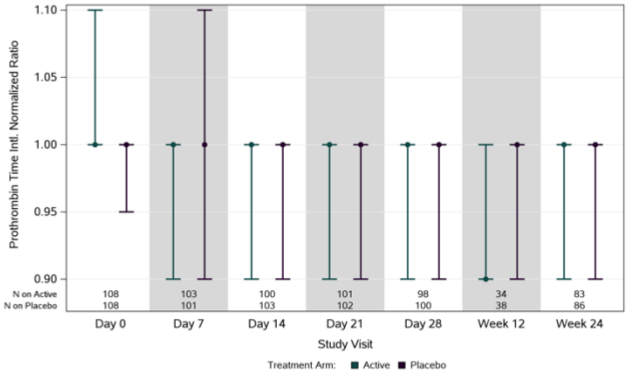

**Supplementary Fig. 7. Absolute values (left-hand panels) and fold-change from baseline (right-hand panels) in serum and plasma inflammatory and coagulation biomarker levels by treatment arm and visit for bamlanivimab 7000 dose cohort.**
(A) C-reactive protein, (B) ferritin, (C) fibrinogen, (D) prothrombin time, (E) lactate dehydrogenase, (F) activated partial thromboplastin time. Only absolute values are given for D-dimer (G) and prothrombin time international normalized ratio (INR) (H) as levels were largely normal/low and/or in a narrow range, and not suitable for fold-change analysis. Dots denote median values and bars interquartile range. * = p<0.05 for between-arm (bamlanivimab vs placebo) comparison by Wilcoxon test using log-transformed values. P-values are two-tailed and not adjusted for multiple comparisons. A p-value below 0.05 was identified for ferritin at Day 7 (p=0.034). Source data for are provided as a Source Data file.

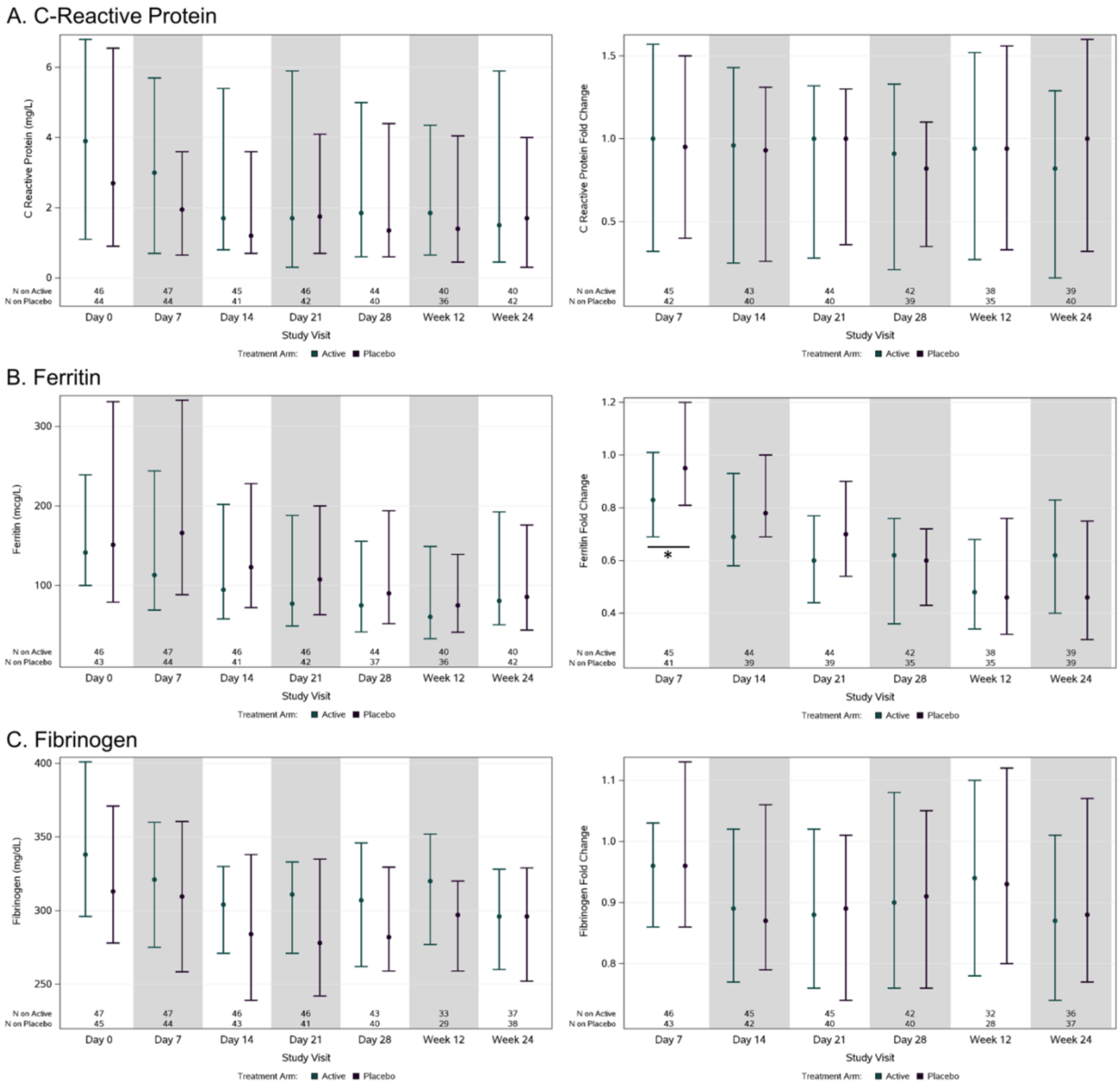

D. Prothrombin Time

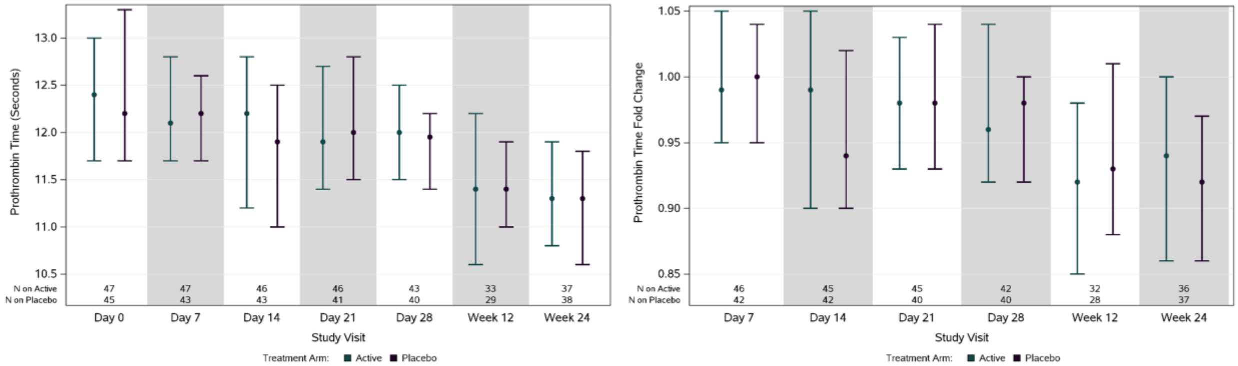


E. Lactate Dehydrogenase

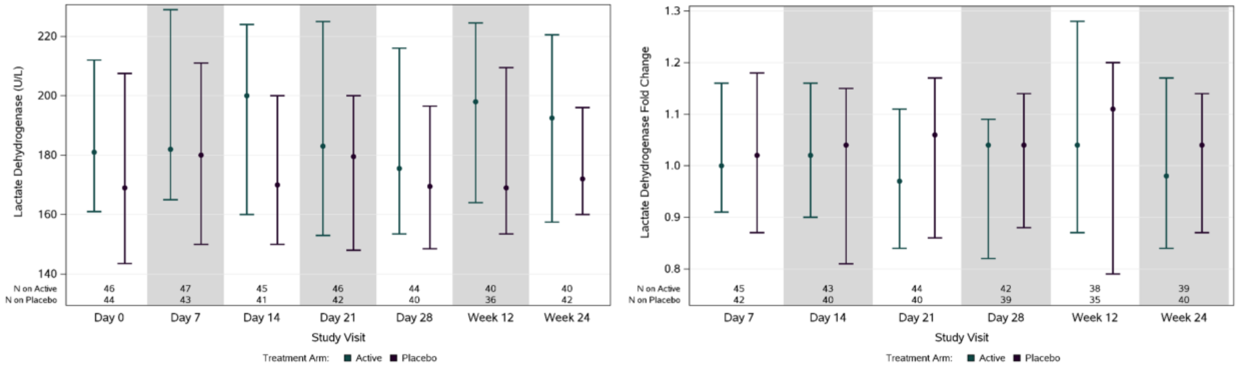


F. Activated Partial Thromboplastin Time

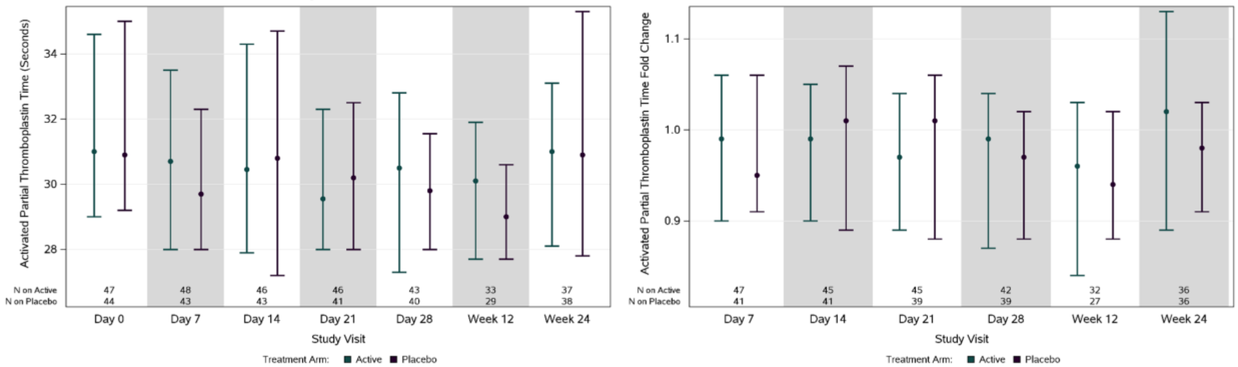


G. D-dimer

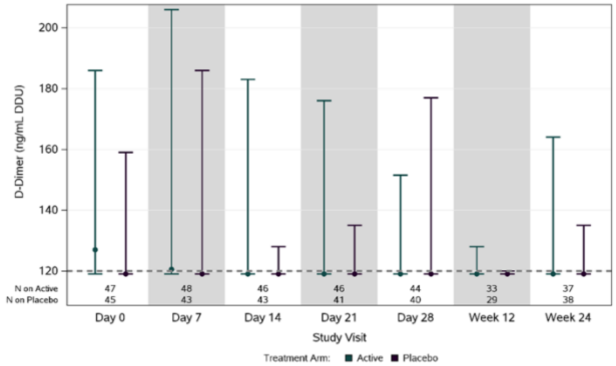


H. Prothrombin Time International Normalized Ratio

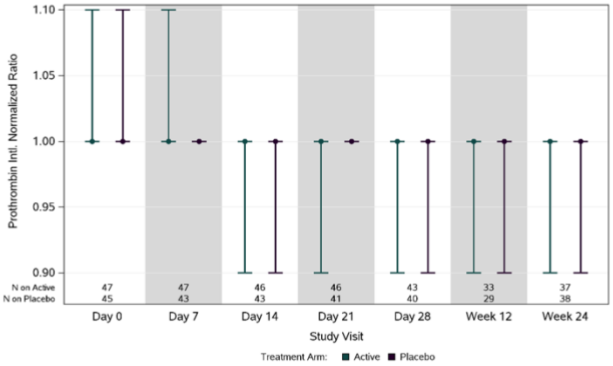

**Supplementary Methods**

Daily Symptom Diary

## ACTG A5401 PARTICIPANT STUDY DIARY CARD

NIAID AIDS CLINICAL TRIALS GROUP

Participant Number ☐☐☐☐ ☐☐☐☐

Today's Date ☐☐☐ ☐☐ ☐☐☐☐
MMM DD YYYY

Protocol Number | A | 5 | 4 | 0 | 1 |

Institution Code ☐☐☐☐☐☐

**SYMPTOMS**

1. Overall, how bad are your COVID-19 symptoms TODAY (check one)?
   ☐ No symptoms ☐ Mild ☐ Moderate ☐ Severe
   ☐ Very severe

2. Overall, how is your general physical health TODAY (check one)
   ☐ Poor ☐ Fair ☐ Good ☐ Very good ☐ Excellent

3. Have you returned to your usual (pre-COVID) health today (check one)?
   ☐ Yes ☐ No

# ACTG A5401 PARTICIPANT STUDY DIARY CARD

NIAID AIDS CLINICAL TRIALS GROUP

Participant Number

Today's Date MMM DD YYYY

Protocol Number A 5 4 0 1

Institution Code

Indicate the severity of your symptoms **DURING THE PAST 24 HOURS**

| **Symptoms** | **Absent** | **Mild** | **Moderate** | **Severe** |
|---|---|---|---|---|
| Cough | ☐ | ☐ | ☐ | ☐ |
| Shortness of breath or difficulty breathing at rest or with activity | ☐ | ☐ | ☐ | ☐ |
| Feeling feverish | ☐ | ☐ | ☐ | ☐ |
| Chills | ☐ | ☐ | ☐ | ☐ |
| Fatigue (low energy) | ☐ | ☐ | ☐ | ☐ |
| Body Pain or muscle pain or aches | ☐ | ☐ | ☐ | ☐ |
| Diarrhea | ☐ | ☐ | ☐ | ☐ |
| Nausea | ☐ | ☐ | ☐ | ☐ |
| Vomiting | ☐ | ☐ | ☐ | ☐ |
| Headache | ☐ | ☐ | ☐ | ☐ |
| Sore throat | ☐ | ☐ | ☐ | ☐ |
| Nasal obstruction or congestion (stuffy nose) | ☐ | ☐ | ☐ | ☐ |
| Nasal discharge (runny nose) | ☐ | ☐ | ☐ | ☐ |
| Other COVID related symptom, specify: | ☐ | ☐ | ☐ | ☐ |

I have a loss of taste: ☐ Yes ☐ No

I have a loss of smell: ☐ Yes ☐ No

## ACTG A5401 PARTICIPANT STUDY DIARY CARD

NIAID AIDS CLINICAL TRIALS GROUP

| | | | |
|---|---|---|---|
| Participant Number | | Today's Date | MMM DD YYYY |
| Protocol Number | A 5 4 0 1 | Institution Code | |

**TEMPERATURE**

**INSTRUCTIONS:** Please take your temperature every evening when you complete your study diary

Please record your temperature here:

______________ ☐ Fahrenheit (F) or ☐ Celsius (C) (check one)

Have you taken any fever reducing medication containing acetaminophen (paracetamol), ibuprofen, naproxen, aspirin, or similar product today? ☐ Yes ☐ No

If yes, please specify: ____________________________________________________________________

**MEDICAL CARE (skip this section if this is the Day 0 card)**

**Since the last time you completed this diary card**, have you:

1. Gone to seek urgent medical care at an emergency room or clinic (check one)? ☐ Yes ☐ No

   If yes, Reason to seek urgent medical care: ___________________________

2. Been hospitalized for at least 24 consecutive hours (check one)? ☐ Yes ☐ No

   If yes, Reason for hospitalization: ___________________________

*For this study, hospitalization is defined as at least 24 hours of acute care, including hospital admission, ER visits, and visits to temporary hospitals.*

Methods for modeling rate of decline of nasopharyngeal and anterior nasal swab SARS-CoV-2 RNA levels

The objective was to quantify the rate of decline of the virus after study entry, assuming an exponential decay in SARS-CoV-2 RNA levels (viral loads, VL) on nasopharyngeal (NP) and anterior nasal (AN) swabs. This assumption was consistent with visual inspection of the data. Separate models were fitted for NP and for AN swab viral loads. The model fitted was a simple biexponential (i.e., biphasic) decay given by

$$V = V_0(Ae^{-\lambda_1 t} + (1 - A)e^{-\lambda_2 t}),$$

where $V$ is the viral load (AN or NP), $V_0$ is the VL from which the decay begins, $A$ is the fraction of $V$ that decays in the first phase at rate $\lambda_1$, and (1-$A$) is the fraction that decays in the second phase at rate $\lambda_2$. We can test if a biphasic or a single-phase decay is better by setting $A$=1 in the expression above, which then causes $V$ to decay as a single exponential, and $A$ and $\lambda_2$ are not estimated.

*Participant selection for models*
Only participants with at least three viral load data points in the decay phase (including the peak value) were included. Additionally, participants identified as harboring resistance mutations either at baseline or emerging were excluded, because on visual inspection their decay dynamics were more complex than simple exponential decay. The effect of resistance is discussed in a separate study.[1]

*Model fitting*
For the fits, a non-linear mixed effects modeling approach was used, implemented in Monolix 2020 (Lixoft, Antony, France) in which the VL data from all the study participants was fitted simultaneously, on a $\log_{10}$ scale. In this modeling approach each estimated parameter is assumed to follow a given distribution in the population ($V_0$ is assumed lognormal, $A$ is assumed logit normal and $\lambda_{1,2}$ are assumed lognormal), and the parameter value for an individual $i$ can be expressed (if log-normal) as $\theta_i = \theta e^{\eta_i}$ where $\theta$ is the median value of the population distribution and $\eta_i$ is the individual random affect, assumed to be normally distributed as $N(0, \omega^2)$, accounting for variability between individuals. Data below the limit of quantification of the assay was handled as censored data, at the limit of detection or the lower limit of quantification.

*Model selection*
We tested multiple model structures including in each case (AN or NP, 700 mg or 7000 mg) whether a biexponential model or a single exponential model described the data better, and whether the decay rates ($\lambda_1$ or $\lambda_2$) were different in the two arms of the study (placebo vs. bamlanivimab) and the two doses of monoclonal antibody used (700 mg vs. 7000 mg). The selection of the best model was based on the Akaike Information Criteria (AIC), which was used to compare different model structures, and Wald tests for inclusion of covariates (i.e., study arm or dose), as provided by Monolix. P-values of these covariate tests are presented in Tables S8B and S8C.

**Supplementary Notes**

ACTIV-2/A5401 Study Team

Kara Chew, MD, MS, Co-Chair, David Geffen School of Medicine at University of California, Los Angeles, Los Angeles, CA, USA

David (Davey) Smith, MD, MAS, Co-Chair, University of California, San Diego, La Jolla, CA, USA

Eric Daar, MD, Vice Chair, Lundquist Institute at Harbor-UCLA Medical Center, Torrance, CA, USA

David Wohl, MD, Vice Chair, University of North Carolina at Chapel Hill School of Medicine, Chapel Hill NC, USA

Judith Currier, MD, MSc, Protocol Investigator and ACTG Chair, David Geffen School of Medicine at University of California, Los Angeles, Los Angeles, CA, USA

Joseph Eron, MD, Protocol Investigator and ACTG Vice Chair, University of North Carolina at Chapel Hill School of Medicine, Chapel Hill NC, USA

Arzhang Cyrus Javan, MD, MPH, DTM&H, NIH Division of AIDS (DAIDS) Clinical Representative, National Institutes of Health, Rockville, MD, USA

Michael Hughes, PhD, Lead Statistician, Harvard T.H. Chan School of Public Health, Boston, MA, USA

Carlee Moser, PhD, Statistician, Harvard T.H. Chan School of Public Health, Boston, MA, USA

Mark Giganti, PhD, Statistician, Harvard T.H. Chan School of Public Health, Boston, MA, USA

Justin Ritz, MS, Statistician, Harvard T.H. Chan School of Public Health, Boston, MA, USA

Lara Hosey, MA, Clinical Trials Specialist, AIDS Clinical Trials Group (ACTG) Network Coordinating Center, Social & Scientific Systems, a DLH Company, Silver Spring, MD, USA

Jhoanna Roa, MD, Clinical Trials Specialist, AIDS Clinical Trials Group (ACTG) Network Coordinating Center, Social & Scientific Systems, a DLH Company, Silver Spring, MD, USA

Nilam Patel, Clinical Trials Specialist, AIDS Clinical Trials Group (ACTG) Network Coordinating Center, Social & Scientific Systems, a DLH Company, Silver Spring, MD, USA

Kelly Colsh, PharmD, DAIDS Pharmacist, NIH/DAIDS Pharmaceutical Affairs Branch, Rockville, MD, USA

Irene Rwakazina, PharmD, DAIDS Pharmacist, NIH/DAIDS Pharmaceutical Affairs Branch, Rockville, MD, USA

Justine Beck, PharmD, DAIDS Pharmacist, NIH/DAIDS Pharmaceutical Affairs Branch, Rockville, MD, USA

Scott Sieg, PhD, Protocol Immunologist, Case Western Reserve University, Cleveland, OH, USA

Jonathan Li, MD, MMSc, Protocol Virologist, Brigham and Women’s Hospital, Harvard Medical School, Boston, MA, USA

Courtney Fletcher, PharmD, Protocol Pharmacologist, University of Nebraska Medical Center, Omaha, NE, USA

William Fischer MD, Protocol Critical Care Specialist, University of North Carolina at Chapel Hill School of Medicine, Chapel Hill NC, USA

Teresa Evering, MD, MS, Protocol Investigator, Weill Cornell Medicine, New York, NY, USA

Rachel Bender Ignacio, MD, MPH, Protocol Investigator, University of Washington, Seattle, WA, USA

Sandra Cardoso, MD, PhD, Protocol Investigator, Fundação Oswaldo Cruz, Rio de Janeiro, Brazil

Katya Corado, MD, Lundquist Institute at Harbor-UCLA Medical Center, Torrance, CA, USA

Prasanna Jagannathan, MD, Protocol Investigator, Stanford University School of Medicine, Palo Alto, CA, USA

Nikolaus Jilg, MD, PhD, Protocol Investigator, Massachusetts General Hospital, Harvard Medical School, Boston, MA, USA

Alan Perelson, PhD, Protocol Investigator, Los Alamos National Laboratory, Los Alamos, NM, USA

Sandy Pillay, MB, CHB, Protocol Investigator, Enhancing Care Foundation, Durban, KwaZulu-Natal, South Africa

Cynthia Riviere, MD, Protocol Investigator, GHESKIO Center, Port-au-Prince, Haiti

Upinder Singh, MD, Protocol Investigator, Stanford University School of Medicine, Palo Alto, CA, USA

Babafemi Taiwo, MBBS, MD, Protocol Investigator, Northwestern University Feinberg School of Medicine, Chicago, IL, USA

Joan Gottesman, BSN, RN, CCRP, Field Representative, Vanderbilt University Medical Center, Nashville, TN, USA

Matthew Newell, BSN, RN, CCRN, Field Representative, University of North Carolina at Chapel Hill School of Medicine, Chapel Hill NC, USA

Susan Pedersen, BSN, RN, Field Representative, University of North Carolina at Chapel Hill School of Medicine, Chapel Hill NC, USA

Joan Dragavon, MLM, Laboratory Technologist, University of Washington, Seattle, WA, USA

Cheryl Jennings, BS, Laboratory Technologist, Northwestern University, Chicago, IL, USA

Brian Greenfelder, BA, Laboratory Technologist, Ohio State University, Columbus, OH, USA

William Murtaugh, MPH, Laboratory Specialist, ACTG Laboratory Center, University of California, Los Angeles, Los Angeles, CA, USA

Jan Kosmyna, MIS, RN, CCPR, ACTG Community Scientific Subcommittee Representative, Case Western University Clinical Research Site, North Royalton, OH, USA

Morgan Gapara, MPH, International Site Specialist, ACTG Network Coordinating Center, Social & Scientific Systems, a DLH Company, Durham, NC, USA

Akbar Shahkolahi, PhD, International Site Specialist, ACTG Network Coordinating Center, Social & Scientific Systems, a DLH Company, Silver Spring, MD, USA

Paul Klekotka, MD, PhD, Industry Representative, Lilly Research Laboratories, San Diego, CA, USA

Participating Sites, Investigators, Study Personnel, and Additional Grant Support

AGA Clinical Trials, Hialeah, FL: Dario Altamirano (PI), Roberto Aguirre

Agile Clinical Research Trials, LLC, Atlanta, GA: Vasundhara Cheekati (PI), Purna Cheekati

AIDS Research and Treatment Center of the Treasure Coast, Vero Beach, FL: Gerald Pierone (PI), Juliana Elliott

Case Western Reserve University, Cleveland, OH: Jeffrey Jacobson (PI), Leila Hojat, Julie Pasternak; additional grant support: UM1AI069501

Cincinnati Clinical Research Site, Cincinnati, OH: Carl Fichtenbaum (PI), Jenifer Baer, Sarah Trentman; UM1AI069501

Cornell Clinical Trials Unit, New York Presbyterian Hospital-Weill Cornell Uptown, New York, NY: Teresa Evering (PI), Jonathan Berardi, Celine Arar; additional grant support: UM1AI069419, UL1TR002384

Duke University Medical Center, Durham, NC: Nwora Lance Okeke (PI), Folavan Morehead

Great Lakes Clinical Trials, Chicago, IL: Yevgeniy Bukhman (PI), Manish Jain, Eugene Bukhman

Harbor UCLA Clinical Research Site, Torrance, CA: Eric Daar (PI), Sadia Shaik, Timothy Hatlen; additional grant support: UM1A1069424, UL1TR000124

Inova Fairfax Medical Campus, Falls Church, VA: Christopher deFilippi (PI), Allen Freiler, Brian Moore

Jamaica Hospital Medical Center, Jamaica, NY: Robert Mendelson (PI), Tofura Ullah

Jasper Summit Research LLC, Jasper, AL: Jan Westerman (PI), David Davis, Donna Sherer

Johns Hopkins University, Baltimore, MD: Kelly Dooley (PI), Becky Becker, Adaliah Wilkins; additional grant support: additional grant support: UM1AI069465

Lakes Research, Miami Lakes, FL: Jose Pérez (PI), Eloy Roman, Heriberto Fernández

Massachusetts General Hospital, Boston, MA: Rajesh Gandhi (PI), Katrina Shea, and Matthew Planchon; additional grant support: UM1AI069412

Metro Infectious Disease Consultants, Burr Ridge, IL: Russell Petrak (PI), Nicholas Van Hise

Miami Clinical Research, Miami, FL: Keila Hoover (PI)

Northwestern University, Chicago, IL: Babafemi O Taiwo (PI), Claudia Hawkins, Baiba Berzins; additional grant support: UM1AI069471 and UL1TR001422

Ohio State University Medical Center, Columbus, OH: Carlos Malvestutto (PI), Heather Harber, Robyn Cicarella; additional grant support: UM1AI69494, UL1TR002733

PanAmerican Clinical Research LLC, Brownsville, TX: Christopher Romero (PI), Roberto Hernandez

Puerto Rico AIDS Clinical Trials Unit, San Juan, Puerto Rico: Jorge Santana-Bagur (PI), Sigrid Perez, Marielly Lopez; additional grant support: UM1AI69415, U54GM133807

Rush University Medical Center, Chicago, IL: Mariam Aziz (PI), Joan Swiatek

Sanford USD Medical Center, Sioux Falls, SD: Susan Hoover (PI), Melissa Carmon, Allison Lutz

Sealy Institute for Vaccine Sciences Clinical Trials Program Clinical Research Site, Galveston, TX: Laura Porterfield (PI), Gerianne Casey, Richard Rupp

Synergy Healthcare, Bradenton, FL: Patrick Weston (PI), Khalilah Weston

The Ponce de Leon Center Clinical Research Site, Atlanta, GA: Carlos del Rio (PI), Clifford Gunthel, Ericka Patric; additional grant support: UM1Al69418, P30AI050409

The University of Pittsburgh, Pittsburgh, PA: Madhu Choudhary (PI), Jennifer Sullivano; additional grant support: UM1AI069494

Thomas Street Health Center (formerly Lyndon B. Johnson Hospital), Houston, TX: Roberto Arduino (PI), Damian Chiandussi, Karen J. Vigil; additional grant support: UM1AI069432; UM1AI069503

University of Alabama at Birmingham, Birmingham, AL: Edgar T. Overton (PI), Sonya L. Heath, Sue Ellen Binkley; additional grant support: UM1AI069452

University of California Los Angeles, Los Angeles, CA: Kara Chew (PI), Aleen Khodabakhshian, Samantha Fortier; additional grant support: UM1AI69424, UL1TR001881

University of California San Diego Antiviral Research Center, San Diego, CA: Constance Benson (PI), Steven Hendrickx, Rosemarie Ramirez

University of California San Francisco, San Francisco, CA: Anne Luetkemeyer (PI), Suzanne Hendler, Dennis Dentoni-Lasofsky; additional grant support: UM1AI069496

University of Kansas Medical Center, Kansas City, KS: Mario Castro (PI), Leslie Spikes, Chase Hall

University of North Carolina at Chapel Hill, Chapel Hill, NC: David Wohl (PI), Jonathan Oakes, Amy James Loftis; additional grant support: UM1AI069423, UL1TR002489

University of Pennsylvania, Philadelphia, PA: Pablo Tebas (PI), William Short; additional grant support: UM1AI069534

University of Washington AIDS Clinical Trials, Seattle, WA: Rachel Bender Ignacio (PI), Sarah McGuffin, Chris Jonsson; additional grant support: UM1AI069841

Vanderbilt Therapeutics Clinical Research Site, Vanderbilt Health-One Hundred Oaks, Nashville, TN: David Haas (PI), Joan Gottesman, Beverly Woodward; additional grant support: UM1AI069439, UL1TR002243

Washington University School of Medicine, Saint Louis, MO: Rachel Presti (PI), Alem Haile; additional grant support: UM1AI069439

**Supplementary References**

1. Choudhary MC, Chew KW, Deo R, et al. Emergence of SARS-CoV-2 Resistance with Monoclonal Antibody Therapy. *medRxiv*. Sep 15 2021;doi:10.1101/2021.09.03.21263105

# ACTIV-2/A5401

# Adaptive Platform Treatment Trial for Outpatients with COVID-19 (Adapt Out COVID)

**A Multicenter Trial of the AIDS Clinical Trials Group (ACTG)**

**Sponsored by:**
**National Institute of Allergy and Infectious Diseases**

**Industry Support Provided by:**
**Lilly Research Laboratories, Eli Lilly and Company**

**IND # 151193**


| | |
|---|---|
| **Protocol Chair:** | **David (Davey) Smith, MD, MAS** |
| **Protocol Vice Chair(s):** | **Kara Chew, MD, MS**<br>**Eric Daar, MD**<br>**David Wohl, MD** |
| **DAIDS Clinical Representative:** | **Arzhang Cyrus Javan, MD, MPH, DTM&H** |
| **Clinical Trials Specialist:** | **Lara A. Hosey, MA, CCRP**<br>**Jhoanna C. Roa, MD** |


**FINAL Version 1.0**
**July 7, 2020**

ACTG AIDS CLINICAL TRIALS GROUP

ACTIV-2/A5401

Adaptive Platform Treatment Trial for Outpatients with COVID-19
(Adapt Out COVID)

SIGNATURE PAGE

I will conduct the study in accordance with the provisions of this protocol and all applicable protocol-related documents. I agree to conduct this study in compliance with United States (US) Health and Human Service regulations (45 CFR 46); applicable US Food and Drug Administration regulations; standards of the International Conference on Harmonization Guideline for Good Clinical Practice (E6); Institutional Review Board/Ethics Committee determinations; all applicable in-country, state, and local laws and regulations; and other applicable requirements (e.g., US National Institutes of Health, Division of AIDS) and institutional policies.

Principal Investigator: ________________________________________________
Print/Type

Signed: ____________________________________ Date: _____________
Name/Title

TABLE OF CONTENTS

Page

SIGNATURE PAGE ..... 2

SITES PARTICIPATING IN THE STUDY ..... 5

PROTOCOL TEAM ROSTER ..... 6

STUDY MANAGEMENT ..... 10

GLOSSARY OF PROTOCOL-SPECIFIC TERMS ..... 11

SCHEMA ..... 12

1.0 STUDY OBJECTIVES ..... 14
- 1.1 Co-Primary Objectives ..... 14
- 1.2 Secondary Objectives ..... 14
- 1.3 Exploratory Objectives ..... 15

2.0 INTRODUCTION ..... 16
- 2.1 Background ..... 16
- 2.2 Rationale ..... 18

3.0 STUDY DESIGN ..... 19

4.0 SELECTION AND ENROLLMENT OF PARTICIPANTS ..... 26
- 4.1 General Eligibility Criteria ..... 26
- 4.2 Study Enrollment Procedures ..... 28
- 4.3 Co-enrollment Guidelines ..... 29

5.0 INVESTIGATIONAL AGENT ..... 29
- 5.1 Regimen, Administration, and Duration ..... 29
- 5.2 Investigational Agent Formulation, Storage, and Preparation ..... 29
- 5.3 Supply, Distribution, and Accountability ..... 29
- 5.4 Concomitant Medications ..... 29

6.0 CLINICAL AND LABORATORY EVALUATIONS ..... 31
- 6.1 Schedule of Evaluations ..... 31
- 6.2 Timing of Evaluations ..... 36
- 6.3 Instructions for Evaluations ..... 37

7.0 ADVERSE EVENTS AND STUDY MONITORING ..... 45
- 7.1 Definitions of Adverse Events ..... 45
- 7.2 Eliciting and Documenting Adverse Events ..... 46
- 7.3 Recording Adverse Events ..... 47
- 7.4 Follow-up of Participants Reporting Adverse Events ..... 49
- 7.5 Study Monitoring ..... 49

8.0 CLINICAL MANAGEMENT ISSUES ..... 50
- 8.1 Toxicity ..... 50
- 8.2 Management of Side Effects ..... 50
- 8.3 Pregnancy ..... 51
- 8.4 Breastfeeding ..... 51

CONTENTS (Cont'd) Page

9.0 CRITERIA FOR DISCONTINUATION........51
9.1 Permanent and Premature Treatment Discontinuation........52
9.2 Premature Study Discontinuation........52

10.0 STATISTICAL CONSIDERATIONS........52
10.1 General Design Issues........52
10.2 Outcome Measures........53
10.3 Randomization and Stratification........56
10.4 Sample Size........57
10.5 Data and Safety Monitoring........61
10.6 Analyses........65
10.7 Unblinding........67

11.0 PHARMACOLOGY PLAN........68

12.0 DATA COLLECTION AND MONITORING........68
12.1 Data Quality Assurance........68
12.2 Records to Be Kept........68
12.3 Role of Data Management........69
12.4 Clinical Site Monitoring and Record Availability........69

13.0 PARTICIPANTS........69
13.1 Institutional Review Board (IRB) Review and Informed Consent........69
13.2 Ethical Conduct of Study........70
13.3 Participant Information and Consent........70
13.4 Participant Confidentiality........70
13.5 Study Discontinuation........71

14.0 PUBLICATION OF RESEARCH FINDINGS........71

15.0 BIOHAZARD CONTAINMENT........71

16.0 REFERENCES........72

APPENDIX I: ANALYTICAL FRAMEWORK FOR IMPLEMENTING THE GUIDELINES FOR GRADUATING AN INVESTIGATIONAL AGENT FROM PHASE II TO PHASE III........73

APPENDIX II: SAMPLE INFORMED CONSENT........76

ATTACHMENT A: CONSENT FOR USE OF EXTRA SAMPLES........91

APPENDIX III: INVESTIGATIONAL AGENT LY3819253........92

APPENDIX IV: SAMPLE INFORMED CONSENT FOR STUDY DRUG LY3819253........107

SITES PARTICIPATING IN THE STUDY

ACTIV-2/A5401 is a multicenter study open to select US- and non-US-based clinical research sites.

PROTOCOL TEAM ROSTER

Chair
David (Davey) Smith, MD, MAS
Antiviral Research Center
University of California, San Diego
220 Dickinson Street
San Diego, CA 92103
Phone: 619-543-7449
Fax: 619-298-0177
E-mail: d13smith@health.ucsd.edu

Vice Chairs
Kara Chew, MD, MS
Clinical AIDS Research and Education
University of California, Los Angeles
11075 Santa Monica Blvd, Suite 100
Los Angeles, CA 90025
Phone: 310-825-0796
Fax: 310-477-7657
E-mail: kchew@mednet.ucla.edu

Eric Daar, MD
Harbor University of California Los Angeles
Center CRS, CDCRC Building
1124 West Carson Street
Torrance, CA 90502
Phone: 424-201-3000
Fax: 310-533-0447
E-mail: edaar@lundquist.org

David Wohl, MD
Chapel Hill CRS,
Suite 2100, Bioinformatics Building
130 Mason Farm Road
Chapel Hill, NC 27514
Phone: 919-843-2723
Fax: 919-966-8928
E-mail: wohl@med.unc.edu

DAIDS Clinical Representative
Arzhang Javan, MD, MPH, DTM&H
TRP, DAIDS, NIAID, NIH
5601 Fishers Lane
Room 9E40, MSC 9830
Rockville, MD 20852
Phone: 301-761-7710
Mobile: 240-475-6683
E-mail: arzhang.javan@nih.gov

Clinical Trials Specialists
Lara Hosey, MA
ACTG Network Coordinating Center
Social & Scientific Systems, a DLH Company
8757 Georgia Avenue, 12th Floor
Silver Spring, MD 20910
Phone: 301-628-3395
Fax: 301-628-3302
E-mail: lara.hosey@dlhcorp.com

Jhoanna C. Roa, MD
ACTG Network Coordinating Center
Social & Scientific Systems, a DLH Company
8757 Georgia Avenue, 12th Floor
Silver Spring, MD 20910-3714
Phone: 301-628-3196
Fax: 301-628-3302
E-mail: jhoanna.roa@dlhcorp.com

Statisticians
Michael Hughes, PhD
Statistical and Data Analysis Center
Harvard School of Public Health
Building 2, Room 439A, 655 Huntington Ave
Boston, MA 02115-6017
Phone: 617-432-3161
Fax: 617-432-2832
E-mail: mhughes@sdac.harvard.edu

Statisticians (Cont'd)
Carlee Moser, PhD
Center for Biostatistics in AIDS Research
Harvard T.H. Chan School of Public Health
FXB Building, Room 513
Boston, MA 02115
Phone: 617-432-2526
Fax: 617-432-2829
E-mail: cmoser@sdac.harvard.edu

Justin Ritz, MS
Center for Biostatistics in AIDS Research
Harvard T.H. Chan School of Public Health
FXB Building, Room 510
Boston, MA 02115
Phone: 617-432-3034
Fax: 617-432-3163
E-mail: jritz@sdac.harvard.edu

DAIDS Pharmacists
Kelly Parsons, PharmD
Pharmaceutical Affairs Branch
DAIDS, OCSO, PAB
5601 Fishers Lane, 9E15
Rockville, MD 20852
Phone: 240-669-5721
E-mail: kelly.parsons@nih.gov

Irene Rwakazina, PharmD
Pharmaceutical Affairs Branch
DAIDS, OCSO, PAB
5601 Fishers Lane, 9E14
Rockville, MD 20852
Phone: 301-761-7269
E-mail: irene.rwakazina@nih.gov

Immunologist
Scott Sieg, PhD
Case Western Reserve University
Biomedical Research Building
Room 1020
2109 Adelbert Road
Cleveland, OH 44106-4984
Phone: 216-368-6594
Fax: 216-368-5415
E-mail: sfs2@case.edu

Virologist
Jonathan Li, MD, MMSc
Brigham and Women's Hospital
Therapeutics (BWHT) CRS
Harvard Medical School
Department of Infectious Diseases
65 Landsdowne Street, Room 421
Boston, MA 02139
Phone: 617-768-8476
Fax: 617-768-8738
E-mail: jli@bwh.harvard.edu

Pharmacologist
Courtney Fletcher, PharmD
University of Nebraska Medical Center
College of Pharmacy
986000 Nebraska Medical Center
Omaha, NE 68198-6000
Phone: 402-559-4333
Email: cfletcher@unmc.edu

Critical Care Specialist
William Fischer, MD
Division of Pulmonary and Critical Care Medicine
University of NC School of Medicine
Phone: 410-908-7779
Email: WFischer@med.unc.edu

Investigators
Judith Currier, MD, MSc
Clinical AIDS Research and Education (CARE) Center CRS
11075 Santa Monica Boulevard
Suite 100
Los Angeles, CA 90025
Phone: 310-825-9283
Fax: 310-477-7657
E-mail: jscurrier@mednet.ucla.edu

Investigators (Cont'd)
Joseph Eron, MD
University of North Carolina Global HIV
Prevention and Treatment CTU
Bioinformatics Building
130 Mason Farm Road, Suite 210
Chapel Hill, NC 27599-7215
Phone: 919-843-2722
Fax: 919-966-6714
E-mail: jeron@med.unc.edu

Field Representatives
Joan Gottesman, BSN, RN, CCRP
Vanderbilt Therapeutics (VT) CRS
One Hundred Oaks
719 Thompson Lane, Suite 47183
Nashville, TN 37204
Phone: 615-936-7143
Fax: 615-936-2644
E-mail: joan.gottesman@vumc.org

Susan Pedersen, RN, BSN
University of North Carolina Global HIV
Prevention and Treatment CTU
Bioinformatics Building, Suite 2100
130 Mason Farm Road
Chapel Hill, NC 27599-7215
Phone: 919-966-6713
Phone: 919-966-6712
Fax: 919-966-8928
E-mail: spederse@med.unc.edu

Laboratory Technologists
Joan Dragavon, MLM
University of Washington AIDS Clinical
Trials Unit, Retrovirology Laboratory
Ninth & Jefferson Building, Room 342.7
908 Jefferson Street
Seattle, WA 98104
Phone: 206-897-5210
E-mail: dragavon@uw.edu

Laboratory Technologists (Cont'd)
Amy James Loftis, BS
University of North Carolina Global HIV
Prevention and Treatment CTU
Bioinformatics Building
130 Mason Farm Road, Suite 2144
Chapel Hill, NC 27599-7215
Phone: 919-966-5462
E-mail: amy_james@med.unc.edu

Community Scientific Subcommittee (CSS)
Representative
Jan Kosmyna, MIS, RN, CCRP
Case CRS, 10041 Brookside Circle
North Royalton, OH 44133
E-mail: jankosmyna@gmail.com

International Site Specialists
Morgan Gapara, MPH
ACTG Network Coordinating Center
Social & Scientific Systems, a DLH Company
4505 Emperor Boulevard, Suite 400
Durham, NC 27703
Phone: 919-287-4503
Fax: 919-941-9349
E-mail: morgan.gapara@dlhcorp.com

Akbar Shahkolahi, PhD
ACTG Network Coordinating Center
Social & Scientific Systems, a DLH Company
8757 Georgia Avenue, 12th Floor
Silver Spring, MD 20910
Phone: 301-628-3318
Fax: 301-628-3302
E-mail: akbar.shahkolahi@dlhcorp.com

Industry Representative
Paul Klekotka, MD, PhD
Lilly Research Laboratories
Lilly Biotechnology Center
10290 Campus Point Drive
San Diego, CA 92121
Phone: 619-346-8191
E-mail: klekotka_paul@lilly.com

Laboratory Specialist
William A. Murtaugh, MPH
ACTG Laboratory Center
University of California Los Angeles
11075 Santa Monica Boulevard, Suite #200
Los Angeles, CA 90025
Phone: 310-794-9894
Email: wmurtaugh@milabcentral.org

STUDY MANAGEMENT

All general questions concerning this protocol and safety and risk management inquiries (including SAE reporting) should be sent to the following PPD 24/7 global coverage hotline:

| 24-Hour Study Protocol Queries and Pharmacovigilance Hotline | Telephone Number |
|---|---|
| North America | 1 888 483 7729 |
| Latin America | 55 11 4504 4801 |
| Europe, Middle East, and Africa (EMEA) and Asia Pacific (APAC) | 44 122 337 4240 |

Protocol E-mail Group

An A5401 email group will be maintained for dissemination of important protocol documents and communications.

Protocol-Specific Web Page

Additional information about management of the protocol can be found on the protocol-specific web page (PSWP).

GLOSSARY OF PROTOCOL-SPECIFIC TERMS

| | |
|---|---|
| ACTIV | Accelerating COVID-19 Therapeutic Interventions and Vaccines |
| AE | adverse event |
| AESI | adverse event of special interest |
| AUC | area under the curve |
| CDMS | Clinical Data Management System |
| CLIA | Clinical Laboratory Improvement Amendments |
| COVID-19 | coronavirus disease 2019 |
| CRS | clinical research site |
| DSMB | Data and Safety Monitoring Board |
| FDA | US Food and Drug Administration |
| ICU | intensive care unit |
| IRT | Interactive Response Technology |
| LPC | lab processing chart |
| mAb | monoclonal antibody |
| NP | nasopharyngeal |
| PBMC | peripheral blood mononuclear cells |
| SAE | serious adverse event |
| SAP | statistical analysis plan |
| SARS-CoV | Severe Acute Respiratory Syndrome coronavirus |
| SARS-CoV-2 | Severe Acute Respiratory Syndrome coronavirus 2 |
| SOE | Schedule of Evaluations |
| TOC | Trial Oversight Committee |

SCHEMA

ACTIV-2 / A5401

Adaptive Platform Treatment Trial for Outpatients with COVID-19
(Adapt Out COVID)

DESIGN

Adapt Out COVID is a master protocol to evaluate the safety and efficacy of investigational agents for the treatment of symptomatic non-hospitalized adults with COVID-19. It begins with a phase II evaluation, followed by a transition into a larger phase III evaluation for promising agents.

The trial is a randomized, blinded, controlled adaptive platform that allows agents to be added and dropped during the course of the study for efficient testing of new agents against placebo within the same trial infrastructure. When two or more new agents are being tested concurrently, the same placebo will be used, if feasible.

The primary outcome measures in the phase II evaluation will be duration of symptoms, similar to the outcome used for outpatient influenza studies, loss of detection of SARS-CoV-2 RNA by nasopharyngeal (NP) swab, and safety. Determination of whether a phase II agent will continue to be evaluated in phase III will be made after the last participant randomized to that agent or placebo group completes their day 28 phase II visit. If continued, data collected from participants enrolled in phase II will be included in the phase III evaluation.

The phase III evaluation is a continuation of the phase II trial for agents that meet study-defined criteria for further evaluation and for which sufficient investigational agent is available. An agent may also enter directly into phase III evaluation based on Trial Oversight Committee (TOC) assessments. The fully powered phase III trial will evaluate the efficacy of each selected investigational agent compared to placebo to prevent hospitalization and death in non-hospitalized adults with COVID-19.

The protocol will be amended when information becomes available from within or outside of the trial indicating that further randomization to a placebo is inappropriate.

DURATION

28 days of intensive follow-up, followed by limited follow-up through 24 weeks.

SAMPLE SIZE

Approximately 110 participants per investigational agent (and 110 on placebo) in the phase II evaluation and 1000 participants per investigational agent (and 1000 on placebo), including the 110 from phase II, in the phase III evaluation. Thus, there will be approximately equal numbers of participants receiving investigational agent and placebo in each phase. Participants who are randomized but do not start investigational agent or placebo will be replaced.

POPULATION

Outpatient adults (≥18 years) with a documented positive SARS-CoV-2 molecular test (antigen or nucleic acid) from a sample collected ≤7 days prior to study entry and with ≤10 days of symptoms of COVID-19 at study entry, plus the presence of select symptoms within 48 hours prior to study entry.

At least 50% of participants will meet the protocol definition of being at “high” risk of progression to severe COVID-19 as defined below.

STRATIFICATION

Randomization in both phase II and phase III will be stratified by 1) time from symptom onset (≤5 days versus >5 days) and 2) “high” versus “low” risk of progression to severe COVID-19, where “high” risk is defined by any of the following:

- persons aged 55 years and older
- persons having at least one of the following conditions:
    - chronic lung disease or moderate to severe asthma
    - obesity (body mass index [BMI] >35; may be based on self-report of height and weight)
    - hypertension
    - cardiovascular disease (including history of stroke)
    - diabetes
    - chronic kidney disease
    - chronic liver disease

REGIMEN

Investigational agents will be selected by the TOC for phase II evaluation based on the presence of in vitro data demonstrating activity against SARS CoV-2 entry or replication, phase I pharmacokinetics and safety data, and availability.

# 1.0 STUDY OBJECTIVES

## 1.1 Co-Primary Objectives

1.1.1 Phases II and III: To evaluate safety of the investigational agent.

1.1.2 Phase II: To determine efficacy of the investigational agent to reduce the duration of COVID-19 symptoms through study day 28.

1.1.3 Phase II: To determine the efficacy of the investigational agent to increase the proportion of participants with undetectable nasopharyngeal (NP) SARS-CoV-2 RNA at study days 3, 7, 14, 21, and 28.

1.1.4 Phase III: To determine if the investigational agent will prevent the composite endpoint of either hospitalization or death through study day 28. Hospitalization is defined as ≥24 hours of acute care, in a hospital or similar acute care facility, including Emergency Rooms or temporary facilities instituted to address medical needs of those with severe COVID-19 during the COVID-19 pandemic.

## 1.2 Secondary Objectives

1.2.1 Phases II and III: To determine whether the investigational agent reduces a COVID-19 Severity Ranking scale based on COVID-19-associated symptom burden (severity and duration), hospitalization, and death, through study day 28.

1.2.2 Phase II and III: To determine whether the investigational agent reduces the progression of COVID-19-associated symptoms.

1.2.3 Phases II and III: To determine if the investigational agent reduces SARS-CoV-2 detection or levels of RNA in nasal swabs.

1.2.4 Phase II: To determine the pharmacokinetics of the investigational agent.

1.2.5 Phase II: To evaluate differences in SARS-CoV-2 RNA levels in NP swabs between the investigational agent versus placebo treatment groups and among subgroups of the population and risk groups defined by age and comorbidities.

1.2.6 Phase II: To determine if the investigational agent reduces SARS-CoV-2 detection or levels of RNA in saliva and nasal swabs.

1.2.7 Phase II: To determine efficacy of the investigational agent to obtain pulse oximetry measurement of ≥96% through day 28.

1.2.8 Phase III: To evaluate differences in symptom duration between the investigational agent versus placebo treatment groups among subgroups of the population, and risk groups defined by age and comorbidities.

1.2.9 Phase III: To determine if the investigational agent will prevent the composite endpoint of either hospitalization or death through study week 24.

## 1.3 Exploratory Objectives

1.3.1 Phases II and III: To explore the impact of the investigational agent on participant-reported rates of SARS-CoV-2 positivity of household contacts.

1.3.2 Phases II and III: To explore if baseline and follow-up hematology, chemistry, coagulation, viral, and inflammatory biomarkers are associated with clinical and virologic outcomes in relation to investigational agent use.

1.3.3 Phases II and III: To explore possible predictors of outcomes across the study population, notably sex, time from symptom onset to start of investigational agent, race/ethnicity, and risk groups defined by age and comorbidities.

1.3.4 Phases II and III: To explore if the investigational agent changes the hospital course once a participant requires hospitalization.

1.3.5 Phases II and III: To explore and develop a model for the interrelationships between virologic outcomes, clinical symptoms, hospitalization, and death in each study group.

1.3.6 Phases II and III: To explore the relationship between exposure to the investigational agent and SARS-CoV-2 innate, humoral or cellular response, including anti-drug antibodies, as appropriate per investigational agent.

1.3.7 Phases II and III: To explore baseline and emergent viral resistance to the investigational agent.

1.3.8 Phases II and III: To explore the association between viral genotypes and phenotypes, and clinical outcomes and response to agents.

1.3.9 Phases II and III: To explore the association between host genetics and clinical outcomes and response to agents.

1.3.10 Phases II and III: To explore relationships between dose and concentration of investigational agent with virology, symptoms, and oxygenation.

1.3.11 Phases II and III: To explore the association between zinc and vitamin D levels and clinical outcomes and response to agents.

1.3.12 Phase II: To explore the impact of investigational agents on SARS-CoV-2 viremia, i.e., detection or level of SARS-CoV-2 RNA in the blood.

1.3.13 Phase II: To explore if self-collected nasal swabs and saliva correlate with the frequency of detection and levels of SARS-CoV-2 RNA in site-collected NP swabs.

# 2.0 INTRODUCTION

## 2.1 Background

Virology

Coronaviruses (CoVs) are positive-sense, single-stranded, enveloped RNA viruses, many of which are commonly found in humans and cause mild symptoms. Over the past two decades, emerging pathogenic CoVs capable of causing life-threatening disease in humans and animals have been identified, namely, severe acute respiratory syndrome coronavirus (SARS-CoV) in 2002-2003 and Middle East Respiratory Syndrome coronavirus (MERS-CoV) in 2012 [1].

New Threat

A novel pneumonia caused by a previously unknown betacoronavirus emerged in Wuhan, China, in December 2019. The virus is closely related to SARS-CoV-1, which caused an outbreak in 2003, and has been named SARS-CoV-2. The human disease caused by SARS-CoV-2 is called COVID-19.

During the current SARS-CoV-2 outbreak, the incidence of known cases has rapidly increased such that, on January 5, 2020, there were 59 confirmed cases, 278 cases on January 20, 2118 cases on January 26, and more than 80,000 cases and 2700 deaths as of February 25, 2020, according to various international health reporting agencies. As a result, on January 30, 2020, the International Health Regulations Emergency Committee of the World Health Organization (WHO) declared the COVID-19 outbreak a Public Health Emergency of International Concern. On January 31, 2020, the US Department of Health and Human Services declared a public health emergency in the United States. Despite quarantine measures, SARS-CoV-2 has spread to over 188 counties, infecting millions worldwide and killing hundreds of thousands [2]. Outbreak forecasting and modeling suggest that these numbers will continue to rise [3]. Global efforts to evaluate novel antivirals and therapeutic interventions to treat COVID-19 have intensified. There is currently no vaccine to prevent SARS-CoV-2 infection nor any therapeutic agent to treat COVID-19. Therefore, there is an urgent public health need for rapid development of novel interventions.

Disease Course

Once infection occurs, the clinical course is variable. Recent data suggest that fewer than 2.5% of infected persons will show symptoms within 2.2 days (CI, 1.8 to 2.9 days) of exposure, and symptom onset will occur within 11.5 days (CI, 8.2 to 15.6 days) for 97.5% of infected persons [4]. In most (~80%) cases, COVID-19 presents as a mild-to-moderately severe, self-limited acute respiratory illness with fever, cough, and shortness of breath. It remains unclear exactly what the rate of progression of COVID-19 is and what the predictors are for complications, including pneumonia, acute respiratory distress syndrome (ARDS), kidney failure, and death. It is clear that older age, male sex,

and comorbidities including diabetes and hypertension increase the risk for worse outcomes [5, 6]. In a recent meta-analysis, the main clinical symptoms were fever (88.5%), cough (68.6%), myalgia or fatigue (35.8%), expectoration (28.2%), and dyspnea (21.9%). Minor symptoms included headache or dizziness (12.1%), diarrhea (4.8%), and nausea and vomiting (3.9%) [7]. Laboratory examinations showed that lymphocytopenia (64.5%), increase of C-reactive protein (CRP) (44.3%), increase of lactate dehydrogenase (LDH) (28.3%), and leukocytopenia (29.4%) were more common in those with COVID-19 [5, 8].

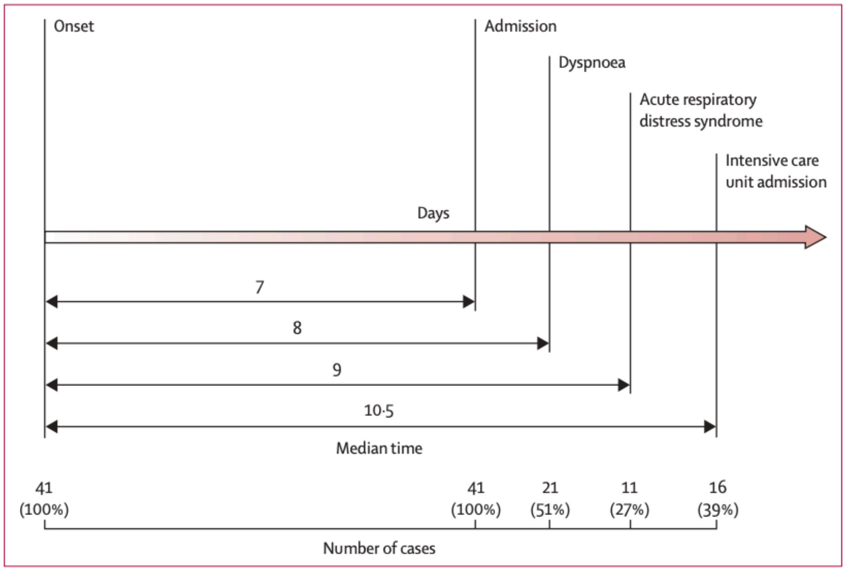


Figure 2.1-1: Timeline of COVID-19 Disease Progression. Onset refers to onset of symptoms.

Shedding

Viral infections jump from host to host through a variety of pathways. Coronaviruses do this through respiratory droplets. Understanding this shedding is important to understanding epidemic spread and how shedding relates to disease progression. Best evidence available now suggests that viral shedding, especially in upper respiratory secretions, is detectable around 2 days before symptoms develop and continues throughout the symptomatic phase. This shedding can be quite high during active disease and can continue for up to 37 days, with a quarter of persons still shedding at 3 weeks, as detected by NP swabs [7].

Biomedical Interventions

There is no clinically proven antiviral treatment for SARS-CoV-2 infection in the outpatient setting. The adenosine analog, remdesivir, has recently shown clinical benefit for COVID-19 in hospitalized patients, and was authorized by the FDA for emergency use for inpatients [4, 5]. Remdesivir must be given intravenously and has a short half-life, and thus is not optimal for an outpatient setting.

New agents are becoming available that may be useful for the treatment of non-hospitalized persons with COVID-19, including anti-SARS-CoV-2 monoclonal antibodies, viral enzyme inhibitors, small interfering RNAs, immune modulators, and other small molecules [9]. Before they can be clinically deployed, they will need to be evaluated quickly in ambulatory persons in a rigorous clinical trial, as will be achieved through ACTIV-2/A5401, the Adapt Out COVID Trial.

## 2.2 Rationale

There is an urgent need for a platform to rapidly evaluate therapies in the outpatient setting, to prevent disease progression, and reduce serious complications of COVID-19 and transmission [10]. ACTIV-2/A5401 is a phase II/III randomized, blinded, controlled adaptive platform trial to efficiently evaluate agents for the treatment of non-hospitalized persons with COVID-19. This will allow:

- comparison of multiple therapies with a common control group, when feasible, thus potentially requiring fewer participants than in independently conducted randomized controlled trials,
- continuous introduction of new promising agents as they become available,
- generation of separate effect size estimates for each therapy, and
- minimized downtime, with rapid movement of promising agents into phase III evaluation.

Additionally, the trial will facilitate the exploration of virologic endpoints as possible future primary endpoints in COVID-19 trials by assessing the correlation between changes in viral shedding and clinical outcomes.

Outcome Measures

Phase II evaluates the potential effect of an investigational agent on COVID-19-associated symptoms and on viral shedding. However, it is unknown a priori if an investigational agent that is effective in reducing symptom duration and/or viral shedding will have meaningful impact on the clinical outcome of hospitalization or death. Therefore, an investigational agent that has shown effects on clinical symptoms, hospitalization, death, oxygenation, and/or viral shedding and has an acceptable safety profile will be considered by the Trial Oversight Committee (TOC) for graduation to phase III evaluation (see section 3.0). The TOC is comprised of protocol, ACTG, and NIH Accelerating COVID-19 Therapeutic Interventions and Vaccines (ACTIV) group leadership.

For the primary symptom endpoint in phase II and as a secondary endpoint for phase III, the trial relies on targeted symptoms, which have been associated with COVID-19, and which are expected to be dynamic and improve with effective anti-SARS-CoV-2 therapy. The symptoms are described in section 6.3.10.

Investigational Agents

See appendices for Rationale for each investigational agent.

Multi-Site Design
In any multi-site study, outcomes can potentially differ due to variation in site populations, stage of epidemic spread, diagnostic capability, and clinical management. It is expected that any differences between sites will be balanced between arms through randomization.

## 3.0 STUDY DESIGN

Overview of Study Design
Adapt Out COVID is a master protocol to evaluate the safety and efficacy of investigational agents for the treatment of symptomatic non-hospitalized adults with COVID-19. It includes a phase II evaluation, with a transition into a larger phase III evaluation, with or without a pause in enrollment depending on the speed of enrollment and interim results from phase II. The trial is a randomized, blinded, controlled adaptive platform that allows investigational agents to be added and dropped during the course of the study for efficient testing of new agents against placebo within the same trial infrastructure [10].

The study is designed to allow both phase II and phase III evaluation of promising investigational agents in a single trial. For promising agents with limited product availability, however, a phase III evaluation may occur at a later time. Up to two dose levels of the same agent may be assessed. Agents may also enter directly into the phase III evaluation, if sufficient safety and efficacy data are available from outside the trial. The general approach for the phase II and phase III components are described in more detail below, followed by a brief discussion of some special design considerations for an adaptive platform trial when evaluating multiple agents concurrently. This protocol will be amended to include information about each new agent to be evaluated, as well as the handling of any design issues in the context of the platform design.

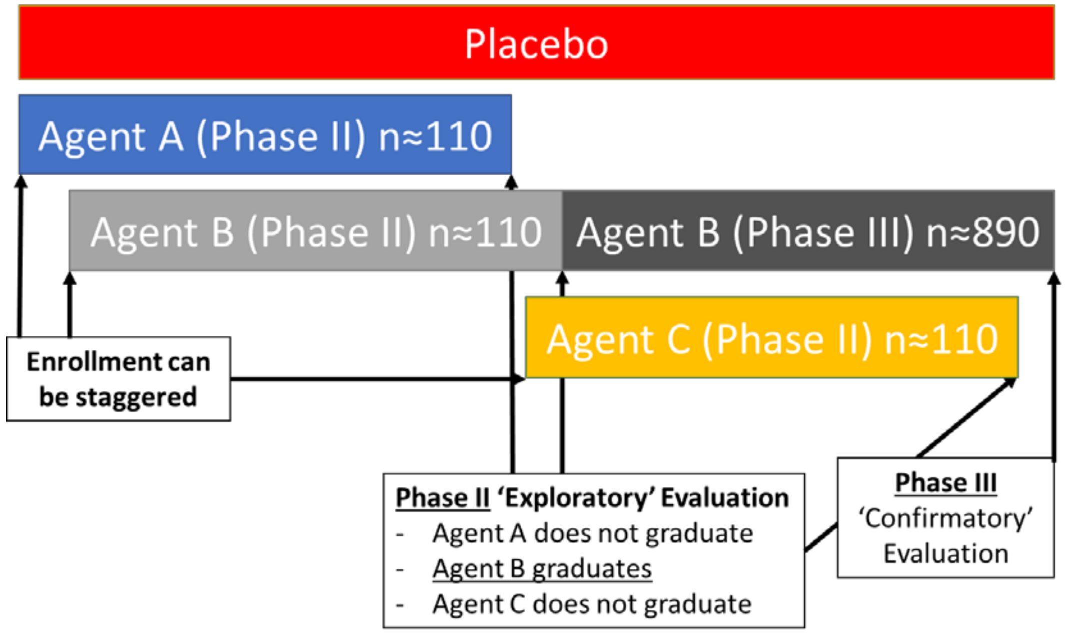


Figure 3.0-1: Adaptive platform trial that evaluates investigational agents to graduate (or not) to phase III. Comparison for a given investigational agent is with concurrently randomized participants receiving placebo. Further, evaluations from participants in phase II will be used for phase III objectives, if an agent continues to phase III evaluation.

Selection of Investigational Agents

The trial will rapidly assess various investigational agents that have shown substantial promise as anti-SARS-CoV-2 therapeutics in pre-clinical testing and for which there are suitable pharmacokinetics and safety data from phase I testing. The TOC will choose which agents are evaluated by the trial and when a standard-of-care agent will replace a placebo [11]. Based on TOC recommendations, an investigational agent can move directly into phase III testing without prior phase II evaluation in this trial. In this instance, the number enrolled in the phase III evaluation will be approximately 2000 persons, versus 1780 participants in phase III if a phase II evaluation had occurred in this Adapt Out COVID trial.

Phase II Period of Evaluation

In phase II, an investigational agent will be evaluated for safety, as well as for activity in reducing the duration of COVID-19 symptoms over 28 days, and frequency of loss of detection of SARS-CoV-2 RNA in NP swabs as compared to control.

Once 220 total participants have been assigned to a given agent or concurrent placebo for phase II evaluation, randomization to that agent will continue with participants going into phase III with its limited set of evaluations. The recommendation to continue further into the phase III evaluation will be made by the TOC. The collaborating company that is responsible for an agent that is recommended for graduation will decide whether to adopt the recommendation.

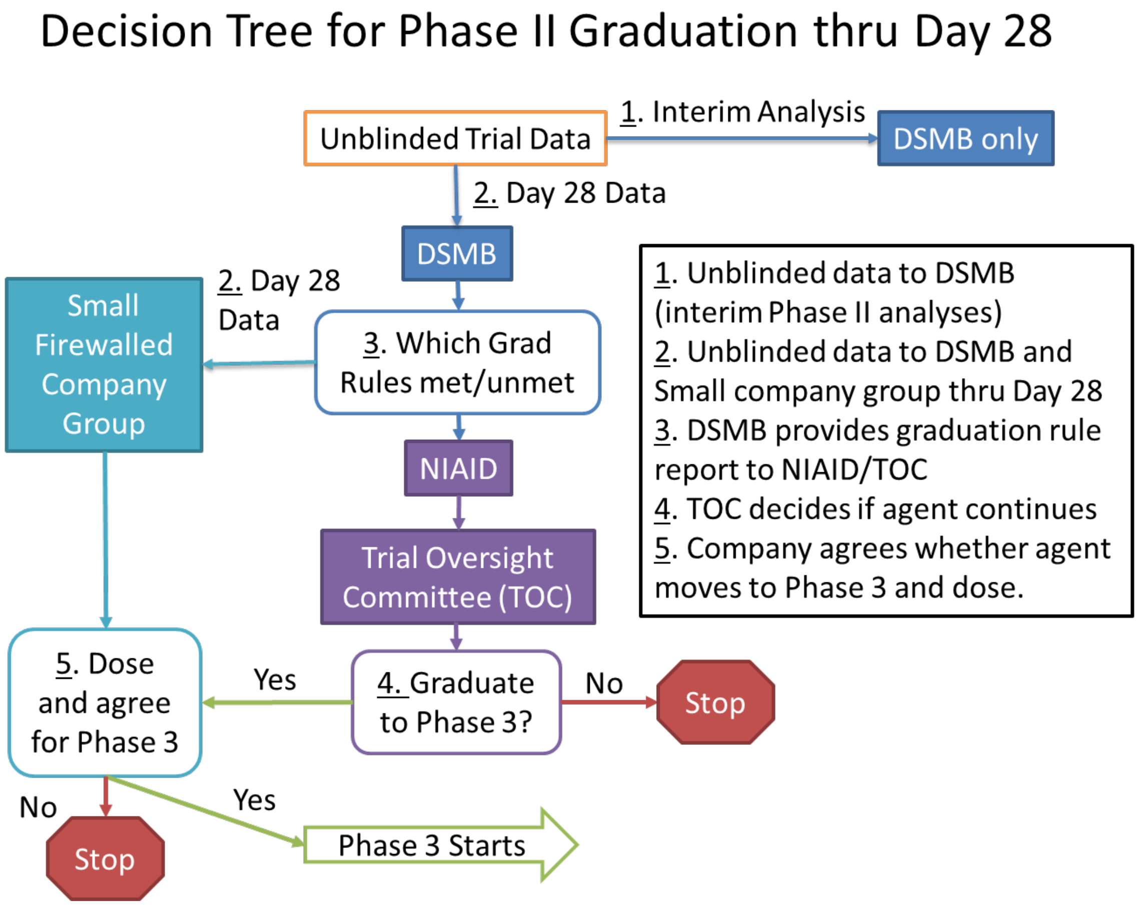


Figure 3.0-2: 1. Unblinded trial data will be provided to the Data and Safety Monitoring Board (DSMB) for interim analyses. 2. Unblinded trial data will also be provided to the DSMB after Day 28 data have been generated for it to assess Phase II graduation rules. Unblinded Day 28 data will also be provided to a small group of people from the company who owns the investigational agent. The small company group will not be allowed to share unblinded trial data outside of their group, per a clinical trial agreement. The rationale for sharing unblinded trial data to the small company group is to assist the company in deciding if their investigational agent should move into phase III evaluation or choosing a dose of their investigational agent to move into phase III. 3. The DSMB will report these graduation rules and safety to NIAID, as the trial sponsor, and then the NIAID will report these DSMB evaluations to the TOC. 4. The company will engage with the TOC about their agent's evaluation. 5. In conjunction with the company, the TOC will then decide whether an investigational agent enters into phase III.

Phase II Early Termination

During the phase II evaluation, there will be reviews of interim safety results by an independent Data and Safety Monitoring Board (DSMB). The DSMB may recommend early termination of randomization to a particular investigational agent if there are safety concerns.

Phase II to Phase III Graduation Rules

Graduation will be based on there being a desired level of evidence of an effect of an investigational agent versus placebo on one or more virologic and clinical outcome measures detailed below, as well as safety measures, as described below. The level of evidence required for the virology and clinical measures will be expressed in terms of Bayesian probability statements of the following form:

Probability (agent is better than placebo by at least X) is greater than 0.6 where X is defined below for each outcome measure. The choice of 0.6 for this probability indicates that there is a 3 to 2 odds of the agent being better than placebo for that parameter. As there is considerable uncertainty about the association between phase II outcomes and the phase III outcome of hospitalization or death, graduation will be considered if this probability statement is met for any one of the virology and symptom outcome measures listed below (i.e., it does not need to be met for all outcome measures listed).

*Virology:* The virology-based graduation guideline for an investigational agent to be eligible for phase III evaluation will be evidence of any one of the following:

1) Higher absolute proportion of participants testing negative for SARS-CoV-2 in NP swabs by at least 20% at one or more of the scheduled in-person measurement times (e.g., 30% for placebo and 50% for investigational agent at day 7) as compared to placebo (i.e., X in the probability statement above is an absolute 20% increase for this outcome); or
2) A decrease in median SARS-CoV-2 RNA levels in NP swabs of at least 1 $log_{10}$ copies/mL at one or more of the scheduled in-person measurement times as compared to placebo (i.e., X in the probability statement above is 1 $log_{10}$ copies/mL); or
3) A relative reduction in median area under the curve measure (AUC) of SARS-CoV-2 RNA levels in NP swab viral loads through study day 28 of at least 20%, as compared to placebo (i.e., X in the probability statement above is a relative 20% reduction)

The absolute difference of 20% in 1) and the 1 $log_{10}$ copies/mL difference in 2) were surpassed in a comparison of interferon beta-1b, ribavirin, and lopinavir-ritonavir to lopinavir-ritonavir alone in a trial among hospitalized COVID-19 patients [8]. The threshold used in 3) also seems achievable based on the same trial though the AUC outcome was not formally evaluated in that trial.

*Symptoms:* The symptom-based graduation guideline for an investigational agent to be eligible for phase III evaluation will be a relative reduction of at least 20% in median duration of symptoms as compared to placebo (i.e., X in the probability statement above is a relative 20% reduction). Based on two very small studies (n=12 and n=16) that have presented results about variability in symptom duration in populations akin to the one in this study, it is anticipated that this study will be well powered to detect a 20% relative reduction in median symptom duration (e.g., from a median of 10 days to 8 days) [6, 7].

*Oxygen Saturation:* The oxygen saturation-based graduation guideline for an investigational agent to be eligible for phase III evaluation will be a higher absolute proportion of participants with pulse oximetry of ≥96% by at least 20% at one or more of the scheduled measurement times as compared to placebo (i.e., X in the probability statement above is an absolute 20% increase for this outcome). It is uncertain what might be the percentage of persons with pulse oximetry of ≥96% at each of these times in the population being studied, and is likely to depend on the time since onset of symptoms at which participants are enrolled. However, a 20% absolute difference in percentage of participants with pulse oximetry of ≥96% is thought to be relevant.

*Safety:* Graduation to phase III will also depend on an acceptable safety profile, as determined by the DSMB. This decision will largely be based on differences in the frequency of Grade 3 and 4 AEs between participants receiving the investigational agent and those receiving placebo.

*Hospitalization/Death*: Although there will be very limited precision to compare an investigational agent to placebo in Phase II, graduation may also be considered based on hospitalization/death if the proportion of participants who are hospitalized or die by day 28 is lower by 33.3% (specifically, one-third) for an investigational agent versus placebo (i.e., X in the probability statement above is a relative reduction of 33.3% for this outcome).

*Other:* The TOC may also consider other secondary outcomes (such as the dynamics of virologic measures and symptoms over time, or any evidence of viral rebound to suggest resistance) in the decision to graduate an investigational agent from phase II to phase III evaluation, as provided by the DSMB. In addition, based on TOC recommendations from review of existing data from outside of the study, an investigational agent may move directly into phase III evaluation without completing phase II evaluation through this trial.

Since this study has a transition between phase II and phase III evaluations, participants will be randomized into the phase III portion of the trial when the phase II portion has been fully enrolled, provided that an interim analysis when 50% of participants have day 14 evaluations shows that at least one of the graduation criteria has been met at that point; otherwise, enrollment will be paused. The final decision to graduate an investigational agent to phase III will be determined when day 28 evaluations have been completed for all phase II participants. This means that some participants may be enrolled into phase III evaluations before all evaluations have been completed in all participants in phase II, and thus, participants may be enrolled in phase III before a decision has been made by the TOC that an agent should graduate to phase III. For participants that are enrolled in phase III for an agent that does not graduate, they will be followed through week 24 per the phase III SOE (Table 6.1-2) for safety and other monitoring.

Phase III Period of Evaluation

If it is decided that an agent graduates to phase III evaluation, then the study will continue for that agent using a continuation of the randomized design to evaluate efficacy of the investigational agent to reduce the composite primary outcome of

hospitalization or death over 28 days (i.e., from study day 0 through day 28) with additional follow-up to week 24 for clinical and immunologic parameters. To increase efficiency of the design, data collected during the phase II evaluation will contribute to the phase III evaluation. Throughout phase II and phase III, participants who do not start their randomized investigational agent or placebo will be replaced with new participants who are re-randomized.

Phase III Early Termination

During the phase III evaluation, there will be reviews of both interim safety and efficacy results by an independent DSMB. The DSMB may recommend early termination of randomization to a particular investigational agent if there are safety concerns, if efficacy of the agent versus placebo has been established, or if it is unlikely that efficacy of the agent versus placebo would be established by continuing to planned maximal sample size. As a guideline for early termination of the comparison of an agent to placebo based on efficacy using concurrently randomized participants, an O'Brien and Fleming type stopping guideline will be used. Early termination for statistical and operational futility will also be considered.

Design Considerations Concerning the Placebo Group and Study Population

The inclusion of a placebo group, rather than an untreated open-label control group, is considered important for the integrity of the study to reduce the possibility of differential retention of participants randomized to an investigational agent versus to the control group, as well as to minimize subjective bias in completion of symptom diaries by participants. However, having exactly the same placebo for multiple investigational agents may not be achievable. To allow for the possibility of separate placebos for each agent, as an example with three agents (A, B, and C) being evaluated, participants will first be randomized to three agent groups (Group A, B and C) and then, within each Group, be immediately randomized to the active agent or the corresponding placebo in a 3:1 ratio (e.g., 3:1 to Agent A or placebo within Group A). Evaluation of Agent A would then be the randomized comparison of participants assigned to Agent A versus the comparable number of participants concurrently assigned to any of the placebos (i.e., the placebo for Agent A, the placebo for Agent B and the placebo for Agent C). Placebo for Agents A, B and C may be the same placebo or different placebos if the placebo cannot be the same due to differences such as route of administration (IV versus oral).

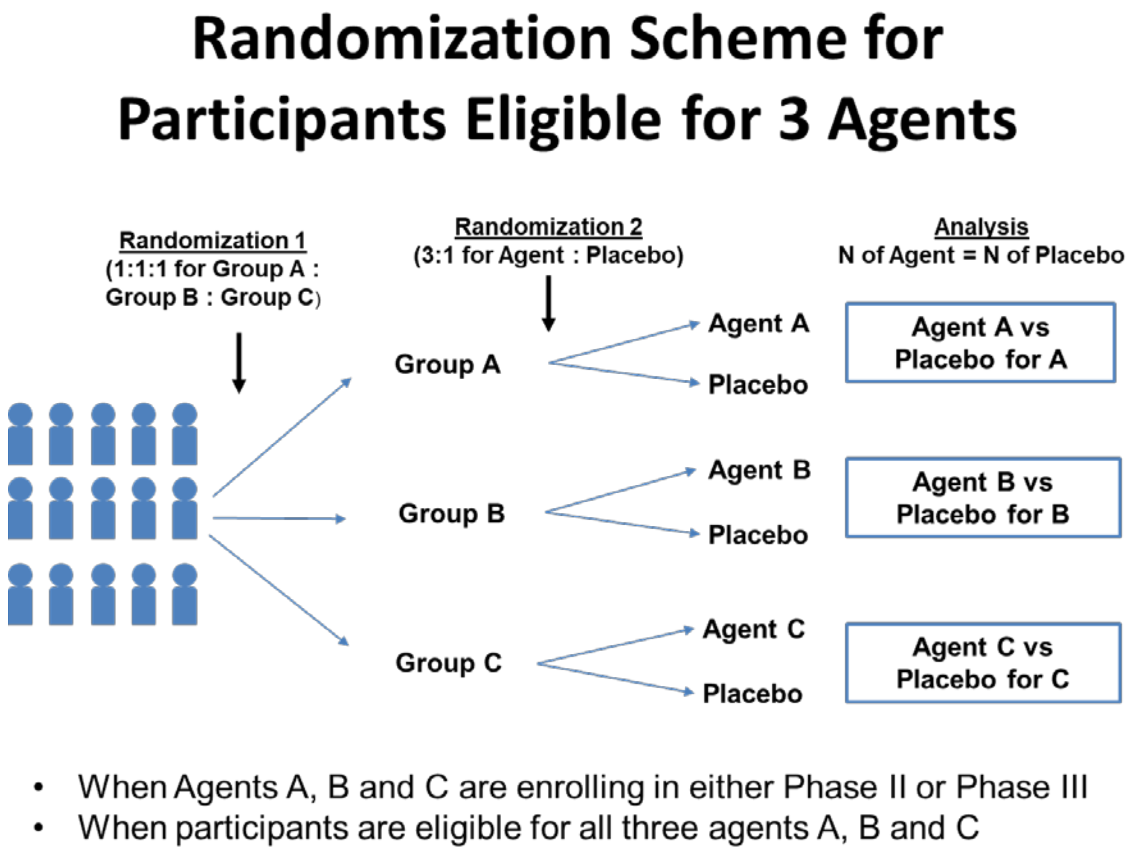


Figure 3.0-3: For either phase II or III, participants will undergo two randomizations. The first randomization will be to each Group equally. The second randomization will ensure that the number of participants receiving each active agent is approximately equal to the number assigned any of the placebos (i.e., combining the placebos into a single control group).

The platform design also needs to be flexible with regard to potential differences in study population eligible for randomization to different agents, for example due to safety or polypharmacy issues. As an example, if some participants are eligible to receive Agent A but not Agent B, then the randomization can be structured to allow randomization of these participants to Agent A or placebo only. In this case, these participants would not be considered as part of the placebo group for evaluating Agent B since their inclusion in this comparison could introduce bias.

<u>Isolation Procedures</u>

Given that SARS-CoV-2 is spread through respiratory secretions, each site must develop procedures to protect study staff and participants in other trials from infectious exposure. Each site will have a plan for appropriate protection by providing PPE, setting up isolation rooms, and providing special access points or contact with study participants, including the possibility for home or other non-clinic in-person visits. Each

site will develop their own set of procedures for such participant contact. Guidance for the sites can be found in the Manual of Procedures (MOP).

# 4.0 SELECTION AND ENROLLMENT OF PARTICIPANTS

## 4.1 General Eligibility Criteria

### 4.1.1 Inclusion Criteria

4.1.1.1 Ability and willingness of participant (or legally authorized representative) to provide informed consent prior to initiation of any study procedures.

4.1.1.2 Individuals ≥18 years of age.

4.1.1.3 Documentation of laboratory-confirmed SARS-CoV-2 infection, as determined by a molecular test (antigen or nucleic acid) from any respiratory tract specimen (e.g., oropharyngeal, NP, or nasal swab, or saliva) collected ≤168 hours prior to study entry and conducted at any US clinic or laboratory that has a Clinical Laboratory Improvement Amendments (CLIA) certification or its equivalent or any non-US DAIDS-approved laboratory.

4.1.1.4 Participants must be expected to begin study treatment no more than 10 days from self-reported onset of COVID-19 related symptoms or measured fever, defined as the self-reported date of first reported sign/symptom from the following list:

- subjective fever or feeling feverish
- cough
- shortness of breath or difficulty breathing at rest or with activity
- sore throat
- body pain or muscle pain/aches
- fatigue
- headache
- chills
- nasal obstruction or congestion
- nasal discharge
- loss of taste or smell
- nausea or vomiting
- diarrhea
- documented temperature >37.8°C

4.1.1.5 One or more of the following signs/symptoms present within 48 hours prior to study entry:

- subjective fever or feeling feverish
- cough

- shortness of breath or difficulty breathing at rest or with activity
- sore throat
- body pain or muscle pain/aches
- fatigue
- headache
- chills
- nasal obstruction or congestion
- nasal discharge
- nausea or vomiting
- diarrhea
- documented temperature >37.8°C

4.1.1.6 Oxygenation saturation of ≥92% obtained at rest by study staff within 48 hours prior to study entry, unless the potential participant regularly receives chronic supplementary oxygen for an underlying lung condition.

4.1.1.7 Agrees to not participate in another clinical trial for the treatment of COVID-19 or SARS-CoV-2 during the study period until reaching hospitalization or 28 days post-entry, whichever is earliest.

4.1.1.8 Additional inclusion criteria as appropriate for the investigational agent (see relevant appendix/appendices).

### 4.1.2 Exclusion Criteria

4.1.2.1 History of or current hospitalization for COVID-19.

4.1.2.2 Current need for hospitalization or immediate medical attention in the clinical opinion of the site investigator.

4.1.2.3 Use of any prohibited medication listed in section 5.4.1 within 30 days prior to study entry.

4.1.2.4 Receipt of convalescent COVID-19 plasma treatment at any time prior to study entry.

4.1.2.5 Receipt of a SARS-CoV-2 vaccine at any time prior to study entry.

4.1.2.6 Receipt of other available investigational treatments for SARS-CoV-2 at any time prior to study entry. This does not include drugs approved for other uses and taken for those uses.

4.1.2.7 Receipt of systemic steroids (e.g., prednisone, dexamethasone) or inhaled steroids within 30 days prior to study entry unless a stable dose used for a chronic condition.

4.1.2.8 Known allergy/sensitivity or any hypersensitivity to components of the investigational agent or placebo. See relevant appendix.

4.1.2.9 Any co-morbidity requiring surgery within 7 days prior to study entry, or that is considered life threatening in the opinion of the site investigator within 30 days prior to study entry.

4.1.2.10 Additional exclusion criteria as appropriate for the investigational agent (see relevant appendix/appendices).

## 4.2 Study Enrollment Procedures

All sites will be registered through the DAIDS Protocol Registration Office by PPD.

Prior to implementation of this protocol, and any subsequent full version amendments, each site must have the protocol and the protocol consent form(s) approved, as appropriate, by the institutional review board (IRB)/ethics committee (EC) and any other applicable regulatory entity (RE) responsible for oversight of the study. Upon receiving final approval, PPD on the site’s behalf will submit all required protocol registration documents to the DAIDS Protocol Registration Office (DAIDS PRO) at the Regulatory Support Center (RSC). The DAIDS PRO will review the submitted protocol registration packet to ensure that all of the required documents have been received.

Site-specific informed consent forms (ICFs) will be reviewed and approved by the DAIDS PRO, and sites and PPD will receive an Initial Registration Notification from the DAIDS PRO. A copy of the Initial Registration Notification should be retained in the site's regulatory files.

Upon receiving final IRB/EC and any other applicable RE approvals for an amendment, sites should implement the amendment immediately. Sites are required to submit an amendment registration packet to the DAIDS PRO at the RSC. The DAIDS PRO will review the submitted protocol registration packet to ensure that all required documents have been received. Site-specific ICF(s) will be reviewed by the DAIDS PRO if the Site ICF was not submitted as part of the initial registration. Sites and PPD will receive an Amendment Registration Notification when the DAIDS PRO receives a complete registration packet. A copy of the Amendment Registration Notification should be retained in the site's regulatory files.

For additional information on the protocol registration process and specific documents required for initial and amendment registrations, refer to the current version of the DAIDS Protocol Registration Manual.

### 4.2.1 Protocol Activation

PPD will be responsible for site activation for both ACTG and non-ACTG sites.

#### 4.2.2 Randomization

Participants who meet the enrollment criteria will be randomized to the study through the IRT (Interactive Response Technology) system.

### 4.3 Co-enrollment Guidelines

Co-enrollment in an observational study is allowed and does not require permission from the A5401 protocol chairs, as long as ACTG network blood collection limits are not exceeded, i.e., 450mL over 8 weeks.

For specific questions and approval for co-enrollment in other studies, sites should follow the directions described in the MOPS and Study Management section.

## 5.0 INVESTIGATIONAL AGENT

Study treatment is defined as any active investigational agent and an appropriate placebo identified by the TOC for use in this study.

### 5.1 Regimen, Administration, and Duration

See relevant appendix/appendices for details of investigational agents.

### 5.2 Investigational Agent Formulation, Storage, and Preparation

See relevant appendix/appendices for details of investigational agents.

### 5.3 Supply, Distribution, and Accountability

#### 5.3.1 Investigational Agent Acquisition/Distribution

See relevant appendix/appendices for details of investigational agents.

#### 5.3.2 Investigational Agent Accountability

See relevant appendix/appendices for details of investigational agents.

### 5.4 Concomitant Medications

Whenever a concomitant medication or investigational agent is initiated or a dose changed, investigators must review the concomitant medications and the relevant protocol appendix/appendices, as well as the most recent package insert, Investigator's Brochure, or updated information from DAIDS to obtain the most current information on drug interactions, contraindications, and precautions.

Additional drug information may be found on the ACTG Precautionary and Prohibited Medications Database located at http://tprc.pharm.buffalo.edu/home/di_search/.

5.4.1 Prohibited Medications

Use of hydroxychloroquine (unless used chronically for autoimmune diseases), chloroquine (unless used for a parasitic infection), ivermectin (unless used for a parasitic infection), remdesivir, systemic and inhaled steroids (unless used chronically), and HIV protease inhibitors (unless used chronically for HIV infection).

See relevant appendix/appendices for additional prohibited medications, if applicable.

5.4.2 Precautionary Medications

See relevant appendix/appendices for precautionary medications, if applicable.

## 6.0 CLINICAL AND LABORATORY EVALUATIONS

See appendix/appendices for additions to the following clinical and laboratory evaluations.

### 6.1 Schedule of Evaluations

Table 6.1-1: Schedule of Evaluations Phase II

| Phase II Evaluation | Screening | Study Entry/Day 0 | Day 2 | Day 3 | Day 7 | Day 10 | Day 14 | Day 21 | Day 28 | Week 12 | Week 24 | Premature Study D/C (Before Day 28 Visit) | Premature Study D/C (After Day 28 Visit) |
|---|---|---|---|---|---|---|---|---|---|---|---|---|---|
| Visit Window | | | +/-1 day | | +/-2 days | | | +4 days | | +/-7 days | | | |
| P = In Person Visit<br>R = Remote Visit | P/R | P | R | P | P | R | P | P | P | P | P | P | P |
| Documentation of SARS-CoV-2 Infection | X | | | | | | | | | | | | |
| COVID-19 Symptom Screen | X | X | | | | | | | | | | | |
| Medical/Medication History | X | X | | | | | | | | | | | |
| Smoking Status | | X | | | | | | | | | | | |
| Clinical Assessments | X | X | X | X | X | X | X | X | X | X | X | X | X |
| Collect/Update Secondary Contacts | | X | X | X | X | | X | X | X | X | | | |
| Vital Status Check | | If Participant Cannot be Reached per Section 6.3.8 | | | | | | | | | | | |

| Phase II Evaluation | Screening | Study Entry/Day 0 | Day 2 | Day 3 | Day 7 | Day 10 | Day 14 | Day 21 | Day 28 | Week 12 | Week 24 | Premature Study D/C (Before Day 28 Visit) | Premature Study D/C (After Day 28 Visit) |
|---|---|---|---|---|---|---|---|---|---|---|---|---|---|
| Visit Window | | | +/-1 day | | +/-2 days | | | +4 days | | +/-7 days | | | |
| P = In Person Visit<br>R = Remote Visit | P/R | P | R | P | P | R | P | P | P | P | P | P | P |
| Investigational Agent Administered | | Per Appendix for Investigational Agent | | | | | | | | | | | |
| Study Kit Dispensed | | X | | | | | | | | | | | |
| Participant-Completed Study Diary | | Every Day through Day 28 | | | | | | | | | | | |
| Study Diary Reminder | | Days 1- 28 | | | | | | | | | | | |
| Staff Review of Study Diary | | | X | X | X | | X | X | X | | | X | |
| Retrieval of Study Diary | | | | | | | | | X | | | X | |
| Household Infection Report | | X | | | | | | | X | X | X | X | X |
| Self-Collected Anterior Nasal Swab (In Person) | | X | | X | X | | X | X | X | | | X | |
| Self-Collected Anterior Nasal Swab (Remote) | | Days 1, 2, 4, 5, 6, 8, 9, 10, 11, 12, 13 | | | | | | | | | | | |
| Retrieval of Self-Collected Anterior Nasal Swabs | | | Follow Instructions in MOP | | | | | | | | | X | |
| Staff-Collected NP Swab | | X | | X | X | | X | X | X | | | X | |
| Saliva Collection (Selected Sites) | | X | | X | X | | X | X | X | | | X | |
| Blood Plasma for SARS-CoV-2 RNA | | X | | | X | | X | X | X | | | X | |

| Phase II Evaluation | Screening | Study Entry/Day 0 | Day 2 | Day 3 | Day 7 | Day 10 | Day 14 | Day 21 | Day 28 | Week 12 | Week 24 | Premature Study D/C (Before Day 28 Visit) | Premature Study D/C (After Day 28 Visit) |
|---|---|---|---|---|---|---|---|---|---|---|---|---|---|
| Visit Window | | | +/-1 day | | +/-2 days | | | +4 days | | +/-7 days | | | |
| P = In Person Visit<br>R = Remote Visit | P/R | P | R | P | P | R | P | P | P | P | P | P | P |
| Inflammatory Markers | | X | | | X | | X | X | X | X | X | | |
| Coagulation Markers | | X | | | X | | X | X | X | X | X | | |
| Zinc and Vitamin D Levels | | X | | | | | | | X | | | | |
| Hematology | Per Appendix for Investigational Agent | | | | | | | | | | | | |
| Chemistry | Per Appendix for Investigational Agent | | | | | | | | | | | | |
| Pregnancy Testing | Per Appendix for Investigational Agent | | | | | | | | | | | | |
| Pharmacokinetics | Per Appendix for Investigational Agent | | | | | | | | | | | | |
| Stored Plasma | | X | | | X | | X | X | X | X | X | X | X |
| Stored Serum | | X | | | X | | X | X | X | X | X | X | X |
| Stored PBMCs (Selected Sites) | | X | | | X | | | | X | | X | X | X |

Table 6.1-2: Schedule of Evaluations Phase III

| Phase III Evaluation | Screening | Study Entry/Day 0 | Day 3 | Day 7 | Day 10 | Day 14 | Day 21 | Day 28 | Week 12 | Week 24 | Premature Study D/C (Before Day 28) | Premature Study D/C (After Day 28) |
|---|---|---|---|---|---|---|---|---|---|---|---|---|
| Visit Window | | | +/-1 day | +/-2 days | | | +4 days | | +/-7 days | | | |
| P = In Person Visit<br>R = Remote Visit | P/R | P | R | R | R | R | R | P | P | P | P | P |
| Documentation of SARS-CoV-2 Infection | X | | | | | | | | | | | |
| COVID-19 Symptom Screen | X | X | | | | | | | | | | |
| Medical/Medication History | X | X | | | | | | | | | | |
| Smoking Status | | X | | | | | | | | | | |
| Clinical Assessments | X | X | X | X | X | X | X | X | X | X | X | X |
| Collect/Update Secondary Contacts | | X | | X | | X | X | X | X | | | |
| Vital Status Check | | If Participant Cannot be Reached per Section 6.3.8 | | | | | | | | | | |
| Investigational Agent Administered | Per Appendix for Investigational Agent | | | | | | | | | | | |
| Study Kit Dispensed | | X | | | | | | | | | | |
| Participant-Completed Study Diary | | Every Day through Day 28 | | | | | | | | | | |
| Study Diary Reminder | | Days 1- 28 | | | | | | | | | | |
| Staff Review of Study Diary | | | | X | | X | X | X | | | X | |
| Retrieval of Study Diary | | | | | | | | X | | | X | |

| Phase III Evaluation | Screening | Study Entry/Day 0 | Day 3 | Day 7 | Day 10 | Day 14 | Day 21 | Day 28 | Week 12 | Week 24 | Premature Study D/C (Before Day 28) | Premature Study D/C (After Day 28) |
|---|---|---|---|---|---|---|---|---|---|---|---|---|
| Visit Window | | | +/-1 day | +/-2 days | | | +4 days | | +/-7 days | | | |
| P = In Person Visit<br>R = Remote Visit | P/R | P | R | R | R | R | R | P | P | P | P | P |
| Household Infection Report | | X | | | | | | X | X | X | X | X |
| Self-Collected Anterior Nasal Swab (In Person) | | X | | | | | | X | | | X | |
| Self-Collected Anterior Nasal Swab (Remote) | | | X | X | X | X | X | | | | | |
| Retrieval of Self-Collected Anterior Nasal Swabs | | X | Follow Instructions in MOP | | | | | | | | X | |
| Blood Plasma for SARS-CoV-2 RNA | | X | | | | | | X | | | X | |
| Inflammatory Markers | | X | | | | | | X | | | X | |
| Coagulation Markers | | X | | | | | | X | | | X | |
| Zinc and Vitamin D Levels | | X | | | | | | X | | | X | |
| Hematology | Per Appendix for Investigational Agent | | | | | | | | | | | |
| Chemistry | Per Appendix for Investigational Agent | | | | | | | | | | | |
| Pregnancy Testing | Per Appendix for Investigational Agent | | | | | | | | | | | |
| Pharmacokinetics | Per Appendix for Investigational Agent | | | | | | | | | | | |
| Stored Plasma | | X | | | | | | X | X | X | X | X |
| Stored Serum | | X | | | | | | X | X | X | X | X |

## 6.2 Timing of Evaluations

### 6.2.1 Screening Evaluations

Screening evaluations must occur prior to the participant starting any study medications, treatments, or interventions.

Screening and study entry visit evaluations may be combined unless not allowed per the relevant appendix/appendices. If feasible, screening evaluations may occur remotely.

### 6.2.2 Entry Evaluations

Entry evaluations must occur ≤48 hours after screening evaluations unless otherwise specified.

Participants must be expected to begin study treatment no more than 10 days from self-reported onset of COVID-19 related symptoms or measured fever as noted in section 4.1.1.4.

### 6.2.3 Post-Entry Evaluations

On-Treatment/Post-Treatment Evaluations
Evaluations should occur in the visit windows described in Tables 6.1-1 and 6.1-2.

In person visits will take place at the clinic, at the participant's home, or at another non-clinic location if the site is able to accomplish all of the scheduled study visit evaluations.

Remote visits can take place over the phone or via telemedicine systems approved for use at the site.

Study Completion Evaluations
Participants will be evaluated at week 24.

### 6.2.4 Event-Driven Evaluations

See relevant appendix/appendices for details of any event-driven evaluations.

#### 6.2.5 Discontinuation Evaluations

Evaluations for Randomized Participants Who Do Not Start Investigational Agent/Placebo

All eCRFs must be keyed for the period up to and including the entry visit. Participants who were randomized but do not start investigational agent or placebo will be prematurely discontinued from the study and will not be followed.

Premature Treatment Discontinuation Evaluations
Participants who discontinue investigational agent or placebo early should remain on study and all evaluations should be performed as outlined in Tables 6.1-1 and 6.1-2.

Premature Study Discontinuation Evaluations
Participants who discontinue study participation should have premature study discontinuation evaluations, as outlined in Tables 6.1-1 and 6.1-2 and the relevant appendix/appendices, prior to being taken off the study, unless the reason for premature study discontinuation was that they did not start investigational agent or placebo.

### 6.3 Instructions for Evaluations

Sites must follow PPD source document guidelines.

All eCRFs for a given visit must be keyed within 5 days, except for the AEs noted in section 7.2, which should be entered within 72 hours.

All evaluations below are for both Phase II and III unless otherwise noted.

All stated evaluations are to be recorded on the eCRF unless otherwise specified. Refer to section 7.0 for information on reporting of adverse events.

In the event of hospitalization, targeted physical examination, study diary entry and review, and specimen collection do not need to be completed during hospitalization but should be restarted after discharge. Other evaluations should be performed as feasible, including ascertainment of interventions, including medications received, and outcomes of interest/study endpoints.

#### 6.3.1 Documentation of SARS-CoV-2 Infection

Section 4.1.1.3 specifies assay requirements for SARS-CoV-2 infection documentation. SARS-CoV-2 infection documentation is recorded on the eCRF. If a viral load level is available, it should be recorded as well.

See the MOP for further guidance.

### 6.3.2 COVID-19 Symptom Screen

Participants will be asked about their first symptoms related to COVID-19 and their current symptoms.

The time from symptom onset at anticipated study entry (≤5 days versus >5 days) should be recorded.

### 6.3.3 Medical History

At Screening and updated at Study Entry, the following diagnoses should be recorded regardless of when the diagnosis was made, except where noted:

- autoimmune disease
- pulmonary embolus
- deep venous thrombosis
- HIV infection
- cancer (exclusive of basal/squamous cell skin cancer)
- acute viral respiratory infection (influenza, parainfluenza, respiratory syncytial virus, rhinovirus) within the previous 14 days (if known by participant)
- chronic lung disease
- asthma requiring daily inhaled medication
- obesity (body mass index [BMI] >35; may be based on self-report of height and weight)
- hypertension
- cardiovascular disease (including history of stroke)
- diabetes
- chronic kidney disease
- chronic liver disease

The participant’s risk category for COVID-19 progression (“high” vs “low” risk) should be recorded. If participant meets the criteria for “high” risk, all high risk criteria that are met should be recorded.

Any allergies to any medications and their formulations must also be documented.

See appendix/appendices for additional elements of the medical history that should be recorded.

### 6.3.4 Medication History

A medication history must be present, including start and stop dates. The table below lists the medications that must be included in the history at screening and updated at entry.

Table 6.3.5-1: Medication History

| Medication/Category | Timeframe |
|---|---|
| All prescription drugs | Last 7 days |
| Prescription drugs for high blood pressure | Last 3 months |
| Prescription drugs for diabetes and pre-diabetes | Last 3 months |
| Prescription drugs for lung disease | Last 3 months |
| Prescription drugs for heart disease | Last 3 months |
| Prescription drugs for autoimmune disease | Last 3 months |
| Cancer chemotherapy | Last 3 months |
| Antiretroviral therapy | Last 3 months |
| Immune-based therapy | Last 3 months |
| Blinded investigational agent | Last 12 months |
| CoV-related vaccines or treatments | Complete history |
| Hydroxychloroquine | Complete history |
| Antibiotics | Last 3 months |
| Anti-parasitics | Last 3 months |
| Alternative therapies | Last 3 months |
| Dietary supplements<br>(including zinc and vitamins C and D) | Last 3 months |

### 6.3.5 Smoking Status

A Smoking Status questionnaire will be completed as part of medical history and recorded on the eCRF.

### 6.3.6 Clinical Assessments

Targeted Physical Examination
A targeted physical examination is done at all in-person visits. It is to include vital signs (weight, temperature, pulse, blood pressure, and resting peripheral oxygen saturation), and is to be driven by any previously identified or new adverse event/targeted condition that the participant has experienced.

Supplemental oxygen use will be recorded at each in-person visit.

At study entry, if peripheral oxygen saturation is <92% on usual supplemental oxygen requirements, the participant should be referred for emergency department evaluation and should not initiate investigational product.

During follow-up in-person visits (after Entry) through Day 28, peripheral oxygenation saturation measures <96% should be reviewed by an investigator and referral for medical attention made at the discretion of the investigator.

See appendix/appendices for any additional elements needed for the targeted exam.

Post entry, see section 8.3 for collection requirements for pregnancy.

Concomitant Medications

Post entry, the following new and discontinued concomitant medications must be recorded:

- high blood pressure medications
- steroids or other immunosuppressive or immunomodulatory medication
- non-steroidal anti-inflammatory drugs (NSAIDS)
- chemotherapy
- antibiotics, antifungals, antiparasitics, and antivirals (including antiretrovirals)
- anticoagulants
- antiplatelets
- any agent felt to have potential COVID-19 activity (including hydroxychloroquine, chloroquine, ivermectin, and HIV protease inhibitors)
- inhalers
- medications for symptoms of COVID-19, including aspirin, ibuprofen, acetaminophen, zinc, dietary supplements, herbal remedies, decongestants, cough suppressants, and anti-histamines.

Assessment for Adverse Events

Beginning at entry, participants will be assessed at every visit (remote or in-person) for any new signs or symptoms and the relationship to study treatment.

Investigational Agent Modifications

Post entry, record any initial dose of treatment, modification to treatment, treatment interruption, and permanent discontinuation of treatment, and the reason for the modification, interruption, or discontinuation.

#### 6.3.7 Collect/Update Secondary Contacts

Sites will capture contact information for at least two individuals that the site can contact if the participant cannot be reached (e.g., spouse, friend, neighbor). Sites will also request health care provider contact information and hospital(s) that the participant is likely to go to if they get sick.

Contact information for secondary contacts or health care provider will not be recorded on any eCRF.

At study entry only, sites will record the participant’s home address in site records (it will not be reported on an eCRF).

6.3.8 Vital Status Check

If a participant cannot be reached after two attempts 24 hours apart, then their listed secondary contact person(s) or health care provider will be contacted for a check of the participant’s vital status.

6.3.9 Investigational Agent Administered

See relevant appendix/appendices for dispensing/administration details.

6.3.10 Study Kit Dispensed

The kit will include:

- copy of informed consent
- information about the study
- instructions on study procedures
- pocket/wallet card with site staff contact information
- instructions on what to do if participants have worsening symptoms/become hospitalized
- thermometer
- swabs for self-collected anterior nasal swabs with storage and transport materials
- study diary (see below)

6.3.11 Study Diary

<u>Participant-Completed Study Diary</u>
Participants will be asked to keep a log of symptoms, temperature, new medications they are taking for COVID-19 symptoms, verification of self-collection of anterior nasal swabs, and major events such as urgent visit to an emergency room or clinic and hospitalization in their study diary. This log will be completed on paper or electronically, if appropriate electronic systems are available.

At study entry, participants will complete the study diary with site staff prior to initiating investigational agent/placebo. Participants will be asked to complete subsequent entries in the study diary each evening on days 1 through 28 (the entry on day 28 may be completed with the site staff during the day 28 visit, if the visit occurs on day 28 versus another day in the day 28 visit window).

The study diary will ask participants to report on the following symptoms:

- feeling feverish
- cough
- shortness of breath or difficulty breathing at rest or with activity
- sore throat
- body pain or muscle pain/aches

- fatigue
- headache
- chills
- nasal obstruction or congestion
- nasal discharge (runny nose)
- nausea or vomiting
- diarrhea
- any other COVID-19 symptoms they experience

Symptom severity scoring (0-3, ranging from absent to severe) will be based on the participant's self-assessment. Participants should use the same self-determined approach to severity scoring each day.

Participants will also record:

- their self-collected daily temperature
- if they self-collected anterior nasal swabs on the required days

Study Diary Reminder and Staff Review of Study Diary

Participant will be contacted every day on days 1-28 and reminded to complete their study diary. This reminder may be by telephone, text message, email, or other method for which the participant provides permission. A direct response from the participant is not required.

The study diary will be reviewed by study staff in person or remotely with each participant according to the schedule in Tables 6.1-1 and 6.1-2. If an appropriate electronic system is available, the participant's diary entries will automatically be captured in the eCRF. If such a system is not available, the study staff will record the participant's answers on the study diary eCRF. If the participant uses a paper diary and it is feasible, prior to or during the remote study visits, sites will ask the participant to send images of each of their study diary entries to be reviewed at the next study contact.

Participants who report worsening symptoms from any cause during the trial may be referred to their health care provider or closest emergency room. Such instances will be recorded at the time of the notification, and during follow-up to assess study endpoints, i.e., hospitalization or death.

Retrieval of Study Diary

If the participant uses a paper diary and the day 28 visit cannot be conducted in person, arrangements should be made for the participant to mail the study diary back to the site after the day 28 visit is completed. Prior to mailing the diary, participants may send images of the study diary entries if not yet done.

6.3.12 Household Infection Report

At Study Entry/Day 0, participants will be asked if anyone who resides in their household, defined as sharing indoor living space or housekeeping space (i.e., kitchen, dining area, or bathroom) has been diagnosed with SARS-CoV-2 infection, and the response recorded on the eCRF.

Post entry, participants will be asked if any new household members have been diagnosed with SARS-CoV-2 infection, and the response recorded on the eCRF.

6.3.13 Virologic Studies

Anterior nasal and NP swabs, saliva, and plasma will be collected for qualitative and quantitative SARS-CoV-2 RNA detection, performed in near real-time.

Additional information can be found in the MOP and the LPC.

Self-Collected Anterior Nasal Swabs (In Person and Remote) (Phase II and III)
Participants will self-collect anterior nasal swabs. Participants will be instructed by study staff and will obtain the day 0 swab while observed by study staff. This swab should be collected prior to the first dose of investigational agent.

In phase II, on days when an in-person visit occurs, the swab will be self-collected at the clinic on that day. On days without an in-person visit, the swabs will be self-collected by the participant on their own in the evening, when completing the study diary and temperature measurement. Participants will turn in their self-collected (remote) swabs at their next in-person visit.

In phase III, nasal swabs will be self-collected by the participant on their own in the evening. Nasal swabs will be stored at home as per the LPC.

Retrieval of Self-Collected Nasal Swabs (Phase II and III)
Site staff will retrieve the nasal swabs collected by the participants at home as per the LPC. The swabs will be processed, stored, and shipped to the central laboratory as per the LPC.

Staff-Collected NP Swab (Phase II only)
NP swabs will be collected during in-person visits after the self-collected nasal swab. At study entry, the sample should be collected prior to the first dose of investigational agent.

Saliva Collection (Phase II only)
Saliva will be collected only at select sites. Saliva will be collected during in-person visits after the NP swab. At study entry, the sample should be collected prior to the first dose of investigational agent.

Blood Plasma for SARS-CoV-2 RNA (Phase II and III)
Blood plasma will be collected during in-person visits. At study entry, the sample

should be collected prior to the first dose of investigational agent.

6.3.14 Laboratory Evaluations

The following laboratory evaluations are for all investigational agents. If additional measures are needed, these are detailed in the relevant investigational agent appendix.

Refer to the LPC for details of collection, processing, and shipping.
At screening, entry, and post-entry, all laboratory values must be recorded unless otherwise specified in the relevant appendix/appendices.

At study entry, blood samples should be collected prior to initiation of the investigational agent.

Blood can be collected outside of a clinic setting (e.g., home) per relevant investigational agent appendix.

Inflammatory Markers
Lactate dehydrogenase, C-reactive protein, ferritin, and D-dimer will be performed.

Coagulation Markers
PT, PTT, INR, and fibrinogen will be performed.

Vitamin Levels
Zinc and vitamin D levels will be performed.

Hematology
See relevant appendix/appendices for testing requirements.

Chemistry
See relevant appendix/appendices for testing requirements.

Pregnancy Testing
See relevant appendix/appendices for testing requirements.

6.3.15 Pharmacokinetics

Pharmacokinetic sampling will be performed per the relevant appendix/appendices.

6.3.16 Stored Samples

Collected plasma, sera, or PBMC will be used to assess SARS-CoV-2 virologic and immune responses. Additional samples will be collected for agent-specific evaluations per the relevant appendix/appendices.

Stored Plasma
Blood plasma will be collected and stored for future testing, including:

- immunologic studies including markers linked to systemic inflammation (IL-6, TNF-a), inflammasome activation ( IL-1beta, IL-18), interferon pathways (IP-10, type I interferon), neutrophil activation (MPO), monocyte activation (sCD14), as well as markers associated with coagulation or endothelial cell dysfunction (VWF, P-selectin, tissue factor)
- SARS-CoV-2 seroconversion and antibody titers (among seroconverters)
- full viral genome sequencing will be performed from select samples that are detectable for SARS-CoV-2 RNA to assess for signs of viral evolution and resistance to the investigational agent or immune responses. If sequence analysis suggests viral escape from the investigational agent (e.g. mutations in putative binding regions or epitopes), then phenotypic analyses may be pursued.

Stored Serum
Blood sera will be collected and stored for future testing, including:

- total and neutralizing antibody assays

Stored Peripheral Blood Mononuclear Cells (PBMCs)
PBMCs will be collected only at select sites. PBMC processing must be done in an IQA-approved lab. PBMCs will be stored for future testing, which may include the following:

- cellular immune responses between treatment and control samples, including assessment of T-cell responses to SARS-Cov-2 protein (phase II: days 0, 7, 28, and week 24)
- cellular activation/exhaustion phenotypes among innate or adaptive immune cells (phase II: days 0, 7, 28, and week 24)
- host genetics

## 7.0 ADVERSE EVENTS AND STUDY MONITORING

See relevant appendix/appendices for any modifications to recording of AEs and study monitoring.

### 7.1 Definitions of Adverse Events

Adverse Event
An adverse event (AE) is any unfavorable and unintended sign (including an abnormal laboratory finding), symptom, or diagnosis that occurs in a study participant during the conduct of the study REGARDLESS of the attribution (i.e., relationship of event to medical treatment/investigational agent/device or procedure/intervention). This includes any occurrence that is new in onset or aggravated in severity or frequency from the baseline condition.

The scale used in the Study Diary for participant symptoms does NOT equate to the AE grading as found in the Division of AIDS Table for Grading the Severity of Adult and Pediatric Adverse Events (DAIDS AE Grading Table), corrected Version 2.1, July 2017.

Sites should grade participant symptoms as they normally would according to the DAIDS AE Grading Table.

Serious Adverse Events (SAEs)
An SAE is defined as any untoward medical occurrence that results in any of the following outcomes:

- results in death
- is life-threatening
- requires inpatient hospitalization or prolongation of existing hospitalization
- results in persistent or significant disability/incapacity
- is a congenital anomaly/birth defect.
- is an important medical event that may not be immediately life threatening or result in death or hospitalization but may jeopardize the participant or may require intervention to prevent one of the other outcomes listed in the definition above).

Adverse Events of Special Interest
An adverse event of special interest (AESI) (serious or nonserious) is defined as an AE or SAE of scientific and medical concern specific to the investigational agent, for which ongoing monitoring and rapid communication by the investigator to the sponsor could be appropriate.

See appendix/appendices for AESIs related to specific investigational agents.

Suspected Unexpected Adverse Events
A Suspected Unexpected Serious Adverse Reaction (SUSAR) is defined as a serious adverse reaction, the nature or severity of which is not consistent with the applicable product information (e.g., Investigator’s Brochure for an unapproved investigational product).

## 7.2 Eliciting and Documenting Adverse Events

Adverse events will be assessed beginning at Entry/Day 0 and through week 24.

If the investigator learns of any SAE, including a death, at any time after a participant has been discharged from the study, and he/she considers the event to be reasonably related to the investigational agent or study participation, the investigator must promptly notify the sponsor.

Serious AEs that occur after week 24 need not be reported unless the investigator considers them related to the investigational product.

At every study visit, participants will be asked a standard nonleading question to elicit any medically related changes in their well-being. They will also be asked if they have been hospitalized, had any accidents, used any new medications, or changed concomitant medication regimens (both prescription and OTC medications).

In addition to participant observations, AEs identified from any study data (e.g., laboratory values, physical examination findings, or identified from review of other documents [e.g., participant diaries]) that are relevant to participant safety will be documented on the AE page in the eCRF.

#### 7.2.1 Assessment of Severity

The severity, or intensity, of an AE refers to the extent to which an AE affects the participant's daily activities.

All AEs that are reported must have their severity graded. To grade AEs, sites must refer to the Division of AIDS Table for Grading the Severity of Adult and Pediatric Adverse Events (DAIDS AE Grading Table), corrected Version 2.1, July 2017, which can be found on the DAIDS RSC website at https://rsc.niaid.nih.gov/clinical-research-sites/daids-adverse-event-grading-tables.

#### 7.2.2 Assessment of Causality

If there is any doubt as to whether a clinical observation is an AE, the event should be reported.

The relationship or association of the investigational agent/placebo in causing or contributing to the AE will be characterized using the following classification and criteria:

- Unrelated: There is no association between the investigational agent/placebo and the reported event.
- Related: A causal relationship exists between administration of the investigational agent/placebo and the AE, and other conditions (concurrent illness, progression/expression of disease state, or concurrent medication reaction) do not appear to explain the event.

### 7.3 Recording Adverse Events

Post entry, the following non-lab AEs must be recorded on the eCRFs within 72 hours:

- Grade ≥1 AEs that are deemed related to study product as determined by the site investigator (phase II)
- Grade ≥2 AEs (phase II and III)
- AEs that led to a change in study treatment/intervention regardless of grade
- AEs meeting serious adverse event (SAE) definition or expedited adverse event (EAE) reporting requirement

- AESIs

Information to be collected includes the following:
- study product group (investigational agent/placebo)
- dose
- event term
- time of onset
- investigator-specified assessment of severity and relationship to the investigational product
- time of resolution of the event
- seriousness
- any required treatment or evaluations
- outcome

Adverse events resulting from concurrent illnesses, reactions to concurrent illnesses, reactions to concurrent medications, or progression of disease states must also be reported. All AEs will be followed to adequate resolution. The MedDRA will be used to code all AEs.

Any medical condition that is present at the time that the participant is screened but does not deteriorate should not be reported as an AE. However, if it deteriorates at any time during the study, it should be recorded as an AE.

7.3.1 Reporting Serious Adverse Events

Any AE that meets SAE criteria must be reported to PPD, Inc., immediately (i.e., within 24 hours of the time that the site personnel first learn about the event). The following contact information is to be used for SAE reporting:

| |
|---|
| 24-Hour Pharmacovigilance Hotline:<br>US phone: 1 888 483 7729<br>Fax: 1 888 529 3580 |

Further reporting instructions are provided in the MOP.

The sponsor has a legal responsibility to notify both the local regulatory authority and other regulatory agencies about the safety of an investigational product under clinical investigation. The sponsor will comply with country-specific regulatory requirements relating to safety reporting to the regulatory authority, institutional review board/independent ethics committee (IRB/IEC), and investigators.

An investigator who receives an investigator safety report describing an SAE or other specific safety information from the sponsor will review and then file it as appropriate and will notify the IRB/IEC, if appropriate according to local requirements.

#### 7.3.2 Reporting Adverse Events of Special Interest

Any AE that meets AESI criteria (section 7.1) must be reported immediately (i.e., within 24 hours of the time that the site personnel first learn about the event).

#### 7.3.3 Reporting Suspected Unexpected Serious Adverse Reactions

The sponsor will promptly evaluate all SUSARs and nonserious AEs of special interest (defined in section 7.1) against cumulative product experience to identify and expeditiously communicate possible new safety findings to investigators, IRBs/IECs, and applicable health authorities based on applicable legislation.

To determine reporting requirements for single AE cases, the sponsor will assess the expectedness of these events using the investigational agent Investigator's Brochure.

The sponsor will compare the severity of each SUSAR and the cumulative event frequency reported for the study with the severity and frequency reported in the applicable reference document.

Reporting requirements will also be based on the investigator's assessment of causality and seriousness, with allowance for upgrading by the sponsor as needed.

### 7.4 Follow-up of Participants Reporting Adverse Events

All AEs must be reported in detail on the appropriate page in the eCRF and followed to satisfactory resolution, until the investigator deems the event to be chronic or not clinically significant, the event is considered to be stable, or the participant is lost to follow-up.

### 7.5 Study Monitoring

The protocol team will monitor the conduct and safety of the study via regular summaries of *accrual, study discontinuation, data completeness, and adverse events.*

The DAIDS Clinical Representative will review and assess select AE reports for potential impact on the study participant safety and protocol conduct as per DAIDS policies, guidance documents, and SOPs as applicable.

The DSMB will conduct interim reviews for safety. Enrollment will pause and the DSMB will review any death that occurs on study that is deemed related to study product as determined by the site investigator. A pause in enrollment for that study product group (investigational agent/placebo) will also occur and the DSMB will review if two participants experience a Grade 4 AE that is deemed related to study product as determined by the site investigator.

See section 10.0 for statistical and other considerations related to interim monitoring.

Detailed plans for study monitoring are outlined in a Safety Management Plan.

See relevant appendix/appendices for additional monitoring procedures.

## 8.0 CLINICAL MANAGEMENT ISSUES

The following guidance pertains to all investigational agents; however, additional guidance for particular agents are included in the appendix relevant for each investigational agent.

### 8.1 Toxicity

Criteria for participant management, dose adjustments and discontinuation, or changes in treatment will be described only for toxicities attributable to the investigational agents, when applicable, and are included in the appendix/appendices.

The grading system for drug toxicities is located in the Division of AIDS Table for Grading the Severity of Adult and Pediatric Adverse Events (DAIDS AE Grading Table), corrected Version 2.1, July 2017, which can be found on the DAIDS RSC website at https://rsc.niaid.nih.gov/clinical-research-sites/daids-adverse-event-grading-tables.

NOTE: The protocol team must be notified within 72 hours regarding toxicities that result in a change in study regimen (follow the directions described in the Study Management section).

For all agents evaluated in this trial, if a participant develops a Grade 4 AE that is related to the study product as determined by the site investigator, no further doses of the study treatment should be administered.

It is possible that some participants will experience transient or prolonged AEs during the study. As some of the visits will be conducted remotely, AEs will often be assessed remotely and unplanned study visits scheduled if deemed necessary by the site investigator. For any concerning AEs that are felt to require clinical intervention, participants should be instructed to contact their health care provider or seek urgent or emergent care, or 911 should be called, as appropriate.

Treatment may be discontinued without contacting the protocol team in advance, but the protocol team should be notified within 24 hours of parenteral and 72 hours of oral treatment discontinuation (follow the directions described in the Study Management section). This includes an interruption in administration for single-dosed agents.

### 8.2 Management of Side Effects

See relevant appendix/appendices for additional details on the management of side

effects.

#### 8.2.1 Overdose

An overdose is any dose of study treatment given to a participant or taken by a participant that exceeds the dose described in the protocol.

Any overdose must be reported to the PPD Drug Safety Center within 24 hours (follow the directions described in the Study Management section). The overdose itself is not to be reported as an AE. However, any AEs associated with the overdose are to be reported on relevant AE/SAE sections in the eCRF.

In the event of an overdose, the site investigator should:

1. Contact the protocol team immediately (follow the directions described in the Study Management section).
2. Closely monitor the participant for any AE/SAE and laboratory abnormalities.
3. Obtain a plasma sample for PK analysis within 3 days from the date of the last dose of investigational agent/placebo if requested by the medical monitor.
4. Document the quantity of the excess dose as well as the duration of the overdose in the eCRF.

Decisions regarding dose interruptions or modifications will be made by the site investigator in consultation with the medical monitor based on the clinical evaluation of the participant.

### 8.3 Pregnancy

The use of investigational agents in pregnancy will vary depending upon agent. The ability to continue or need to discontinue investigational agent in event of pregnancy is outlined in the relevant appendix/appendices.

### 8.4 Breastfeeding

The use of investigational agent in breastfeeding participants who meet inclusion criteria for the study will vary depending upon agent and is outlined in the relevant appendix/appendices.

## 9.0 CRITERIA FOR DISCONTINUATION

Participants may discontinue from the investigational product or withdraw from the study at any time and for any reason without prejudice to their future medical care by the investigator or at the study site. Every effort should be made to keep participants in the study. The reasons for participants discontinuing the investigational product and/or withdrawing from the study will be recorded.

## 9.1 Permanent and Premature Treatment Discontinuation

- Drug-related toxicity mandating discontinuation (see appendix/appendices).
- Participant experiencing an SAE that is considered related to investigational agent.
- Requirement for prohibited concomitant medications (see section 5.4).
- Request by participant to terminate treatment.
  NOTE: The reason for treatment discontinuation should be documented (e.g., concern for AE, lack of efficacy, or other reason).
- Clinical reasons believed life threatening by site clinical staff, even if not addressed in the Toxicity section of the protocol.
- Any additional indications are outlined in the relevant appendix/appendices.

## 9.2 Premature Study Discontinuation

- Failure to initiate investigational agent at the study entry visit.
- Request by the participant to withdraw consent.
- Request of the health care provider if they think the study is no longer in the best interest of the participant.
- At the discretion of the IRB/EC, FDA, NIAID, ACTG, Office for Human Research Protections (OHRP), other government agencies as part of their duties, investigator, or industry supporter.
- Any additional indications are outlined in the relevant appendix/appendices.

In the event that a participant prematurely discontinues from the study, unless they have withdrawn consent or never initiated investigational agent/placebo, sites will attempt to obtain information regarding vital status (including date last seen alive, hospitalization, date of death, and primary cause of death) from other sources (e.g., family members, other designated secondary contacts, or clinic records). See the MOP for further guidance.

# 10.0 STATISTICAL CONSIDERATIONS

## 10.1 General Design Issues

There are two major benefits of the proposed trial design. First, the platform trial aspect of the design allows for efficient evaluation of multiple investigational agents compared to concurrently randomized participants (who were eligible for a particular agent) in a combined placebo control group. Second, the transition from phase II evaluation to phase III evaluation for graduating investigational agents provides for more rapid evaluation of an investigational agent than having separate phase II and phase III trials. In both phase II and phase III evaluation, the intent is to focus on comparisons between each investigational agent and the placebo control, and not on comparisons among investigational agents. Control of Type I error rate will be undertaken separately for each investigational agent rather than across all investigational agents (so not the experiment-wise or family-wise error rate).

There is very little data available for ambulatory persons with COVID-19 and so this section provides information about the general approach that will be pursued with initial agents evaluated in this study. However, it is expected that this study will rapidly provide key information about clinical and virologic outcomes and their inter-relationships, and so the study design may be modified as this information accumulates. In particular, based on this accumulating information, a Bayesian framework will be developed to improve the process for deciding which agents graduate from phase II to phase III evaluation. This information will also be important for re-evaluation of sample size requirements for both phases. The initial Bayesian analytical framework is described in Appendix I.

It is expected the study will need to undergo a significant protocol amendment if an agent is shown to be effective in reducing hospitalization/death in the phase III evaluation or a new standard of care for the outpatient population is established outside of this study. Therefore, this possibility is not considered in this section.

## 10.2 Outcome Measures

Primary and secondary outcome measures listed below will be addressed in the study's primary Statistical Analysis Plan, which will define the content of the Primary Analysis Report of outcomes through day 28 of follow-up and a Secondary Analysis Report of further outcomes through to week 24. These reports will form the basis for the main study manuscript(s) and results reporting to ClinicalTrials.gov.

### 10.2.1 Phase II: Primary Outcome Measures

10.2.1.1 Clinical (Symptom Duration): Duration of targeted COVID-19 associated symptoms from start of investigational agent (day 0) based on self-assessment. Duration defined as the last day on or before study day 28 when any symptoms scored as moderate or severe at study entry (pre-treatment) are still scored as moderate or severe (i.e., not mild or absent), or any symptoms scored as mild or absent at study entry are scored as mild or worse (i.e., not absent). The targeted symptoms are fever or feeling feverish, cough, shortness of breath or difficulty breathing at rest or with activity, sore throat, body pain or muscle pain/aches, fatigue, headache, chills, nasal obstruction or congestion, nasal discharge (runny nose), nausea or vomiting, and diarrhea. Each symptom is scored daily by the participant as absent (score 0), mild (1) moderate (2) and severe (3).

10.2.1.2 Virologic: At each of days 3, 7, 14, 21 and 28, detection (detectable versus undetectable) of SARS-CoV-2 RNA from site-collected NP swabs.

10.2.1.3 Safety: New Grade 3 or higher AE through 28 days.

10.2.2 Phase III: Primary Outcome Measures

10.2.2.1 Efficacy: Death from any cause or hospitalization during the 28-day period from and including the day of the first dose of investigational agent or placebo. Hospitalization is defined as ≥24 hours of acute care, in a hospital or similar acute care facility, including Emergency Rooms or temporary facilities instituted to address medical needs of those with severe COVID-19 during the COVID-19 pandemic.

10.2.2.2 Safety: New Grade 3 or higher AE through 28 days.

10.2.3 Secondary Outcome Measures

The clinical primary outcome measure in phase II (symptom duration) will also be assessed in phase III as a secondary outcome measure.

The primary outcome measure in phase III (death from any cause or hospitalization through 28 days) will also be assessed in phase II as a secondary outcome measure.

The following secondary outcome measures will also be assessed:

10.2.3.1 Phases II and III: Detection (detectable versus undetectable) and level of SARS-CoV-2 RNA from participant-collected nasal swabs through day 28.

10.2.3.2 Phases II and III: COVID-19 severity ranking based on symptom severity scores over time during the 28-day period from and including the day of the first dose of investigational agent or placebo, hospitalization, and death. For participants who are alive at 28 days and not previously hospitalized, the severity ranking will be based on their area under the curve AUC of the symptom score associated with COVID-19 disease over time (through 28 days counting day 0 as the first day) defined as the sum of scores for the targeted symptoms in the participant’s study diary (each individual symptom is scored from 0 to 3). Participants who are hospitalized or who die during follow-up through 28 days will be ranked as worse than those alive and never hospitalized as follows (in worsening rank order): alive and not hospitalized at 28 days; hospitalized but alive at 28 days; and died at or before 28 days.

10.2.3.3 Phases II and III: Progression through day 28 of one or more COVID-19-associated symptoms to a worse status than recorded in the study diary at study entry, prior to start of investigational agent or placebo.

10.2.3.4 Phases II and III: Duration of fever through day 28 defined as the last day in the participant’s study diary on which a temperature greater

than 37.8°C was recorded or a potentially antipyretic drug, such as acetaminophen or ibuprofen, was taken.

10.2.3.5 Phases II and III: Time to self-reported return to usual (pre-COVID-19) health as recorded in a participant's study diary through day 28.

10.2.3.6 Phases II and III: Death from any cause or hospitalization during the 24-week period from and including the day of the first dose of investigational agent.

10.2.3.7 Phase II only: Oxygen saturation (i.e., pulse oximeter measures) as a quantitative measure and categorized as <96 versus ≥96% through day 28.

10.2.3.8 Phase II only: Area under the curve and above the assay lower limit of quantification of quantitative SARS-CoV-2 RNA over time from site-collected NP swabs at days 0, 3, 7, 14, 21 and 28, from saliva samples at days 0, 3, 7, 14, 21, and 28, and from self-collected nasal swabs daily at days 0-14 and at days 21 and 28.

10.2.3.9 Phase II only: Level (quantitative) of SARS-CoV-2 RNA from site-collected NP swabs at days 3, 7, 14, 21, and 28.

10.2.3.10 Phase II only: Detection (detectable versus undetectable) and level of SARS-CoV-2 RNA from saliva at days 3, 7, 14, 21, and 28.

10.2.3.11 Phase II only: New Grade 2 or higher AE through 28 days, and through week 24.

10.2.3.12 Phase III only: New Grade 3 or higher AE through week 24.

10.2.3.13 Phase II only: Pharmacokinetic measures will be defined in the agent-specific appendices.

10.2.4 Other Outcome Measures

10.2.4.1 Phases II and III: Worst clinical status assessed using ordinal scale among participants who become hospitalized. Ordinal scale defined as:

- death
- hospitalized, on invasive mechanical ventilation or ECMO;
- hospitalized, on non-invasive ventilation or high flow oxygen devices;
- hospitalized, requiring supplemental oxygen;
- hospitalized, not requiring supplemental oxygen (COVID-19 related or otherwise)

10.2.4.2 Phases II and III: Duration of hospital stay among participants who become hospitalized.

10.2.4.3 Phases II and III: ICU admission (yes versus no) among participants who become hospitalized.

10.2.4.4 Phases II and III: Duration of ICU admission among participants who are admitted to the ICU.

10.2.4.5 Phases II and III: New SARS-CoV-2 positivity among household contacts through to 28 days and through to 24 weeks from start of investigational agent or placebo.

10.2.4.6 Phases II and III: Detection (detectable vs. undetectable) and level of SARS-CoV-2 RNA in blood.

10.2.4.7 Phases II and III: Hematology, chemistry, coagulation, and inflammatory markers through 28 days from start of investigational agent.

10.2.4.8 Phases II and III: Plasma markers of inflammation and antibody responses to SARS-CoV-2 infections, measured in blood in all phase II participants and in a subset of phase III participants per relevant appendix.

10.2.4.9 Phase II and III: Viral resistance (to be defined at the time of laboratory analysis).

10.2.4.10 Phase II only: Immune cell phenotypes and T and B cell responses to SARS-CoV-2 measured in PBMCs (to be defined at the time of laboratory analysis).

## 10.3 Randomization and Stratification

At any time that enrollment is ongoing, participants will be randomized in two steps with the ultimate intent of having approximately equal numbers on a given investigational agent and on the control group for that agent (i.e., combining participants who were eligible to receive the agent but who were randomized to any of the available placebos). Participants may be randomized to agents that are in phase II evaluation and to agents that are in the phase III evaluation.

To allow for the possibility that each agent may have a matching placebo for blinding, the randomization will be undertaken in two steps (see example in Figure 3.0-3). First, participants at a site will be randomized in approximately equal numbers to groups corresponding to the investigational agents that they are eligible to receive which are under study at that site. For example, when enrollment is ongoing for Agents A, B and C

at a given site, participants will be randomized to Groups A, B and C if they are eligible to receive any of Agents A, B and C. Participants who are only eligible to receive two of the three agents (e.g., Agents A and B) would only be randomized to the two respective groups (e.g., Groups A and B). Participants who are only eligible for one agent (e.g., Agent A) would be assigned to the respective group (e.g., Group A).

Immediately following the first randomization, participants will be randomized within their assigned group to receive the interventional agent or the matching placebo for that agent. For example, in Group A, participants would be randomized to receive Agent A or the placebo for Agent A. In this second randomization, the ratio of assignment to interventional agent or placebo will be r:1 where r is the number of agents a given participant is eligible to receive (this is the same as the number of Groups that participant could have been randomized to in the first randomization). For example, if a participant was eligible to receive any of three agents, then r=3 and the ratio of assignment to agent or placebo will be 3:1. This two-step randomization process will achieve approximately equal numbers being assigned to an investigational agent and its concurrent control group (comprised of all concurrently enrolled placebo groups combined, restricted to participants who were eligible to receive that agent).

Both randomization steps will be stratified (using blocked randomization) by time from symptom onset (≤ versus >5 days) and “high” versus “low” risk of progression to severe COVID-19, as defined in the Schema, Stratification. There will therefore be four strata.

## 10.4 Sample Size

### 10.4.1 Phase II

The sample size for phase II is initially justified by standard (frequentist) power calculations in which the true difference between an interventional agent and placebo is assumed to be the targeted difference in the Bayesian probability statement for the graduation rules. As data become available concerning the distribution of outcomes in the study population, the sample size and power considerations may also be evaluated to address power to graduate for given true differences between randomized groups based on the Bayesian probability statements.

The phase II evaluation of an investigational agent involves the comparison of two primary outcomes (undetectable SARS-CoV-2 RNA at days 3, 7, 14, 21 and 28; and symptom duration) among participants randomized to that agent versus participants concurrently randomized to the placebo. This evaluation will involve approximately 110 participants randomized to the investigational agent and approximately 110 participants concurrently randomized to the control group for that agent (combined across one or more concurrently randomized placebo groups). The choice of sample size has been chosen to give high power to identify an active agent mainly based on the primary virologic outcome so we describe that first. As there are very limited data about variability in symptom durations in the outpatient COVID-19 population, we then provide

some underpinning as to the possible power of the study to detect an effect on symptom duration. In addition, as this is the phase II component of the study and hence there will be further evaluation of an agent that graduates to phase III, no adjustment is made for the multiplicity of outcomes being assessed for a given investigational agent (or across investigational agents).

Virologic Outcome

The percentage of participants with undetectable SARS-CoV-2 RNA in NP swabs will be compared between an investigational agent and placebo control at each of days 3, 7 14, 21, and 28. It is uncertain what might be the percentage undetectable at each of these times in the population being studied, and is likely to depend on the time since onset of symptoms at which participants are enrolled. However, a 20% absolute increase in percentage of participants with undetectable SARS-CoV-2 is thought to be relevant. For example, in a clinical trial comparing the combination of interferon beta-1b, ribavirin, lopinavir/ritonavir (n=86) to lopinavir/ritonavir alone (n=41) in hospitalized COVID-19 patients in China, there was both a difference in clinical outcomes and more than a 20% reduction in undetectable virus at about 7 days (with the caveat that this does not establish that a difference in virologic outcome is a surrogate for a difference in clinical outcome) [8]. The median time to undetectable virus was 7 versus 14 days in this trial (based on daily NP swabs obtainable in the hospitalized setting), indicating that 50% of participants were undetectable at 7 and 14 days in the two groups.

With a phase II sample size of 110 participants assigned to an investigational agent and a similar number concurrently assigned to placebo, we assume that about 100 participants in each group will have NP swabs available at a scheduled measurement time. Table 10.3.1-1 shows the power to detect a 20% absolute increase in percentage of participants with undetectable virus for a range of percentages with undetectable virus in the placebo group. The power was calculated for the comparison of two proportions using a normal approximation to the binomial distribution and unpooled variance, with two-sided Type I error rate of 5%. A power of over 82% is achieved regardless of the percentage of participants with undetectable virus in the control group. A sample size of 100 per group with NP swabs would also provide reasonable precision in estimating the absolute difference between groups in percentage with undetectable virus: for example, the width of a two-sided 95% confidence interval would be no more than ±13.6% around the observed difference, and the width of a two-sided 90% confidence interval would be no more than ±11.4%.

Table 10.3.1-1: Power to Detect a 20% Absolute Increase in % Undetectable for SARS-CoV-2 RNA for Various Percentages Undetectable in Control Group (calculated in PASS15 software)

| Control Group: Number with NP Swabs | Investigational Group: Number with NP Swabs | Percentage Undetectable in Investigational Group | Percentage Undetectable in Placebo Group | Power (%) |
|---|---|---|---|---|
| 100 | 100 | 30 | 10 | 95.5 |
| 100 | 100 | 40 | 20 | 88.5 |
| 100 | 100 | 50 | 30 | 83.9 |
| 100 | 100 | 60 | 40 | 82.3 |
| 100 | 100 | 70 | 50 | 83.9 |
| 100 | 100 | 80 | 60 | 88.5 |
| 100 | 100 | 90 | 70 | 95.5 |

The duration of symptoms from the start of investigational agent through 28 days of follow-up will be compared between an investigational agent and placebo control. A 20% relative reduction in median symptom duration is considered clinically relevant (e.g., from 10 days to 8 days, or from 15 days to 12 days). As there is very little data available about the distribution of symptom durations in outpatient populations, we show here that the proposed sample size is likely to have good power to detect a 20% reduction in median duration based on data about variability in symptom duration from two very small studies [6, 7]. However, if the variability is larger than that assumed based on these two studies, the power will be diminished. In addition, the discussion below ignores the fact that symptom durations in this study will be measured in integer days rather than as a continuous measurement.

To evaluate power and precision for this comparison, an estimate of the variability in durations is needed. However, there is a paucity of information about this in the outpatient population. We use data from a very small US study (n=12), in which the median duration of COVID-19 symptoms (from initial onset) was 14 days and the inter-quartile range (IQR) was 11 to 19.5 days [7]. For the purposes of calculating sample size, we assume that the relative variability of durations among participants will be the same for this study's symptom duration outcome measure as in this recent data (recognizing that this study is measuring duration from start of investigational agent rather than from symptom onset). To proceed with an assessment of power, we make the simplifying assumption that the $\log_{10}$-transformed symptom duration will be approximately normally distributed and use this normality assumption to infer a standard deviation based on the above IQR, specifically that the standard deviation equals

$$[\log_{10}(19.5) - \log_{10}(11)]/1.35 = 0.185.$$

Division by 1.35 in this expression arises because the IQR for a normal distribution has width 1.35 times its standard deviation. Using data from another small study undertaken in China (n=16) gives a similar estimate of the standard deviation (0.196) based on an IQR of 6.25 to 11.5 days [6]. For simplicity, we therefore assume a standard deviation of 0.2 for the $\log_{10}$ of symptom duration measured in days. As an example, if the median duration is 10 days, the IQR would then be 7.3 to 13.6 days, and we can reasonably expect that essentially all participants will have durations of less than 28 days (so there will be no censored durations).

Assuming that 100 of the 110 participants in each of the investigational agent and placebo control groups will provide study diary data, and continuing to assume a normal distribution for $\log_{10}$ durations with standard deviation of 0.2, then the phase II component of the study will have about 91% power to show a 20% relative reduction in median duration of symptoms from the start of investigational agent (e.g., 10 days to 8 days). This calculation is based on using a Wilcoxon rank sum test to compare groups using a two-sided significance level of 0.05. Note that a 20% relative reduction in symptom duration corresponds to a difference in $\log_{10}$ duration of 0.097, so approximately one-half of the assumed standard deviation of 0.2.

Note that the power will be reduced if the variability of durations is larger than what was observed in these two studies. Table 10.3.1-2 illustrates this for standard deviations of 0.25 and 0.3 compared with the value assumed of 0.2. Note that although a standard deviation of 0.3 seems much larger than the value of 0.2 suggested by the two small studies, the lower quartile of the distribution of durations differs by only one day (assuming a normal distribution for log durations) and the upper quartile differs by just over two days (Table 10.3.1-2).

Table 10.3.1-2: Power for Selected Standard Deviations for $\text{Log}_{10}$-transformed Symptom Durations (calculated in PASS15 software)

| Standard Deviation of $\text{Log}_{10}$-transformed Durations | Quartiles, Days (assuming normal distribution for log durations with median of 10 days) | Power to Detect 20% Relative Reduction in Median for Two-sided Type 1 Error Rate=0.05 |
|---|---|---|
| 0.2 | 7.3, 13.6 | 91% |
| 0.25 | 6.8, 14.7 | 76% |
| 0.3 | 6.3, 15.9 | 60% |

### 10.4.2 Phase III

The phase III aspect of the study is designed to evaluate the efficacy of an investigational agent to reduce the proportion of participants hospitalized or dying by 28 days after starting investigational agent in outpatient adults

diagnosed with COVID-19 compared to those receiving placebo. The primary analysis will focus on comparing the ratio of proportions because of the uncertainty in knowing what the hospitalization/death proportion will be.

For each investigational agent that graduates to phase III, a total of 1000 participants will be randomized to receive that agent and approximately 1000 participants will be concurrently randomized as the placebo control. This sample size includes enrollment during the phase II evaluation. With 2000 participants, the study has 88.7% power to detect a relative reduction of 33.3% in the proportion of participants hospitalized/dying between the study groups (investigational agent vs. placebo), using the following assumptions:

- proportion hospitalized/dying in the placebo group is 15%. Although there is very little information about hospitalization rates in the targeted population, this proportion is considered plausible for the United States based on evolving surveillance information from the Centers for Disease Control (CDC).
- three interim analyses and one final analysis, equally spaced, with stopping guideline for efficacy of an agent versus placebo determined using the Lan-DeMets spending function approach with an O'Brien and Fleming boundary. (The analysis of data at the end of phase II are considered to be additional interim analyses, but the impact of this on sample size is negligible as the O'Brien and Fleming boundary is extremely conservative at that information time.)
- non-binding stopping guideline for futility using a moderately aggressive Type II error spending function, specifically a Gamma (-2) spending function, implemented using the Lan-DeMets spending function approach. Further details about these stopping guidelines are in section 10.5.
- allowance for 5% of participants to be lost-to-follow-up prior to being hospitalized or dying.

## 10.5 Data and Safety Monitoring

### 10.5.1 Phase II Period

Monitoring of safety during the time an investigational agent is in phase II evaluation is described in section 7.5. This includes the possibility that an independent NIAID-appointed DSMB may be asked to undertake an unblinded review of adverse events.

As described in section 3.0, there will be an interim analysis of safety data and the activity of an investigational agent when 50% of participants have completed the day 14 evaluation. Overall, if activity data support graduation and there are no safety concerns, then the DSMB may recommend continued enrollment of participants once phase II enrollment is complete pending results from complete phase II follow-up. If activity data do not yet support graduation, then enrollment will be paused once phase II enrollment is complete. It is not generally intended

to stop the phase II period of evaluation early for futility.

The DSMB will also review results from complete phase II follow-up. If these results indicate that the graduation criteria have been met and there are no safety or resistance concerns, then the DSMB may recommend continuation of the study into the phase III period of evaluation.

### 10.5.2 Phase III Period

A NIAID-appointed DSMB will undertake reviews of interim data from the study to help ensure the safety of participants in the study, and to recommend changes to the study including termination or modification for safety reasons or if there is persuasive evidence of efficacy or lack of efficacy of an investigational agent versus placebo in preventing hospitalizations and deaths. The DSMB may also recommend termination or modification of the study if it appears futile on statistical or operational grounds to continue the study as designed. The operation of the DSMB is governed by the NIAID DSMB Charter.

At each interim review, the DSMB will review summaries of data by randomized treatment group for the primary outcome of hospitalization/death, the secondary outcome of death, losses to follow-up, and adverse events (including early discontinuation of investigational agent). By-stratum summaries will also be reviewed.

*Stopping Guideline for Efficacy and Timing of Interim Efficacy Analyses*
Unless otherwise recommended by the DSMB, it is intended that the DSMB review three interim analyses of safety and efficacy data for an investigational agent versus placebo after about 25%, 50%, and 75% of the expected maximal efficacy (hospitalization/death) information in the trial is obtained. As a stopping guideline for greater efficacy of an investigational agent compared with placebo, the O'Brien and Fleming boundary will be used. The stopping guideline will be implemented using the Lan-DeMets spending function approach to allow for the possibility of changes in the timing of interim analyses and/or additional (or fewer) interim analyses if recommended by the DSMB. Note that the analysis of data at the completion of the phase II evaluation will be considered as an additional interim analysis for the purpose of calculating Type I error spending, though the error spent at that analysis will be negligible because it occurs very early in information time (i.e., at about 10% of the expected information for the comparison of a given agent to placebo).

With regard to the timing of interim analyses, the expected maximal efficacy information is approximately proportional to the expected number of hospitalizations/deaths under the assumed design parameters, i.e., assuming a proportion hospitalized/dying of 15% in the placebo control group and a relative reduction of 33.3% giving a proportion hospitalized/dying for the investigational agent of 10%, and a sample size of 1000 in each group. This gives a total number of participants hospitalized/dying across the two groups combined of

250. However, there is uncertainty about what might be the proportion of participants who are hospitalized or die in the placebo control group and so, unless otherwise recommended by the DSMB, interim analyses will be undertaken at the following times:

1. The earlier of when approximately 500 participants from the two groups combined (including the phase II evaluation) have been followed for the primary outcome assessed at day 28, or when approximately 62 participants in the two groups combined have been hospitalized or have died (i.e., one-quarter of the expected number of 250 participants hospitalized/dying in phase III for the investigational agent);
2. The earlier of when approximately 1000 participants from the two groups combined have been followed for the primary outcome assessed at day 28, or when approximately 125 participants in the two groups combined have been hospitalized or have died; and
3. The earlier of when approximately 1500 participants from the two groups combined have been followed for the primary outcome assessed at day 28, or when approximately 187 participants in the two groups combined have been hospitalized or have died.

Formal details of the expected maximal information and calculation of information time will be provided in the Statistical Analysis Plan.

In considering possible modifications to the study or termination of the study for efficacy, the DSMB may consider interim results for the secondary outcome of death, or differences in the primary outcome within strata. For example, the DSMB might make recommendations based on a high level of evidence for a difference between randomized groups in the proportion dying. It is not intended, however, to terminate a group for efficacy based on virologic or symptom outcome measures. Also, for example, recognizing that there may be more hospitalizations/deaths in the higher risk stratum of the study or for participants enrolled closer to onset of symptoms, recommendations might be based on a high level of evidence for a difference in that stratum in the proportion hospitalized/dying or in the proportion dying. In these contexts, a “high level of evidence” might be based on application of the O’Brien and Fleming stopping guideline to the death outcome, or for high-risk participants or those treated closer to symptom onset. In these circumstances, consideration should also be given to the increased risk of a Type I error.

There is the possibility that differences between the treatment groups may be observed early in follow-up. However, the overall goal of the study is to prevent hospitalization and deaths regardless of the timing, and therefore the focus of the treatment group comparisons will be at day 28.

*Stopping Enrollment to an Investigational Agent Because of Lack of Effect*
If enrollment to the study is fast, there may be limited opportunity to stop enrollment to a specific investigational agent before the target of 1000

participants randomized to that agent is complete (because it will take time to achieve follow-up of participants and additional time to analyze and review results). However, if the rate of enrollment allows for potential discontinuation of randomization to a specific investigational agent, then the following provides non-binding guidance on how this might be approached:

- an agent may be discontinued for statistical futility based on evidence of lack of effect or very limited effect compared with placebo. For the purposes of evaluating this, a moderately aggressive Type II error spending function will be used, specifically the Gamma (-2) spending function implemented using the Lan-DeMets spending function approach [12].

Figure 10.5.2-1 illustrates the stopping guidelines for both efficacy and futility assuming four equally spaced analyses. The left panel shows the stopping guidelines in terms of critical values for a z-test statistic comparing an agent to placebo for the four analyses. The right panel shows the stopping guidelines in terms of observed differences in proportions for the scenario when the observed proportion in the placebo control arm is 0.15 (i.e. 15%). In both panels, greater negative values favor greater effects of an investigational agent versus placebo, and values in the blue area suggest stopping for efficacy whereas values in the pink area suggest stopping for futility. As an example, focusing on the right-hand panel, if the observed proportion for placebo was 0.15 (i.e. 15%) at the first interim analysis, an absolute difference in proportions of 0.019 or larger (i.e. favoring placebo by 1.9%) at the first interim analysis would suggest stopping for futility. At the second interim analysis, an absolute difference of -0.008 (i.e. -0.8%) or smaller (i.e. negative but closer to zero than 0.8%, or positive hence favoring placebo) would suggest stopping for futility.

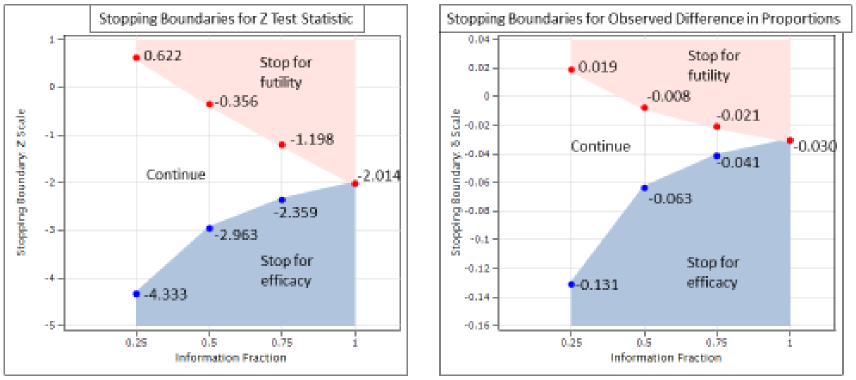


Figure 10.5.2-1: Stopping Boundaries for Efficacy and Futility

*Modifying or Stopping the Study for Operational Futility*
The DSMB will also monitor operational futility. With respect to operational futility, the DSMB may recommend modification or termination of the study if the proportion hospitalized/dying in the control group is much lower than expected in designing the trial. In particular, the DSMB might recommend restricting or closing enrollment to the low risk stratum in favor of increasing enrollment to the high-risk stratum. In addition, the DSMB will monitor the loss to follow-up (LTFU) rate. As a benchmark, an overall LTFU rate of more than 10% would be cause for concern.

## 10.6 Analyses

A Statistical Analysis Plan (SAP) will be developed that describes, in detail, the analyses to address the study's primary and secondary objectives in both phase II and phase III. The following provides an outline of the methods for the main comparisons between randomized groups, particularly for the primary outcome measures in each of phase II and phase III.

All analyses involving randomized comparisons will include all randomized participants who started an investigational agent or the concurrent placebo, according to a modified intention-to-treat approach. This should not introduce bias into the randomized comparison because of the use of a placebo. However, if evaluation of an investigational agent involves combining different placebos (i.e., because the study is partially blinded, with different placebos for different investigational agents), then consideration of the sensitivity of results to the possibility of different outcomes according to type of placebo taken will be considered; details will be provided in the SAP.

A general principle in all analyses is that outcomes among participants randomized to receive a specific investigational agent will be compared to outcomes among participants who were eligible to have been randomized (in the two-step randomization process) to the investigational agent but who were randomized instead to receive any of the placebos available at the time. This ensures that the comparison is restricted to concurrently randomized participants eligible to have taken the investigational agent of interest.

### 10.6.1 Primary Outcome Measures for Phase II

For evaluating the graduation criteria described in section 3.0, a Bayesian framework will be used to calculate the posterior probability that the difference in outcome for an investigational agent versus placebo exceeds the desired target. Initially non-informative prior distributions for relevant parameters will be used (see Appendix I). The choice of prior distributions may be updated as data accrue in the study; this will be described in an amendment to the protocol or in the appendix specific to an agent.

*Virologic Outcome: Undetectable SARS-CoV-2 RNA in NP Swabs*
Descriptive statistics will be used to describe the proportion of participants with undetectable RNA at each scheduled measurement time. Because of uncertainty about whether hospitalization might be driven by immunologic factors rather than virologic factors, the main analysis will not impute virologic outcome if results are not available because of hospitalization (though the sensitivity of this issue will be explored by considering an imputation of having detectable virus during hospitalization). For (frequentist) inference in presenting results, a repeated measures analysis will be undertaken across the scheduled measurement times using a binary regression model fitted using the generalized estimating equation approach with an independence working correlation structure, and two-sided 5% Type I error rate applied to a Wald-type test of the treatment by time interaction term (time included with indicator variables for each evaluation time).

*Clinical Outcome: Symptom Duration*
Symptom durations will be compared between study groups using a two-sided Wilcoxon test with a 5% Type I error rate, with descriptive summaries of the distribution of symptoms durations among participants.

*Safety and Tolerability: Grade 3 or Higher AE*
Safety and tolerability will be evaluated by estimating the proportion of participants with new Grade 3 or higher AE(s) by study day 28, and will be compared between groups using regression.

### 10.6.2 Primary Outcome Measures for Phase III

*Hospitalization/Death*
The cumulative proportion of participants hospitalized or dying during the first 28 days of follow-up will be estimated for each randomized group using Kaplan-Meier methods to take account of losses to follow-up. The difference between randomized groups in the estimated log cumulative proportion will be calculated and the variance for this difference will be obtained using Greenwood's formula. Two-sided 95% confidence intervals (adjusted for multiple interim analyses) and associated p-value for the test of no difference between groups will then be obtained.

Participants who prematurely discontinue the study, who are not able to be contacted by the site to ascertain outcomes after discontinuation, will have follow up censored at the date of last known status.

The above analysis assumes that losses to follow-up are non-informative. As a sensitivity analysis of this assumption, causal inference methods, specifically inverse probability of censoring, may be used.

*Safety and Tolerability: Grade 3 or Higher AE*
Safety and tolerability will be evaluated by estimating the proportion of

participants with new Grade 3 or higher AE(s) by study day 28, and will be compared between groups using binary regression.

#### 10.6.3 Secondary Outcomes

The cumulative proportion of participants dying during the first 28 days of follow-up, and through to 24 weeks, and the cumulative proportion hospitalized/dying through to 24 weeks will be analyzed in a similar manner to the phase III primary outcome.

Analysis of the proportion of participants with new Grade 2 or higher AE(s) by day 28 in phase II, and new Grade 3 or higher AE(s) by week 24 in phase III, and the proportion with progression of symptoms, will be undertaken using the same approach as for the primary safety analysis.

The AUC virologic outcome, COVID-19 severity ranking, duration of fever, and duration of time to self-reported return to usual health will be analyzed using similar methods as for the analysis of symptom durations.

Levels of SARS-CoV2 RNA on days 3, 7, 14, 21, and 28 will be compared between groups using non-parametric Wilcoxon rank-sum tests and descriptive statistics, separately at each scheduled measurement time (considering RNA results below assay limit as the lowest rank). A repeated measures analysis will also be undertaken using non-parametric methods.

In phase III, the large sample size will enable exploration of differences in symptom duration across strata defined by age, co-morbidities, and time from symptom onset to start of investigational agent using statistical methods for personalized/stratified medicine.

Descriptive summaries of clinical outcomes among those hospitalized will be provided by group, recognizing that this would not be a randomized comparison, if restricted to participants who were hospitalized.

### 10.7 Unblinding

Unblinding requests will follow PPD procedures.

In general, participants who become hospitalized at any time during the study period of 24 weeks can have their individual study treatment unblinded if essential for their future treatment management or if necessary for enrollment into a COVID-19 treatment clinical trial. This determination should be made by the Investigator of Record at the trial site and documented on the eCRF.

If treatment assignment is unblinded, this information should only be shared with the physicians responsible for the management of the participant on a need-to-know basis.

Treatment assignment should not be shared with others. This includes not sharing treatment assignment with the study team.

## 11.0 PHARMACOLOGY PLAN

The phase II pharmacology objective is to determine the pharmacokinetics of the investigational agent. For phases II and III, the pharmacology objective is to explore relationships between dose and concentration of investigational agent with virology, symptoms, and oxygenation. Samples for quantification of concentrations of the investigational agent will be obtained using a collection schedule appropriate for that agent and phase of evaluation, taking into consideration known pharmacokinetic characteristics (e.g., elimination half-life). Pharmacokinetic data analysis will use conventional and accepted approaches such as non-compartmental analysis, compartmental analysis, and population approaches. Usual parameters of interest are area under the concentration-time curve (AUC), total or apparent body clearance (CL), elimination half-life ($T_{1/2}$), and maximum and minimum concentrations ($C_{max}$, $C_{min}$). Exploration of relationships between dose and concentration of investigational agent with virology, symptoms, and oxygenation will be approached using conventional and accepted methods for pharmacokinetic/pharmacodynamic (PK/PD) data analyses. Such methods might include the $E_{max}$ or sigmoid $E_{max}$ model or structurally linked PK/PD models to explore exposure-response relationships. Exposure-response relationships will be performed in conjunction with the protocol statisticians.

See relevant appendix/appendices for details of the agent-specific pharmacology plan.

## 12.0 DATA COLLECTION AND MONITORING

### 12.1 Data Quality Assurance

This study will be conducted according to the ICH E6(R2) risk and quality processes described in the applicable procedural documents. The quality management approach to be implemented in this study will be documented and will comply with the current ICH guidance on quality and risk management. The sponsor assumes accountability for actions delegated to other individuals (e.g., Contract Research Organizations).

### 12.2 Records to Be Kept

Electronic case report form (eCRF) screens will be made available to sites for data entry. Participants must not be identified by name on any data submitted to the DMC. Participants will be identified by the subject number provided by the Clinical Data Management System (CDMS) upon enrollment.

## 12.3 Role of Data Management

As part of the responsibilities assumed by participating in the study, the investigator agrees to maintain adequate case histories for the participants treated as part of the research under this protocol. The investigator agrees to maintain accurate eCRFs and source documentation as part of the case histories.

All eCRF information is to be filled in. If an item is not available or is not applicable, this fact should be indicated. Blank spaces should not be present unless otherwise directed.

Investigative site personnel will enter participant data into CDMS. The analysis data sets will be a combination of these data and data from other sources (e.g., laboratory data).

Clinical data management will be performed in accordance with applicable DAIDS and PPD standards and data cleaning procedures to ensure the integrity of the data, for example, removing errors and inconsistencies in the data. Adverse event terms will be coded using MedDRA, an internal validated medical dictionary, and concomitant medications will be coded using WHODRUG.

## 12.4 Clinical Site Monitoring and Record Availability

12.4.1 Site monitors under contract to the NIAID will visit participating clinical research sites to review the individual participant records, including consent forms, eCRFs, supporting data, laboratory specimen records, and medical records (physicians’ progress notes, nurses’ notes, individuals’ hospital charts), to ensure protection of study participants, compliance with the protocol, and accuracy and completeness of records. The monitors also will inspect sites’ regulatory files to ensure that regulatory requirements are being followed and sites’ pharmacies to review product storage and management.

12.4.2 The site investigator will make study documents (e.g., consent forms, drug distribution forms, eCRFs) and pertinent hospital or clinic records readily available for inspection by the local IRB/IEC, the site monitors, the FDA, the NIAID, the ACTG, the OHRP, the industry supporter(s) or designee (as appropriate), other local, US, and international regulatory authorities/entities for confirmation of the study data.

# 13.0 PARTICIPANTS

## 13.1 Institutional Review Board (IRB) Review and Informed Consent

Federal regulations and the ICH guidelines require that approval be obtained from an IRB/IEC before human subjects participate in research studies. Before study onset, the protocol, informed consent, advertisements to be used for the recruitment of study participants, and any other written information regarding this study to be provided to the participant or the participant’s legal guardian must be approved by the IRB/IEC. Documentation of all IRB/IEC approvals and of the IRB/IEC compliance with ICH

harmonised tripartite guideline E6(R2): GCP will be maintained by the site and will be available for review by the sponsor or its designee.

All IRB/IEC approvals should be signed by the IRB/IEC chair or designee and must identify the IRB/IEC name and address, the clinical protocol by title or protocol number or both, and the date approval or a favorable opinion was granted.

The investigator is responsible for providing written summaries of the progress and status of the study at intervals not exceeding 1 year or otherwise specified by the IRB/IEC. The investigator must promptly supply the sponsor or its designee, the IRB/IEC, and, where applicable, the institution, with written reports on any changes significantly affecting the conduct of the study or increasing the risk to participants.

13.2 Ethical Conduct of Study

The study will be performed in accordance with the ethical principles that have their origin in the Declaration of Helsinki, ICH GCP, and all applicable regulations.

13.3 Participant Information and Consent

Informed consent in compliance with US Title 21 CFR Part 50 and US Title 45 CFR Part 46 shall be obtained from each participant before entering the study or performing any unusual or nonroutine procedure that involves risk to the participant. An informed consent template may be provided by the sponsor to investigative sites. If any institution-specific modifications to study-related procedures are proposed or made by the site, the consent should be reviewed by the sponsor or its designee or both before IRB/IEC submission. Once reviewed, the consent will be submitted by the investigator to his or her IRB/IEC for review and approval before the start of the study. If the ICF is revised during the course of the study, all active participants must be reconsented by signing the revised form.

Before recruitment and enrollment, each prospective participant or his or her legal guardian will be given a full explanation of the study, be allowed to read the approved ICF, and have any questions answered. Once the investigator is assured that the participant/legal guardian understands the implications of participating in the study, the participant/legal guardian will be asked to give consent to participate in the study. A witness may be used for the informed consent process if remote consent is performed and it is not possible to obtain a copy of the signed consent form from the participant (or legal guardian or person with power of attorney for participants who cannot consent for themselves).

13.4 Participant Confidentiality

All laboratory specimens, evaluation forms, reports, and other records that leave the site will be identified by coded number only to maintain participant confidentiality. All records will be kept locked. All computer entry and networking programs will be done with coded numbers only. Clinical information will not be released without written permission of the

participant, except as necessary for monitoring by the ACTG, IRB/EC, FDA, NIAID, OHRP, other local, US, and international regulatory authorities/entities as part of their duties, or the industry supporter(s) or designee.

## 13.5 Study Discontinuation

The study may be discontinued at any time by the ACTG, IRB/EC, FDA, NIAID, OHRP, other country-specific government agencies as part of their duties to ensure that research participants are protected (as appropriate), or the industry supporter(s).

# 14.0 PUBLICATION OF RESEARCH FINDINGS

Publication of the results of this trial will be governed by ACTG policies. Any presentation, abstract, or manuscript will be made available for review by the industry supporter(s) prior to submission.

# 15.0 BIOHAZARD CONTAINMENT

As the transmission of SARS-CoV-2 and other pathogens can occur through contact with contaminated needles, respiratory secretions, blood, and blood products, appropriate blood and secretion precautions will be employed by all personnel in the drawing of blood and shipping and handling of all specimens for this study, as currently recommended by the CDC and the National Institutes of Health.

All dangerous goods and materials, including diagnostic specimens and infectious substances, must be transported using packaging mandated by CFR 42 Part 72. Please refer to instructions detailed in the International Air Transport Association (IATA) Dangerous Goods Regulations.

## 16.0 REFERENCES

1. Tay MZ, Poh CM, Renia L, MacAry PA, Ng LFP. The trinity of COVID-19: immunity, inflammation and intervention. Nat Rev Immunol 2020;Apr 28:1-12. PMID: 32346093. PMC7187672. doi: 10.1038/s41577-020-0311-8.
2. COVID-19 Dashboard by the Center for Systems Science and Engineering (CSSE) at Johns Hopkins University (JHU) [Educational]; 2020 [updated 2020/05/03/]. Available from: http://coronavirusstatistics.org/.
3. ESRI StoryMaps Team. Mapping the Wuhan coronavirus outbreak [February 18, 2020]. Available from: https://storymaps.arcgis.com/stories/4fdc0d03d3a34aa485de1fb0d2650ee0.
4. Sheahan TP, Sims AC, Graham RL, et al. Broad-spectrum antiviral GS-5734 inhibits both epidemic and zoonotic coronaviruses. Sci Transl Med 2017;9(396): eaal3653. DOI: 10.1126/scitranslmed.aal3653.
5. Beigel JH, Tomashek KM, Dodd LE, et al.; ACTT-1 Study Group Members. Remdesivir for the treatment of Covid-19 -- preliminary report. N Engl J Med 2020;May 22. DOI: 10.1056/NEJMoa2007764.
6. Chang D, Mo G, Yuan X, et al. Time kinetics of viral clearance and resolution of symptoms in novel coronavirus infection. Am J Respir Crit Care Med 2020;201(9):1150-2. Epub 2020/03/24.
7. COVID-19 Investigation Team. Clinical and virologic characteristics of the first 12 patients with coronavirus disease 2019 (COVID-19) in the United States. Nat Med 2020;Apr 23. Epub 2020/04/25.
8. Hung IF, Lung KC, Tso EY, et al. Triple combination of interferon beta-1b, lopinavir-ritonavir, and ribavirin in the treatment of patients admitted to hospital with COVID-19: an open-label, randomised, phase 2 trial. Lancet 2020;395(10238):P1695-1704.
9. Coronavirus Disease 2019 (COVID-19) Treatment Guidelines [Government]; 2020. Available from: https://www.covid19treatmentguidelines.nih.gov/introduction/ https://covid19treatmentguidelines.nih.gov/introduction/.
10. The Adaptive Platform Trials Coalition. Adaptive platform trials: definition, design, conduct and reporting considerations. Nat Rev Drug Discov 2019;18:797-807. Epub 2019/08/30.
11. NIH to launch public-private partnership to speed COVID-19 vaccine and treatment options: NIH 2020 [updated 2020/04/16/T16:30:35-04:00; cited 2020 05/03]. Available from: https://www.nih.gov/news-events/news-releases/nih-launch-public-private-partnership-speed-covid-19-vaccine-treatment-options.
12. East 6.5 User Manual (Software Version 6.5). Cytel Inc., Waltham, MA, USA; 2018.

# APPENDIX I: ANALYTICAL FRAMEWORK FOR IMPLEMENTING THE GUIDELINES FOR GRADUATING AN INVESTIGATIONAL AGENT FROM PHASE II TO PHASE III

## Phase II to Phase III Graduation Rules

Graduation will be based on there being a desired level of evidence of an effect of an investigational agent versus placebo on one or more virologic and clinical outcome measures detailed below. The level of evidence required will be expressed in terms of Bayesian probability statements of the following form:

Probability (agent is better than placebo by at least X) is greater than 0.6

where X is defined below for each outcome measure. This probability defines a critical success factor (CSF) for an investigational agent. The choice of 0.6 for this probability indicates that there is a 3 to 2 odds of the agent being better than placebo than the agent being worse than placebo for that parameter. As there is considerable uncertainty about the association between phase II outcomes and the phase III outcome of hospitalization or death, graduation will be considered if this probability statement is met for any one of the outcome measures listed below (i.e., it does not need to be met for all outcome measures listed).

### *Primary Outcome Measures for Phase II*

The specific covariates used and any modifications to the models or CSFs below will be described in the appendix to the protocol specific to an investigational agent or in the associated Statistical Analysis Plan (SAP).

*Virologic Outcomes:*

(i) To assess difference in proportion of participants testing negative evaluated at each day NP swabs are taken will use the following Bayesian logistic regression model:

$$r_{ij}|p_{ij} \sim Bernoulli(p_{ij})$$

$$logit(p_{ij}) = \beta_0 + \delta x_{ij} + z_{ij}'\gamma$$

where i indexes participants in group *j* (*j*=1 denotes the placebo group and *j*=2 the treatment group), $x_{ij}$ =0 if *j*=1 and $x_{ij}$=1 if *j*=1, $p_{ij}$ is the probability of testing negative, $r_{ij}$ is 1 if the participant tested negative and zero otherwise, $\beta_0$ is the common intercept parameter, δ is the log odds ratio between the treatment group and control group, and $\gamma = (\gamma_1, \dots, \gamma_k)'$ is a vector of slope effects for *k* baseline covariates $z = (z_{ij1}, \dots, z_{ijk})'$ observed on participant *i* in group *j*. The following default independent non-informative priors are used: $\beta_0, \beta_1, \gamma_1, \dots \gamma_k \sim N(0,10^2)$

(ii) To assess the difference in median viral loads at each time point, the model with a Bayesian longitudinal mixed effect regression model:

$$y_{ijt}|\mu_{ijt} \sim Normal(\mu_{ij}, \sigma^2)$$
$$\mu_{ij} = \beta_0 + \beta x_{ij} + z_{ij}'\gamma + \psi_{ij}$$

where, i indexes participants in group j (j=1 denotes the placebo group and j=2 the treatment group) at time t, $y_{ij}$ is the $\log_{10}$ viral load, $\beta_0$ is the common intercept

parameter, $\delta$ is mean (and median) difference between the treatment group and control group, $\gamma = (\gamma_1, \ldots, \gamma_k)'$ is a vector of slope effects for k covariates $z = (z_{ij1}, \ldots, z_{ijk})'$, and $\psi_{ij}$ is a subject specific random effect. In analysis, values below the assay limit of quantification will be left censored observations. The following default independent noninformative priors are used

$$\beta_0, \beta_1, \gamma_1, \ldots \gamma_k \sim N(0,100^2)$$
$$\psi_{ij} \sim N(0, \tau^2)$$
$$\sigma^2 \sim IG(0.01,0.01)$$
$$\tau^2 \sim IG(0.01,0.01)$$

(iii) To assess AUC differences at each day NP swabs are taken the model is the same as in (ii) except $y_{ijt}$ is the AUC for participant *i* of group *j* at time *t*.

*Clinical Outcome: Symptom Duration*

To assess difference in median duration of symptoms, the following Bayesian Weibull regression model will be used:

$$y_{i,j} | \alpha, \beta_{ij} \sim Weibull(\alpha, \beta_{ij})$$
$$\log(\beta_{ij}) = \log(\beta_0) + \lambda x_{ij} + z'_{ij}\gamma$$

where i indexes participants in group j (j=1 denotes the placebo group and j=2 the treatment group), $y_{i,j}$ is the time of recovery or censoring, $\alpha$ is the common scale parameter, $\beta_0$ is the rate parameter for the control group and $\lambda$ is the log hazard ratio between the treatment group and control group, and $\gamma = (\gamma_1, \ldots, \gamma_k)'$ is a vector of slope effects for k baseline covariates $z = (z_{ij1}, \ldots, z_{ijk})'$. The following default independent noninformative priors are used

$$\alpha \sim HN(0,1)$$
$$\beta_0, \gamma_1, \ldots \gamma_k \sim Gamma(0.1,0.1)$$
$$\lambda \sim N(0,100^2)$$

If a reasonable proportion of participants are hospitalized or die, this model will be extended to include a mixture of distributions including a distribution for whether hospitalized/dead and the above Weibull distribution for symptom duration among participants who are alive and never hospitalized at day 28.

*Predictive Probability of Phase III Success*

To evaluate phase III probability of success based on phase II outcome, a joint model of the phase III primary endpoint ($\delta_t^H$: the log odds ratio for the hospitalization rate of agent t compared to placebo) and phase II outcomes will be developed. For example, one model could be using the binary virologic log odds ratio ($\delta_t^S$) and symptom duration log hazard ratio ($\lambda_t^S$) could be

$$\begin{pmatrix} \delta_t^V \\ \lambda_t^S \\ \delta_t^H \end{pmatrix} \sim N\left( \begin{pmatrix} \mu^V \\ \mu^S \\ \mu^H \end{pmatrix}, \begin{bmatrix} \sigma^{2V} & \rho^{VS}\sigma^V\sigma^S & \rho^{VH}\sigma^V\sigma^H \\ \rho^{VS}\sigma^V\sigma^S & \sigma^{2S} & \rho^{SH}\sigma^S\sigma^H \\ \rho^{VH}\sigma^V\sigma^H & \rho^{SH}\sigma^S\sigma^H & \sigma^{2H} \end{bmatrix} \right)$$

Where an appropriate prior for the $\mu, \rho,$ and $\sigma^2$ would be defined initially based on information outside of the trial. These parameters would be updated with all available information in the trial. The resulting predictive posterior of $\delta_t^H$would be used for evaluating probability of phase III success. Details will be included in a protocol amendment of the appendix or SAP for a given agent as the study evolves and data are accumulated to underpin such a modeling framework.

APPENDIX II: SAMPLE INFORMED CONSENT

DIVISION OF AIDS
AIDS CLINICAL TRIALS GROUP (ACTG) SAMPLE INFORMED CONSENT
FOR PROTOCOL: ACTIV-2 / A5401

Adaptive Platform Treatment Trial for Outpatients with COVID-19, FINAL Version 1.0

SHORT TITLE FOR THE STUDY: Adapt Out COVID

SUMMARY

| | |
|---|---|
| PURPOSE | This is a research study and your participation in this study is voluntary. The purpose of this study is to evaluate the ability of various drugs to improve health outcomes for people with COVID-19. We also want to see if these drugs are safe, and if these drugs can stop the disease process and prevent hospitalization. This study is designed to quickly identify safe and effective drugs that can treat COVID-19. |
| STUDY DRUG | Study drug will be either an active drug or a placebo. A placebo looks like a “real” drug, but it does not have any active medication in it.<br><br>As drugs are recommended for the treatment of COVID-19 symptoms, some of them will be selected for testing in this study. Therefore, there may be different drugs being used as part of the study at different times. You will receive information about specific drugs being tested at this time in a separate consent form.<br><br>If, during the course of the study, a standard treatment for COVID-19 is identified, that treatment will be substituted for placebo. |
| NUMBER OF PARTICIPANTS | For each drug being tested, a minimum of 110 people will receive that drug and an equal or smaller number will receive placebo. If a drug appears to be safe and effective when 110 people have received it, then more people will be enrolled so that 1000 receive that drug. Again, an equal or smaller number will receive placebo. |
| LENGTH OF STUDY | Your participation in this study will last for about 24 weeks (6 months). |
| REQUIRED ACTIVITIES | If you are in this study, the following study procedures are required:<br>• you will record your symptoms and temperature<br>• you will provide blood samples |

- you will provide self-collected nasal swab samples
- you may have nasopharyngeal swabs (i.e., deep nasal swabs) and saliva collected by a study staff person

RISKS

There are some risks that are specific to the study drug that you might receive. We will tell you about those risks in the second part of this consent process.

BENEFITS

If you take part in this study, there may be a direct benefit to you, but no guarantee can be made. It is also possible that you will receive no benefit from being in this study. Information learned from this study may help others who have COVID-19.

OTHER CHOICES

Instead of being in this study, you have the option of:

- treatment with prescription drugs available to you through your health care provider
- treatment with other experimental drugs, if you qualify
- no treatment

INTRODUCTION

You are being asked to take part in this research study because you have been diagnosed with SARS-CoV-2 and have symptoms of the disease it causes, which is commonly known as COVID-19. This study is sponsored by the National Institutes of Health (NIH). The doctor in charge of this study at this site is: (insert name of Principal Investigator). Before you decide if you want to be a part of this study, we want you to know about the study.

This is a consent form. It gives you information about this study. The study staff will talk with you about this information. You are free to ask questions about this study at any time. If you agree to take part in this study, you will be asked to sign this consent form. You will get a copy to keep.

WHY IS THIS STUDY BEING DONE?

SARS-CoV-2 is a new virus that has caused a widespread outbreak of an illness called COVID-19. In most people, it causes a mild to moderate symptoms, like a “cold”. In others, this virus can cause a pneumonia (an inflammation of the lungs), which can be serious and life threatening. There is no proven treatment for COVID-19 for people who are not sick enough to be hospitalized.

For each drug that is tested in this study, there could be two study parts. In the first part, we will see if the drug is safe. We will also see if it can decrease the how long people have COVID-19 symptoms and if it can help get rid of SARS-CoV-2 virus more than the placebo. Drugs that appear to be safe and to work better than the placebo in the first part of the study will be tested in the second part of the study.

In the second part of the study, we will continue to test how safe the drug is. We will also continue to compare it to a placebo to see if it can reduce the number of people who have to go into the hospital or who die from COVID-19.

You will be told which part of the study is open for enrollment during this consent process. At each stage, new study drugs may be added (in other words, multiple study drugs may be studied at one time).

The study is designed to rapidly evaluate new therapies for COVID-19. This could mean that the study finds that a drug that you were started on will not be studied further. If this happens, we will tell you. If you agree we would like you to continue to participate in the study and have all of the study visits, but this is your choice. We will not ask you to stay on the study drug if early results suggest that the study drug is not safe.

If you are randomized to an active drug in Phase II that is selected to continue to Phase III testing, you will not be notified of this decision.

WHAT DO I HAVE TO DO IF I AM IN THIS STUDY?

Information Collected at Screening
There is some information that we collect on everyone who is screened for this study. As part of your screening visit, some demographic (for example, age, gender, race), clinical (for example, disease condition, diagnosis), and laboratory values will be collected from you.

We will collect this information even if you do not enroll in this study. This information is collected so that researchers may determine whether there are patterns and/or common reasons why people do not join a study.

Blood Drawn
The site staff can tell you how much blood will be collected at any particular visit. At most visits, the amount will be no more than *XX mL (x tablespoons)* of blood collected. At a few visits, up to *XX-XX mL (x-x tablespoons)* will be collected.

Screening Visit
If you would like to be in this study, after you have read and signed this consent form, you will have a screening visit to make sure you meet the requirements for joining the study. This visit will take about 1 hour. You may come to the clinic, or if it is possible or necessary, this visit might be done remotely. At this visit:

- study staff will review your history and confirm that you have tested positive for SARS-CoV-2 infection.
- you will be asked about symptoms you are experiencing.
- study staff will ask you about any health conditions you have and questions about your health in general.
- study staff will ask you about your medication history and any medications you are taking.
- you may have a brief physical exam if your screening visit takes place at the clinic.

Entry Visit

If you qualify for the study, you will have an entry visit at the clinic. This visit might occur on the same day as your screening visit. At this visit, you will be randomly assigned (like flipping a coin or rolling dice) to a study group. You and the study staff will not be able to choose which treatment group you are in. You will not know whether you are receiving active drug or placebo. We will tell you more about the treatment groups that you might be in during the second part of this consent process.

Also at the Entry visit:

- you will have a physical exam and answer questions about your medical history and any medications you are taking or have taken in the past.
- you will be asked about symptoms you are experiencing.
- you will be asked about your smoking status and history.
- the study staff will ask if anyone else in your household has been diagnosed with SARS-CoV-2 infection.
- you will be asked to provide your home address.
- you will be asked to provide contact information for people the study staff could contact in case we cannot reach you for a study visit. You will need to tell these people that you are in the study, and that they could receive a call from study staff. If study staff cannot reach you after two tries (separated by 24 hours), they will call one of the people you have identified.
- you will be asked to provide your health care provider contact information, like your physician or commonly used clinic and hospital.
- you will receive a kit that includes information about the study, instructions and supplies for self-collection of certain samples, a thermometer, a diary in which you will record your temperature and how you are feeling, instructions on what to do if you have worsening symptoms, and contact information for the study staff.
- you will complete your first entry in the study diary with the study staff to make sure that you understand how to complete the diary.
- a swab will be collected from your nose. This swab is used to detect viruses. You will place a swab in each nostril and rotate the swab several times. Study staff will provide you with further instructions about the nose swabs.
- you will have blood drawn. This blood will be used for the following tests:
    - to find out the levels of SARS-CoV-2 virus, inflammation markers, and clotting factors in your blood
    - to measure zinc and vitamin D levels
    - for future protocol-required testing
- you will start study drug. Details of this are provided in the next part of the consent.

If you participate in the first part of the study:

- you will have a second swab collected from your nose. For this swab, the site staff will insert a different kind of swab into your nostril. The swab will be placed deep towards to the back of your throat. The swab will be left in place for several seconds and then slowly removed. This procedure is uncomfortable and it might make you gag or make your nose bleed.
- you will be asked to provide about 1 tablespoon of saliva. This saliva will be used to detect and measure SARS-CoV-2.

Study Visits
After the Entry visit, your study visits and evaluations will be different depending on whether you are in the first part of the study or the second part of the study.

IF YOU ARE IN THE FIRST PART OF THE STUDY:

Daily on Days 1-14
You will collect a nose swab every day on days 1-14. If you have a clinic visit scheduled, you will collect the nose swab at the clinic. If you do not have a clinic visit scheduled, you will collect the swab on your own and save it at home. You will be given instructions for how and when to return the swabs to the study staff.

You will record if you collected your nose swab in your study diary each evening.

Daily on Days 1-28
You will record your symptoms and your temperature in your study diary each evening. If you are not feeling well, someone can help you by writing the responses down for you, but the responses should come from you.

You will receive a reminder every day on days 1-28 to complete your study diary. This reminder may be by telephone, text message, email, or other method that you give permission for.

Day 2 Study Visit
On day 2, your study visit will be done over the phone or by video conference. At this visit:
- the study staff will ask questions about how you are feeling and what medications you are taking.
- the study staff will ask you if there are any updates to the contact information for the people who you have identified.
- you will review the entries in your study diary with study staff.

Study Visits on Days 3, 7, 10, 14, 21, 28
On these days you will have a study visit. You may come to the clinic, or if it is possible or necessary, these visits might be done somewhere else, possibly at your home. You and the staff may need to discuss where each of these visits will take place.

At these visits:
- you will have a brief physical exam and answer questions about any medications you are taking.
- the study staff will ask you if there are any updates to the contact information for the people you have identified.
- tou will review the entries in your study diary with study staff. On day 28, the study staff will collect your diary.
- the study staff may ask you if anyone else in your household has been diagnosed with SARS-CoV-2 infection.

- you may have blood drawn. This blood will be used for the following tests:
  - to find out the levels of SARS-CoV-2 virus, inflammation markers, and clotting factors in your blood
  - to measure zinc and vitamin D levels
  - for future protocol-required testing
- the site staff will collect a nasal swab as described above.
- you may be asked to provide about 1 tablespoon of saliva.

<u>Study Visits at Weeks 12 and 24</u>
On these days you will have a study visit. You may come to the clinic, or if it is possible or necessary, these visits might be done somewhere else, possibly at your home. You and the staff may need to discuss where each of these visits will take place.

At these visits:
- you will have a brief physical exam and answer questions about any medications you are taking.
- at week 12, the study staff will ask you if there are any updates to the contact information for the people you have identified.
- the study staff may ask you if anyone else in your household has been diagnosed with SARS-CoV-2 infection.
- you will have blood drawn. This blood will be used for the following tests:
  - to find out the levels of inflammation markers and clotting factors in your blood
  - for future protocol-required testing

<u>Genetic Testing</u> *[sites remove this section if PBMCs are not collected at your site]*
Your body, like all living things, is made up of cells. Cells contain deoxyribonucleic acid, also known as “DNA”. DNA is like a string of information put together in a certain order. Parts of the string make up “genes”. Genes contain instructions on how to make your body work and fight disease. Differences or changes in DNA explain some of the physical differences among people. These differences partly explain why some people get diseases like cancer or diabetes while others do not. Genetic testing looks at the differences in people’s DNA. This testing also looks at how differences affect health and the body’s response to disease and treatment.

If you agree, some of your blood that is collected will be used to study whether there are genetic differences in how sick people get when they are infected with SARS-CoV-2 or how they respond to study drugs. This genetic testing might include might include whole genome sequencing (WGS). “Sequencing” is looking at the order of a person’s genes to see how this order is different from the order of most people.

You do not have to agree to participate in this genetic testing. Even if you do not agree, you can still participate in the rest of the study.

Please put your initials below to indicate your choice:

________ (initials) I understand and I agree to this use of my samples

*OR*

________ (initials) I understand but I do not agree to this use of my samples

IF YOU ARE IN THE SECOND PART OF THE STUDY:

Days 3, 7, 10, 14, and 21
You will collect a nose swab on each of these days. You will collect the swabs on your own and save them at home. You will be given instructions for how and when to return the swabs to the study staff.

You will record if you collected your nose swab in your study diary each evening.

Daily on Days 1-28
You will record your symptoms and your temperature in your study diary each evening. If you are not feeling well, someone can help you by writing the responses down for you, but the responses should come from you.

You will receive a reminder every day on days 1-28 to complete your study diary. This reminder may be by telephone, text message, email, or other method that you give permission for.

Study Visits on Days 3, 7, 10, 14, and 21
On these days your study visit will be done over the phone or by video conference. At these visits:
- you will answer questions about how you are feeling and any medications you are taking.
- the study staff will ask you if there are any updates to the contact information for the people you have identified.
- you will review the entries in your study diary with study staff.

Study Visit on Day 28
On this day you will come to the clinic for a study visit. If it is possible or necessary, this visit might be done somewhere else, possibly at your home. You and the staff may need to discuss where this visit will take place.

At this visit:
- you will have a brief physical exam and answer questions about any medications you are taking.
- the study staff will ask you if there are any updates to the contact information for the people you have identified.
- you will review the entries in your study diary with study staff and the study staff will collect your diary.

- the study staff will ask you if anyone else in your household has been diagnosed with SARS-CoV-2 infection.
- you will collect a swab from your nose as described above.
- you will have blood drawn. This blood will be used for the following tests:
    - to find out how much SARS-CoV-2 virus, inflammation, and clotting factors are in your blood
    - to measure zinc and vitamin D levels
    - for future protocol-required testing

Study Visits at Weeks 12 and 24
On these days you will have a study visit. You may come to the clinic, or if it is possible or necessary, these visits might be done somewhere else, possibly at your home. You and the staff may need to discuss where each of these visits will take place.

At these visits:
- you will have a brief physical exam and answer questions about any medications you are taking.
- at week 12, the study staff will ask you if there are any updates to the contact information for the people you have identified.
- the study staff may ask you if anyone else in your household has been diagnosed with SARS-CoV-2 infection.
- you will have blood drawn. This blood will be used for the following tests:
    - for future protocol-required testing.

Genetic Testing *[sites remove this section if PBMCs are not collected at your site]*
Your body, like all living things, is made up of cells. Cells contain deoxyribonucleic acid, also known as “DNA”. DNA is like a string of information put together in a certain order. Parts of the string make up “genes”. Genes contain instructions on how to make your body work and fight disease. Differences or changes in DNA explain some of the physical differences among people. These differences partly explain why some people get diseases like cancer or diabetes while others do not. Genetic testing looks at the differences in people’s DNA. This testing also looks at how differences affect health and the body’s response to disease and treatment.

If you agree, some of your blood that is collected will be used to study whether there are genetic differences in how sick people get when they are infected with SARS-CoV-2 or how they respond to study drugs. This genetic testing might include might include whole genome sequencing (WGS). “Sequencing” is looking at the order of a person’s genes to see how this order is different from the order of most people.

You do not have to agree to participate in this genetic testing. Even if you do not agree, you can still participate in the rest of the study.

Please put your initials below to indicate your choice:

________ (initials) I understand and I agree to this use of my samples

*OR*
________ (initials) I understand but I do not agree to this use of my samples

Early Discontinuation
If at any point in the study you want to stop participating in the study, you must contact the site immediately. The study doctor may ask you to continue to be part of the study and return for some study visits and procedures.

If you have not withdrawn consent but must discontinue participation in the study after starting study drug, the site will attempt to obtain information regarding vital status (whether you are living or have died) from other sources, such as family members, other secondary contacts that you have provided, or clinical records.

## WILL I RECEIVE THE RESULTS OF ANY TESTS?

Some of the blood that is collected from you will be stored and tested later. Some of these tests will be done after you are done with the study, and other tests are not yet approved by the FDA and are still considered “research” tests. For these reasons, you will not receive the results of the tests to:

- check levels of SARS-CoV-2 in your blood, nasal swabs, and saliva
- check how well your blood clots
- check the level of inflammation markers and clotting factors in your blood
- check if your body developed antibodies to SARS-CoV-2

You will be told of any new information learned during the course of the study that might cause you to change your mind about staying in the study. At the end of the study, you will be told when study results may be available and how to learn about them. As with all studies, if we find out important information that may affect your care, you will be provided with those results.

## HOW MANY PEOPLE WILL TAKE PART IN STUDY?

In the first part of the study, 110 people will receive each study drug and a similar number of people will receive placebo. If the study proceeds to the second part for a particular study drug, up to 1000 participants will receive that study drug and a similar number will receive placebo.

## HOW LONG WILL I BE IN THIS STUDY?

You will be in this study for about 24 weeks (6 months).

WHY WOULD THE DOCTOR TAKE ME OFF THIS STUDY EARLY?

The study doctor may need to take you off the study early without your permission if:

- the study is stopped or cancelled.
- your health care provider requests that you stop participating in the study.
- you do not receive the first dose of study drug when you start the study.

The study doctor may also need to take you off the study drug without your permission if:

- you are taking other medications that should not be taken with the study drug.
- continuing the study drug may be harmful to you.

If you must stop taking the study drug before you are finished with the study, the study doctor will ask you to continue to be part of the study and return for study visits and procedures.

WHAT HAPPENS IF I DECIDE TO PERMANENTLY STOP TAKING STUDY-PROVIDED MEDICATIONS?

If you must permanently stop taking study drug before your study participation is over, the study staff will discuss other options that may be of benefit to you.

WHAT HAPPENS WHEN I FINISH THE STUDY?

After you have completed your study participation, the study will not be able to continue to provide you with the study drug you received on the study. If continuing to take these or similar drugs/agents would be of benefit to you, the study staff will discuss how you may be able to obtain them.

WHAT ARE THE RISKS OF THE STUDY?

<u>Risks of Study Drug</u>
There are risks to taking part in any research study. The effectiveness of the study drug is not known. One risk is that the study drug may not stop you from becoming sicker, being hospitalized, or dying from SARS-CoV-2.

There is a risk of serious and/or life-threatening side effects when non-study medications are taken with the study drug. For your safety, you must tell the study doctor or nurse about all medications you are taking before you start the study.

There are some risks that are specific to the study drug that you might be assigned to. We will tell you about those risks in the second part of this consent process.

Risks of Blood Draw
Having blood drawn may cause some discomfort, bleeding, bruising, and/or swelling where the needle enters the body, and in rare cases it may result in fainting. There is a small risk of infection.

Risks of Nose Swabs
Nose swabs might make you gag or sneeze. They may also cause discomfort or cause your nose to bleed.

ARE THERE RISKS RELATED TO PREGNANCY AND BREASTFEEDING?

In the second part of the consent process we will tell you about the specific drugs that you might receive and whether they have any risks related to pregnancy and breastfeeding.

If you become pregnant while on study, the study staff would like to obtain information from you about the outcome of the pregnancy (even if it is after your participation in the study ends).

ARE THERE BENEFITS TO TAKING PART IN THIS STUDY?

If you take part in this study, there may be a direct benefit to you, but no guarantee can be made. It is also possible that you may receive no benefit from being in this study. Information learned from this study may help others who have COVID-19.

WHAT OTHER CHOICES DO I HAVE BESIDES THIS STUDY?

Instead of being in this study you have the choice of:

- treatment with prescription drugs available to you from your health care provider.
- treatment with other experimental drugs, if you qualify.
- no treatment.

Please talk to your doctor about these and other choices available to you. Your doctor will explain the risks and benefits of these choices.

WHAT ABOUT CONFIDENTIALITY?

For sites in the US
We will do everything we can to protect your privacy. In addition to the efforts of the study staff to help keep your personal information private, we have gotten a Certificate of Confidentiality from the US Federal Government. This certificate means that researchers cannot be forced to tell people who are not connected with this study, such as the court system, about your participation. Any publication of this study will not use your name or identify you personally.

Your records may be reviewed by the US Food and Drug Administration (FDA), the ACTG, the US Office for Human Research Protections (OHRP), or other local, US, and international

regulatory entities as part of their duties, (insert name of site) institutional review board (IRB) (a committee that protects the rights and safety of participants in research), National Institutes of Health (NIH), study staff, study monitors, drug companies supporting this study, and their designees. Having a Certificate of Confidentiality does not prevent you from releasing information about yourself and your participation in the study.

Even with the Certificate of Confidentiality, if the study staff learns of possible child abuse and/or neglect or a risk of harm to yourself or others, we will be required to tell the proper authorities.

A description of this clinical trial will be available on ClinicalTrials.gov, as required by US law. This website will not include information that can identify you. At most, the website will include a summary of the results. You can search this website at any time.

For sites outside the US

Efforts will be made to keep your personal information confidential. We cannot guarantee absolute confidentiality. Your personal information may be disclosed if required by law. Any publication of this study will not use your name or identify you personally.

Your records may be reviewed by the US Food and Drug Administration (FDA), the ACTG, the US Office for Human Research Protections (OHRP), or other local, US, and international regulatory entities as part of their duties (insert name of site) institutional review board (IRB) or Ethics Committee (a committee that protects the rights and safety of participants in research), National Institutes of Health (NIH), study staff, study monitors, drug companies supporting this study, and their designees.

A description of this clinical trial will be available on ClinicalTrials.gov, as required by US law. This website will not include information that can identify you. At most, the website will include a summary of the results. You can search this website at any time.

WHAT IF THE SITE CAN NO LONGER REACH ME DURING THE STUDY?

If you cannot be reached after two attempts to contact you (with 24 hours between attempts), study staff may try to contact you through the family, friends, or acquaintances you provided at screening and updated at each visit.

If you are still unable to be reached, we will attempt to obtain information about your status (whether you are living or have died) by contacting your health care provider (if you agree) or by accessing publicly available records (you do not have to give your permission for us to access these records).

WHAT ARE THE COSTS TO ME?

There will be no cost to you for study-related visits or procedures. If you require medical care as a result of taking study drug, it is possible that your insurance company will not pay for these

costs because you are taking part in a research study. Costs related to acute care/hospitalization will not be covered by the study.

WILL I RECEIVE ANY PAYMENT?

*[Insert site-specific information on compensation to study participants.]*

WHAT HAPPENS IF I AM INJURED?

If you are injured as a result of being in this study, you will be given immediate treatment for your injuries.

[Sites: Please modify (if necessary) and insert one of these two statements, as appropriate to your site. If your site is required to carry CTI, this must be indicated in the informed consent.

- this site has clinical trials insurance. This insurance will allow the site to provide you with monetary compensation if you suffer harm as a result of participating in this research study. OR
- the cost for this treatment will be charged to you or your insurance company. There is no program for compensation either through this institution or the NIH.]

You will not be giving up any of your legal rights by signing this consent form.

WHAT ARE MY RIGHTS AS A RESEARCH PARTICIPANT?

Taking part in this study is completely voluntary. You may choose not to take part in this study or leave this study at any time. Your decision will not have any impact on your participation in other studies and will not result in any penalty or loss of benefits to which you are otherwise entitled.

We will tell you about new information from this or other studies that may affect your health, welfare, or willingness to stay in this study. If you want the results of the study, let the study staff know.

WHAT DO I DO IF I HAVE QUESTIONS OR PROBLEMS?

For questions about this study or a research-related injury, contact:

- name of the investigator or other study staff
- telephone number of above

For questions about your rights as a research participant, contact:

- name or title of person on the Institutional Review Board (IRB) or other organization appropriate for the site
- telephone number of above

Contacting Your Health Care Provider
*[Sites modify per local requirements for obtaining health care records*.]
With your permission, for which you would need to sign a waiver, study staff may contact your health care provider or hospital(s) where you might receive care to determine if you have been hospitalized or died while in the study, and the cause of death. You can still participate in this study even if you do not give us permission to contact your health care provider or hospital(s).

Will you allow us to contact your health care provider or hospital(s) to obtain this information?

________ YES

________ NO

________ Initials

If you said Yes, please list the names of your health care provider and the hospitals you would likely be admitted to, below:

______________________________________________________________________________

______________________________________________________________________________

______________________________________________________________________________

______________________________________________________________________________

SIGNATURE PAGE

If you have read this consent form (or had it explained to you), all your questions have been answered and you agree to take part in this study, please sign your name below.

| | |
|---|---|
| Participant's Name (print) | Participant's Signature and Date |
| Participant's Legally Authorized Representative (As appropriate) | Legally Authorized Representative (print) Signature and Date |
| Study Staff Conducting Discussion (print) | Study Staff's Signature and Date Consent |
| Witness's Name (print) | Witness's Signature and Date (As appropriate) |

ATTACHMENT A: CONSENT FOR USE OF EXTRA SAMPLES

When samples are no longer needed for this study, the ACTG may want to use them in other studies and share them with other researchers. These samples are called "extra samples." The ACTG will only allow your extra samples to be used in other studies if you agree to this. If you have any questions, please ask.

Identifiers will be removed from your samples and from any private information that has been collected about you. This means that no one looking at the labels or at other information will be able to know that the samples or information came from you.

Extra samples are stored in a secure central place called a repository. Your samples will be stored in the ACTG repository located in the United States.

There is no limit on how long your extra samples will be stored. *[Site: Revise the previous sentence to insert limits if your regulatory authority imposes them.]*

When a researcher wants to use your samples and information, the research plan must be approved by the ACTG. Also, the researcher's institutional review board (IRB) or ethics committee (EC) will review the plan. *[Site: If review by your institution's IRB/EC/RE is also required, insert a sentence stating this.]* IRBs/ECs protect the rights and well-being of people in research. If the research plan is approved, the ACTG will send your samples to the researcher's location. This means that researchers who are not part of the protocol team may use your samples without asking you again for your consent.

You will not be paid for your samples. Also, a researcher may make a new scientific discovery or product based on the use of your samples. If this happens, there is no plan to share any money with you.

You may withdraw your consent for research on your extra samples at any time and the specimens will be discarded.

Please choose the response that matches what you want by putting your initials in the space provided. Please ask the staff any questions that you have before you indicate your selection.

<u>Research without Human Genetic Testing</u>
If you agree, your extra samples may be stored (with usual protection of your identity) and used for ACTG-approved research that does not include human genetic testing.

________ (initials) I understand and I agree to this storage and possible use of my samples.

*OR*
________ (initials) I understand but I do not agree to this storage and possible use of my samples.

# APPENDIX III: INVESTIGATIONAL AGENT LY3819253

Information/evaluations noted in this agent-specific appendix are IN ADDITION to those presented in the master protocol. Section numbering aligns with the master protocol.

## 2.0 INTRODUCTION

## 2.2 Rationale

Monoclonal Antibodies (mAbs)

Sera obtained from persons or animals who recovered from a particular infection has shown prophylactic and therapeutic potential for a variety of infections, and Emil von Behring won the Nobel Prize in 1893 for his work on use of immune serum from the blood of infected animals to provide immunity to diphtheria [1]. Currently, hyperimmune human sera immunoglobulin is still used to treat many viral infections including cytomegalovirus (CMV), respiratory syncytial virus (RSV), hepatitis A virus (HAV), hepatitis B virus (HBV), and rabies [2].

Unfortunately, heterologous sera was associated with a variety of complications including serum sickness and hypersensitivity, which significantly limited its usefulness clinically [3]. Given the long history of use of antibodies for infectious diseases, monoclonal antibodies were developed (mAbs). Improved purification techniques and the ability to engineer humanized mAbs allowed for the development of broadly reactive and potent mAbs, which helped reduce some of the issues that hampered the utility of heterologous sera [3, 4]. In fact, current technology allows mAbs to be produced requiring only tissue culture or microbial expression systems, thus the potential toxicity of humanized mAbs is comparable to antibiotics [2, 4].

Engineered humanized mAbs have shown considerable efficacy for viral infections. The first was Palivizumab in 1998, which is used for RSV [5]. Monoclonal antibodies have also been quickly developed for emerging infections such as Ebola [6]. As a part of the massive scientific effort to stop COVID-19, mAbs have been developed for treatment of COVID-19. These agents now need to be evaluated in rigorous randomized clinical trials.

The limitations of mAbs continue to be cost and that these antibodies are perishable, require refrigeration, and must be administered parenterally [4]; however, their use may still be useful in the outpatient setting, as one dose often stays in the therapeutic range for months [5], potentially allowing an entire treatment course with a single administration.

A number of viral infectious diseases have been successfully treated with mAbs, including RSV and HIV. Some of these mAbs were derived from persons who were infected with these viruses and mounted neutralizing humoral responses. The first investigational agent to be evaluated in this trial will be the mAb LY3819253 made by

Lilly Research Laboratories, Eli Lilly and Company, in partnership with AbCellera Biologics. LY3819253 was derived from a person who was infected with and recovered from SARS-CoV-2.

Investigational Agent
LY3819253 is a neutralizing immunoglobulin G (IgG)-1 mAb directed to the spike (S) protein of SARS-CoV-2. It was developed as a potential treatment for COVID-19. This mAb blocks S protein attachment to human angiotensin-converting enzyme 2 (ACE2) receptors, thus preventing viral entry into human cells and its subsequent viral replication. This treatment is expected to result in a clinically important decrease of viral replication, mitigating the severity of COVID-19 in persons with the infection in whom ongoing viral replication is the primary driver of pathophysiology. The potential reduction in viral replication may also decrease a treated person's extent and duration of viral shedding and transmission, thus potentially positively impacting public health.

The first in-human clinical studies of LY3819253 started on May 28, 2020 (NCT04411628) [7].

Nonclinical single-dose studies of IV administered LY3819253 have been performed in rats and cynomolgus monkeys. In rats, the mean elimination half-life was 277 hours (11.5 days); in cynomolgus monkeys, the mean elimination half-life was 315 hours (13.1 days). These data informed predictions of clinical pharmacokinetic characteristics of a total body clearance of 0.26 L/day and an elimination half-life of 19 days. Preliminary data available from a limited number of subjects who received a single dose of 700 mg indicate pharmacokinetic behavior consistent with predictions. [LY3819253 Investigator's Brochure, June 26, 2020].

## 4.0 SELECTION AND ENROLLMENT OF PARTICIPANTS

Participants must meet inclusion and exclusion criteria from the master protocol, as well as the appropriate inclusion and exclusion criteria for the investigational agent included below.

## 4.1 General Eligibility Criteria

### 4.1.1 Inclusion Criteria

4.1.1.8 For participants who are of reproductive potential, negative serum or urine pregnancy test at within 48 hours prior to study entry by any clinic or laboratory that has a CLIA certification or its equivalent, or by a point of care (POC)/CLIA-waived test.

NOTE: Reproductive potential is defined as:
- participants who have reached menarche
- participants who have not been post-menopausal for at least 12 consecutive months with follicle-stimulating hormone (FSH) ≥40 IU/mL or 24 consecutive months if an FSH is not available

- participants who have not undergone surgical sterilization (e.g., hysterectomy, bilateral oophorectomy, bilateral tubal ligation, or bilateral salpingectomy)
- participants with no other clinical conditions (such as anorexia nervosa) that could induce amenorrhea
- participants not taking medications such as oral contraceptives, hormones, gonadotropin-releasing hormone, anti-estrogens, selective estrogen receptor modulators (SERMs) or chemotherapy that could induce amenorrhea
- For individuals with permanent infertility due to an alternate medical cause (e.g., Mullerian agenesis, androgen insensitivity), investigator discretion should be applied to determining study entry.

4.1.1.9 If participating in sexual activity that could lead to pregnancy, participants who are of reproductive potential must agree to use two forms of effective contraception, where at least one form is highly effective (less than 1% failure rate), for the entirety of the study and for 90 days after investigational agent is administered.

Highly effective methods of contraception (less than 1% failure rate) include, but are not limited to:

- combination oral contraceptives
- implanted contraceptives
- intrauterine devices

Effective methods of contraception include, but are not limited to

- diaphragms and cervical caps with spermicide
- cervical sponges
- condoms with spermicide

NOTE: Participants not of reproductive potential are eligible without requiring the use of a contraceptive method. Participant-reported history is acceptable documentation of surgical sterilization and menopause.

NOTE:

- Use of male and female condoms as a double barrier method is not considered acceptable due to the high failure rate when these barrier methods are combined.
- Barrier protection methods without concomitant use of a spermicide are not an effective or acceptable method of contraception.
- Periodic abstinence (e.g., calendar, ovulation, symptothermal, post-ovulation methods), declaration of abstinence just for the duration of a trial, and withdrawal are not acceptable methods of contraception.

4.1.1.10 Participants that engage in sexual activity that may lead to pregnancy in their partner must agree to either remain abstinent or use male or

female condoms with spermicide as well as one additional form of effective contraception with non-pregnant sexual partners of reproductive potential, for the entirety of the study and for 90 days after investigational agent is administered.

Additional forms of effective contraception that may be used by the partner include:

- hormone-based contraception (oral, patch, parenteral, implants, or vaginal ring)
- intrauterine device (IUD)
- diaphragms and cervical caps with spermicide
- cervical sponge

Participants with pregnant partners should use condoms during vaginal intercourse through 90 days after investigational agent administration.

Participants should refrain from sperm donation through 90 days after investigational agent administration.

4.1.2 Exclusion Criterion

4.1.2.11 Currently pregnant

4.1.2.12 Currently breastfeeding

## 5.0 INVESTIGATIONAL AGENT

## 5.1 Regimen, Administration, and Duration

Participants will be randomized to receive one of the following two regimens:

Investigational Agent: LY3819253, 7000 mg, to be administered intravenously (IV) over approximately 60 minutes for one dose at study Entry/Day 0

*OR*

Placebo for LY3819253: 0.9% Sodium Chloride for Injection, USP, to be administered IV over approximately 60 minutes for one dose at study Entry/Day 0

Participants will be monitored for signs and symptoms of infusion reaction per section 6.3.8 and the infusion rate may be slowed, paused, or stopped, lengthening the duration of infusion as deemed necessary if an infusion reaction is observed (sections 8.2.2 and 8.2.3).

## 5.2 Formulation, Storage, and Preparation

### 5.2.1 Formulation and Storage

LY3819253 is supplied as a 700 mg/20 mL (35 mg/mL) aqueous solution in 20 mL glass vials. The aqueous solution concentrate is a clear to slightly opalescent and colorless to slightly yellow to slightly brown solution. LY3819253 vials must be stored between 2° to 8°C (refrigerated storage) until use. LY3819253 is described in further detail in the LY3819253 Investigator's Brochure.

Placebo for LY3819253 will be 0.9% Sodium Chloride for Injection, USP. The product must be locally sourced and stored according to the manufacturer's recommendation.

### 5.2.2 Preparation

Pharmacists must follow appropriate aseptic technique and sterile preparation procedures/guidance as outlined in USP <797>, utilizing a pharmacy biosafety cabinet/isolator. Local regulations and site institutional policies and procedures for use of personal protective equipment, such as gloves, gowns, face masks and safety glasses, must be followed. Pharmacists should follow the requirements of their country, institution, and pharmacy regulatory authority regarding these procedures.

Any unused portion of investigational agent must not be used for another participant. Any empty vials, unused portion of entered vials, or unused solution which contains investigational agent should be discarded in a biohazard containment bag and incinerated or autoclaved in accordance with institutional or pharmacy policy.

#### 5.2.2.1 LY3819253

1. Remove ten (10) vials of LY3819253 from the refrigerator and an empty, sterile IV bag of appropriate size to contain 200 mL volume of LY3819253. Equilibrate the LY3819253 vials to room temperature for approximately 20 minutes.
2. Gently invert the vials by hand approximately 10 times to ensure homogeneity of the contents. Do not shake or vigorously agitate the vials. Visually inspect the vials for the presence of any visible particulate matter. If visible particulate matter is observed, appropriately discard the vials, obtain new vials, and restart the preparation.
3. Using appropriately sized syringes fitted with 21-gauge needles, withdraw 200 mL of LY3819253 solution from ten (10) vials. When the stopper of the first vial is punctured to start preparation, record this time as the investigational agent preparation time. Assign a 4-hour beyond use date and time from the preparation time.

4. Inject the contents of each syringe prepared in Step 3 into an empty, sterile IV bag of appropriate size to contain 200 mL volume of LY3819253.
5. Gently invert the prepared IV bag by hand approximately ten times to ensure homogeneity of the contents. Do not shake or vigorously agitate the prepared bag. Avoid foaming. Visually inspect the bag after preparation. The contents of the bag should be free of any visible particulate matter. Obtain new vials and re-prepare the dose if visible particulate matter is observed.
6. Attach an infusion set containing a 0.2 or 0.22 micron polyethersulfone (PES) in-line filter to the IV bag and prime the infusion set with the prepared investigational agent. Encase the IV bag and the primed infusion set in an opaque cover.

Prepared investigational agent in an IV bag should be administered immediately. If immediate administration is not possible, the investigational agent should be stored at room temperature and administered within 4 hours of preparation (refer to the assigned beyond use time in Step 3 above).

5.2.2.2 Placebo for LY3819253

1. Remove one 250 mL IV bag of 0.9% Sodium Chloride for Injection, USP from storage and one empty, sterile IV bag of appropriate size to contain 200 mL volume of 0.9% Sodium Chloride for Injection, USP.
2. Using appropriately sized syringes, withdraw 200 mL of 0.9% Sodium Chloride for Injection, USP from the 250 mL IV bag and inject into the empty, sterile IV bag. When the IV bag of 0.9% Sodium Chloride for Injection, USP is first punctured to start preparation, record this time as the placebo preparation time. Assign a 4-hour beyond use date and time from the preparation time.
3. Visually inspect the bag after preparation. The contents of the bag should be free of any visible particulate matter. Obtain a new IV bag of 0.9% Sodium Chloride for Injection, USP and re-prepare the dose if visible particulate matter is observed.
4. Attach an infusion set containing a 0.2 or 0.22 micron polyethersulfone (PES) in-line filter to the IV bag and prime the infusion set with the prepared placebo. Encase the IV bag and the primed infusion set in an opaque cover.

Prepared placebo in an IV bag should be administered immediately. If immediate administration is not possible, the placebo should be stored at room temperature and administered within 4 hours of preparation (refer to the assigned beyond use time in Step 2 above).

#### 5.2.2.3 Labeling of Investigational Agent and Placebo

Label the prepared IV bag with the following information:

a. Participant identifier(s)
b. Protocol number: ACTIV-2/A5401
c. Investigational agent name: LY3819253 7000 mg or Placebo
d. Total volume: 200 mL
e. Route: IV
f. Infusion rate/time: 200 mL/hour over 60 minutes
g. Preparation date and time
h. Beyond use date and time: 4 hours after preparation
i. Any additional information required by jurisdiction

## 5.3 Supply, Distribution, and Accountability

### 5.3.1 Supply/Distribution

LY3819253 will be provided by Eli Lilly and Company and will be available through the NIAID Clinical Research Products Management Center (CRPMC).

0.9% Sodium Chloride for Injection, USP, infusion sets, and any other ancillary supplies will be locally sourced by the site.

The site pharmacist should obtain the investigational agent(s) for this protocol by following the instructions in the manual *Pharmacy Guidelines and Instructions for DAIDS Clinical Trials Networks.*

### 5.3.2 Accountability

The site pharmacist is required to maintain complete records of all investigational agents received from the NIAID CRPMC and subsequently dispensed. At US CRSs, all unused investigational agents must be returned to the NIAID CRPMC (or as otherwise directed by the sponsor) after the study is completed or terminated. The procedures to be followed are provided in the manual *Pharmacy Guidelines and Instructions for DAIDS Clinical Trials Networks.* At non-US CRSs, the site pharmacist must follow the instructions in the *Pharmacy Guidelines and Instructions for DAIDS Clinical Trials Networks* for the destruction of unused investigational agents.

## 5.4 Concomitant Medications

Any pre-medications given will be documented as a concomitant medication.

## 6.0 CLINICAL AND LABORATORY EVALUATIONS

### 6.1 Schedule of Evaluations

Table 6.1-1: Schedule of Evaluations Phase II

| Phase II Evaluation | Screening | Entry / Day 0 | Day 2 | Day 3 | Day 7 | Day 14 | Day 21 | Day 28 | Week 12 | Week 24 | Hypersensitivity Reaction (Three Sample Time Points) | Premature Study D/C (Before Day 28) | Premature Study D/C (After Day 28) |
|---|---|---|---|---|---|---|---|---|---|---|---|---|---|
| Visit Window | | | +/-1 day | | +/-2 days | | +4 days | | +/-7 days | | | | |
| P = In Person Visit<br>R = Remote Visit | P/R | P | R | P | P | P | P | P | P | P | P | P | P |
| Investigational Agent Administered | | X | | | | | | | | | | | |
| Hematology | | X | | X | | X | | X | | | X | X | |
| Chemistry | | X | | X | | X | | X | | | X | X | |
| Pregnancy Testing | X | Whenever pregnancy suspected | | | | | | | | X | | | |
| PK Studies | | X | | | | X | | X | X | X | | X | X |
| Antidrug Antibodies | | X | | | | X | | X | X | X | | X | X |
| Blood Collected for Evaluation of Hypersensitivity Reaction | | | | | | | | | | | X | | |
| Urine Collected for Evaluation of Hypersensitivity Reaction | | | | | | | | | | | X | | |

Table 6.1-2: Schedule of Evaluations Phase III

| Phase III Evaluation | Screening | Study Entry/Day 0 | Day 2 | Day 3 | Day 7 | Day 10 | Day 14 | Day 21 | Day 28 | Week 12 | Week 24 | Hypersensitivity Reaction (Three Sample Time Points) | Premature Study D/C (Before Day 28) | Premature Study D/C (After Day 28) |
|---|---|---|---|---|---|---|---|---|---|---|---|---|---|---|
| Visit Window | | | +/-1 day | | +/-2 days | | | +4 days | | +/-7 days | | | | |
| P = In Person Visit<br>R = Remote Visit | P/R | P | R | R | R | R | R | R | P | P | P | | P | P |
| Investigational Agent Administered | | X | | | | | | | | | | | | |
| Hematology | | X | | | | | | | X | | | X | X | |
| Chemistry | | X | | | | | | | X | | | X | X | |
| Pregnancy Testing | X | | Whenever pregnancy suspected | | | | | | | | X | | | |
| PK Studies | | X | | | | | | | X | X | X | | X | X |
| Antidrug Antibodies | | X | | | | | | | X | X | X | | X | X |
| Blood Collected for Evaluation of Hypersensitivity Reaction | | | | | | | | | | | | X | | |
| Urine Collected for Evaluation of Hypersensitivity Reaction | | | | | | | | | | | | X | | |

## 6.2 Timing of Evaluations

### 6.2.1 Screening Evaluations

Screening evaluations must occur prior to the participant starting any study medications, treatments, or interventions.

Screening and study entry visit evaluations may be combined.

### 6.2.4 Event Driven Evaluations

Hypersensitivity Reactions (see section 8.2.3)
In the case of generalized urticaria or anaphylaxis occurring at any time following investigational agent administration through day 28, additional blood and urine samples should be collected at the following time points following instructions in the LPC:

- collect initial sample after the participant has been stabilized, and within 1 to 2 hours of the event; however, samples may be obtained as late as 12 hours after the event as analytes can remain altered for an extended period of time. Record the time at which the sample was collected
- obtain a follow-up sample at the next regularly scheduled visit or after approximately 4 weeks, whichever is later
- obtain a third follow-up sample at 12 weeks after the event

## 6.3 Instructions for Evaluations

### 6.3.9 Investigational Agent Administered

Pre-Medication
Pre-medication for infusions is not planned. However, if an infusion reaction occurs during administration or if the participant has a medical history suggesting a potential benefit from pre-medication, the study investigator(s) should determine the appropriate pre-medication.

Any pre-medications given will be documented as a concomitant medication.

If minor infusion reactions are observed, administration of acetaminophen, 500 mg to 1000 mg, antihistamines, and/or other appropriately indicated medications may be given prior to the start of infusions for subsequent participants.

Before the Infusion
Vital signs (temperature, heart rate, respiratory rate, blood pressure, and SpO2).

During the Infusion
Vital signs (temperature, heart rate, respiratory rate, blood pressure, and SpO2) will be measured every 15 minutes during the infusion.

After Infusion
Vital signs (temperature, heart rate, respiratory rate, blood pressure, and SpO2) will be measured every 30 minutes for 2 hours post infusion.

Only vital signs that meet AE reporting requirements will be recorded on an eCRF.

6.3.14 Laboratory Evaluations

Hematology
Participants will have blood drawn for complete blood cell count (CBC) with automated differential and platelet count. White blood cell count, hemoglobin, hematocrit, platelet count, absolute lymphocyte neutrophil and eosinophil counts will be recorded on an eCRF.

At Entry/Day 0, blood should be drawn before study drug administration.

Chemistry
Participants will have blood drawn for liver function tests (ALT, ALP, AST, total bilirubin, direct bilirubin, and total protein), and renal function tests (albumin, BUN, creatinine, potassium, glucose, and sodium) and recorded on an eCRF.

At Entry/Day 0, blood should be drawn before study drug administration.

Pregnancy Testing
For participants of reproductive potential: Serum or urine β-HCG. (Urine test must have a sensitivity of ≤25 mIU/mL).

Post-screening, pregnancy testing should be done any time pregnancy is suspected.

In the event of pregnancy occurring during the study, record pregnancy and pregnancy outcome per section 8.3.

6.3.15 Pharmacokinetics

Serum will be collected and used to measure investigational agent levels.

At Entry/Day 0, serum should be collected before the dose of investigational agent/placebo and again 30 minutes after the end of the infusion.

Post-entry, serum should be collected as per the SOE. Date and time of collection should be recorded.

Samples will be analyzed at a laboratory approved by the sponsor and stored at a facility designated by the sponsor. Concentrations of the investigational agent will be assayed using a validated bioanalytical method. Analyses of samples collected from placebo-treated subjects are not planned. Samples will be

retained for up to 2-years after last patient visit. Remaining samples used for PK may be pooled and used for exploratory metabolism or bioanalytical method experiments as deemed appropriate.

6.3.17 Anti-Drug Antibodies

Serum will be collected to measure anti-drug antibodies. At Entry/Day 0, serum should be collected before the dose of investigational agent/placebo.

Post-entry, serum should be collected as per the SOE (at the same time as serum collection for PK analysis). Date and time of collection should be recorded.

Samples will be analyzed at a laboratory approved by the sponsor and stored at a facility designated by the sponsor.

# 7.0 ADVERSE EVENTS AND STUDY MONITORING

## 7.1 Definitions of Adverse Events

Adverse Events of Special Interest
The following are AESIs for the agent LY3819253 or placebo for LY3819253:

- ≥ Grade 1 infusion-related reactions
- ≥ Grade 1 allergic/hypersensitivity reactions

# 8.0 CLINICAL MANAGEMENT ISSUES

## 8.2 Management of Side Effects

### 8.2.1 Overdose

There is no known antidote for LY3819253 overdose. In the event this occurs, the participant should be closely monitored for AE/SAE and laboratory abnormalities, and supportive care provided as indicated.

### 8.2.2 Infusion-Related Reactions

All participants should be monitored closely, as there is a risk of infusion reaction (including anaphylaxis) with any biological agent.

Symptoms and signs that may occur as part of an infusion reaction include, but are not limited to fever, chills, nausea, headache, bronchospasm, hypotension, angioedema, throat irritation, rash including urticaria, pruritus, myalgia, and dizziness.

The severity of infusion-related reactions will be assessed and reported using the Division of AIDS Table for Grading the Severity of Adult and Pediatric Adverse Events (DAIDS AE Grading Table), corrected Version 2.1, July 2017, which can

be found on the DAIDS RSC website at https://rsc.niaid.nih.gov/clinical-research-sites/daids-adverse-event-grading-tables.

The clinical site should have necessary equipment and medications for the management of any infusion reaction, which may include but is not limited to oxygen, IV fluid, epinephrine, acetaminophen and antihistamine.

Investigators should determine the severity of the infusion reaction and manage infusion reactions based on standard of care and their clinical judgment. If an infusion reaction occurs, then supportive care should be provided in accordance with the signs and symptoms.

A participant will stop investigational agent/placebo with ongoing dosing if a Grade 4 event occurs that is deemed related to the investigational agent/placebo. A participant will also stop the investigational agent/placebo with ongoing dosing for a participant if a Grade ≥2 allergic or hypersensitivity reaction occurs. Dosing can be modified for mild reactions (Grade ≥1), such as slowing infusion.

#### 8.2.3 Hypersensitivity

Signs and symptoms of infusion-related immediate hypersensitivity reactions may include, but are not limited to anaphylaxis, angioedema, bronchospasm, chills, diarrhea, hypotension, itching, skin rash, shortness of breath, urticaria, tachycardia, and throat irritation or tightness [8].

Participants will be closely monitored for immediate hypersensitivity reactions.

Sites should have appropriately trained medical staff and appropriate medical equipment available when study participants are receiving LY3819253. It is recommended that participants who experience a systemic hypersensitivity reaction be treated per the local standard of care.

### 8.3 Pregnancy

Since there are no data regarding the use of LY3819253 in participants who are pregnant, participants who are pregnant are not eligible for the study. Participants of childbearing potential and participants who may impregnate their partners are required to follow the instructions for prevention of pregnancy provided in the protocol.

If a participant becomes pregnant during the study (post-entry), study follow up will continue for the duration of the study.

At the end of the pregnancy, outcome and adverse events for participant and infant will be recorded on the outcome eCRF.

## 8.4 Breastfeeding

Since there are no data regarding the use of LY3819253 in participants who are breastfeeding, participants who are breastfeeding are not eligible for the study.

# 11.0 PHARMACOLOGY PLAN

## 11.1 Pharmacology Objectives

The phase II pharmacology objective is to determine the pharmacokinetics of LY3819253. For phases II and III, the pharmacology objective is to explore relationships between dose and concentration of LY3819253 with virology, symptoms, and oxygenation.

## 11.2 Pharmacology Study Design Overview

The Schedule of Evaluations shows the collection schedule for Phase II and for Phase III. LY3819253 has a long-elimination in preclinical animal studies, and is expected to be between 2-4 weeks in humans; the predicted elimination half-life based on the preclinical data is 19 days. Very limited data in participants who received a single dose of 700 mg indicated PK behavior consistent with expectations. The PK sample schedules are based on the long-elimination half-life of LY3819253 and are designed to meet the phase II objective of determination of LY3819253 pharmacokinetics and the phase III objective to explore dose/concentration-response relationships. By design, the sample collection schedules are different, with the phase II schedule being more intense to determine PK behavior, and the phase III schedule more sparse to confirm PK behavior and support dose/concentration-response analyses.

## 11.3 Pharmacology Data Analysis and Modeling

Pharmacokinetic data analysis of phase II data will use conventional and accepted approaches such as non-compartmental analysis or compartmental analysis to determine the PK characteristics of LY3819253. Population pharmacokinetic approaches (e.g. nonlinear mix effects modeling such as implemented in NONMEM) may also be used. The usual parameters of interest are area under the concentration-time curve (AUC), total body clearance (CL), elimination half-life ($T_{1/2}$), and maximum and minimum concentrations ($C_{max}$, $C_{min}$). Exploration of relationships between dose and concentration of LY3819253 with virology, symptoms, and oxygenation will be approached using conventional and accepted methods for pharmacokinetic/pharmacodynamic (PK/PD) data analyses. Such methods will include the $E_{max}$ or sigmoid $E_{max}$ model or structurally linked PK/PD models (as could be performed within NONMEM) to explore exposure-response relationships. Exposure-response relationships will be performed in conjunction with the protocol statisticians.

# 16.0 REFERENCES

1. Doherty M, Robertson MJ. Some early trends in immunology. Trends Immunol 2004;25:623-31.

2. Casadevall A, Dadachova E, Pirofski LA. Passive antibody therapy for infectious diseases. Nat Rev Microbiol 2004;2:695-703.
3. Saylor C, Dadachova E, Casadevall A. Monoclonal antibody-based therapies for microbial diseases. Vaccine 2009;27:G38-G46.
4. Casadevall A. The case for pathogen-specific therapy. Expert Opin Pharmacother 2009;10:1699-703.
5. IMpact-RSV Study Group. Palivizumab, a humanized respiratory syncytial virus monoclonal antibody, reduces hospitalization from respiratory syncytial virus infection in high-risk infants. Pediatrics 1998;102(3 Pt 1):531-7.
6. Fan P, Chi X, Liu L, et al. Potent neutralizing monoclonal antibodies against Ebola virus isolated from vaccinated donors. MAbs 2020;12(1):1742457.
7. ClinicalTrials.gov. A Study of LY3819253 (LY3819253) in Participants Hospitalized for COVID-19. ClinicalTrials.gov Identifier: NCT04411628. First posted June 2, 2020.
8. Everds NE, Tarrant JM. Unexpected hematologic effects of biotherapeutics in nonclinical species and in humans. Toxicol Pathol 2013;41:280-302.

APPENDIX IV: SAMPLE INFORMED CONSENT FOR STUDY DRUG LY3819253

One of the study drugs that you might be assigned to in this study is LY3819253 or the placebo for LY3819253.

LY3819253 is a type of drug called a monoclonal antibody. Many antibodies are naturally made by your body and help fight diseases. LY3819253 is made in a laboratory. "Monoclonal" means that LY3819253 is made up of many copies of just one antibody.

Your assignment is random, like the flip of a coin. You will have an equal chance of being in each of the two groups. You will not be able to choose your group, and neither you, your study doctor, nor the study staff at your site will know which group you are in.

The United States Food and Drug Administration (FDA) has not approved LY3819253 for general use by the public. However, we have told the FDA about this study and they have given us permission to conduct this study.

ARE THERE ANY ADDITIONAL STUDY PROCEDURES IF I RECEIVE LY3819253 OR PLACEBO?

<u>Screening Visit</u>

- At your screening visit, if you can become pregnant, you will be asked to give blood (1 teaspoon) or a urine sample for a pregnancy test. You cannot receive LY3819253 or placebo if you are pregnant.

<u>Entry Visit</u>

- You will have blood drawn. This blood will be used for the following tests:
    - routine safety tests (liver and kidney tests and blood counts)
    - levels of the drug in your blood
    - levels of antibodies to the drug (your body's immune response to the drug)
    - for future protocol-required testing
- You will have the infusion of LY3819253 or placebo. The infusion will be given through a small plastic tube that will be placed into a vein in your arm. This is called an intravenous (IV) infusion. The infusion will take approximately 1 hour. You will be monitored in the clinic for 2 hours after the end of the infusion.

<u>Study Visits</u>

After the Entry visit, your study visits and evaluations will be different depending on whether you are in the first part of the study or the second part of the study.

IF YOU ARE IN THE FIRST PART OF THE STUDY:

<u>Study Visits on Days 3, 14, and 28</u>

- You will have blood drawn. This blood will be used for the following tests:
    - routine safety tests (liver and kidney tests and blood counts)

- on days 14 and 28, levels of the drug and levels of antibodies to the drug (your body's immune response to the drug)

Study Visits on Week 12 and Week 24

- You will have blood drawn. This blood will be used for the following tests:
  - levels of the drug
  - levels of antibodies to the drug (your body's immune response to the drug)
  - for future protocol-required testing
- If you can become pregnant, you will be asked to give blood (1 teaspoon) or a urine sample for a pregnancy test. (week 24)

Extra Visits

If you have a bad reaction to the infusion of study drug, you may have to come back for two extra visits 4 weeks and 12 weeks after the reaction, unless there is another visit at this time.

- You will have blood drawn. This blood will be used for the following tests:
  - levels of the drug
  - levels of antibodies to the drug (your body's immune response to the drug)
  - levels of inflammatory markers and cells in your blood
- You will be asked to give a urine sample to check for markers of inflammation (week 4)

IF YOU ARE IN THE SECOND PART OF THE STUDY:

Study Visit on Day 28

- You will have blood drawn. This blood will be used for the following tests:
  - routine safety tests (liver and kidney tests and blood counts)
  - levels of the drug
  - levels of antibodies to the drug (your body's immune response to the drug)

Study Visits on Week 12 and Week 24

- You will have blood drawn. This blood will be used for the following tests:
  - levels of the drug
  - levels of antibodies to the drug (your body's immune response to the drug)

Extra Visits

If you have a bad reaction to the infusion of study drug, you may have to come back for two extra visits 4 weeks and 12 weeks after the reaction, unless there is another visit at this time.

- You will have blood drawn. This blood will be used for the following tests:
  - levels of the drug
  - levels of antibodies to the drug (your body's immune response to the drug)
  - levels of inflammatory markers and cells in your blood
- You will be asked to give a urine sample to check for markers of inflammation (week 4)

WHAT ARE THE RISKS OF LY3819253?

There is a risk of serious and/or life-threatening side effects when non-study medications are taken with the study drugs. For your safety, you must tell the study doctor or nurse about all medications you are taking before you start the study.

Another risk is that the study drug used in this study may have side effects, some of which are listed below. Additionally, the study drug tested in the study may have unknown side effects in persons with SARS-CoV-2 infection. In a research study, all of the risks or side effects may not be known before you start the study. You need to tell your doctor or a member of the study team immediately if you experience any side effects.

Please note that these lists do not include all the side effects seen with this study drug. These lists include the more serious or common side effects with a known or possible relationship to the study drug. If you have questions concerning the additional side effects, please ask the medical staff at your site.

<u>Risks Associated with LY3819253</u>
LY3819253 is being administered for the first time to hospitalized persons with COVID-19 in a separate study.

There is limited safety data on LY3819253 since it has not been given to a lot of people. As of June 22, 2020, there have been no serious unwanted effects reported by people taking LY3819253 or placebo to date. Most effects after taking LY3819253 or placebo have been mild or moderate and have either all gone away or are getting better. Three people had difficulty breathing that was severe; all these people have either recovered or are getting better.

One patient had chills that were mild and started a few hours after LY3819253 injection; they lasted 1 hour and were thought to be related to LY3819253.

Two people had a drop in white blood cell counts (below the normal range), which continued for a few days after LY3819253 or placebo administration and returned to normal again soon after that.

Administration of LY3819253 may result in allergic reactions. Signs and symptoms of these reactions include:
- chills
- skin rash
- itching
- hives
- swelling of the face or other soft tissues
- low blood pressure
- rapid heart rate
- throat irritation or tightness
- tightening of the muscles that line the airways
- shortness of breath
- loose stools

Administration of LY3819253 may induce release of chemicals called cytokines in the body. These chemicals may induce allergic reactions listed above as well as:

- fever
- muscle aches
- nausea
- vomiting
- headache
- dizziness

Some of these reactions may be serious or life-threatening including:

- skin rash
- swelling of the face or other soft tissues
- low blood pressure
- rapid heart rate
- throat irritation or tightness
- tightening of the muscles that line the airways
- shortness of breath

You will be monitored closely during administration of study drug. Medical personnel, equipment, and medication will be available to manage these reactions appropriately if they occur.

Administration of study drug may also cause the following risks and discomforts:

- development of proteins (antibodies) against LY3819253. This may cause your body to get rid of LY3819253 more quickly or change the effect of LY3819253 on the body. Your blood will be tested to find out whether your body made antibodies to LY3819253. The anticipated risk of this is low because LY3819253 is a fully human antibody. Therefore, it is less likely to be seen as “foreign” by your body’s immune system and your body is less likely to form antibodies against LY3819253.
- mixture of antibody and other chemicals in the body that may be deposited in tissues such as blood vessels and kidneys.
- unexpected increase in virus reproduction in your body. Although this has been observed with some viruses, this has not been observed with COVID-19 or with the use of serum-containing antibodies given to people with COVID-19. This risk of increased viral growth is perhaps greater when there is lower levels of antibodies in the blood in the presence of virus. To avoid this, LY3819253 will be given at a dose that is felt to be high enough to keep this from occurring.

ARE THERE RISKS RELATED TO PREGNANCY AND BREASTFEEDING?

<u>Pregnancy</u>
Since there are no data regarding the use of this study drug in people who are pregnant, you are not eligible to receive this study drug if you are pregnant.

The study drug may involve risks to you (or to the embryo or fetus, if you or your partner become pregnant), which are currently unforeseen.

If you are participating in sexual activity that could lead to you becoming pregnant, you must agree to use two forms of effective contraception, where at least one form is highly effective, for the entirety of the study and for 90 days after you receive the study drug.

Highly effective methods of contraception (less than 1% failure rate) include, but are not limited to:

- combination oral contraceptives
- implanted contraceptives
- intrauterine devices

Effective methods of contraception include, but are not limited to

- diaphragms with spermicide
- cervical sponges

If you engage in sexual activity that may lead to pregnancy in a partner, you must agree to either remain abstinent or use condoms with spermicide AND your partner must use one additional form of effective contraception, through 90 days after you receive the study drug.

Additional forms of effective contraception your partner may use include:

- hormone-based contraception (oral, patch, parenteral, implants, or vaginal ring)
- intrauterine device (IUD)

If applicable, if your partner is pregnant you must use condoms during vaginal intercourse through 90 days after you receive the study drug.

If applicable, you must not donate sperm through 90 days after you receive the study drug.

Let your doctor know immediately if you become pregnant. If you become pregnant while on the study, the study staff would like to obtain information from you about the outcome of the pregnancy (even if it is after your participation in the study ends).

<u>Breastfeeding</u>
It is not known if this study drug is safe to use in people who are breastfeeding. You are not eligible to receive this study drug if you are breastfeeding.

SIGNATURE PAGE

If you have read this consent form (or had it explained to you), all your questions have been answered and you agree to take part in this study, please sign your name below.

| | |
|---|---|
| Participant's Name (print) | Participant's Signature and Date |
| Participant's Legally Authorized Representative (As appropriate) | Legally Authorized Representative (print) Signature and Date |
| Study Staff Conducting Discussion (print) | Study Staff's Signature and Date Consent |
| Witness's Name (print) | Witness's Signature and Date (As appropriate) |

**ACTG NETWORK COORDINATING CENTER**
**Social & Scientific Systems**
**8757 Georgia Avenue, 12th Floor**
**Silver Spring, MD 20910-3714**
**Phone: 301-628-3000**
**Fax: 301-628-3302**

---

## CLARIFICATION MEMO

DATE: July 31, 2020

TO: A5401 Principal Investigators and Site Staff

FROM: A5401 Protocol Team

SUBJECT: Clarification Memo #1 for Protocol A5401

**This clarification memo (CM) does not result in a change in the protocol informed consent document. The Division of AIDS does not require sites to forward it to their institutional review board (IRB); however, each site must follow their IRB's policies and procedures. If IRB review of clarification memos is required at a site, please submit this document for review.**

**Each site should file a copy of this CM with the protocol for reference.**

**The protocol clarifications contained in this memo should be implemented immediately.**

The following are clarifications (noted in bold or strikethrough) to Protocol A5401, Version 1.0, 7/7/20, titled "Adaptive Platform Treatment Trial for Outpatients with COVID-19 (Adapt Out COVID)". These clarifications will be included in the next version of the A5401 protocol if it is amended at a future date.

1. Section 4.1.1.4, Inclusion Criteria

   Participants must be expected to begin study treatment no more than 10 days from self-reported onset of COVID-19-related symptoms or measured fever, defined as the self-reported date of first reported sign/symptom from the following list:
   - subjective fever or feeling feverish
   - cough
   - shortness of breath or difficulty breathing at rest or with activity
   - sore throat
   - body pain or muscle pain/aches
   - fatigue
   - headache
   - chills
   - nasal obstruction or congestion
   - nasal discharge
   - loss of taste or smell
   - nausea or vomiting
   - diarrhea
   - documented temperature ~~>37.8°C~~ **≥38**°C

2. Section 4.1.1.5, Inclusion Criteria

One or more of the following signs/symptoms present within 48 hours prior to study entry:

- subjective fever or feeling feverish
- cough
- shortness of breath or difficulty breathing at rest or with activity
- sore throat
- body pain or muscle pain/aches
- fatigue
- headache
- chills
- nasal obstruction or congestion
- nasal discharge
- nausea or vomiting
- diarrhea
- documented temperature ~~>37.8°C~~ **≥38**°C

3. Section 10.2.3.4, Secondary Outcome Measures

Phases II and III: Duration of fever through day 28 defined as the last day in the participant's study diary on which a temperature ~~greater than 37.8°C~~ **≥38**°C was recorded ~~or a potentially antipyretic drug, such as acetaminophen or ibuprofen, was taken~~.

**ACTG NETWORK COORDINATING CENTER**
**Social & Scientific Systems**
**8757 Georgia Avenue, 12th Floor**
**Silver Spring, MD 20910-3714**
**Phone: 301-628-3000**
**Fax: 301-628-3302**

---

## CLARIFICATION MEMO

DATE: August 23, 2020

TO: A5401 Principal Investigators and Site Staff

FROM: A5401 Protocol Team

SUBJECT: Clarification Memo #2 for Protocol A5401

**This clarification memo (CM) does not result in a change in the protocol informed consent document. The Division of AIDS does not require sites to forward it to their institutional review board (IRB); however, each site must follow their IRB's policies and procedures. If IRB review of clarification memos is required at a site, please submit this document for review.**

**Each site should file a copy of this CM with the protocol for reference.**

**The protocol clarifications contained in this memo should be implemented immediately.**

The following are clarifications (noted in bold or strikethrough) to Protocol A5401, Version 1.0, 7/7/20, titled "Adaptive Platform Treatment Trial for Outpatients with COVID-19 (Adapt Out COVID)". These clarifications will be included in the next version of the A5401 protocol if it is amended at a future date.

1. APPENDIX III: INVESTIGATIONAL AGENT LY3819253, Section 5.1, Regimen, Administration, and Duration

   Participants will be randomized to receive one of the following two regimens:

   Investigational Agent: LY3819253, 7000 mg, to be administered intravenously (IV) over approximately 60 minutes for one dose at study Entry/Day 0. **The entire contents of the IV bag must be infused to the participant.**

   *OR*

   Placebo for LY3819253: 0.9% Sodium Chloride for Injection, USP, to be administered IV over approximately 60 minutes for one dose at study Entry/Day 0. **The entire contents of the IV bag must be infused to the participant.**

2. APPENDIX III: INVESTIGATIONAL AGENT LY3819253, Section 5.2.2, Preparation, 5.2.2.1, LY3819253

   1. Remove ten (10) vials of LY3819253 from the refrigerator and an empty, sterile IV bag of appropriate size to contain 200 mL volume of LY3819253. Equilibrate the LY3819253 vials to room temperature**, not exceeding 30°C,** for approximately 20 minutes **(or no longer cool to the touch).**

2. Gently invert the vials by hand approximately 10 times to ensure homogeneity of the contents. Do not shake or vigorously agitate the vials. Visually inspect the vials for the presence of any visible particulate matter. If visible particulate matter is observed, appropriately discard the vials, obtain new vials, and restart the preparation.
3. Using appropriately sized syringes fitted with ~~21~~ **18**-gauge **(or larger gauge)** needles, withdraw 200 mL of LY3819253 solution from ten (10) vials. When the stopper of the first vial is punctured to start preparation, record this time as the investigational agent preparation time. Assign a ~~four~~-**seven**-hour beyond use date and time from the preparation time **if stored at room temperature or a 24-hour beyond use date and time from the preparation time if stored refrigerated.**
4. Inject the contents of each syringe prepared in Step 3 into an empty, sterile IV bag of appropriate size to contain 200 mL volume of LY3819253.
5. Gently invert the prepared IV bag by hand approximately ten times to ensure homogeneity of the contents. Do not shake or vigorously agitate the prepared bag. Avoid foaming. Visually inspect the bag after preparation. The contents of the bag should be free of any visible particulate matter. Obtain new vials and re-prepare the dose if visible particulate matter is observed.
6. Attach an infusion set containing a 0.2 or 0.22 **µm in-line** polyethersulfone (PES) ~~in-line~~ filter to the IV bag and prime the infusion set with the prepared investigational agent. Encase the IV bag ~~and the primed infusion set~~ in an opaque cover.

Prepared investigational agent in an IV bag should be administered <u>immediately</u>. If immediate administration is not possible, the investigational agent **may be held at refrigerated conditions for NOT MORE THAN 24 hours or at ambient light and room temperature conditions for NOT MORE THAN 7 hours. The hold time includes preparation + solution hold + infusion + flush. Any solution which exceeds these time period requirements must be discarded and a fresh solution must be prepared.** ~~should be stored at room temperature and administered within 4 hours of preparation~~ (refer to the assigned beyond use time in Step 3 above).

3. APPENDIX III: INVESTIGATIONAL AGENT LY3819253, Section 5.2.2, Preparation, 5.2.2.2, Placebo for LY3819253

1. Remove one 250 mL IV bag of 0.9% Sodium Chloride for Injection, USP from storage and one empty, sterile IV bag of appropriate size to contain 200 mL volume of 0.9% Sodium Chloride for Injection, USP.
2. Using appropriately sized syringes, withdraw 200 mL of 0.9% Sodium Chloride for Injection, USP from the 250 mL IV bag and inject into the empty, sterile IV bag. When the IV bag of 0.9% Sodium Chloride for Injection, USP is first punctured to start preparation, record this time as the placebo preparation time. Assign a ~~four~~**-seven**-hour beyond use date and time from the preparation time **if stored at room temperature or a 24-hour beyond use date and time from the preparation time if stored refrigerated.**
3. Visually inspect the bag after preparation. The contents of the bag should be free of any visible particulate matter. Obtain a new IV bag of 0.9% Sodium Chloride for Injection, USP and re-prepare the dose if visible particulate matter is observed.
4. Attach an infusion set containing a 0.2 or 0.22 **µm in-line** ~~micron~~ polyethersulfone (PES) ~~in-line~~ filter to the IV bag and prime the infusion set with the prepared placebo. Encase the IV bag ~~and the primed infusion set~~ in an opaque cover.

Prepared placebo in an IV bag should be administered <u>immediately</u>. If immediate administration is not possible, the placebo **may be held at refrigerated conditions for NOT MORE THAN 24 hours or at ambient light and room temperature conditions for NOT MORE THAN 7 hours. The hold time includes preparation + solution hold + infusion + flush. Any solution which exceeds these time period requirements must be discarded and a fresh solution must be prepared.** ~~should be stored at room temperature and administered within 4 hours of preparation~~ (refer to the assigned beyond use time in Step 2 above).

4. APPENDIX III: INVESTIGATIONAL AGENT LY3819253, Section 5.2.2, Preparation, 5.2.2.3, Labeling of Investigational Agent and Placebo

   Label the prepared IV bag with the following information:

   a. Participant identifier(s)
   b. Protocol number: ACTIV-2/A5401
   c. Investigational agent name: LY3819253 7000 mg or Placebo
   d. Total volume: 200 mL
   e. Route: IV
   f. Infusion rate/time: 200 mL/hour over 60 minutes
   g. Preparation date and time
   h. Beyond use date and time: ~~four~~ **seven** hours **at room temperature conditions or 24 hours at refrigerated conditions** after preparation
   i. Any additional information required by jurisdiction

**ACTG NETWORK COORDINATING CENTER**
**Social & Scientific Systems**
**8757 Georgia Avenue, 12th Floor**
**Silver Spring, MD 20910-3714**
**Phone: 301-628-3000**
**Fax: 301-628-3302**

---

**LETTER OF AMENDMENT**

DATE: October 1, 2020

TO: A5401 Principal Investigators and Site Staff

FROM: A5401 Protocol Team

SUBJECT: Letter of Amendment #1 for Protocol A5401

**The following information affects the A5401 study and must be forwarded to your institutional review board (IRB)/ethics committee (EC) as soon as possible for their information and review. This Letter of Amendment (LOA) must be approved by your IRB/EC before implementation.**

**The following information may also affect the Sample Informed Consent. Your IRB/EC is responsible for determining the process of informing participants of the contents of this LOA.**

**Upon receiving final IRB/EC and any other applicable regulatory entity approvals for this LOA, sites should implement the LOA immediately. Sites are still required to submit an LOA registration packet to the DAIDS Protocol Registration Office (PRO) at the Regulatory Support Center. Sites will receive a registration notification for the LOA once the DAIDS PRO verifies that all required LOA registration documents have been received and are complete. An LOA registration notification from the DAIDS PRO is not required prior to implementing the LOA. A copy of the LOA registration notification, along with this letter and any IRB/EC correspondence, should be retained in the site's regulatory file.**

**On September 16, 2020, preliminary results were reported from dose-finding study of the monoclonal antibody LY-CoV555 (also referred to as LY3819253), BLAZE-1, sponsored by Eli Lilly. Three doses (700mg, 2800mg, and 7000mg) were evaluated in non-hospitalized persons with early COVID-19. Based on these data and in discussion with Lilly, the protocol team will lower the dose of the investigational agent studied in the protocol from 7000mg to 700mg.**

The following are changes (noted in bold or strikethrough) to A5401, Version 1.0, 7/7/20, titled "Adaptive Platform Treatment Trial for Outpatients with COVID-19 (Adapt Out COVID)". These changes will be included in the next version of the A5401 protocol if it is amended at a future date. Changes that have already been made (either by Letter of Amendment or by Clarification Memo) have been incorporated in the excerpted text shown below (and are no longer presented in bold or strikethrough).

1. APPENDIX III: INVESTIGATIONAL AGENT LY3819253, Section 2.2, Rationale

   <u>Rationale for Dose of 700mg</u>
   - **On September 16, 2020, preliminary results were reported from a dose-finding study of the monoclonal antibody LY-CoV555 (also referred to as LY3819253), BLAZE-1, sponsored by Eli Lilly. Three doses (700 mg, 2800 mg, and 7000 mg) were evaluated in non-hospitalized persons with early COVID-19. This study reported that:**
   - **The primary endpoint of viral load change from baseline at day 11 was met for the 2800mg**

**dose, although all doses showed virologic response.**

- **Rate of hospitalizations and ER visits was 1.7 percent (5/302) for LY-CoV555 versus 6 percent (9/150) for placebo—a 72 percent risk reduction.**
- **LY-CoV555 was well tolerated across all doses with no drug-related serious adverse events. Of 409 persons who received LY-CoV555 or placebo, eight people experienced possible reactions to the infusion ("infusion reactions") that were mild or moderate, including itching, flushing, rash, and face swelling. All eight were able to complete the infusion and all symptoms resolved with or without an antihistamine.**

**Pharmacokinetic analysis suggests that a dose of 700mg will have a sustained concentration above in vitro IC90 of viral cell-entry neutralization for at least 28 days in 90% of the patient population. The benefits of this action include lower exposure of investigational agent to participants, expanded availability of this dose to the public if found effective and potential re-formulation of the compound to subcutaneous delivery instead of an infusion, which could expand its utility in an outpatient setting.**

2. APPENDIX III: INVESTIGATIONAL AGENT LY3819253, Section 5.1, Regimen, Administration, and Duration

Participants will be randomized to receive one of the following two regimens:

Investigational Agent: LY3819253, 700~~0~~ mg, to be administered intravenously (IV) over approximately 60 minutes for one dose at study Entry/Day 0. The entire contents of the IV bag must be infused to the participant.

OR

Placebo for LY3819253: 0.9% Sodium Chloride ~~for~~ Injection, USP, to be administered IV over approximately 60 minutes for one dose at study Entry/Day 0. The entire contents of the IV bag must be infused to the participant.

3. APPENDIX III: INVESTIGATIONAL AGENT LY3819253, Section 5.2.1, Formulation and Storage

Placebo for LY3819253 will be 0.9% Sodium Chloride ~~for~~ Injection, USP. The product must be locally sourced and stored according to the manufacturer's recommendation.

4. APPENDIX III: INVESTIGATIONAL AGENT LY3819253, Section 5.2.2, Preparation

Pharmacists must follow appropriate aseptic technique and sterile preparation procedures/ guidance as outlined in USP **General Chapter** <797> **Pharmaceutical Compounding – Sterile Preparations. LY3819253 and placebo should be prepared in a sterile environment, utilizing a biosafety cabinet/isolator. If a biosafety cabinet or isolator is not available, a laminar flow hood may be used.** Local regulations and site institutional policies and procedures for use of personal protective equipment, such as gloves, gowns, face masks and safety glasses, must be followed. Pharmacists should follow the requirements of their country, institution, and pharmacy regulatory authority regarding these procedures.

5. APPENDIX III: INVESTIGATIONAL AGENT LY3819253, Section 5.2.2, Preparation, 5.2.2.1, LY3819253

1. Remove ~~ten (10)~~ **one (1)** vial~~s~~ of LY3819253 from the refrigerator**, an appropriately sized IV bag of 0.9% Sodium Chloride Injection, USP from storage,** and an empty, sterile IV bag of appropriate size to contain 200 mL volume ~~of LY3819253~~. Equilibrate the LY3819253 vial~~s~~ to room temperature, not exceeding 30°C, for approximately 20 minutes (or no longer cool to the touch).

2. Gently invert the **LY3819253** vial~~s~~ by hand approximately 10 times to ensure homogeneity of the contents. Do not shake or vigorously agitate the vial~~s~~. Visually inspect the vial~~s~~ for the presence of any visible particulate matter. If visible particulate matter is observed, appropriately discard the vial~~s~~, obtain **a** new vial~~s~~, and restart the preparation.
3. **Using appropriately sized syringes fitted with 18-gauge (or larger gauge) needles, withdraw 180 mL of 0.9% Sodium Chloride Injection, USP from the bag obtained in Step 1 and inject into the empty, sterile IV bag.**
4. Using **an** appropriately sized syringe~~s~~ fitted with **an** 18-gauge (or larger gauge) needle, withdraw **20 mL** ~~200mL~~ of LY3819253 ~~drug product~~ solution from ~~ten (10)~~ **~~one (1)~~ the** vial**s obtained in Step 1.** When the stopper of the vial is punctured to start preparation, record this time as the investigational agent preparation time. Assign a seven-hour beyond use date and time from the preparation time if stored at room temperature or a 24-hour beyond use date and time from the preparation time if stored refrigerated.
5. Inject the contents of ~~each~~ **the** syringe prepared in Step ~~3~~ **4** into **the IV bag with Sodium Chloride Injection, USP prepared in Step 3, such that the IV bag now contains a total volume of 200 mL (180 mL of 0.9% Sodium Chloride Injection, USP and 20 mL of LY3819253)** ~~an empty, sterile IV bag of appropriate size to contain 200 mL volume of LY3819253~~.
6. Gently invert the prepared IV bag by hand approximately ten times to ensure homogeneity of the contents. Do not shake or vigorously agitate the prepared bag. Avoid foaming. Visually inspect the bag after preparation. The contents of the bag should be free of any visible particulate matter. Obtain ~~new vials~~ **a new LY3819253 vial** and re-prepare the dose if visible particulate matter is observed.
7. Attach an infusion set containing a 0.2 or 0.22 µm in-line polyethersulfone (PES) filter to the IV bag and prime the infusion set with the prepared investigational agent. Encase the IV bag in an opaque cover. **Note: an infusion set rated for at least 200 mL/hour flow rate should be used.**

Prepared investigational agent in an IV bag should be administered <u>immediately</u>. If immediate administration is not possible, the investigational agent may be held at refrigerated conditions for NOT MORE THAN 24 hours or at ambient light and room temperature conditions for NOT MORE THAN 7 hours. The hold time includes preparation + solution hold + infusion + flush. Any solution which exceeds these time period requirements must be discarded and a fresh solution must be prepared (refer to the assigned beyond use time in Step ~~3~~ **4** above).

6. APPENDIX III: INVESTIGATIONAL AGENT LY3819253, Section 5.2.2, Preparation, 5.2.2.2, Placebo for LY3819253

1. Remove ~~one~~ ~~250 mL~~ **an appropriately sized** IV bag of 0.9% Sodium Chloride ~~for~~ Injection, USP from storage and one empty, sterile IV bag of appropriate size to contain 200 mL volume of 0.9% Sodium Chloride ~~for~~ Injection, USP.
2. Using appropriately sized syringes, **fitted with 18-gauge (or larger gauge) needles,** withdraw 200 mL of 0.9% Sodium Chloride ~~for~~ Injection, USP from the ~~250 mL~~ IV bag **obtained in Step 1** and inject into the empty, sterile IV bag. When the IV bag of 0.9% Sodium Chloride ~~for~~ Injection, USP is first punctured to start preparation, record this time as the placebo preparation time. Assign a seven-hour beyond use date and time from the preparation time if stored at room temperature or a 24-hour beyond use date and time from the preparation time if stored refrigerated.
3. Visually inspect the bag after preparation. The contents of the bag should be free of any visible particulate matter. Obtain a new IV bag of 0.9% Sodium Chloride ~~for~~ Injection, USP and re-prepare the dose if visible particulate matter is observed.
4. Attach an infusion set containing a 0.2 or 0.22 µm in-line polyethersulfone (PES) filter to the IV bag and prime the infusion set with the prepared placebo. Encase the IV bag in an opaque cover. **Note: an infusion set rated for at least 200 mL/hour flow rate should be used.**

Prepared placebo in an IV bag should be administered <u>immediately</u>. If immediate administration is not possible, the placebo may be held at refrigerated conditions for NOT MORE THAN 24 hours or at

ambient light and room temperature conditions for NOT MORE THAN 7 hours. The hold time includes preparation + solution hold + infusion + flush. Any solution which exceeds these time period requirements must be discarded and a fresh solution must be prepared (refer to the assigned beyond-use time in Step 2 above).

7. APPENDIX III: INVESTIGATIONAL AGENT LY3819253, Section 5.2.2, Preparation, 5.2.2.3, Labeling of Investigational Agent and Placebo

   Label the prepared IV bag with the following information:
   a. Participant identifier(s)
   b. Protocol number: ACTIV-2/A5401
   c. Investigational agent name: LY3819253 700~~0~~ mg or Placebo
   d. Total volume: 200 mL
   e. Route: IV
   f. Infusion rate/time: 200 mL/hour over approximately 60 minutes
   g. Preparation date and time
   h. Beyond use date and time: Seven hours at room temperature conditions or 24 hours at refrigerated conditions after preparation
   i. Any additional information required by jurisdiction

8. APPENDIX III: INVESTIGATIONAL AGENT LY3819253, Section 5.3.1, Supply/Distribution

   LY3819253 will be provided by Eli Lilly and Company and will be available through the NIAID Clinical Research Products Management Center (CRPMC). ~~The site pharmacist should obtain the investigational agent(s) for this protocol by following the instructions in the manual *Pharmacy Guidelines and Instructions for DAIDS Clinical Trials Networks*.~~ **The site pharmacist will receive ordering instructions for the LY3819253 vials from the NIAID CRPMC.**

   0.9% Sodium Chloride ~~for~~ Injection, USP, infusion sets, and any other ancillary supplies will be locally sourced by the site.

9. APPENDIX III: INVESTIGATIONAL AGENT LY3819253, Section 5.3.2, Accountability

   The site pharmacist is required to maintain complete records of all investigational agents received from the NIAID CRPMC and subsequently dispensed. At US CRSs, all unused investigational agents must be returned to the NIAID CRPMC (or as otherwise directed by the sponsor) after the study is completed or terminated. ~~The procedures to be followed are provided in the manual *Pharmacy Guidelines and Instructions for DAIDS Clinical Trials Networks*.~~ At non-US CRSs, the site pharmacist must follow the instructions **provided by the NIAID CRPMC** ~~in the *Pharmacy Guidelines and Instructions for DAIDS Clinical Trials Networks*~~ for the destruction of unused investigational agents.

10. A Protocol Signature Page (PSP) is appended for submission to DAIDS Protocol Registration System (DPRS) as part of the LOA registration packet.

Adaptive Platform Treatment Trial for Outpatients with COVID-19 (Adapt Out COVID)

SIGNATURE PAGE

I will conduct the study in accordance with the provisions of this protocol and all applicable protocol-related documents. I agree to conduct this study in compliance with United States (US) Health and Human Service regulations (45 CFR 46); applicable US Food and Drug Administration regulations; standards of the International Conference on Harmonization Guideline for Good Clinical Practice (E6); Institutional Review Board/Ethics Committee determinations; all applicable in-country, state, and local laws and regulations; and other applicable requirements (e.g., US National Institutes of Health, Division of AIDS) and institutional policies.

Principal Investigator: ______________________________________________
Print/Type

Signed: ________________________________________ Date: ___________
Name/Title

**ACTG NETWORK COORDINATING CENTER**
**Social & Scientific Systems**
**8757 Georgia Avenue, 12th Floor**
**Silver Spring, MD 20910-3714**
**Phone: 301-628-3000**
**Fax: 301-628-3302**

---

**LETTER OF AMENDMENT**

DATE: October 27, 2020

TO: A5401 Principal Investigators and Site Staff

FROM: A5401 Protocol Team

SUBJECT: Letter of Amendment #2 for Protocol A5401

**The following information affects the A5401 study and must be forwarded to your institutional review board (IRB)/ethics committee (EC) as soon as possible for their information and review. This Letter of Amendment (LOA) must be approved by your IRB/EC before implementation. The following information may also affect the Sample Informed Consent. Your IRB/EC is responsible for determining the process of informing participants of the contents of this LOA.**

**Your site will receive this LOA along with the PPD notification letter with instructions for implementation at your site. Please provide PPD with the signed LOA. Upon receiving final IRB/EC and any other applicable regulatory entity approvals for this LOA, please provide the approvals to PPD. PPD will provide an amendment follow-up letter to your site prior to implementation.**

**PPD will submit a LOA registration packet to the DAIDS Protocol Registration Office (PRO) at the Regulatory Support Center on behalf of the sites. Sites will receive a registration notification for the LOA once the DAIDS PRO verifies that all required LOA registration documents have been received and are complete. An LOA registration notification from the DAIDS PRO is not required prior to implementing the LOA. A copy of the LOA registration notification, along with this letter and any IRB/EC correspondence, should be retained in the site's regulatory file.**

**Since the beginning of the protocol, laboratory supplies for assessment of pharmacokinetics (PK), antidrug antibodies (ADA), and infusion related reaction (IRR) have often been delayed from the supplier, Covance. These delays have delayed protocol sites from opening and open protocol sites from enrolling participants. These laboratory measures do not have safety consequences for protocol participants, and are not essential for primary endpoint determinations. The protocol team would, therefore, like to state that samples for these measures should be collected unless the required Covance kits for these assays are not available, in which case they do not have to be collected.**

The following are changes (noted in bold or strikethrough) to A5401, Version 1.0, 7/7/20, titled "Adaptive Platform Treatment Trial for Outpatients with COVID-19 (Adapt Out COVID)". These changes will be included in the next version of the A5401 protocol if it is amended at a future date. Changes that have already been made (either by Letter of Amendment or by Clarification Memo) have been incorporated in the excerpted text shown below (and are no longer presented in bold or strikethrough).

1. APPENDIX III: INVESTIGATIONAL AGENT LY3819253, Section 6.1-1: Schedule of Evaluations Phase II

| Phase II Evaluation | Screening | Entry / Day 0 | Day 2 | Day 3 | Day 7 | Day 14 | Day 21 | Day 28 | Week 12 | Week 24 | Hypersensitivity Reaction (Three Sample Time Points) | Premature Study D/C (Before Day 28) | Premature Study D/C (After Day 28) |
|---|---|---|---|---|---|---|---|---|---|---|---|---|---|
| Visit Window | | | +/-1 day | | +/-2 days | | +4 days | | +/-7 days | | | | |
| P = In Person Visit<br>R = Remote Visit | P/R | P | R | P | P | P | P | P | P | P | P | P | P |
| PK Studies[1] | | X | | | | X | | X | X | X | | X | X |
| Antidrug Antibodies[1] | | X | | | | X | | X | X | X | | X | X |
| Blood Collected for Evaluation of Hypersensitivity Reaction[1] | | | | | | | | | | | X | | |
| Urine Collected for Evaluation of Hypersensitivity Reaction[1] | | | | | | | | | | | X | | |

[1] **Samples should be collected unless the required Covance kits for these assays are not available**

2. APPENDIX III: INVESTIGATIONAL AGENT LY3819253, Section 6.1-2: Schedule of Evaluations Phase III

| Phase III Evaluation | Screening | Study Entry/Day 0 | Day 2 | Day 3 | Day 7 | Day 10 | Day 14 | Day 21 | Day 28 | Week 12 | Week 24 | Hypersensitivity Reaction (Three Sample Time Points) | Premature Study D/C (Before Day 28) | Premature Study D/C (After Day 28) |
|---|---|---|---|---|---|---|---|---|---|---|---|---|---|---|
| Visit Window | | | +/-1 day | | +/-2 days | | | +4 days | | +/-7 days | | | | |
| P = In Person Visit<br>R = Remote Visit | P/R | P | R | R | R | R | R | R | P | P | P | | P | P |
| PK Studies[1] | | X | | | | | | | X | X | X | | X | X |
| Antidrug Antibodies[1] | | X | | | | | | | X | X | X | | X | X |
| Blood Collected for Evaluation of Hypersensitivity Reaction[1] | | | | | | | | | | | | X | | |

| Phase III Evaluation | Screening | Study Entry/Day 0 | Day 2 | Day 3 | Day 7 | Day 10 | Day 14 | Day 21 | Day 28 | Week 12 | Week 24 | Hypersensitivity Reaction (Three Sample Time Points) | Premature Study D/C (Before Day 28) | Premature Study D/C (After Day 28) |
|---|---|---|---|---|---|---|---|---|---|---|---|---|---|---|
| Visit Window | | | +/-1 day | | +/-2 days | | | +4 days | | +/-7 days | | | | |
| P = In Person Visit<br>R = Remote Visit | P/R | P | R | R | R | R | R | R | P | P | P | | P | P |
| Urine Collected for Evaluation of Hypersensitivity Reaction[1] | | | | | | | | | | | | X | | |

[1] **Samples should be collected unless the required Covance kits for these assays are not available**

3. APPENDIX III: INVESTIGATIONAL AGENT LY3819253, Section 6.2.4, Timing of Evaluations, Event Driven Evaluations

   Hypersensitivity Reactions (see section 8.2.3)
   In the case of generalized urticaria or anaphylaxis occurring at any time following investigational agent administration through day 28, additional blood and urine samples should be collected at the following time points following instructions in the LPC **unless the required Covance kits for these assays are not available**:
   - ~~collect initial sample after the participant has been stabilized, and within 1 to 2 hours of the event; however, samples may be obtained as late as 12 hours after the event as analytes can remain altered for an extended period of time. Record the time at which the sample was collected~~
   - ~~obtain a follow-up sample at the next regularly scheduled visit or after approximately 4 weeks, whichever is later~~
   - ~~obtain a third follow-up sample at 12 weeks after the event~~
   - **Hypersensitivity Reaction Visit**
   - **Hypersensitivity Reaction Visit + 4 weeks post**
   - **Hypersensitivity Reaction Visit +12 weeks post**

4. APPENDIX III: INVESTIGATIONAL AGENT LY3819253, Section 6.3.15, Pharmacokinetics

   Serum will be collected and used to measure investigational agent levels **unless the required Covance kits for these assays are not available.**

5. APPENDIX III: INVESTIGATIONAL AGENT LY3819253, Section 6.13.17, Anti-Drug Antibodies

   Serum will be collected to measure anti-drug antibodies **unless the required Covance kits for these assays are not available.**

Adaptive Platform Treatment Trial for Outpatients with COVID-19 (Adapt Out COVID)

SIGNATURE PAGE

I will conduct the study in accordance with the provisions of this protocol and all applicable protocol-related documents. I agree to conduct this study in compliance with United States (US) Health and Human Service regulations (45 CFR 46); applicable US Food and Drug Administration regulations; standards of the International Conference on Harmonization Guideline for Good Clinical Practice (E6); Institutional Review Board/Ethics Committee determinations; all applicable in-country, state, and local laws and regulations; and other applicable requirements (e.g., US National Institutes of Health, Division of AIDS) and institutional policies.

Principal Investigator: ______________________________________________
Print/Type

Signed: ________________________________________Date: ___________
Name/Title

**ACTG NETWORK COORDINATING CENTER**
**Social & Scientific Systems**
**8757 Georgia Avenue, 12th Floor**
**Silver Spring, MD 20910-3714**
**Phone: 301-628-3000**
**Fax: 301-628-3302**

---

## LETTER OF AMENDMENT

DATE: November 13, 2020

TO: A5401 Principal Investigators and Site Staff

FROM: A5401 Protocol Team

SUBJECT: Letter of Amendment #3 for Protocol A5401

**The following information affects the ACTIV-2/A5401study and must be forwarded to your institutional review board (IRB)/ethics committee (EC) as soon as possible for their information and review. This Letter of Amendment (LOA) must be approved by your IRB/EC before implementation. The following information may also affect the Sample Informed Consent. Your IRB/EC is responsible for determining the process of informing participants of the contents of this LOA.**

**Your site will receive this LOA along with the PPD notification letter with instructions for implementation at your site. Please provide PPD with the signed LOA. Upon receiving final IRB/EC and any other applicable regulatory entity approvals for this LOA, please provide the approvals to PPD. PPD will provide an amendment follow-up letter to your site prior to implementation.**

**PPD will submit a LOA registration packet to the DAIDS Protocol Registration Office (PRO) at the Regulatory Support Center on behalf of the sites. Sites will receive a registration notification for the LOA once the DAIDS PRO verifies that all required LOA registration documents have been received and are complete. An LOA registration notification from the DAIDS PRO is not required prior to implementing the LOA. A copy of the LOA registration notification, along with this letter and any IRB/EC correspondence, should be retained in the site's regulatory file.**

The following are changes (noted in bold or strikethrough) to A5401, Version 1.0, 7/7/20, titled "Adaptive Platform Treatment Trial for Outpatients with COVID-19 (Adapt Out COVID)". These changes will be included in the next version of the A5401 protocol if it is amended at a future date. Changes that have already been made (either by Letter of Amendment or by Clarification Memo) have been incorporated in the excerpted text shown below (and are no longer presented in bold or strikethrough).

1. APPENDIX III: INVESTIGATIONAL AGENT LY3819253, **SCHEMA**

   DESIGN
   **After enrollment of approximately 220 participants in the phase II LY3819253 700mg arm, LY3819253 will enter directly into a phase III open-label, single-arm evaluation, without a graduation analysis of the phase II data. The phase III arm of LY3819253 will evaluate the safety of the investigational agent.**

**SAMPLE SIZE**
**Approximately 220 participants in the phase II evaluation of LY3819253 700mg (110 receiving LY3819253 and 110 receiving placebo). In phase III, enrollment will continue until another agent enters the study, at which point the phase III evaluation of LY3819253 700mg will close. At current enrollments rates, it is expected that the number of participants who will receive the investigational agent (including those enrolled in phase II) may be 300 or more.**

**REGIMEN**
- **Phase II: LY3819253 or placebo for LY3819253 700mg administered intravenously (IV) for one dose.**
- **Phase III: LY3819253 700mg IV administered IV for one dose.**

2. APPENDIX III: INVESTIGATIONAL AGENT LY3819253, **Section 1.1, Co-Primary Objectives**

**1.1.1 Phases II and III: To evaluate safety of the investigational agent.**

**1.1.2 Phase II: To determine efficacy of the investigational agent to reduce the duration of COVID-19 symptoms through study day 28.**

**1.1.3 Phase II: To determine the efficacy of the investigational agent to increase the proportion of participants with undetectable nasopharyngeal (NP) SARS-CoV-2 RNA at study days 3, 7, 14, 21, and 28.**

3. APPENDIX III: INVESTIGATIONAL AGENT LY3819253, **Section 1.2, Secondary Objectives**

**1.2.1 Phase II: To determine whether the investigational agent reduces a COVID-19 Severity Ranking scale based on COVID-19-associated symptom burden (severity and duration), hospitalization, and death, through study day 28.**

**1.2.2 Phase II: To determine whether the investigational agent reduces the progression of COVID-19-associated symptoms.**

**1.2.3 Phase II: To determine if the investigational agent reduces SARS-CoV-2 detection or levels of RNA in nasal swabs.**

**1.2.4 Phase II: To determine the pharmacokinetics of the investigational agent.**

**1.2.5 Phase II: To evaluate differences in SARS-CoV-2 RNA levels in NP swabs between the investigational agent versus placebo treatment groups and among subgroups of the population and risk groups defined by age and comorbidities.**

**1.2.6 Phase II: To determine if the investigational agent reduces SARS-CoV-2 detection or levels of RNA in saliva and nasal swabs.**

**1.2.7 Phase II: To determine efficacy of the investigational agent to obtain pulse oximetry measurement of ≥ 96% through day 28.**

**1.2.8 Phase III: Among participants receiving the investigational agent, to describe SARS-CoV-2 RNA levels in nasal swabs, symptom duration and severity, and proportion of participants hospitalized or dying through to Day 28.**

**1.2.9 Phase III: Among participants receiving the investigational agent, to explore associations between SARS-CoV-2 RNA levels in nasal swabs, symptom duration and severity, and risk of hospitalization/death.**

4. APPENDIX III: INVESTIGATIONAL AGENT LY3819253, **Section 1.3, Exploratory Objectives**

**1.3.1 Phases II and III: To explore the impact of the investigational agent on participant-reported rates of SARS-CoV-2 positivity of household contacts.**

**1.3.2 Phases II: To explore if baseline and follow-up hematology, chemistry, coagulation, viral, and inflammatory biomarkers are associated with clinical and virologic outcomes in relation to investigational agent use.**

**1.3.3 Phases II and III: To explore possible predictors of outcomes across the study population, notably sex, time from symptom onset to start of investigational agent, race/ethnicity, and risk groups defined by age and comorbidities.**

**1.3.4 Phases II and III: To explore if the investigational agent changes the hospital course once a participant requires hospitalization.**

**1.3.5 Phases II and III: To explore and develop a model for the interrelationships between virologic outcomes, clinical symptoms, hospitalization, and death in each study group.**

**1.3.6 Phases II and III: To explore the relationship between exposure to the investigational agent and SARS-CoV-2 innate, humoral, or cellular response, including anti-drug antibodies.**

**1.3.7 Phases II and III: To explore baseline and emergent viral resistance to the investigational agent.**

**1.3.8 Phases II and III: To explore the association between viral genotypes and phenotypes, and clinical outcomes and, in phase II, response to agents.**

**1.3.9 Phases II and III: To explore the association between host genetics and clinical outcomes and, in phase II, response to agents.**

**1.3.10 Phases II and III: To explore relationships between dose and concentration of investigational agent with virology, symptoms, and oxygenation.**

**1.3.11 Phases II and III: To explore the association between zinc and vitamin D levels and clinical outcomes and, in phase II, response to agents.**

**1.3.12 Phase II: To explore the impact of investigational agents on SARS-CoV-2 viremia, i.e., detection or level of SARS-CoV-2 RNA in the blood.**

**1.3.13 Phase II: To explore if self-collected nasal swabs and saliva correlate with the frequency of detection and levels of SARS-CoV-2 RNA in site-collected NP swabs.**

5. APPENDIX III: INVESTIGATIONAL AGENT LY3819253, Section 2.2, Rationale

Rationale for Dose of 700mg **and Graduation Directly to Open-Label, Single-Arm, Phase III Study**
On September 16, 2020, preliminary results were reported from a dose-finding study of the monoclonal antibody LY-CoV555 (also referred to as LY3819253 **and bamlanivimab**), BLAZE-1, sponsored by Eli Lilly. Three doses (700mg, 2800mg, and 7000mg) were evaluated in non-hospitalized persons with early COVID-19. This study reported that:

- The primary endpoint of viral load change from baseline at day 11 was met for the 2800mg dose, although all doses showed virologic response.
- Rate of hospitalizations and ER visits was 1.7 percent (5/302) for LY-CoV555 versus 6 percent (9/150) for placebo—a 72 percent risk reduction.

- LY-CoV555 was well tolerated across all doses with no drug-related serious adverse events. Of 409 persons who received LY-CoV555 or placebo, eight people experienced possible reactions to the infusion (“infusion reactions”) that were mild or moderate, including itching, flushing, rash, and face swelling. All eight were able to complete the infusion and all symptoms resolved with or without an antihistamine.

Pharmacokinetic analysis suggests that a dose of 700mg will have a sustained concentration above in vitro IC90 of viral cell-entry neutralization for at least 28 days in 90% of the patient population. **Selection of the 700mg dose allows** lower exposure of investigational agent to participants, expanded availability of this dose to the public if found effective and potential re-formulation of the compound to subcutaneous delivery instead of an infusion, which could expand its utility in an outpatient setting.

**On November 9, 2020, based on the available interim data from the BLAZE-1 trial, the FDA issued an Emergency Use Authorization (EUA) for LY3819253 in the United States for mild to moderate COVID-19 illness in outpatients. Access to the antibody through the EUA process will take time to implement and data collection during the EUA process will only be via FDA MedWatch reporting of serious adverse events or medication errors (EUA factsheet). The only studies outside of ACTIV-2 that are currently recruiting that include LY3819253 are a study of LY3819253 as monotherapy in low risk patients and a study of LY3819253 in combination with a second antibody (LY38324279) (NCT04427501). Clinical data for LY3819253 remain limited and the safety profile of LY3819253 monotherapy has not been established. Therefore, the current randomized comparison of LY3819253 will be converted in phase III to a single arm, open-label study to continue to capture more detailed safety data (primary objective) and to collect additional viral shedding, clinical symptom improvement, and hospitalization data (secondary objectives) using our phase III schedule of events. The intent is to continue this single arm study until another agent enters the study. This is likely to occur in November/December 2020. These data will enhance our understanding of the safety of this agent and provide correlative data between our phase II and phase III assessments.**

6. APPENDIX III: INVESTIGATIONAL AGENT LY3819253, Section 5.1, Regimen, Administration, and Duration

Participants will be randomized to receive one of the following two regimens:

Investigational Agent: LY3819253, 700~~0~~ mg, to be administered intravenously (IV) over approximately 60 minutes for one dose at study Entry/Day 0. The entire contents of the IV bag must be infused to the participant.
*OR*
Placebo for LY3819253: 0.9% Sodium Chloride for Injection, USP, to be administered IV over approximately 60 minutes for one dose at study Entry/Day 0. The entire contents of the IV bag must be infused to the participant.

**NOTE: Phase III is an open-label, single-arm evaluation of LY3819253; participants will not be randomized to receive placebo in phase III.**

7. APPENDIX III: INVESTIGATIONAL AGENT LY3819253, Section 6.3.9, Investigational Agent Administered

<u>After Infusion</u>
Vital signs (temperature, heart rate, respiratory rate, blood pressure, and SpO2) will be measured every 30 minutes for **one** ~~2~~ hour~~s~~ post infusion. **(This post-infusion observation period is consistent with the observation period required in the EUA for LY3819253)**

8. APPENDIX III: INVESTIGATIONAL AGENT LY3819253, **Section 10.2, Outcome Measures**

   **The outcomes in phase III will use the same definitions as the corresponding outcomes in phase II.**

9. APPENDIX III: INVESTIGATIONAL AGENT LY3819253, **Section 10.3, Randomization and Stratification**

   **There is no randomization in phase III. All participants will receive the investigational agent (700mg dose).**

10. APPENDIX III: INVESTIGATIONAL AGENT LY3819253, **Section 10.4, Sample Size**

    **The uncontrolled open-label study in phase III will be started after phase II is fully enrolled. The intent is to continue enrollment in phase III until the next investigational agent is opened to enrollment. This next agent is expected to start enrollment in December 2020. At current enrollments rates, it is expected that the number of participants who will receive the investigational agent (including those enrolled in phase II) may be 300 or more. As the intent of phase III is to provide additional safety data for the investigational agent, and to describe further SARS-CoV-2 RNA levels in nasal swabs, symptom duration and severity, and proportion of participants hospitalized or dying through to Day 28 among participants receiving the investigational agent, no formal power considerations are provided.**

11. APPENDIX III: INVESTIGATIONAL AGENT LY3819253, **Section 10.5, Data and Safety Monitoring**

    **No formal review of phase III will be undertaken by the DSMB unless the criteria described in Section 7 for triggering a safety review are met, or unless requested by the DSMB.**

12. APPENDIX III: INVESTIGATIONAL AGENT LY3819253, **Section 10.6, Analyses**

    **The descriptive analysis of data on safety and efficacy outcomes will be undertaken using the same methods as for phase II. Any comparisons, if undertaken, of the overall population of participants receiving the investigational agent to the placebo group enrolled during phase II will be considered as exploratory (as such comparisons are not randomized and so are subject to potential bias due to changes in the population of participants enrolled). Among participants receiving the investigational agent, analyses of associations between SARS-CoV-2 RNA levels in nasal swabs, symptom duration and severity, and risk of hospitalization/death will be exploratory.**

13. APPENDIX IV, SAMPLE INFORMED CONSENT FOR STUDY DRUG LY3819253, ARE THERE ANY ADDITIONAL STUDY PROCEDURES IF I RECEIVE LY3819253 OR PLACEBO?

    Entry Visit

    - You will have the infusion of LY3819253 or placebo. The infusion will be given through a small plastic tube that will be placed into a vein in your arm. This is called an intravenous (IV) infusion. The infusion will take approximately 1 hour. You will be monitored in the clinic for **one** ~~2~~ hour~~s~~ after the end of the infusion.

14. APPENDIX IV, SAMPLE INFORMED CONSENT FOR STUDY DRUG LY3819253

    **If you are in the first part of this study, o**ne of the study drugs that you might be assigned to ~~in this study~~ is LY3819253 or the placebo for LY3819253. **If you are in the second part of this study, you will be assigned to receive LY3819253. No participants will receive the placebo for LY3819253 in the second part of the study.**

The United States Food and Drug Administration (FDA) has not approved LY3819253 for general use by the public. However, we have told the FDA about this study and they have given us permission to conduct this study. **On November 9, 2020 the FDA issued an emergency use authorization (EUA) for LY3819253 for the treatment of mild-to-moderate COVID-19 in patients who are 12 years of age and older and who are at high risk for progressing to severe COVID-19 and/or hospitalization. The issuance of an EUA is different from FDA approval. The data for LY3819253 are still limited, but in deciding whether to issue an EUA, the FDA determined that the known and potential benefits of LY3819253 outweigh the known and potential risks for use during an emergency. Even though there is now an EUA for LY3819253, we want to continue to study LY3819253 so that we can collect more information about the safety of LY3819253 and risk factors associated with COVID-19 disease progression.**

Adaptive Platform Treatment Trial for Outpatients with COVID-19 (Adapt Out COVID)

SIGNATURE PAGE

I will conduct the study in accordance with the provisions of this protocol and all applicable protocol-related documents. I agree to conduct this study in compliance with United States (US) Health and Human Service regulations (45 CFR 46); applicable US Food and Drug Administration regulations; standards of the International Conference on Harmonization Guideline for Good Clinical Practice (E6); Institutional Review Board/Ethics Committee determinations; all applicable in-country, state, and local laws and regulations; and other applicable requirements (e.g., US National Institutes of Health, Division of AIDS) and institutional policies.

Principal Investigator: ________________________________________________
Print/Type

Signed: ____________________________________________Date: ____________
Name/Title

# Approvals

# ACTIV-2/ACTG A5401

# Primary Statistical Analysis Plan

# Version 2.0

## Adaptive Platform Treatment Trial for Outpatients with COVID-19

## (Adapt Out COVID)

## Based on Protocol Version 1.0, Clarification Memos #1 and #2,

## and Letters of Amendment #1, #2, and #3

**ClinicalTrials.gov Identifier: NCT04518410**

**January 19, 2021**

**Created by:**

**Carlee Moser, PhD; Justin Ritz, MS;**

**Mark Giganti, PhD; Michael Hughes, PhD;**

**Harvard T.H. Chan School of Public Health**

## Table of Contents

**TABLE OF CONTENTS** ..... 2

**VERSION HISTORY** ..... 4

**GLOSSARY OF TERMS** ..... 5

**1 INTRODUCTION** ..... 7
**1.1 Purpose** ..... 7
**1.2 Key Updates to the SAP** ..... 7
1.2.1 Version 2.0 ..... 7

**2 STUDY OVERVIEW** ..... 8
**2.1 Study Design** ..... 8
**2.2 Randomization Process** ..... 8
**2.3 Study Objectives** ..... 9
2.3.1 Primary Objectives ..... 9
2.3.2 Secondary Objectives ..... 10
2.3.3 Exploratory Objectives ..... 10
**2.4 Overview of Sample Size Considerations** ..... 11
2.4.1 Phase II ..... 11
2.4.2 Phase III ..... 12
**2.5 Overview of Formal Interim Monitoring** ..... 12
2.5.1 Phase II ..... 13
2.5.2 Phase III ..... 13
**2.6 Graduation to Phase III** ..... 15

**3 OUTCOME MEASURES** ..... 15
**3.1 Primary Outcome Measures Phase II** ..... 15
**3.2 Primary Outcome Measures Phase III** ..... 16
**3.3 Secondary Outcome Measures** ..... 16
**3.4 Other Outcome Measures** ..... 19

**4 STATISTICAL PRINCIPLES** ..... 21
**4.1 General Considerations** ..... 21

**5 ANALYSIS APPROACHES** ..... 23
**5.1 Analyses of the Primary Objectives** ..... 23
5.1.1 Primary Safety (Phase II and III) ..... 24
5.1.2 Primary Clinical Symptoms (Phase II) ..... 24
5.1.3 Primary Virologic (Phase II) ..... 26
5.1.4 Primary Efficacy (Phase III) ..... 28
**5.2 Analyses of Secondary Objectives** ..... 31
5.2.1 Secondary Clinical Symptoms ..... 31
5.2.2 Secondary Virology ..... 37

**5.3 Exploratory Analyses** .......... **40**
5.3.1 New SARS-CoV-2 among Household Contacts .......... 40
5.3.2 Hospitalization Course .......... 41
5.3.3 Exploratory Virology .......... 41
5.3.4 Resistance Mutations .......... 41
**5.4 Interim Analysis Considerations** .......... **41**
5.4.1 Phase II to Phase III Graduation Criteria .......... 41
5.4.2 Phase III Statistical Considerations .......... 41

**6 APPENDIX 1: STATISTICAL CONSIDERATIONS FOR LY3819253** .......... **43**
**6.1 Phase II** .......... **43**
**6.2 Phase III** .......... **43**
**6.3 Phase II: Exploratory Analysis Pooling Over the 7000mg and 700mg Doses** .......... **44**

## Version History

| Version | Changes Made | Date Finalized |
|---|---|---|
| 1 | Original Version | July 29, 2020 |
| 2 | Updated SAP to address the following:<br>- Changes to the protocol based on CMs and LOAs<br>- Typos/errors found after finalization of version 1.0<br>- Revised handing of missing symptom, virology, and oxygen saturation data in analysis<br>- Clarifying imputation of virology data<br>- Add analyses of resistance mutations<br>- Added LY3819253-specific appendix to address LOA #3 | Jan 19, 2021 |

**Glossary of Terms**

| | |
|---|---|
| ACTIV | Accelerating COVID-19 Therapeutic Interventions and Vaccines |
| AE | Adverse Event |
| AUC | Area Under the Curve |
| CSF | Critical Success Factor |
| CM | Clarification Memo |
| COVID-19 | Coronavirus Disease 2019 |
| DSMB | Data and Safety Monitoring Board |
| ECMO | Extracorporeal Membrane Oxygenation |
| GEE | Generalized Estimating Equations |
| ICU | Intensive Care Unit |
| IPCW | Inverse Probability of Censoring Weights |
| LOA | Letter of Amendment |
| LoD | Limit of Detection |
| LLoQ | Lower Limit of Quantification |
| LTFU | Loss to Follow Up |
| MCAR | Missing Completely at Random |
| mITT | Modified Intent-to-Treat |
| NIAID | National Institute of Allergy and Infectious Diseases |
| NP | Nasopharyngeal |
| SAP | Statistical Analysis Plan |
| SARS-CoV-2 | Severe Acute Respiratory Syndrome Coronavirus 2 |

| | |
|---|---|
| SOE | Schedule of Evaluations |
| TOC | Trial Oversight Committee |
| ULoQ | Upper Limit of Quantification |

# 1 Introduction

## 1.1 Purpose

This Primary Statistical Analysis Plan (SAP) describes the proposed content and general framework for the interim and primary statistical analysis reports of the phase II and phase III investigations of ACTIV-2/A5401. The Primary SAP addresses the primary, secondary and a subset of exploratory objectives of the study and describes the primary and secondary outcome measures for which results will be posted on ClinicalTrials.gov and that will be included in primary manuscripts. The Primary SAP outlines the general statistical approaches that will be used in the analysis of the study and has been developed to facilitate discussion of the statistical analysis components among the study team, industry collaborators, and study sponsor; and to provide agreement between the study team and statisticians regarding the statistical analyses to be performed and presented. Given the design of the study and that, multiple investigational agents will be studied; separate analysis reports may be generated for each investigational agent and each study phase. Analysis considerations that are specific to a given investigational agent are provided in corresponding supplements to this SAP.

## 1.2 Key Updates to the SAP

### 1.2.1 Version 2.0

The following revisions have been made to the SAP in version 2.0:

1) Changes to protocol via CMs and LOAs
    a. Updated fever duration outcome per CM #1
    b. Added Appendix #1 to address statistical considerations for the LY3819253 agent in phase III per LOA #3
    c. No changes to SAP based on CM #2 or LOAs #1 or #2
2) Fixed typos/errors and added clarifications
    a. Added AUC outcome for RNA from blood (plasma)
    b. Added analyses by time point for dichotomous virology and oxygen saturation outcome measures
    c. Clarified that time points with zero events excluded in joint hypothesis test of treatment effects for dichotomous virology and oxygen saturation outcome measures over time
    d. Clarified that “sporadic” missingness means “non-monotonic” for analysis of dichotomous virology and oxygen saturation outcome measures
    e. Clarified that duration of fever is calculated from start of investigational agent/placebo (instead of from study entry) to be consistent across all analyses
    f. Clarified calculation of rescaling of AUC for symptom severity outcome
    g. Clarified primary dichotomous oxygen saturation analysis will adjust for baseline and added supportive analysis that does not adjust for baseline, consistent with virology analyses
3) Virology imputation
    a. Clarified how imputation of virology results will be done given that result can be <LoD and/or <LLoQ, and some may be >ULoQ
    b. Corrected typo in calculation of virology AUC

4) Missing symptom and virology data
    a. Revised how missing symptom data (for reasons other than hospitalization or death) will be handled and clarified how to handle missing Day 0 symptom data
    b. Clarified how to handle missing diary cards at Day 0 in the AUC symptom severity analysis
    c. Updated imputation of missing virology or oxygen data at Day 28 to be considered "monotonic" missingness in dichotomous analysis
5) Removed DeLong, DeLong, Clarke-Pearson composite test for oxygen saturation and virology
6) Added details related to analysis windows
7) Added analyses for resistance mutations including adding baseline mutations as subgroup in primary analyses to LY3819253 appendix.

# 2 Study Overview

## 2.1 Study Design

ACTIV-2/A5401 is a master protocol to evaluate the safety and efficacy of investigational agents for the treatment of symptomatic non-hospitalized adults with COVID-19. It includes a phase II evaluation, followed by a transition into a larger phase III evaluation of promising agents that 'graduate' from phase II. The trial has a randomized, blinded, controlled adaptive platform study design that allows agents to be added or dropped during the course of the study for efficient testing of new agents against placebo within the same trial infrastructure. The graduation criteria may be changed (adapted) as new agents are included in the study and so analyses supporting the recommendation to graduate or otherwise are described in a separate analysis plan.

Eligible participants will have intensive follow-up through day 28, followed by limited follow up through week 24 to capture long-term safety information, hospitalizations or death.

The study population consists of adults (≥ 18 years of age) with documented positive SARS-CoV-2 molecular test results collected within 7 days prior to study entry with no more than 10 days of symptoms of COVID-19 prior to study entry, and with presence of select symptoms within 48 hours of study entry.

## 2.2 Randomization Process

The randomization process is designed to be flexible for this adaptive platform study, in which participants may be eligible for randomization to different investigational agents, and investigational agents can be added or dropped during the course of the study. The ultimate intent is having a similar number of participants on a given investigational agent and on the comparison group for that agent. The comparison group for a given investigational agent includes all participants who were concurrently randomized to a placebo, who were also eligible to have received that investigational agent.

To achieve this, eligible participants will be randomized in two steps. The first randomization will be to the Investigational Agent Group, and the second randomization will be to investigational agent or placebo within the Investigational Agent Group they were assigned in the first

randomization. Participants may be randomized to investigational agents that are in phase II evaluation or to agents that are in phase III evaluation.

For a given participant, the first randomization will occur at an equal ratio (e.g., 1:1, 1:1:1, …) with the ratio determined by the number (n) of investigational agents the participant is eligible to receive (if eligible for only one agent, then the participant would be assigned to the one appropriate Investigational Agent Group). For example, if there were three investigational agents and a participant was only eligible to receive two of the three (so n=2), the ratio used for their first randomization would be 1:1.

The second randomization will occur at a ratio of n:1, which is dependent on the number of investigational agents a participant is eligible to receive. In the example where a participant was only eligible for two of three available investigational agents, the second randomization to investigational agent or placebo would occur at a 2:1 ratio.

Both randomization steps will be stratified (using blocked randomization) by (1) time from symptom onset (≤ 5 days vs > 5 days), and (2) risk of progression to severe COVID-19 (‘high’ vs ‘low’), ‘high’ defined as a person with age ≥ 55 years or having a least one of several protocol-specified comorbidities.

Additional details on randomization are provided in protocol section 10.3.

### 2.3 Study Objectives

The following sections list the primary, secondary and exploratory objectives from the study protocol; corresponding protocol numbering is shown in brackets. This Primary SAP addresses all of the primary and secondary objectives shown below, with the exception of the secondary PK objectives in phase II, which will be addressed in supplementary analysis plans. In addition, exploratory objectives 1, 4, and 12 will also be addressed in this SAP; however, other exploratory objectives will be addressed in subsequent analysis plans.

#### 2.3.1 Primary Objectives

1) Phases II and III: To evaluate safety of the investigational agent [Protocol Objective 1.1.1].

2) Phase II: To determine efficacy of the investigational agent to reduce the duration of COVID-19 symptoms through study day 28 [Protocol Objective 1.1.2].

3) Phase II: To determine the efficacy of the investigational agent to increase the proportion of participants with undetectable nasopharyngeal (NP) SARS-CoV-2 RNA at study days 3, 7, 14, 21, and 28 [Protocol Objective 1.1.3].

4) Phase III: To determine if the investigational agent will prevent the composite endpoint of either hospitalization or death through study day 28. Hospitalization is defined as ≥24 hours of acute care, in a hospital or similar acute care facility, including Emergency Rooms or temporary facilities instituted to address medical needs of those with severe COVID-19 during the COVID-19 pandemic [Protocol Objective 1.1.4].

### 2.3.2 Secondary Objectives

1) Phases II and III: To determine whether the investigational agent reduces a COVID-19 severity ranking scale based on COVID-19-associated symptom burden (severity and duration), hospitalization, and death, through study day 28 [Protocol Objective 1.2.1].

2) Phases II and III: To determine whether the investigational agent reduces the progression of COVID-19-associated symptoms [Protocol Objective 1.2.2].

3) Phases II and III: To determine if the investigational agent reduces SARS-CoV-2 detection or levels of RNA in nasal swabs [Protocol Objective 1.2.3].

4) Phase II: To determine the pharmacokinetics of the investigational agent [Protocol Objective 1.2.4].

5) Phase II: To evaluate differences in SARS-CoV-2 RNA levels in NP swabs between the investigational agent versus placebo treatment groups and among subgroups of the population and risk groups defined by age and comorbidities [Protocol Objective 1.2.5].

6) Phase II: To determine if the investigational agent reduces SARS-CoV-2 detection or levels of RNA in saliva and nasal swabs [Protocol Objective 1.2.6].

7) Phase II: To determine efficacy of the investigational agent to obtain pulse oximetry measurement of ≥ 96% through day 28 [Protocol Objective 1.2.7].

8) Phase III: To evaluate differences in symptom duration between the investigational agent versus placebo treatment groups among subgroups of the population, and risk groups defined by age and comorbidities [Protocol Objective 1.2.8].

9) Phase III: To determine if the investigational agent will prevent the composite endpoint of either hospitalization or death through study week 24 [Protocol Objective 1.2.9].

### 2.3.3 Exploratory Objectives

1) Phases II and III: To explore the impact of the investigational agent on participant-reported rates of SARS-CoV-2 positivity of household contacts [Protocol Objective 1.3.1].

2) Phases II and III: To explore if baseline and follow-up hematology, chemistry, coagulation, viral, and inflammatory biomarkers are associated with clinical and virologic outcomes in relation to investigational agent use [Protocol Objective 1.3.2].

3) Phases II and III: To explore possible predictors of outcomes across the study population, notably sex, time from symptom onset to start of investigational agent, race/ethnicity, and risk groups defined by age and comorbidities [Protocol Objective 1.3.3].

4) Phases II and III: To explore if the investigational agent changes the hospital course once a participant requires hospitalization [Protocol Objective 1.3.4].

5) Phases II and III: To explore and develop a model for the interrelationships between virologic outcomes, clinical symptoms, hospitalization, and death in each study group [Protocol Objective 1.3.5].

6) Phases II and III: To explore the relationship between exposure to the investigational agent and SARS-CoV-2 innate, humoral or cellular response, including anti-drug antibodies, as appropriate per investigational agent [Protocol Objective 1.3.6].

7) Phases II and III: To explore baseline and emergent viral resistance to the investigational agent [Protocol Objective 1.3.7].

8) Phases II and III: To explore the association between viral genotypes and phenotypes, and clinical outcomes and response to agents [Protocol Objective 1.3.8].

9) Phases II and III: To explore the association between host genetics and clinical outcomes and response to agents [Protocol Objective 1.3.9]

10) Phases II and III: To explore relationships between dose and concentration of investigational agent with virology, symptoms, and oxygenation [Protocol Objective 1.3.10].

11) Phases II and III: To explore the association between zinc and vitamin D levels and clinical outcomes and response to agents [Protocol Objective 1.3.11].

12) Phase II: To explore the impact of investigational agents on SARS-CoV-2 viremia, i.e., detection or level of SARS-CoV-2 RNA in the blood [Protocol Objective 1.3.12].

13) Phase II: To explore if self-collected nasal swabs and saliva correlate with the frequency of detection and levels of SARS-CoV-2 RNA in site-collected NP swabs [Protocol Objective 1.3.13].

## 2.4 Overview of Sample Size Considerations

The following is adapted from the protocol; further details on the assumptions and sample size calculation are provided in protocol section 10.4.

### 2.4.1 Phase II

For each investigational agent in phase II, the proposed sample size in 220 participants, consisting of 110 participants who receive that agent and 110 participants who are concurrently randomized to placebo control. Participants who are randomized but do not start their randomized investigational agent or placebo will not be followed and will be replaced.

This sample size is chosen to give high power to identify an active agent on the basis of the primary virology outcome, due to limited data on the variability of symptom duration in the outpatient COVID-19 population.

Assuming 100 participants in each group will have NP swabs available at a scheduled measurement time, there is at least 82% power to detect a 20% absolute increase in the percentage of participants with undetectable virus in the investigational agent group vs concurrent placebo group, regardless of the assumed percent undetectable in the placebo group (range: 10-70%); calculated for the comparison of two proportions using a normal approximation to the binomial distribution, unpooled variance, and two-sided Type I error rate of 5%.

With respect to symptom duration, assuming 100 participants in each group will provide study diary data, the study will have 91% power to show a 20% relative reduction in median duration of symptoms, assuming:

- Log-10 Durations are normally distributed with 0.2 standard deviation;
- Wilcoxon rank sum test with two-sided 5% Type I error rate.

### 2.4.2 Phase III

For each investigational agent in phase III, the proposed sample size is 2000 participants consisting of 1000 participants who receive that agent and 1000 participants who are concurrently randomized to placebo control. Participants who are randomized but do not start their randomized investigational agent or placebo will not be followed and will be replaced.

This sample size has been chosen to provide 88.7% power to detect a relative reduction of 33.3% in the proportion of participants hospitalized/dying between the study groups. This is based on the following assumptions:

- Proportion hospitalized/dying in the placebo group is 15%;
- Two-sided test of two proportions with 5% Type I error rate;
- Three interim analyses and one final analysis, equally spaced, with stopping guideline for efficacy of an investigational agent versus concurrent placebo determined using the Lan-DeMets spending function approach with an O'Brien and Fleming boundary, and a non-binding stopping guidelines for futility using a Gamma(-2) Type II spending function also implemented using the Lan-DeMets spending function;
- Allowance for 5% of participants to be lost-to-follow-up prior to being hospitalized or dying, and non-informative loss-to-follow-up.

## 2.5 Overview of Formal Interim Monitoring

During the course of the study (phase II and phase III), an independent NIAID-appointed Data and Safety Monitoring Board (DSMB) will undertake reviews of interim data from the study. The following sections outline plans for interim monitoring during each phase of the study; additional details on monitoring can be found in protocol section 10.5. Statistical considerations for interim monitoring are shown in section 5.4 of this SAP.

Regardless of study phase, in the event that there is any death deemed related to investigational agent or placebo or if two participants experience a Grade 4 AE deemed related to investigational agent or placebo, enrollment to the investigational agent or placebo group will be paused and the DSMB will review interim safety data.

### 2.5.1 Phase II

During phase II, the DSMB will review interim data to ensure the safety of participants in the study, and to evaluate the activity of each investigational agent in order to provide graduation recommendations to the Trial Oversight Committee (TOC) via NIAID. The DSMB may recommend early termination of randomization to a particular investigational agent if there are safety concerns, but it is not intended to stop for futility in the phase II evaluation period.

There will be an interim analysis of a given investigational agent when 50% of participants (i.e., 110 of the 220 for a given investigational agent group) have completed the day 14 evaluation and all data (including virology) is available in the database. This review will include analyses of interim safety and will evaluate the activity of the investigational agent via assessment of graduation criteria; see section 5.4.1 for details on graduation rules.

At this early review, if activity data support graduation to phase III and there are no safety concerns, then the DSMB may recommend to continue enrollment of participants into phase III without a pause at the end of phase II enrollment (i.e., continue enrollment while results from complete phase II follow-up are still pending). However, at this early review, if activity data do not yet support graduation, then enrollment will be paused at the end of phase II enrollment (i.e., no enrollment into phase III), until a review of complete phase II results, through day 28, occurs.

Regardless if enrollment to phase III is paused, the DSMB will also review results from complete phase II follow-up once all participants (n=220) have completed the day 28 evaluation. For investigational agents that have not graduated to phase III, if these results indicate that graduation criteria have been met and there are no safety or resistance concerns, then the DSMB may recommend continuation of the study into the phase III period of evaluation.

At the interim reviews, recommendations for graduation will depend on an acceptable safety profile. This will largely be based on differences in the frequency of Grade 3 or 4 AEs between participants receiving the investigational agent and placebo.

### 2.5.2 Phase III

During phase III, the DSMB will review interim data to help ensure the safety of participants in the study, and to recommend changes to the study. The DSMB may recommend termination or modification of the study for safety reasons, if there is persuasive evidence of efficacy or lack of efficacy of an investigational agent versus placebo in preventing hospitalizations and deaths, or on the basis of statistical or operational futility. At each interim review, the DSMB will review summaries of data by unblinded randomized arms for the primary outcome of hospitalization/death, the secondary outcome of death, losses to follow-up, and adverse events (including early discontinuation of the investigational agent). By-stratum summaries will also be reviewed.

For monitoring the primary efficacy outcome, the O'Brien Fleming boundary will be used as the stopping guideline, implemented using the Lan-DeMets spending function to allow for changes in the timing or number of interim analyses if recommended by the DSMB.

Three interim efficacy analyses are planned during phase III, corresponding to 25%, 50%, and 75% of the expected maximal efficacy information of the trial. An additional early interim efficacy analysis will also be conducted at the end of phase II, which will be considered in calculating Type I error spending, though the total error spent at this analysis will be negligible given the early timing (i.e., ~10% of the expected information for a comparison of a given investigational agent vs placebo).

The expected maximal efficacy information available at the planned interim analyses is approximately proportional to the expected number of hospitalizations/deaths under design assumption parameters. Assuming 15% of participants will be hospitalized/die in the placebo/control group and 10% will be hospitalized/die in the investigational agent group (i.e., relative reduction of 33.3%), with 1000 participants per group, this corresponds to 250 participants hospitalized/died across both groups. Because of the uncertainty around the design assumptions, interim efficacy analyses will occur as follows (unless DSMB recommends otherwise):

- The earlier of when approximately 500 participants from the two groups combined (including phase II, 25% of the 2000) have been followed for the primary outcome assessed at day 28, or when approximately 62 participants in the two groups combined have been hospitalized/died (i.e. 25% of the expected 250 participants hospitalized/died);
- The earlier of when approximately 1000 participants from the two groups combined (50% of the 2000) have been followed for the primary outcome assessed at day 28, or when approximately 125 participants in the two groups combined have been hospitalized or have died;
- The earlier of when approximately 1500 participants from the two groups combined have been followed for the primary outcome assessed at day 28, or when approximately 187 participants in the two groups combined have been hospitalized of have died.

In considering possible modifications to the study or termination of the study for efficacy, the DSMB may also consider interim results for the secondary outcome of death, or for differences in the primary outcome within strata. The DSMB may make recommendations based on a high level of evidence for a difference between randomized arms, which might be based on application of the O'Brien and Fleming stopping guideline to the death outcome, or for those in one of the risk strata (e.g. high-risk participants or those treated closer to symptom onset). In these circumstances, consideration should be given to the increased risk of a Type I error.

There is the possibility that differences between the randomized arms may be observed at an early study time point (for example, cumulative proportion at day 6); however, the overall goal of the study is to prevent hospitalization and deaths regardless of the timing, and therefore the focus of the randomized arm comparisons will be at day 28.

The DSMB will monitor for statistical futility (i.e., stopping early for the absence of difference between groups). An investigational agent may be discontinued based on evidence of lack of effect or very limited effect compared with placebo/control. For the purpose of evaluating statistical futility, a moderately aggressive Type II error spending function, Gamma (-2) spending function implemented using the Lan-DeMets spending function approach, will be used.

The DSMB will also monitor operational futility. With respect to operational futility, the DSMB may recommend modification or termination of the study if the proportion hospitalized/die in the control group is much lower than expected in designing the trial. For example, the DSMB might recommend restricting or closing enrollment to the low-risk stratum in favor or increasing enrollment to the high-risk stratum. In addition, the DSMB will monitor the loss to follow-up (LTFU) rate. As a benchmark, an overall LTFU rate of more than 10% would be cause for concern.

Additional details on interim monitoring are provided in protocol section 10.5.

### 2.6 Graduation to Phase III

During the phase II period of the study, the DSMB will review interim safety and efficacy data to provide recommendations to the TOC via NIAID as to whether an investigational agent should graduate to phase III. The TOC will review DSMB recommendations, and may consider other secondary outcomes (e.g. dynamics of virologic measures and symptoms over time, or any evidence of viral rebound) in the decision to graduate an investigational agent from phase II to phase III.

The TOC will also consult with the company that owns the investigational agent, to determine the graduation decision. An independent, unblinded, group from the company will receive and review day 28 analysis data from the phase II comparisons of the investigational agent. The independent group will assist the company in deciding if the investigational agent should graduate to phase III and/or chose the dose of the phase III investigational agent. Based on these discussions and in consultation with the company, the TOC will decide whether an investigational agent enters into phase III.

## 3 Outcome Measures

All outcome measures are copied from the protocol. Only outcome measures addressed in this SAP are included below. See protocol section 10.2 for additional outcome measures.

### 3.1 Primary Outcome Measures Phase II

1) Safety: New Grade 3 or higher AE through 28 days. [For Primary Objective 1]

   New Grade 3 or higher AE is defined as: Grade 3 or higher event that was new in onset or aggravated in severity or frequency from the baseline condition (i.e., Grade 1 or 2 at baseline escalates to Grade 3 or higher, or Grade 3 at baseline escalates to Grade 4 or higher), following the start of study treatment.

2) Clinical (Symptom Duration): Duration of targeted COVID-19 associated symptoms from start of investigational agent (day 0) based on self-assessment. [For Primary Objective 2]

   Duration defined as the last day on or before study day 28 when any symptoms scored as moderate or severe at study entry are still scored as moderate or severe (i.e., not mild or absent), or any symptoms scored as mild or absent at study entry are scored as mild or worse (i.e., not absent). The targeted symptoms are fever or feeling feverish, cough, shortness of breath or difficulty breathing at rest or with activity, sore throat, body pain or muscle pain/aches, fatigue, headache, chills, nasal obstruction or congestion, nasal discharge (runny nose), nausea or vomiting, and diarrhea. Each symptom is scored daily by the participant as absent (score 0), mild (1) moderate (2) and severe (3).

3) Virologic: Detection (detectable versus undetectable) of SARS-CoV-2 RNA from site-collected NP swabs at days 3, 7, 14, 21, and 28.
   [For Primary Objective 3 and Secondary Objective 5]

### 3.2 Primary Outcome Measures Phase III

1) Safety: New Grade 3 or higher AE through 28 days. [For Primary Objective 1]

   New Grade 3 or higher AE is defined as: Grade 3 or higher event that was new in onset or aggravated in severity or frequency from the baseline condition (i.e., Grade 1 or 2 at baseline escalates to Grade 3 or higher, or Grade 3 at baseline escalates to Grade 4 or higher), following the start of study treatment.

2) Efficacy: Death from any cause or hospitalization during the 28-day period from and including the day of the first dose of investigational agent or placebo.
   [For Primary Objective 4]

   Hospitalization is defined as ≥24 hours of acute care, in a hospital or similar acute care facility, including Emergency Rooms or temporary facilities instituted to address medical needs of those with severe COVID-19 during the COVID-19 pandemic.

### 3.3 Secondary Outcome Measures

Safety

1) Phase II only: New Grade 2 or higher AE through 28 days.
   [Supportive of Primary Objective 1]

   New Grade 2 or higher AE is defined as: Grade 2 or higher event that was new in onset or aggravated in severity or frequency from the baseline condition (i.e., Grade 1 at baseline escalates to Grade 2 or higher, or Grade 2 at baseline escalates to Grade 3 or higher, or Grade 3 at baseline escalates to Grade 4 or higher), following the start of study treatment.

2) Phase II only: New Grade 2 or higher AE through week 24.
[Supportive of Primary Objective 1, with follow-up beyond day 28]

New Grade 2 or higher AE is defined as: Grade 2 or higher event that was new in onset or aggravated in severity or frequency from the baseline condition (i.e., Grade 1 at baseline escalates to Grade 2 or higher, or Grade 2 at baseline escalates to Grade 3 or higher, or Grade 3 at baseline escalates to Grade 4 or higher), following the start of study treatment.

3) Phase III only: New Grade 3 or higher AE through week 24.
[Supportive of Primary Objective 1, with follow-up beyond day 28]

New Grade 3 or higher AE is defined as: Grade 3 or higher event that was new in onset or aggravated in severity or frequency from the baseline condition (i.e., Grade 1 or 2 at baseline escalates to Grade 3 or higher, or Grade 3 at baseline escalates to Grade 4 or higher), following the start of study treatment.

<u>Clinical Symptoms</u>

4) Phase III only: Duration of targeted COVID-19 associated symptoms from start of investigational agent (day 0) through day 28 based on self-assessment.
[Supportive of Primary Objective 2 and Secondary 8]

Duration defined as the same as the primary phase II outcome.

5) Phase II and III: Duration of fever through day 28 defined as the last day in the participant's study diary on which a temperature ≥ 38°C was recorded
[Supportive of Primary Objective 2 and Secondary Objective 8]

6) Phase II and III: Time to self-reported return to usual (pre-COVID-19) health as recorded in a participant's study diary through day 28.
[Supportive of Primary Objective 2 and Secondary Objective 8]

7) Phase II and III: COVID-19 severity ranking based on symptom severity scores over time during the 28-day period from and including the day of the first dose of investigational agent or placebo, hospitalization, and death. [For Secondary Objective 1].

Participants who are alive at 28 days and not previously hospitalized, the severity ranking will be based on their area under the curve (AUC) of the symptom score associated with COVID-19 disease over time (through 28 days counting day 0 as the first day) defined as the sum of scores for the targeted symptoms in the participant's study diary (each individual symptom is scored as absent (score 0), mild (1) moderate (2) and severe (3)). Participants who are hospitalized or who die during follow-up through 28 days will be ranked as worse than those alive and never hospitalized as follows (in worsening rank

order): alive and not hospitalized at 28 days; hospitalized but alive at 28 days; and died at or before 28 days.

8) Phase II and III: Progression through day 28 of one or more COVID-19-associated symptoms to a worse status than recorded in the study diary at study entry, prior to start of investigational agent or placebo. [For Secondary Objective 2]

9) Phase II only: Oxygen saturation (i.e., pulse oximeter measure) categorized as <96 versus ≥96% through day 28. [For Secondary Objective 7]

10) Phase II only: Level (quantitative) of oxygen saturation (i.e., pulse oximeter measure) through day 28. [For Secondary Objective 7]

<u>Virology</u>

11) Phase II only: Level (quantitative) of SARS-CoV-2 RNA from site-collected NP swabs at days 3, 7, 14, 21, and 28.
[Supportive of Primary Objective 3 and Secondary Objective 5]

12) Phase II only: Area under the curve and above the assay lower limit of quantification of quantitative SARS-CoV-2 RNA over time from site-collected NP swabs at days 0, 3, 7, 14, 21 and 28. [Supportive of Primary Objective 3 and Secondary Objective 5]

13) Phase II and III: Detection (detectable versus undetectable) of SARS-CoV-2 RNA from participant-collected nasal swabs through day 28. [For Secondary Objective 3]

    Swabs collected at entry and days 1-14 in phase II, and at entry and days 3, 7, 10, 14, 21, and 28 in phase III.

14) Phase II and III: Level of SARS-CoV-2 RNA from participant-collected nasal swabs through day 28. [For Secondary Objective 3]

    Swabs collected at entry and days 1-14, 21 and 28 in phase II, and at entry and days 3, 7, 10, 14, 21, and 28 in phase III.

15) Phase II only: Area under the curve and above the assay lower limit of quantification of quantitative SARS-CoV-2 RNA over time from participant-collected nasal swabs daily at days 0-14 and at days 21 and 28. [For Secondary Objective 3]

16) Phase II only: Detection (detectable versus undetectable) of SARS-CoV-2 RNA from saliva at days 3, 7, 14, 21, and 28. [For Secondary Objective 6]

17) Phase II only: Level of SARS-CoV-2 RNA from saliva at days 3, 7, 14, 21, and 28.
[For Secondary Objective 6]

18) Phase II only: Area under the curve and above the assay lower limit of quantification of quantitative SARS-CoV-2 RNA over time from saliva samples at days 0, 3, 7, 14, 21, and 28. [For Secondary Objective 6]

Efficacy

19) Phase II only: Death from any cause or hospitalization during the 28-day period from and including the day of the first dose of investigational agent or placebo. [Supportive of Primary Objective 4]

    Hospitalization is defined as the same as the primary phase III outcome.

20) Phase II and III: Death from any cause during the 28-day period from and including the day of the first dose of investigational agent or placebo. [Supportive of Primary Objective 4]

21) Phase II and III: Death from any cause or hospitalization during the 24-week period from and including the day of the first dose of investigational agent or placebo. [For Secondary Objective 9, with follow-up beyond the day 28]

    Hospitalization is defined as the same as the primary phase III outcome.

22) Phase II and III: Death from any cause during the 24-week period from and including the day of the first dose of investigational agent or placebo. [For Secondary Objective 9, with follow-up beyond the day 28]

### 3.4 Other Outcome Measures

1) Phase II and III: New SARS-CoV-2 positivity among household contacts through to 28 days from start of investigational agent or placebo. [For Exploratory Objective 1]

2) Phase II and III: New SARS-CoV-2 positivity or COVID-19 symptoms among household contacts through to 28 days from start of investigational agent or placebo. [For Exploratory Objective 1]

3) Phase II and III: New SARS-CoV-2 positivity among household contacts through to 24 weeks from start of investigational agent or placebo. [For Exploratory Objective 1]

4) Phase II and III: New SARS-CoV-2 positivity or COVID-19 symptoms among household contacts through to 24 weeks from start of investigational agent or placebo. [For Exploratory Objective 1]

5) Phase II and III: Worst clinical status assessed using ordinal scale among participants who become hospitalized through day 28. [For Exploratory Objective 4]

Ordinal scale defined as:

Death
Hospitalized, on invasive mechanical ventilation or ECMO;
Hospitalized, on non-invasive ventilation or high flow oxygen devices;
Hospitalized, requiring supplemental oxygen;
Hospitalized, not requiring supplemental oxygen (COVID-19 related or otherwise).

6) Phase II and III: Duration of hospital stay among participants who become hospitalized through day 28.
[For Exploratory Objective 4]

7) Phase II and III: ICU admission (yes versus no) among participants who become hospitalized through day 28. [For Exploratory Objective 4]

8) Phase II and III: Duration of ICU admission among participants who are admitted to the ICU through day 28. [For Exploratory Objective 4]

9) Phase II and III: Worst clinical status assessed using ordinal scale among participants who become hospitalized through week 24. [For Exploratory Objective 4]

Ordinal scale defined as:

Death
Hospitalized, on invasive mechanical ventilation or ECMO;
Hospitalized, on non-invasive ventilation or high flow oxygen devices;
Hospitalized, requiring supplemental oxygen;
Hospitalized, not requiring supplemental oxygen (COVID-19 related or otherwise).

10) Phase II and III: Duration of hospital stay among participants who become hospitalized through week 24.
[For Exploratory Objective 4]

11) Phase II and III: ICU admission (yes versus no) among participants who become hospitalized through week 24. [For Exploratory Objective 4]

12) Phase II and III: Duration of ICU admission among participants who are admitted to the ICU through week 24. [For Exploratory Objective 4]

13) Phase II and III: Detection (detectable versus undetectable) of SARS-CoV-2 RNA in blood through day 28. [For Secondary Objective 6]

Blood collected at entry and days 7, 14, 21, and 28 in phase II, and at entry and day 28 in phase III.

14) Phase II and III: Level of SARS-CoV-2 RNA in blood through day 28.
[For Secondary Objective 6]

Blood collected at entry and days 7, 14, 21, and 28 in phase II, and at entry and day 28 in phase III.

15) Phase II only: Area under the curve and above the assay lower limit of quantification of quantitative SARS-CoV-2 RNA over time in blood. [For Secondary Objective 6]

    Blood collected at entry and days 7, 14, 21, and 28 in phase II.

16) Phase II and III: Emergence of any new resistance mutations after study entry. [For Exploratory Objective 6]

    New resistance mutations are mutations that were not present at entry that were observed after study entry.

# 4 Statistical Principles

## 4.1 General Considerations

The following analysis populations are defined for a given investigational agent:

- Screened Population: All participants who were screened for enrollment into the study, between the time of screening of the first and last participants who were eligible to be randomized to the given Investigational Agent Group.

- Randomized Population: All participants who were enrolled and were eligible to be randomized to the given Investigational Agent Group.

- Treated Population: All participants who could have been randomized to the given Investigational Agent Group and received any investigational agent/placebo (this is a modified intent-to-treat [mITT] population).

In general, the Treated Population is the focus of randomized comparisons to evaluate the safety and efficacy outcomes of an investigational agent versus placebo. Exclusion of participants who are randomized, but who do not start their investigational agent/placebo should not introduce bias as the study is blinded. In all analyses of a given investigational agent, the comparison group will include all participants who were concurrently randomized to a placebo, who were also eligible to have received the investigational agent of interest. The comparison group will pool across all relevant placebo groups. For the primary analysis of each investigational agent, a supplemental analysis will restrict the comparison group to include only participants who received the placebo for that investigational agent of interest.

Study visit windows for reporting are based on the Schedule of Evaluations (SOE) defined in the protocol (in person visits shown in the below table) and will be derived based on the evaluation/specimen date and study treatment initiation date (at interim analyses, if not available, study start date will be used). In the event that multiple results fall within the same analysis

window, the one closest to the target time point will be prioritized, or if equidistant from the target time point, the earlier result will be prioritized. For interim analyses, if a result does not fall in an analysis window, the visit label will be used to identify the target time point.

| SOE Visit | Protocol Range (Days) | Analysis Range (Days) | Analysis Window (Days) |
|---|---|---|---|
| Screening | -2, 0 | -10, 0 | -10, 0 |
| Day 0* | 0 | -1, 0 | -1, 0 |
| Day 3 | 2, 4 | 2, 4 | +/- 1 |
| Day 7 | 5, 9 | 5, 10 | -2, +3 |
| Day 14 | 12, 16 | 11, 17 | +/- 3 |
| Day 21 | 21, 25 | 18, 25 | -3, +4 |
| Day 28 | 28, 32 | 26, 38 | -2, +10 |
| Week 12 | 77, 91 | 56, 112 | +/- 28 |
| Week 24 | 161, 175 | 140, 196 | +/- 28 |

*The Day 0 analysis window is designed to capture data in scenarios where randomization occurs on the day prior to treatment initiation. Evaluations that occur on Day 0, post-treatment initiation (ex. vital signs evaluations), will consider the time of the evaluation compared to the time of treatment administration (and will be presented as ‘Day 0’ with the relative time). Windows cited above do not apply to data with daily collections (i.e., diary cards or nasal swabs).

Key study visits are Entry (Day 0), day 28, week 24, and the day of last dose of investigational agent/placebo (day X); day of last dose of investigational agent/placebo depends on the specific investigational agent dosing schedule and investigational agent or placebo, see relevant protocol appendix for details. Baseline is defined as the last available measure prior to the initiation of investigational agent/placebo.

Entry (Day 0): First dose of investigational agent/placebo occurs.

Day X: Last day of investigational agent/placebo.

See protocol appendices for details on specific investigational agents.

Day 28: Last day primary outcome may occur.

Week 24: Last study visit.

Statistical comparison across randomized arms of baseline characteristics are not planned because the study is randomized and placebo-controlled; hence, any differences should reflect chance variation. In addition, comparisons between investigational agents are not planned. Control of the Type I error rate will be undertaken separately for each investigational agent, and not across all investigational agents (i.e., not for the experiment-wise or family-wise error rate of the study).

Analyses of primary and secondary outcomes will not adjust for multiple comparisons. Analyses of primary outcomes will adjust for the multiple interim reviews using group sequential methods.

Continuous variables will be summarized using mean, standard deviation, median, interquartile range (Q1 and Q3), $10^{th}$ and $90^{th}$ percentile, and min and max; categorical variables will be summarized using frequency and percentage.

NIH requires that the primary outcomes also be summarized by randomized arm by sex/gender and by race/ethnicity, and that treatment interactions with sex/gender and race/ethnicity be evaluated.

SARS-CoV-2 RNA results may be below the assay lower limit of quantification (LLoQ) or above the upper limit of quantification (ULoQ). Values below the LLoQ and above the ULoQ will generally be considered as censored observations in statistical analyses (with left censoring at the LLoQ and right censoring at the ULoQ, respectively). However, if necessary for any analyses (and for graphical presentations), values may be imputed in the following manner:

- Values below the LLoQ, but above the limit of detection (LoD) will be imputed as half the distance from the log-10 transformed LoD to the log-10 transformed LLoQ
- Values below the LLOQ and below the LoD will be imputed as half the distance from zero to the log-10 transformed LoD;
- Values above the ULoQ will be imputed as one unit higher than the log-10 transformed ULoQ; actual values obtained from assay reruns with dilution will be used instead, if available.

# 5 Analysis Approaches

## 5.1 Analyses of the Primary Objectives

Analysis Population

The analyses of the primary objectives will include all randomized participants who started an investigational agent or the concurrent placebo, according to a modified intent-to-treat (mITT) approach (Treated Population). Participants who have protocol violations, such as those who start investigational agent or placebo outside of the protocol-defined study windows, or who are found to be ineligible, will be included in the analysis, but the protocol violations will be documented and described.

Note: According to the protocol, participants who are randomized but do not start investigational agent or placebo are not to be followed and will be replaced.

### 5.1.1 Primary Safety (Phase II and III)

<u>Analysis Approaches</u>

Occurrence of any new Grade 3 or higher AE through 28 days will be analyzed in the following manner. The proportion of participants who experienced a new Grade 3 or higher AE will be estimated and compared between randomized arms using log-binomial regression, with log link, in order to obtain a risk ratio estimate; the model will include a main effect for randomized arm. In the event the log-binomial regression model fails to converge, a Poisson regression model with robust variance and log-link will be used instead.

<u>Sensitivity Analyses</u>

Because some agents may be administered using injections or infusions and others will not be, the primary safety analyses will be repeated, but will exclude any occurrence of Grade 3 or higher local injection/infusion site reactions for investigational agents/placebos administered by injection or infusion.

<u>Supportive Analyses</u>

Secondary outcome 1 is included as supportive to the primary safety outcome in phase II. This outcome evaluates the occurrence of new Grade 2 or higher AEs through 28 days, and will be analyzed in the same manner as the primary outcome.

Secondary outcomes 2 and 3, which are included in support of the primary safety objective, evaluate the occurrence of new Grade 2 or higher AEs (in phase II) and Grade 3 or higher AEs (in phase III) through week 24. These outcomes will be analyzed separately as part of a supplementary analysis report (for week 24 outcomes) in the same manner as the primary safety outcomes.

In addition, for all analyses outlined above (primary, sensitivity, and supportive), the absolute difference in proportion of participants who experienced a new Grade 3 or higher AE (or new Grade 2 or higher AE) will be calculated, with associated 95% confidence interval (calculated using the normal approximation to the binomial distribution).

### 5.1.2 Primary Clinical Symptoms (Phase II)

<u>Analysis Approaches</u>

Duration of symptoms will be summarized with descriptive statistics. Participant specific durations will be compared between randomized arms using a two-sided Wilcoxon test with 5% type I error rate. In addition, Hodges-Lehmann estimate and associated 95% CI for the location shift between the two arms will also be provided.

The symptoms considered in calculating symptom duration are: feeling feverish, cough, shortness of breath or difficulty breathing at rest or with activity, sore throat, body pain or muscle pain/aches, fatigue (low energy), headache, chills, nasal obstruction or congestion (stuffy nose), nasal discharge (runny nose), nausea, vomiting, and diarrhea. Each of these symptoms is scored

daily in a study diary by the participant as absent (score 0), mild (1) moderate (2) and severe (3) from day 0 (pre-treatment) to day 28.

The symptom duration is defined as the time (days) from start of investigational agent/placebo to the last day on or before day 28 when any symptoms are reported as at least moderate for those that were moderate or severe at study entry, or are reported as at least mild for those that were mild or absent at study entry.

To operationalize this, a duration will be calculated for each targeted symptom. The symptom duration outcome measure will be the maximum duration across the targeted symptoms. For symptoms that are absent at study entry that remain as absent through day 28, a duration of zero will be assigned; however, for symptoms that are absent at entry and emerge as mild, moderate, or severe, duration will be calculated as the number of days from study entry to the last day the symptom was scored as mild, moderate, or severe. For symptoms that are a mild at study entry, the duration will be calculated as the number of days from study entry to the last day the symptom was scored as mild, moderate, or severe. For symptoms that are moderate or severe, the duration will be calculated as the number of days from study entry to the last day the symptom was scored as moderate or severe. For symptoms that remit during the 28-day period, but then reoccur, the period of remission will be ignored in calculating the duration.

Special considerations are made for participants who are hospitalized or die on or before day 28. For participants who become hospitalized on or before day 28, all symptoms are assumed to be at least moderate during hospitalization (i.e., imputed in analysis), regardless if they were present at study entry or at the time of hospitalization. Programmatically, all symptoms will be imputed as '*severe'* during hospitalization (starting from day of hospital admission through to the day before the day of hospital discharge or to day 28, whichever is earliest). Participants who die on or before day 28 will be ranked as the worst outcome (i.e., longest duration) in these analyses. Programmatically, all participants who die will be assigned a duration of 29 days. Diary cards that are filled out during hospitalization (starting from day of admission to day before day of discharge) will be ignored (as, per protocol, they are not required to be completed during hospitalization), and the algorithm outlined above (and in the protocol) will be used during the hospitalization period.

Missing values for reasons other than hospitalization or death will be imputed using the following algorithmic approach (after taking account of hospitalization and death as described above):

1) Impute missing value on Day 0 as "absent". If also missing on Day 1 or for a sequence of consecutive days from Day 1 but with at least one score during follow-up, impute the missing values through to the first available score as '*moderate*' [symptom duration will therefore be at least as long as the duration of a sequence of missing values starting at Day 0]

2) For intermittent missingness during follow-up, impute as the worst of (a) the last available value (actually provided by the participant or imputed due to hospitalization) before the missing value and (b) the first available value (actually provided by the participant or imputed due to hospitalization) after the missing value, irrespective of length of sequence

of missing values [this gives potentially longer times until symptom improvement or resolution if either of the preceding and succeeding values don't meet the criterion for improvement or resolution, but potentially shorter time if both the preceding and succeeding values meet the criteria].

3) For monotonic missingness through to Day 28 (i.e. a sequence of missing values through to and including Day 28 due to loss to follow-up or participant choice not to complete the diaries), impute as '*moderate*', hence assuming that the relevant criterion for improvement or resolution has not been met [this has the effect of lengthening the symptom duration].

<u>Sensitivity Analyses</u>

(1) The duration of symptoms analyses will be repeated using different assumptions for symptom scores that are missing for reasons other than hospitalization or death. In this analysis, the missing symptoms will be imputed as '*absent*' so having the effect of potentially shortening the symptom duration versus the imputation used in the primary analysis.

(2) A strength of the symptom duration definition is that it recognizes the possibility that symptoms may resolve and then reappear or may improve and then worsen. A weakness, however, is that the duration could be classified as long because, for example, of the appearance of a single symptom after a period with no symptoms. To assess sensitivity of the interpretation of the results to this type of issue, the following analysis of duration will be done. In this analysis, duration of symptoms will be defined as the time (days) from start of investigational agent/placebo to the day before two successive days of improved symptoms. Improved symptoms is defined as having all symptoms that were scored moderate/severe at baseline be resolved to absent/mild and all symptoms that were scored absent/mild at baseline be resolved to absent. Participants who are alive on day 28 and did not have two such successive days of improved symptoms, but all symptoms met these criteria on day 28, will be assigned a duration of 27 days; otherwise they will be assigned a duration of 28 days. Participants who die on or before day 28 will be assigned a duration as for the primary outcome definition above.

### 5.1.3 Primary Virologic (Phase II)

<u>Analysis Methods</u>

Descriptive statistics (number and percentage) will be used to describe the proportion of participants with undetectable SARS-CoV-2 RNA by NP swabs at each scheduled measurement time (entry and days 3, 7, 14, 21, and 28).

The proportion of participants with undetectable SARS-CoV-2 RNA will be compared between randomized arms using log-binomial regression for repeated binary measurements with log-link. This model will be fitted using generalized estimating equations (GEE) to handle the repeated

measurements with an independence working correlation structure and robust standard errors. For each time point after starting treatment, the model will include a main effect for time (indicator variable for each evaluation time), an interaction between time and randomized arm to evaluate differences between arms, and will adjust for baseline (day 0) log-10 transformed SARS-CoV-2 RNA level. The estimated adjusted relative risk of being undetectable (and associated 95% CI) will be obtained for each measurement time from the model by taking the exponential of the time*randomized arm interaction parameter estimate (and associated 95% CI) for that measurement time. In the event the log-binomial regression model fails to converge, a Poisson regression model with robust variance and log-link will be used instead.

A joint test of randomized arm across the time points will also be assessed, with degrees of freedom determined by the number of time points included. With this model, the comparison between randomized arms will use a two-sided Wald test with 5% type I error rate. Time points with zero events in either arm will not be included in the model (as estimation for such a model may be problematic; however data for these time points will be included in a descriptive summary of results over time points).

Missing data are assumed to be missing completely at random (MCAR) and will be ignored in the primary analysis. Sensitivity analyses will address possible informative missingness (see below).

In this analysis, baseline SARS-CoV-2 RNA values will be imputed as outlined in section 4.1. It is not expected that a high proportion of baseline results will be < LLoQ. However, in the event that there is a non-negligible amount of censoring (defined as 10% or more of baseline results < LLoQ), an additional variable will be added to the model that will indicate whether the baseline result was above or below assay lower quantification limit (included programmatically as “0” if above LLoQ, and “1” if below LLoQ).

<u>Sensitivity Analyses</u>

The following sensitivity analyses are included to evaluate impact of different assumptions on the inference of the virology outcomes.

1) Repeat primary analysis, but restrict analysis population to exclude those with undetectable SARS-CoV-2 RNA at Day 0. This model will adjust for baseline log-10 transformed SARS-CoV-2 RNA level with handling of detected levels below the LLoQ as described above.
2) Repeat primary analysis, but impute missing data in the following manner (ignores missingness due to hospitalization and death):
    - For non-monotonic missingness, participants with missing SARS-CoV-2 results will have their values imputed as undetectable if the preceding and succeeding results are undetectable, otherwise the results will be imputed as detectable.
    - For monotonic missingness, inverse probability weighted GEE will be used (as implemented in SAS PROC GEE [Lin G, Rodriguez RN. Weighted methods for analyzing missing data with the GEE procedure. Paper SAS166-2015. 2015.]; based on Robins and Rotnitzky. Journal of the American Statistical Association.

1995 Mar 1;90(429):122-9; Preisser, Lohman, and Rathouz. Statistics in Medicine. 2002 Oct 30;21(20):3035-54).

3) Repeat primary analysis, but impute missing data in the following manner (special considerations for missingness due to hospitalization and death):
    - For missingness due to hospitalization or death, participants with missing SARS-CoV-2 results will have their values imputed as *detectable*.
    - For non-monotonic missingness, participants with missing SARS-CoV-2 results will have their values imputed as *undetectable* if the preceding and succeeding results are undetectable, otherwise the results will be imputed as *detectable*.
    - For monotonic missingness, inverse probability weighted GEE will be used.

Supportive Analysis

The primary analysis will be repeated without adjustment for baseline (Day 0) SARS-CoV-2 RNA level. In addition, the absolute difference in proportion of participants with undetectable levels will be calculated at each measurement time, with associated 95% confidence intervals (calculated using the normal approximation to the binomial distribution).

Subgroup Analyses

To evaluate the effect of the investigational agent in specific populations, the primary virology outcome will be assessed among different subgroups. The same approaches outlined for the primary analysis will be implemented within each subgroup; formal comparisons across subgroups will not be done in phase II analyses. Pre-specified subgroups of interest include:

1) Sex (Male sex at birth, female sex at birth)
2) Race (white, non-white)
3) Ethnicity (Hispanic, non-Hispanic)
4) ‘Risk of Severe Disease’ Stratification (<55 years and no comorbidities, ≥ 55 years or at least one comorbidity)
5) Age Group (<55, ≥55)
6) Co-morbidity Status (no comorbidities, at least one comorbidity)
7) Calendar days from first symptom associated with COVID-19 to start of investigational agent/placebo Stratification (≤ 5 days, > 5 days)
8) Site (if applicable) or site location (if applicable)

    Subgroup analyses by site will be considered if there are a limited number of sites that contributed to enrollment. Otherwise, subgroup analyses by site location (e.g. by country or region) will be conducted if non-US sites contribute to enrollment.

### 5.1.4 Primary Efficacy (Phase III)

Analysis Approaches

The analysis of the primary efficacy outcome will compare the cumulative proportion of participants hospitalized or died (from any cause), from day 0 through day 28, between

randomized arms using a ratio of proportions; hospitalizations that begin on day 28 and deaths that occur on day 28 will be included. For analysis purposes, the integer scale will be used as the time scale, where study day 1 is considered day 1 and study day 28 is considered day 28; if an event occurs on day zero then event time will be set to 0.5 for analysis. The cumulative proportion will be estimated for each randomized arm using Kaplan-Meier methods to account for losses to follow up (and differential follow-up at the interim reviews). Participants will have follow-up censored at the date they were last known to be alive and not hospitalized through day 28. The primary analysis assumes non-informative censoring.

The absolute difference in the estimated log-cumulative proportion will be calculated between randomized arms; a 95% CI will be obtained for this difference in log-cumulative proportion calculated using a variance for this difference being the sum of the variances for each randomized arm obtained using Greenwood's formula. Results will be anti-logged to give the estimated ratio of cumulative proportions through day 28 (investigational agent vs concurrent placebo) and associated 95% CI. Two-sided 95% confidence intervals (CIs) and p-value (for the test of no difference between groups) will be obtained, which adjust for the interim analyses; a nominal 95% CI and p-value will also be provided.

Sensitivity Analyses

The following sensitivity analyses are included to evaluate impact of different assumptions on the inference of the primary comparisons. The third sensitivity analyses is an exploratory analysis.

1) Evaluate the composite outcome of being hospitalized, dead, or lost to follow up.

Approach: Repeat the primary analysis, but assume all participants who prematurely discontinue the study prior to day 28, who are unable to be contacted by the site to ascertain outcomes after discontinuation, had a primary event at day 28.

2) Evaluate the impact of participants enrolling from the same household.

Approach: Repeat the primary analysis only including the first participant who enrolled from each household.

In the event that differences are observed between the primary analysis and this sensitivity analysis, analysis methods that account for clustering will be considered, if feasible.

3) Exploratory: Evaluate the impact of differential loss-to-follow-up.

Approach: In the event that interpretation of the results for the primary analysis differs substantially between the primary analysis and the first sensitivity analysis, the impact of participants being LTFU will be explored using IPCW potentially using both pre-treatment variables and using variables after starting study treatment to

determine weights. The primary analysis will be repeated but, within each group, participants who are not LTFU will be weighted using IPCW determined by baseline variables that predict LTFU.

Supportive Analyses

Secondary outcome 20 is included as supportive to the primary efficacy outcome. The cumulative proportion of participants dead (from any cause) by day 28 will be analyzed in the same manner as the primary outcome.

Secondary outcomes 21 and 22, which address secondary objective 1.2.9 from the protocol, evaluate the proportion of participants who are hospitalized or died through week 24, and the proportion who die (from any cause) through week 24. These outcomes will be analyzed in the same manner as the primary efficacy outcome. In these analyses, however, participants will have their follow-up censored at the date they were last known to be alive and not hospitalized (or date they were last known to be alive) through 168 days (i.e. 24 times 7 days).

Secondary outcome 19 is included to assess the phase III primary efficacy outcome of hospitalization or death during phase II. This outcome will be analyzed in the same manner as the primary efficacy outcome in phase III if there are sufficient number of participants who died or were hospitalized. If not, descriptive summaries of the deaths and hospitalizations will be done in phase II.

Subgroup Analyses

To evaluate the effect of the investigational agent in specific populations, the primary outcome will be assessed among different subgroups. The same approaches outlined for the primary analysis will be implemented for each subgroup. Within each subgroup, the difference between randomized arms in the log-proportion will be estimated, and compared between subgroups by constructing a test of interaction and 95% confidence interval. This will be implemented by determining the difference between subgroups of the differences between randomized arms, and the variance of the difference will be determined by summing the variance of the subgroup-specific variances. In the event that the proportion of participants in a subgroup is low, or the number of events is low, descriptive summaries of the number of hospitalizations and deaths will be done. Pre-specified subgroups of interest include:

1) Sex (Male sex at birth, female sex at birth)
2) Race (white, non-white)
3) Ethnicity (Hispanic, non-Hispanic)
4) ‘Risk of Severe Disease’ Stratification (<55 years and no comorbidities, ≥ 55 years or at least one comorbidity)
5) Age Group (<55, ≥55)
6) Co-morbidity Status (no comorbidities, at least one comorbidity)
7) Calendar days from first symptom associated with COVID-19 to start of investigational agent/placebo Stratification (≤ 5 days, > 5 days)
8) Site (if applicable) or site location (if applicable)

Subgroup analyses by site will be considered if there are a limited number of sites that contributed to enrollment. Otherwise, subgroup analyses by site location (e.g. by country or region) will be conducted if non-US sites contribute to enrollment.

### 5.2 Analyses of Secondary Objectives

<u>Analysis Population</u>

The analyses of the COVID-19 symptoms will include all randomized participants who started an investigational agent or the concurrent placebo, according to a modified intent-to-treat (mITT) approach (Treated Population). Participants who have protocol violations will be included in the analysis but the protocol violations will be documented and described.

Note: Participants who are randomized but do not start investigational agent or placebo are, per protocol, not to be followed and will be replaced.

#### 5.2.1 Secondary Clinical Symptoms

<u>Analyses Methods</u>

*Duration of Clinical Symptoms*

Duration of clinical symptoms in phase III will be analyzed in the same manner as the primary phase II clinical symptom duration outcome.

*Progression of Symptoms*

Progression of one or more COVID-19-associated symptoms to a worse status than recorded in the study diary on day 0 (pre-treatment) on or before day 28 (i.e., absent to at least mild, mild to at least moderate, or moderate to severe) will be analyzed in the following manner. The proportion of participants who progressed will be estimated and compared between randomized arms using log-binomial regression, with log link, in order to obtain a risk ratio estimate; the model will include a main effect for randomized arm. In the event the log-binomial regression model fails to converge, a Poisson regression model with robust variance and log-link will be used instead. Participants who do not report worsened symptoms in study diaries, but are hospitalized or die in the first 28 days will be counted as having progression of symptoms in this analysis. Missing symptom scores not due to hospitalization or death will be imputed in the same manner as the primary symptom duration outcome (see above).

*Duration of Fever*

Duration of fever will be summarized with descriptive statistics, and will be compared between randomized arms using a two-sided Wilcoxon test with a 5% type I error rate. In addition, Hodges-Lehmann estimate and associated 95% CI for the location shift between the two arms will be provided.

The calculation of fever duration will take into consideration the temperature readings reported by the participants.

The fever duration is defined as the time (days) from start of investigational agent/placebo to the last day on or before day 28 when a fever was reported (temperature ≥ 38°C).

Participants who never report a temperature ≥ 38°C will be assigned a duration of fever of zero days. For the main analysis, special considerations will not be made for missing diary cards due to hospitalization or death (as it is possible that all fevers resolved prior to hospitalization or death). As fevers are expected to be very infrequent at study entry, missing fever evaluations on diary cards are assumed to be missing completely at random (MCAR) and will be ignored in these analyses. Programmatically, missing fever evaluations on diary cards for any reasons will have fever imputed as "*no*".

*Return to Usual Health*

Duration of time without self-reported return to usual health will be summarized with descriptive statistics, and will be compared between randomized arms using a two-sided Wilcoxon test with a 5% type I error rate. In addition, Hodges-Lehmann estimate and associated 95% CI for the location shift between the two arms will be provided.

The study diary includes a question: "*Have you returned to your usual (pre-COVID) health today?*" which is answered each day with possible responses "*yes*" or "*no*". Duration of time without self-reported return to usual health is defined as the time (days) from start of treatment to the last day on or before day 28 that self-reported return to usual health was "*no*".

Participants who never report "no" after starting study treatment will be assigned a time of zero days.

Special considerations are made for participants who are hospitalized or die on or before day 28. For participants who are hospitalized, the diary card answer is imputed as "no" for the period of hospitalization. Programmatically, self-reported return to usual health will be imputed as '*no'* starting from day of hospital admission through to day of hospital discharge or day 28, whichever is earliest. Diary cards that are filled out during hospitalization will be ignored (as, per protocol, they are not required to be completed during hospitalization), and the algorithm outlined above (and in the protocol) will be used during the hospitalization period. Participants who die on or before day 28 will be ranked as the worst outcome (i.e., longest time without return to usual health) in these analyses. Programmatically, all participants who die will be assigned a time of 29 days.

Return to Health answers that are missing for reasons other than hospitalization or death will be imputed in the analysis using the worst of the succeeding and preceding values. Return to Health answers that are missing at Day 0 and in a sequence of values starting at Day 0 for reasons other than hospitalization and death will imputed as "no". Monotonic missing values through to Day 28 will be imputed as "no".

*COVID-19 Severity Ranking*

COVID-19 severity ranking will be summarized with descriptive statistics. Participant specific scores will be compared between randomized arms using a two-sided Wilcoxon test with a 5% type I error rate. In addition, Hodges-Lehmann estimate and associated 95% CI for the location shift between the two arms will be provided.

The symptoms considered in calculating symptom duration are: feeling feverish, cough, shortness of breath or difficulty breathing at rest or with activity, sore throat, body pain or muscle pain/aches, fatigue (low energy), headache, chills, nasal obstruction or congestion (stuffy nose), nasal discharge (runny nose), nausea, vomiting, and diarrhea. Each of these symptoms is scored daily in a study diary by the participant as absent (score 0), mild (1) moderate (2) and severe (3) from day 0 (pre-treatment) to day 28.

COVID-19 severity ranking is defined as the participant-specific AUC of the total symptom score associated with COVID-19 disease, over time (through 28 days counting day 0 as the first day). For participants who are alive and were never hospitalized on or before day 28, the total symptom score on a particular day is the sum of scores for the targeted symptoms in the participant's study diary for that day. The AUC will be calculated using the trapezoidal rule and is defined as the area below the line formed by joining total symptom scores on each daily diary card from day 0 through day 28. The AUCs will be rescaled by time by dividing by 28, corresponding to the number of trapezoids created from daily diary cards between day 0 and day 28, in order to provide results on a symptom scale from 0 to 39.

Special considerations are made for participants who are hospitalized or die on or before day 28. Participants who are hospitalized or who die during follow-up through day 28 will be ranked as worse (i.e., worse severity) than those alive and never hospitalized through day 28 as follows (in worsening rank order): alive and not hospitalized at day 28; alive but hospitalized at day 28; and died on or before day 28. Programmatically, participants who were hospitalized, but are alive and no longer hospitalized at day 28 will be assigned an AUC (severity score) of 40, participants who are alive but remain hospitalized at day 28 will be assigned an AUC (severity score) of 41, and participants who die (regardless of when the death occurred through day 28) will be assigned a severity score of 42.

Participants who have incomplete diary cards for reasons other than hospitalization or death, and who are not subsequently hospitalized and do not die through day 28, will be addressed in the following manner:

1) Participants who are missing day 0 total symptom scores (i.e., participants who failed to complete the diary card on Day 0 and have no scores for any symptoms) will have their total symptom score imputed as the mean day 0 total symptom score among participants who report a total symptom score on day 0;
2) Participants who have some symptom scores missing at Day 0 (i.e., completed the diary card but did not score all symptoms) will have their total symptom score calculated as the mean of the available symptoms scores at Day 0, multiplied by 13;

3) Participants who stop completing their symptom diaries before day 28 will have their last total symptom score carried forward through day 28, and their AUC calculation done as noted above;
4) Participants who have diary cards with some, but not all symptom scores reported, their missing symptoms scores will be linearly interpolated based on the preceding and succeeding available scores for a given symptom, and their AUC calculation done as noted above;
5) Participants who have intermittent days with no symptom scores reported (i.e., all scores missing), their missing scores will be ignored in the AUC calculation, which is analogous to interpolating the total symptom scores.

Methods such as multiple imputation or IPCW may be considered if more than 10% of participants in either group stop completing their diaries before day 28 for reasons other than death or hospitalization.

To programmatically implement the imputation of the missing diary cards in order to calculate the AUC for participants who are not hospitalized and do not die by day 28, the following steps will be followed. First, imputation of total symptom scores will be done according to (1), (2), and (3). Next, (4) intermittent missing symptom scores for particular symptoms will be imputed using linear interpolation (see below formula) of the preceding and succeeding scores. Note: no imputation done for (5).

X = (Succeeding Score – Preceding Score) ÷ (Succeeding Day – Preceding Day)

Score on $1^{st}$ Day missing = 1*X + Preceding Score

Score on $2^{nd}$ Day missing = 2*X + Preceding Score

…..

Score on $Z^{th}$ Day missing = Z*X + Preceding Score.

*Oxygen Saturation*

Participants who are on supplemental oxygen at day 0 (pre-treatment) will not be included in these analyses.

Oxygen saturation will be analyzed in the same manner as the virology outcomes.

Descriptive statistics (number and percentage) will be used to describe the proportion of participants with oxygen saturation ≥ 96% at each scheduled measurement time (day 0 [pre-treatment] and days 3, 7, 14, 21, and 28).

The proportion of participants with any oxygen saturation values ≥ 96% will be compared between randomized arms using log-binominal regression for binary repeated measurements with log-link. This model will be fitted using generalized estimating equations (GEE) to handle the repeated measurements with an independence working correlation structure and robust standard

errors. For each time point after starting treatment, the model will include a main effect for time (indicator variable for each evaluation time), and an interaction between time and randomized arm to evaluate differences between arms, and will adjust for baseline oxygen saturation level. The estimated adjusted relative risk of having oxygen saturation values ≥ 96% (and associated 95% CI) will be obtained for each measurement time from the model by taking the exponential of the time*randomized arm interaction parameter estimate (and associated 95% CI) for that measurement time. In the event the log-binomial regression model fails to converge, a Poisson regression model with robust variance and log-link will be used instead.

A joint test of randomized arm across the time points will also be assessed, with degrees of freedom determined by the number of time points included. With this model, the comparison between randomized arms will use a two-sided Wald-test with 5% type I error rate. Time points with zero events in either arm will not be included in the model (as estimation for such a model may be problematic; however data for these time points will be included in a descriptive summary of results over time points).

Missing data are assumed to be missing completely at random (MCAR) and will be ignored in this analysis. Sensitivity analyses will address possible informative missingness (see below).

Non-parametric Wilcoxon rank-sum tests with a 5% type I error rate will compare oxygen saturation levels (continuous) between randomized arms, separately at each post-entry study day. In addition, Hodges-Lehmann estimate and associated 95% CI for the location shift between the two arms will also be provided.

<u>Sensitivity Analyses</u>

The following sensitivity analyses are included to evaluate impact of different assumptions on the inference of the clinical symptoms outcomes.

*Duration of fever*

1) Repeat duration of fever analyses, however, special considerations will be given for missing diary cards due to hospitalization or death. For participants who are hospitalized on or before day 28, fever will be assumed to be present during hospitalization. Programmatically, fever will be imputed as "*yes*" during hospitalization (starting from day of hospital admission through to day of hospital discharge or day 28, whichever is earliest). Participants who die on or before day 28 will be ranked as the worst outcome (i.e., longest duration) in these analyses. Programmatically, all participants who die on or before day 28 will be assigned a duration of 29 days.
2) Repeat duration of fever analyses, but define duration of fever as the time from day 0 to the last day on or before day 28 when a fever was reported (temperature ≥ 38°C was recorded) or anti-pyretic medications were reported as being used in the participant's diary. This analysis makes special considerations for participants who indicated using anti-pyretic medications (i.e., will include the use of a potentially anti-pyretic drug in the definition of fever). In this sensitivity analysis, those who never report fever and never report use of anti-pyretic medications will be assigned duration of fever of zero days.

*Oxygen Saturation ≥ 96%*

1) Repeat primary analysis, but impute missing data in the following manner (ignores missingness due to hospitalization and death):
   - For non-monotonic missingness, participants with missing oxygen saturation results will have their values imputed as ≥96% if the preceding and succeeding results are ≥96%, otherwise the results will be imputed as <96%.
   - For monotonic missingness, inverse probability weighted GEE will be used.
2) Repeat primary analysis, but impute missing data in the following manner (special considerations for missingness due to hospitalization and death):
   - For missingness due to hospitalization or death, participants with missing oxygen saturation results will have their values imputed as <96%.
   - For non-monotonic missingness, participants with missing oxygen saturation results will have their values imputed as ≥96% if the preceding and succeeding results are ≥96% , otherwise the results will be imputed as <96%.
   - For monotonic missingness, inverse probability weighted GEE will be used.

<u>Supportive Analyses</u>

*COVID-19 Severity Ranking*

To evaluate the effect of the investigational agent on COVID-19 symptom severity over different time-periods, analyses of COVID-19 severity ranking based on partial AUCs will also be examined. The time-periods considered include day 0 to day 7, day 0 to day 14, and day 0 to day 21. These analyses will compare participant specific AUCs between randomized arms using a two-sided Wilcoxon test with a 5% type I error rate. In addition, Hodges-Lehmann estimate and associated 95% CI for the location shift between the two arms will also be provided.

For each time period, for participants who are alive and were never hospitalized in that time period (i.e., as of 7 days, 14 days, and 21 days), the severity ranking will be based on their AUC of the symptom score associated with COVID-19 disease over time (through day 7, 14, 21, respectively, counting day 0 as the first day) assigned as the sum of scores for the targeted symptoms in the participant's study diary. The AUCs will be calculated using the trapezoidal rule and is defined as the area below the line formed by joining total symptom scores on each daily diary card from day 0 through day 7, 14, and 21, respectively. The AUCs will be rescaled by time in order to provide results on a symptom scale from 0 to 39. This will be done by dividing the AUC by 7, 14, or 21, respectively, corresponding to the number of trapezoids created from daily diary cards between day 0 and the last day considered in the calculation (i.e., day 7, day 14, and day 21).

Participants who die or are hospitalized in the time interval being considered (through day 7, day 14, or day 21, respectively) will be ranked as worse (i.e., worse severity) than those alive and never hospitalized in worsening rank order. Programmatically, participants who die in the time interval will be assigned an AUC (severity score) of 42 (worst rank) regardless of when the death occurred in the interval, participants who are alive but remain hospitalized at last day of the interval will be assigned an AUC (severity score) of 41 (second worst rank), and participants who

are alive but are no longer hospitalized on the last day of the interval will be assigned an AUC (severity score) of 40 (the third worst rank).

Participants who have incomplete diary cards for reasons other than hospitalization or death will be addressed in the same manner as the analyses of COVID-19 severity through day 28, outlined in the above section of the SAP.

*Oxygen Saturation*

The primary analysis will be repeated without adjustment for baseline oxygen saturation level. In addition, the absolute difference in proportion of participants with oxygen saturation ≥ 96% will be calculated at each measurement time, with associated 95% confidence intervals (calculated using the normal approximation to the binomial distribution).

Subgroup Analyses

*Duration of Clinical Symptoms*

In phase III, to evaluate the effect of the investigational agent on symptom duration in specific populations (address secondary objective 8), secondary outcome 4 will be assessed among different subgroups. Descriptive analyses for the following subgroups will be considered. A separate analysis plan for multivariate/personalized-medicine type analyses across subgroups will be developed at a later time.

Pre-specified subgroups of interest include:

1) Sex (Male sex at birth, female sex at birth)
2) Race (white, non-white)
3) Ethnicity (Hispanic, non-Hispanic)
4) 'Risk of Severe Disease' Stratification (<55 years and no comorbidities, ≥ 55 years or at least one comorbidity)
5) Age Group (<55, ≥55)
6) Co-morbidity Status (no comorbidities, at least one comorbidity)
7) Calendar days from first symptom associated with COVID-19 to start of investigational agent/placebo Stratification (≤ 5 days, > 5 days)
8) Site (if applicable) or site location (if applicable)

   Subgroup analyses by site will be considered if there are a limited number of sites that contributed to enrollment. Otherwise, subgroup analyses by site location (e.g. by country or region) will be conducted if non-US sites contribute to enrollment.

### 5.2.2 Secondary Virology

Analysis Population

The analyses of the virology objectives will include all randomized participants who started an investigational agent or the concurrent placebo, according to a modified intent-to-treat (mITT)

approach (Treated Population). Participants who have protocol violations will be included in the analysis but the protocol violations will be documented and described.

Note: Participants who are randomized but do not start investigational agent or placebo are, per protocol, not to be followed and will be replaced.

Analysis Methods

*Detection (Detectable vs Undetectable) of SARS-CoV-2 RNA*

Descriptive statistics (number and percentage) will be used to describe the proportion of participants with undetectable SARS-CoV-2 RNA at each scheduled measurement time. Analysis will be conducted separately for each specimen type (i.e., anterior nasal swabs and saliva).

The proportion of participants with undetectable SARS-CoV-2 RNA will be compared between randomized arms using log-binominal regression for repeated binary measurements with log-link. This model will be fitted using generalized estimating equations (GEE) to handle the repeated measurements with an independence working correlation structure and robust standard errors. For each time point after starting treatment, the model will include a main effect for time (indicator variable for each evaluation time), an interaction between time and randomized arm to evaluate differences between arms, and will adjust for baseline (day 0) log-10 transformed SARS-CoV-2 RNA level. The estimated adjusted relative risk of being undetectable (and associated 95% CI) will be obtained for each measurement time from the model by taking the exponential of the time*randomized arm interaction parameter estimate (and associated 95% CI) for that measurement time. In the event the log-binomial regression model fails to converge, a Poisson regression model with robust variance and log-link will be used instead.

In addition, a joint test of randomized arm across the time points will also be assessed, with degrees of freedom determined by the number of time points included. With this model, the comparison between randomized arms will use a two-sided Wald-test with 5% type I error rate. Time points with zero events in either arm will not be included in the model (as estimation for such a model may be problematic; however data for these time points will be included in a descriptive summary of results over time points).

Missing data are assumed to be missing completely at random (MCAR) and will be ignored in these analyses; however, sensitivity analyses will address possible informative missingness (see below).

In this analysis, baseline SARS-CoV-2 RNA values will be imputed as outlined in section 4.1. It is not expected that a high proportion of baseline results will be < LLoQ; however, in the event that there is a non-negligible amount of censoring (defined as 10% or more of baseline results < LLoQ), an additional variable will be added to the model that will indicate whether the baseline result was above or below assay lower quantification limit (included programmatically as “0” if above LLoQ, and “1” if below LLoQ).

*Level (Quantitative) of SARS-CoV-2 RNA*

Descriptive statistics will be used to describe the levels of SARS-CoV-2 RNA at each scheduled measurement time. Analysis will be conducted separately for each specimen type (i.e., NP swabs, anterior nasal swabs and saliva).

Non-parametric Wilcoxon rank-sum tests with a 5% type I error rate will compare SARS-CoV-2 RNA level (continuous) between randomized arms, separately at each post-entry study day; results below the limit of detection will be imputed as the lowest rank and values above the limit of detection but below the LLoQ will be imputed as the second lowest rank. In addition, Hodges-Lehmann estimate and associated 95% CI for the location shift between the two arms will also be provided.

Missing data in analysis of continuous SARS-CoV-2 RNA levels are assumed to be missing completely at random (MCAR) and will be ignored in analysis.

*AUC of SARS-CoV-2 RNA*

Levels of log-10 transformed SARS-CoV-2 RNA, measured from NP swabs, anterior nasal swabs, and saliva will be analyzed using participant-specific AUCs; this will be done separately for each specimen type. In this analysis, the AUC is defined as the area below the line formed by joining measured values at each successive measurement time and above the lower limit of quantification of the assay, calculated using trapezoidal rule. Programmatically, the trapezoidal rule will be applied to the following values: $\max[0, \log_{10}(\text{RNA})-\log_{10}(\text{LLoQ})]$, obtained at the scheduled measurement times between and including day 0 and day 28.

Missing values with preceding and succeeding values will be ignored, which is equivalent to linearly interpolating the RNA levels from preceding and succeeding values. Missing values with no succeeding values will be imputed using linear imputation assuming that the RNA level at day 28 equals the LLoQ (as it is anticipated that nearly everyone will clear virus over 28 days). If the day 0 result is missing then the participant will be excluded from analysis. The participant-specific AUCs will be compared between randomized arms using a two-sided Wilcoxon test with 5% type I error rate. In addition, Hodges-Lehmann estimate and associated 95% CI for the location shift between the two arms will also be provided.

Missing data in the AUC analysis of continuous SARS-CoV-2 RNA levels are assumed to be missing completely at random (MCAR) and will be ignored in analysis.

<u>Sensitivity Analyses</u>

The following sensitivity analyses are included to evaluate impact of different assumptions on the inference of the virology outcomes.

*All Virology Outcomes*

1) Repeat primary analysis, but restrict analysis population to exclude those with undetectable SARS-CoV-2 RNA at Day 0.

*Dichotomous Virology Outcomes*

1) Repeat primary analysis, but impute missing data in the following manner (ignores missingness due to hospitalization and death):
    - For non-monotonic missingness, participants with missing SARS-CoV-2 results will have their values imputed as undetectable if the preceding and succeeding results are undetectable, otherwise the results will be imputed as detectable.
    - For monotonic missingness, inverse probability weighted GEE will be used
2) Repeat primary analysis, but impute missing data in the following manner (special considerations for missingness due to hospitalization and death):
    - For missingness due hospitalization or death, participants with missing SARS-CoV-2 results will have their values imputed as *detectable*.
    - For non-monotonic missingness, participants with missing SARS-CoV-2 results will have their values imputed as *undetectable* if the preceding and succeeding results are undetectable, otherwise the results will be imputed as *detectable*.
    - For monotonic missingness, inverse probability weighted GEE will be used.

<u>Supportive Analysis</u>

The dichotomous virology analysis will be repeated without adjustment for baseline (Day 0) SARS-CoV-2 RNA level. In addition, the absolute difference in proportion of participants with undetectable levels will be calculated at each measurement time, with associated 95% confidence intervals (calculated using the normal approximation to the binomial distribution).

## 5.3 Exploratory Analyses

### 5.3.1 New SARS-CoV-2 among Household Contacts

The analysis of household contacts will include all randomized participants who started an investigational agent or the concurrent placebo, and will restricted to participants who report that they share indoor living space or housekeeping space with someone.

New SARS-CoV-2 positivity among household contacts through day 28 will be analyzed in the following manner. The proportion of participants with a household contact that tests positive for SARS-CoV-2 after the participant initiates study investigational agent or concurrent placebo through day 28, will be estimated and compared between randomized arms using log-binomial regression, with log link, in order to obtain a risk ratio estimate; the model will include a main effect for randomized arm. In the event the log-binomial regression model fails to converge, a Poisson regression model with robust variance and log-link will be used instead. Missing data will be considered ignorable in analysis. The same analysis approach will be used to compare the proportion of participants with a household contact that tests positive for SARS-CoV-2 or has COVID-19 symptoms after the participant initiates study investigational agent or concurrent placebo through day 28.

Analysis of new SARS-CoV-2 positivity, and SARS-CoV-2 positivity or COVID-19 symptoms, among household contacts through week 24 will be analyzed as in the same way as above for these outcomes through day 28.

#### 5.3.2 Hospitalization Course

Analyses of clinical outcomes among those hospitalized will include all randomized participants who started an investigational agent or the concurrent placebo who were also hospitalized. The analyses will be limited to descriptive summaries by randomized arm, as these analyses are restricted to participants who were hospitalized and so are not randomized comparisons.

Duration of hospitalization and duration of ICU admission will be summarized with continuous descriptive statistics. Duration of hospitalization/ICU through day 28 will be calculated as the difference between the date of discharge and the date of admission; the duration will be truncated at Day 28, if the participant is still hospitalized at Day 28. If data on discharge dates occurring after Day 28 are complete at the time of analysis of the Day 28 data, an additional descriptive analysis of durations for hospitalizations starting on or before Day 28 will be undertaken. The proportion of participants with ICU admission, among those hospitalized, will be summarized with frequencies and percentages. The worst clinical status (ordinal outcome) will be summarized with frequencies and percentages. Descriptive summaries of use of remdesivir and dexamethasone, and other approved medications for treatment of COVID-19 used during hospitalization will also be included.

This analysis will be done through day 28 and separately through week 24.

#### 5.3.3 Exploratory Virology

The analysis of SARS-CoV-2 RNA in blood (plasma) will be done in the same manner as the secondary analysis of SARS-CoV-2 RNA from saliva and nasal swabs. See section 5.2.2 for details.

#### 5.3.4 Resistance Mutations

Analyses addressing the emergence of new resistance mutations will be outlined for each investigational agent in agent-specific SAP appendices.

### 5.4 Interim Analysis Considerations

#### 5.4.1 Phase II to Phase III Graduation Criteria

Each investigational agent considered in phase II will be evaluated for graduation to phase III. Graduation will be based on there being a desired level of evidence of an effect of an investigational agent versus placebo on one or more virologic and clinical outcome measures, as well as consideration of safety. The plan for these analyses will be provided in a separate document.

#### 5.4.2 Phase III Statistical Considerations

The DSMB will review interim data from the study including descriptive summaries of study conduct and adverse events, and efficacy analyses that contrast randomized arms. The primary outcome of death or hospitalization will be compared between groups using the statistical methods outlined in this SAP; the secondary outcome of death from any cause will be also be compared between randomized arms. Interim efficacy analyses are planned when approximately

500, 1000, and 1500 participants have been followed for the primary outcome assessed at day 28, or earlier if the total number of hospitalizations or deaths is higher than anticipated (See SAP Section 2.5 or Protocol Section 10.5).

At each interim review, the stopping boundary for the primary analysis will be determined based on the proportion of planned maximum information that is available at the given review. The statistical information (Fisher's Information) at a given review will be calculated using the inverse of the variance (square of standard error) obtained from Greenwood's formula as part of the primary analysis. The maximum information will be pre-determined using the following formula (Tsiatis AA. Statistics in medicine. 2006 Oct 15;25(19):3236-44):

$$MI = \left\{\frac{\left(Z_{\alpha/2}+Z_{\beta}\right)^2}{\delta_A^2}\right\} * (Inflation\ Factor) = \frac{(1.96+1.28)^2}{\left\{\ln\left(\frac{0.10}{0.15}\right)\right\}^2} * 1.03 = 65.8.$$

As a sensitivity analysis, we will assume all participants who prematurely discontinue the study prior to day 28, who are unable to be contacted by the site to ascertain outcomes after discontinuation, had a primary event one day after the date they were lost to follow up. If the interpretation of the results from the primary analysis and this sensitivity analysis are substantially different, then considerations of the potential impact of delayed ascertainment of the primary endpoint will be considered, using an approach suggested by a DSMB statistician (personal correspondence A.A. Tsiatis).

# 6 Appendix 1: Statistical Considerations for LY3819253

## 6.1 Phase II

Emergence of Resistance Mutations

Four potential escape mutations were pre-identified for LY3819253: E484K, E484Q, E490S, and S494P. The laboratory conducting the resistance testing has stated that a mutation will be considered as present for a particular NP sample if at least 20% of the viral population in that sample have the mutation.

The emergence of resistant mutations after starting treatment will be summarized in the following manner. Descriptive statistics (number and percent) will be use to describe the proportion of participants with quantifiable (≥ LLoQ) SARS-CoV-2 RNA at Day 0 from NP swabs. Among those with and without quantifiable RNA at Day 0 from NP swabs, descriptive statistics will summarize the number and percent with quantifiable RNA during follow up from NP, and whether the participants developed new resistance mutations during follow up. The number and percent of mutations present at Day 0 will also be summarized.

The proportion of participants who have a new resistance mutation after study entry will be estimated and compared between randomized arms using log-binomial regression, with log link, in order to obtain a risk ratio estimate; the model will include a main effect for randomized arm. In the event the log-binomial regression model fails to converge, a Poisson regression model with robust variance and log-link will be used instead. Note: this analysis assumes that those who were <LLoQ at Day 0 and stayed <LLoQ, and those who were <LLoQ throughout follow-up, did not develop resistance.

A supportive analysis will be exclude participants who had all mutations present at Day 0, as they could not develop new resistance.

Phase II primary virology analyses will include an additional subgroup analysis: Presence of Resistance Mutations (yes, no).

## 6.2 Phase III

After fully enrolling phase II (approximately 110 participants receiving LY3819253 and 110 receiving placebo), the LY3819253 agent will move directly (without graduation analysis of phase II data) into phase III as an open-label, single-arm, evaluation. There is no randomization in phase III. Enrollment into phase III will continue until another investigational agent enters the study; at this point phase III evaluation of LY3819253 will close.

Because of the single arm nature of the phase III evaluation, all phase III analyses will be descriptive using the same definitions for outcome measures and handling of missing data as described in the SAP. No formal analysis comparing participants who enrolled in the single arm Phase III component of the study with participants who participated in Phase II will be undertaken. If summaries are done by subgroups, and if available, summaries will also be done by whether there is presence of resistance mutations (yes, no).

### 6.3 Phase II: Exploratory Analysis Pooling Over the 7000mg and 700mg Doses

Analyses of LY3819253 will be done separately by dose (7000mg and 700mg), since each is considered as a separate agent in ACTIV-2 per protocol. In support of these analyses, exploratory analyses that pool across doses may also be considered (pooled 7000mg+700mg active vs pooled placebos for 7000mg+700mg).
