# Supplementary material for: Antiviral and clinical activity of bamlanivimab in a randomized trial of non-hospitalized adults with COVID-19
Source: Nat Commun. 2022 Aug 22;13:4931. doi: 10.1038/s41467-022-32551-2 (PMC9395368; doi:10.1038/s41467-022-32551-2)
Supplement: Supplementary file 3 — Reporting Summary [file 41467_2022_32551_MOESM3_ESM.pdf]

nature portfolio

Corresponding author(s): Kara Chew

Last updated by author(s): July 21, 2022

Reporting Summary

Nature Portfolio wishes to improve the reproducibility of the work that we publish. This form provides structure for consistency and transparency in reporting. For further information on Nature Portfolio policies, see our Editorial Policies and the Editorial Policy Checklist.

Statistics

For all statistical analyses, confirm that the following items are present in the figure legend, table legend, main text, or Methods section.

n/a	Confirmed	
☐	☒	The exact sample size (*n*) for each experimental group/condition, given as a discrete number and unit of measurement
☐	☒	A statement on whether measurements were taken from distinct samples or whether the same sample was measured repeatedly
☐	☒	The statistical test(s) used AND whether they are one- or two-sided<br>*Only common tests should be described solely by name; describe more complex techniques in the Methods section.*
☐	☒	A description of all covariates tested
☐	☒	A description of any assumptions or corrections, such as tests of normality and adjustment for multiple comparisons
☐	☒	A full description of the statistical parameters including central tendency (e.g. means) or other basic estimates (e.g. regression coefficient) AND variation (e.g. standard deviation) or associated estimates of uncertainty (e.g. confidence intervals)
☐	☒	For null hypothesis testing, the test statistic (e.g. *F*, *t*, *r*) with confidence intervals, effect sizes, degrees of freedom and *P* value noted<br>*Give P values as exact values whenever suitable.*
☒	☐	For Bayesian analysis, information on the choice of priors and Markov chain Monte Carlo settings
☒	☐	For hierarchical and complex designs, identification of the appropriate level for tests and full reporting of outcomes
☐	☒	Estimates of effect sizes (e.g. Cohen's *d*, Pearson's *r*), indicating how they were calculated

Our web collection on statistics for biologists contains articles on many of the points above.

Software and code

Policy information about availability of computer code

Data collection	No software were used to collect data.
Data analysis	All analyses were performed using code available from standard software packages. Mathematical modeling of viral kinetics was performed using Monolix 2020 (Lixoft, Antony, France). PK parameters were calculated using WinNonLin v8.3.4, Certara (Princeton, NJ, USA). Sequencing was performed on the Illumina MiSeq platform and deep sequencing data analysis was carried out using the Stanford Coronavirus Antiviral & Resistance Database (CoVDB) platform (https://covdb.stanford.edu/sierra/sars2/by-reads/?cutoff=0.01&mixrate=0.01). Input FASTQ sequence alignment with Wuhan-Hu-1 reference was done using MiniMap2 version 2.22 in CodFreq pipeline (https://github.com/hivdb/codfreq). The output of MiniMap2, an aligned SAM file, is converted to a CodFreq file by an in-house written Python script using a PySam library (version: 0.18.0) and further analyzed with the CoVDB. SARS-COV-2 variant calling was done using 3 different variant calling platforms, namely, CoVDB27, Scorpio call v1.2.123 (https://pangolin.cog-uk.io/) and Nextclade v.1.13.2 (https://clades.nextstrain.org/). Statistical analyses were conducted using SAS version 9.4 and R version 4.1.0.

For manuscripts utilizing custom algorithms or software that are central to the research but not yet described in published literature, software must be made available to editors and reviewers. We strongly encourage code deposition in a community repository (e.g. GitHub). See the Nature Portfolio guidelines for submitting code & software for further information.

March 2021

Data

Policy information about availability of data

All manuscripts must include a data availability statement. This statement should provide the following information, where applicable:

- Accession codes, unique identifiers, or web links for publicly available datasets
- A description of any restrictions on data availability
- For clinical datasets or third party data, please ensure that the statement adheres to our policy

The following publicly available databases were used for viral sequence analysis in this study: Stanford Coronavirus Antiviral & Resistance Database (CoVDB) platform (https://covdb.stanford.edu/sierra/sars2/by-reads/?cutoff=0.01&mixrate=0.01), MiniMap2 version 2.22 in CodFreq pipeline (https://github.com/hivdb/codfreq), Scorpio call v1.2.123 (https://pangolin.cog-uk.io/) and Nextclade v.1.13.2 (https://clades.nextstrain.org/). The next-generation sequencing data generated in this study have been deposited on the NCBI Short Read Archive (SRA) under accession number PRJNA816433 [https://www.ncbi.nlm.nih.gov/bioproject/816433] and PRJNA859660 [https://www.ncbi.nlm.nih.gov/bioproject/859660]. Other data are available under restricted access due to ethical restrictions. Access can be requested by submitting a data request at https://submit.mis.s-3.net/ and will require the written agreement of the AIDS Clinical Trials Group (ACTG) and the manufacturer of the investigational product. Requests will be addressed as per ACTG standard operating procedures. Completion of an ACTG Data Use Agreement may be required. Aggregate data generated in this study are provided in the Source Data file.

Field-specific reporting

Please select the one below that is the best fit for your research. If you are not sure, read the appropriate sections before making your selection.

☒ Life sciences ☐ Behavioural & social sciences ☐ Ecological, evolutionary & environmental sciences

For a reference copy of the document with all sections, see nature.com/documents/nr-reporting-summary-flat.pdf

Life sciences study design

All studies must disclose on these points even when the disclosure is negative.

Sample size	The sample size of 110 participants randomized to each arm was selected to give high power to identify an active agent based on the primary virologic outcome. At the time the study was designed, there were no data in outpatients with COVID-19 to inform expected differences in proportion undetectable for NP SARS-CoV-2 RNA over 28 days. We estimated that a 20% absolute increase in the proportion undetectable would be clinically relevant, and 110 participants assigned to each arm would have 82.5 to 95.5% power dependent on the proportion undetectable in the placebo arm, with a two-sided Type I error rate of 5%.
Data exclusions	No data were excluded from analyses
Replication	Virologic and serum and plasma biomarkers were performed using validated assays in CLIA-certified laboratories. All assays were performed once only.
Randomization	Participants were randomized 1:1 to active versus placebo arm. Randomization was stratified by time from symptom onset (<= or >5 days) and risk of progression to severe COVID-19 (higher vs lower).
Blinding	Site staff, with the exception of unblinded pharmacists, and investigators were blind to randomized treatment.

Reporting for specific materials, systems and methods

We require information from authors about some types of materials, experimental systems and methods used in many studies. Here, indicate whether each material, system or method listed is relevant to your study. If you are not sure if a list item applies to your research, read the appropriate section before selecting a response.

Materials & experimental systems

n/a	Involved in the study	
☒	☐	Antibodies
☒	☐	Eukaryotic cell lines
☒	☐	Palaeontology and archaeology
☒	☐	Animals and other organisms
☐	☒	Human research participants
☐	☒	Clinical data
☒	☐	Dual use research of concern

Methods

n/a	Involved in the study	
☒	☐	ChIP-seq
☒	☐	Flow cytometry
☒	☐	MRI-based neuroimaging

Human research participants

Policy information about studies involving human research participants

Population characteristics	Participant characteristics are summarized in Table 1.
Recruitment	Participants were recruited through a variety of mechanisms including a study-specific website with IRB-approved content describing the study. The website listed a telephone number that connected to a 24 hour call center staffed by English and Spanish speaking operators who followed an IRB-approved script to pre-screen callers for basic eligibility (e.g., recent diagnosis of COVID-19, age of 18 and older) and then connected callers to the nearest study site. In addition, digital marketing was conducted using the paid search services of the Google search engine such that IRB-approved advertisements for the trial were displayed when searching key words (e.g., COVID-19 treatment, COVID-19 treatment trial). IRB-approved study advertisements were also placed periodically on Facebook and Instagram. Persons testing COVID-19 positive at testing venues associated with the clinical laboratories Covance and Quest, operated by eTrueNorth Inc, or partnered with Verily Life Sciences or the PPD Accelerated Enrollment Solutions (AES) and who opted-in to receive information regarding research opportunities received IRB-approved messages or calls describing the study. Study sites also conducted their own outreach including the circulation of IRB-approved brochures, postcards, and flyers at COVID-19 testing centers and engagement of local providers. Lastly, the trial was listed on the public access website www.clinicaltrials.gov. Participants who feel less well or with risk factors for severe COVID-19 or already engaged in care may be more likely to seek participation in a clinical trial of COVID-19 treatment, but as described above extensive efforts were made to recruit a diverse population, including those without ready or established access to care, and the recruited population was ultimately diverse in geographic location, clinical research site type (academic or community), demographics, and symptom burden. Participant compensation was approved prior to participant accrual by central and local IRBs as required for each site.
Ethics oversight	Ethics oversight was performed by a central IRB (Advarra). In addition to central IRB approval, the following institutions' IRB also reviewed and approved the protocol: Case Western Reserve University, Weill Cornell Medicine, Johns Hopkins University, Rush University Medical Center, and Vanderbilt University. All other participating sites obtained central IRB approval only or the institution agreed to rely on Advarra review.

Note that full information on the approval of the study protocol must also be provided in the manuscript.

Clinical data

Policy information about clinical studies

All manuscripts should comply with the ICMJE guidelines for publication of clinical research and a completed CONSORT checklist must be included with all submissions.

Clinical trial registration	NCT04518410
Study protocol	The protocol is provided in Supplementary Information.
Data collection	Participating sites are provided in Supplementary Information. Participants were recruited between August and November 2020. Participants were followed for 24 weeks.

Outcomes

Primary and secondary outcome measures and the analysis approach are described in the protocol and Statistical Analysis Plan (provided in Supplementary Information).

This phase 2 study was designed to evaluate the safety of bamlanivimab and determine the efficacy of bamlanivimab to reduce the duration of COVID-19 symptoms and SARS-CoV-2 RNA shedding from NP swabs. NP swabs were collected on days 0 (day of study intervention, pre-intervention), 3, 7, 14, 21, and 28. Participants completed a study diary each day from day 0 to day 28, which included self-assessment of 13 targeted COVID-19 symptoms, scored by the participant as absent, mild, moderate, or severe (see Supplementary Methods for symptom diary). A numerical total symptom score was calculated for each day by summing scores for each symptom, with absent scored as 0, mild as 1, moderate as 2, and severe as 3; therefore, the range of total symptoms scores was 0 to 39. Clinical assessments for adverse events were conducted at days 0, 2, 3, 7 10, 14, 21, 28, week 12, and week 24. Safety laboratories were performed at days 0, 3, 14, and 28, and included complete blood cell count with automated differential and platelet count, liver, and kidney function tests. All safety laboratories were performed at a central laboratory, PPD® Laboratory Services Global Central Labs. Adverse events including laboratory values were assessed and graded using the Division of AIDS Table for Grading the Severity of Adult and Pediatric Adverse Events, corrected Version 2.1, July 2017 (https://rsc.niaid.nih.gov/clinical-research-sites/daids-adverse-event-grading-tables).

Primary outcome measures were: 1) development of a Grade 3 or higher treatment-emergent adverse event (TEAE) through 28 days; 2) detection (detectable versus undetectable) of SARS-CoV-2 RNA from NP swabs at days 3, 7, 14, 21, and 28; 3) duration of targeted COVID-19-associated symptoms from day 0 (utilizing daily diary data), where duration was defined as the number of days from day 0 to the last day on or before study day 28 when any targeted symptoms that were self-assessed as moderate or severe at day 0 (before study intervention) were still scored as moderate or severe (i.e., not mild or absent), or any targeted symptoms scored as mild or absent at day 0 were still scored as mild or worse (i.e. not absent). Participants with ongoing unimproved symptoms at day 28 were treated as having a symptom duration of 28 days for analysis.

Secondary outcome measures included all-cause hospitalization and death, quantitative NP SARS-CoV-2 RNA levels; area under the curve (AUC) of symptom scores from days 0-28; and change in inflammatory markers from baseline through week 24; development of AEs of special interest (AESIs, specifically Grade 1 or higher infusion-related reactions [IRRs] and Grade 1 or higher allergic/hypersensitivity reactions), and serious adverse events (SAEs) through day 28 and through week 24; progression of 1 or more COVID-19-associated symptoms to a worse status than recorded in the study diary at entry; and PK measures including area under the concentration-time curve (AUC), total body clearance (CL), elimination half-life (T1/2), and maximum and minimum

concentrations (Cmax, Cmin).

Inflammatory and coagulation markers assessed were lactate dehydrogenase (LDH), C-reactive protein (CRP), ferritin, D-dimer, prothrombin time (PT)/international normalized ratio (INR), activated partial thromboplastin time (PTT), and fibrinogen. All markers were measured in real time by a central clinical laboratory (PPD® Laboratory Services Global Central Labs) at days 0, 7, 14, 21, and 28 and weeks 12 and 24, per the manufacturers' protocols.

Blood samples for quantitation of bamlanivimab serum concentrations were collected pre-dose and at the following times after the end of infusion: 30 minutes, days 14 and 28 and weeks 12 and 24. Serum concentrations of bamlanivimab were determined using a validated hybrid LC-MS/MS method. that has met FDA requirements for accuracy and reproducibility. PK parameters were calculated based on the statistical moment theory using the trapezoidal rule and linear regression (WinNonLin v8.3.4, Certara, Princeton, NJ, USA).

Statistical Analysis
The analysis population included all participants who initiated study intervention (bamlanivimab or placebo). Four participants enrolled to the 7000 mg dose cohort received 700 mg bamlanivimab or placebo and were included in the 700 mg analysis population (the randomization to active agent or placebo remained valid). One participant enrolled in the 700 mg dose cohort received 7000 mg bamlanivimab and was included in the 7000 mg analysis population.

The proportion of participants experiencing a grade 2 or higher and grade 3 or higher TEAE was compared between arms using log-binomial regression and summarized with a risk ratio (RR), corresponding 95% CI, and p-value based on the Wald test. The proportion of participants with undetectable SARS-CoV-2 RNA was compared between arms across study visits using Poisson regression with robust variance adjusted for baseline (day 0) log10 transformed SARS-CoV-2 RNA level and summarized with RR and 95% CI at each time, and Wald test across the multiple times. Quantitative SARS-CoV-2 RNA levels were compared between arms using Wilcoxon rank-sum tests, separately at each post-entry study visit, without adjustment for baseline value. For this comparison, results below the LoD were imputed as the lowest rank and values above the LoD but below the LLoQ were imputed as the second lowest rank. For summaries of quantitative RNA levels, values below the LoD were imputed as 0.7 log10 copies/ml (i.e., half the distance from zero to the LoD), values above the LoD but below the LLoQ were imputed as 1.7 log10 copies/ml (i.e., half the distance from the LoD to the LLoQ), and values above the ULoQ were imputed as 8 log10 copies/ml if a numerical value was not available.

The two dose cohorts (700 and 7000 mg) were combined for post-hoc exploratory analyses of baseline NP, AN, and plasma SARS-CoV-2 RNA levels and modeling of viral decay. Spearman correlations evaluated associations between total symptoms scores and NP and AN RNA levels, Wilcoxon tests and chi-square tests were used to evaluate NP and AN RNA levels and symptom scores between subgroups. The rates of decline of NP and AN virus after study entry were quantified in separate models using Monolix 2020 (Lixoft, Antony, France). Methods for model fitting and selection are described in Supplementary Methods.

Participant-specific symptom durations and area under the curve (AUC) of total symptom score from days 0-28 were compared between arms using a Wilcoxon rank sum test. Due to the small number of hospitalization/death events, the proportion hospitalized/dead in the bamlanivimab and placebo arms was summarized with descriptive statistics and compared between arms using Fisher's exact test as a post-hoc analysis. Change from baseline in log-transformed inflammatory and coagulation biomarker levels were compared between bamanivimab and placebo arms using Wilcoxon tests.
